# Defining, identifying and addressing problematic polypharmacy within multimorbidity in primary care: a scoping review

**DOI:** 10.1136/bmjopen-2023-081698

**Published:** 2024-05-24

**Authors:** Jung Yin Tsang, Matthew Sperrin, Thomas Blakeman, Rupert A Payne, Darren Ashcroft

**Affiliations:** 1 Centre for Primary Care and Health Services Research, School of Health Sciences, The University of Manchester Division of Population Health Health Services Research and Primary Care, Manchester, UK; 2 NIHR Greater Manchester Patient Safety Research Collaboration (GMPSRC), Faculty of Biology, Medicine and Health, Manchester Academic Health Sciences Centre (MAHSC), The University of Manchester, Manchester, UK; 3 Division of Informatics, Imaging and Data Sciences, School of Health Sciences, The University of Manchester, Manchester, UK; 4 Department of Health and Community Sciences, University of Exeter Medical School, Exeter, UK; 5 Division of Pharmacy and Optometry, School of Health Sciences, Faculty of Biology Medicine and Health, The University of Manchester, Manchester, UK

**Keywords:** drug combinations, chronic disease, adverse events, primary health care, primary prevention, medication adherence

## Abstract

**Introduction:**

Polypharmacy and multimorbidity pose escalating challenges. Despite numerous attempts, interventions have yet to show consistent improvements in health outcomes. A key factor may be varied approaches to targeting patients for intervention.

**Objectives:**

To explore how patients are targeted for intervention by examining the literature with respect to: understanding how polypharmacy is defined; identifying problematic polypharmacy in practice; and addressing problematic polypharmacy through interventions.

**Design:**

We performed a scoping review as defined by the Joanna Briggs Institute.

**Setting:**

The focus was on primary care settings.

**Data sources:**

Medline, Embase, Cumulative Index to Nursing and Allied Health Literature and Cochrane along with ClinicalTrials.gov, Science.gov and WorldCat.org were searched from January 2004 to February 2024.

**Eligibility criteria:**

We included all articles that had a focus on problematic polypharmacy in multimorbidity and primary care, incorporating multiple types of evidence, such as reviews, quantitative trials, qualitative studies and policy documents. Articles focussing on a single index disease or not written in English were excluded.

**Extraction and analysis:**

We performed a narrative synthesis, comparing themes and findings across the collective evidence to draw contextualised insights and conclusions.

**Results:**

In total, 157 articles were included. Case-finding methods often rely on basic medication counts (often five or more) without considering medical history or whether individual medications are clinically appropriate. Other approaches highlight specific drug indicators and interactions as potentially inappropriate prescribing, failing to capture a proportion of patients not fitting criteria. Different potentially inappropriate prescribing criteria also show significant inconsistencies in determining the appropriateness of medications, often neglecting to consider multimorbidity and underprescribing. This may hinder the identification of the precise population requiring intervention.

**Conclusions:**

Improved strategies are needed to target patients with polypharmacy, which should consider patient perspectives, individual factors and clinical appropriateness. The development of a cross-cutting measure of problematic polypharmacy that consistently incorporates adjustment for multimorbidity may be a valuable next step to address frequent confounding.

**STRENGTHS AND LIMITATIONS OF THIS STUDY**

- This is the first scoping review to explore and conceptualise how patients with problematic polypharmacy are targeted for intervention
- It includes multiple types of evidence, including systematic reviews, quantitative, qualitative and mixed methods studies, along with policy documents.
- Our synthesis capitalises on the shared challenges involved in managing both polypharmacy and multimorbidity with a greater focus on articles regarding polypharmacy in chronic conditions rather than acute medication adjustments.
- It was not always possible to separate results in studies encompassing both primary and secondary care.

## Introduction

Polypharmacy in multimorbidity is an increasing global priority.^1^ With an ageing population, over a quarter of the population are living with multiple long-term conditions also known as multimorbidity.^1^ This is often associated with polypharmacy, which is broadly defined as the use of multiple medications.^2^ Medications carry clear benefits, yet the use of multiple medicines can be linked to adverse consequences, including increased treatment burden, unplanned hospitalisation and death.^3 4^ For single conditions, people with more severe disease often require more medications. For example, the National Institute for Health and Care Excellence (NICE) guidelines recommend six medicines to be initiated post myocardial infarction for secondary prevention.^5^ Yet in multimorbidity, the number of medicines quickly add up, with limited evidence of benefit over risk as this population is frequently excluded in trials.^6^ As the number of medicines prescribed increases, so does the direct risk of adverse drug reactions, increasing health service costs and utilisation, reducing adherence and decreasing quality of life.^7–9^ This can be particularly problematic for older patients, for whom prescribing is more common and thus associated with greater possibility of prescribing errors. Moreover, the risks of harms are increased due to changes in pharmacokinetics (eg, impaired drug metabolism, changes in drug binding) and pharmacodynamics (eg, increased sensitivity to adverse effects).^10–12^ Problematic polypharmacy has previously been defined as ‘the prescribing of multiple medications inappropriately, or where the intended benefit of the medication is not realised’.^3^

Despite numerous interventions targeting polypharmacy, there remains little evidence of improvement of health outcomes, such as hospitalisations and death.^13–15^ However, some reductions in inappropriate prescribing have been observed. Successes of these interventions have been highly variable and greatly affected by differences in implementation and targeting of patients.^13–15^ Further conceptualising the complex and varied approaches to targeting patients with problematic polypharmacy and multimorbidity may inform empirical research and improve future intervention design.^2^ Therefore, a scoping review was performed, to adopt an effective approach for assessing a broad evidence base. This review centres on considering the pivotal role of primary care professionals and capitalises on the shared challenges involved in managing polypharmacy and multimorbidity. The overarching aim of the review was to explore how patients are targeted for intervention by examining the literature with respect to (1) understanding how polypharmacy is defined; (2) identifying problematic polypharmacy in practice; and (3) addressing problematic polypharmacy through interventions.

## Methods

A scoping review as defined by the Joanna Briggs Institute was performed consistent with the Preferred Reporting Items for Systematic Reviews extension for Scoping Reviews (PRISMA-ScR) guidance.^16^ This allowed an exploration of both breadth and depth of the topic, which was imperative given the complexity and heterogeneity of evidence. We purposely retained multiple types of evidence (eg, randomised controlled trials (RCT), consensus trials and qualitative video ethnography) to allow learning through quantitative, qualitative and mixed methods studies, as well as policy and grey literature, to increase relevance and examine the latest evidence base to date.

### Search strategy

A literature search was conducted within Medline, Embase, Cumulative Index to Nursing and Allied Health Literature and Cochrane Database of Systematic Reviews in January 2023. Search terms were developed after a preliminary search of articles covering our population, concept and context of interest, provided in table 1. This included the population of people with multimorbidity, the concept of problematic polypharmacy and the context of primary care. We limited our final search strategy to include only articles from 2004 onwards based on the earliest date of relevant articles from a preliminary search. Three additional databases were then searched for grey literature and clinical trial records: ClinicalTrials.gov, Science.gov and WorldCat in February 2023. We then followed an iterative process of snowballing through a supplementary search of references, citation lists and related articles using Google Scholar. Consistent with scoping reviews guidance, critical appraisal was not undertaken. An updated search was then completed in February 2024.

**Table 1:** Search terms used

Category	Search terms used
Population: *multimorbidity*	Multimorbid* or multiple long-term conditions or multiple health conditions
Concept: *problematic polypharmacy*	Polypharmacy or polypharmacotherapy or hyperpolypharmacy or polymedicine* or polimedicin* or multiple medic* or multimedic* or inappropriate prescrib* or overprescrib* or underprescrib* or deprescrib*
Context: *primary care*	Primary care or primary healthcare or general practi*

### Eligibility criteria

The eligibility criteria with typical exclusion examples are presented in table 2, guided by the Population, Concept and Context framework recommended by PRISMA-ScR^16^:

**Table 2:** Eligibility criteria and typical exclusion examples

Inclusion criteria	Typical exclusion examples
Population — adults living with multimorbidity: Studies must include adults (18 years and older) Studies must focus on those with multimorbidity—defined as 2 or more long-term conditions, not linked to an ‘index disease’	Studies focusing on patients with diabetes with renovascular disease (ie, has an index condition of diabetes)
Concept — problematic polypharmacy: Studies focusing on polypharmacy—defined as the concurrent use of multiple medications Studies that consider the long-term clinical impact of multiple medicines Studies that consider the consequences of multiple medicines or the ‘problematic’ element of polypharmacy	Studies focused on single medications Studies based on prescribing of antibiotics for acute presentations only Studies that are simply descriptive of the number of tablets taken and do not report any risk factors, outcomes or consequences
Context — primary care: Studies with relevance to primary care, including studies which crossed the primary-secondary care interface.	Studies solely on hospital-based pharmacists
Study type Studies written in English Studies presenting full descriptions of the research (eg, research studies, systematic reviews, randomised controlled trials, pilot studies and policy documents)	Letters, comments, conference abstracts, protocols, proceedings and so on.

### Study selection

Studies meeting the inclusion criteria were initially selected, based on screening the titles, abstracts and subsequent full papers by one researcher (JT). A random selection of 10% the records was analysed independently by a second researcher (TB) with 97% agreement of inclusion. Disagreements were resolved through discussion with the wider team.

### Data extraction and analysis

The data were extracted from eligible studies using a standardised data extraction form and included the author, year of publication, country of origin, type of the publication, polypharmacy definitions, type of participants, descriptions of interventions (if applicable) and key findings (see additional file 1). Further elaboration of the extracted data involved grouping studies according to their focus on either defining, identifying and addressing polypharmacy, with some spanning multiple elements. The main analysis took the form of a narrative synthesis, using mainly qualitative descriptive data consistent with PRISMA-ScR guidance.^16^ This compared themes and findings from grouped studies and using the collective evidence to draw contextualised insights and conclusions.

## Results

**Figure 1:**
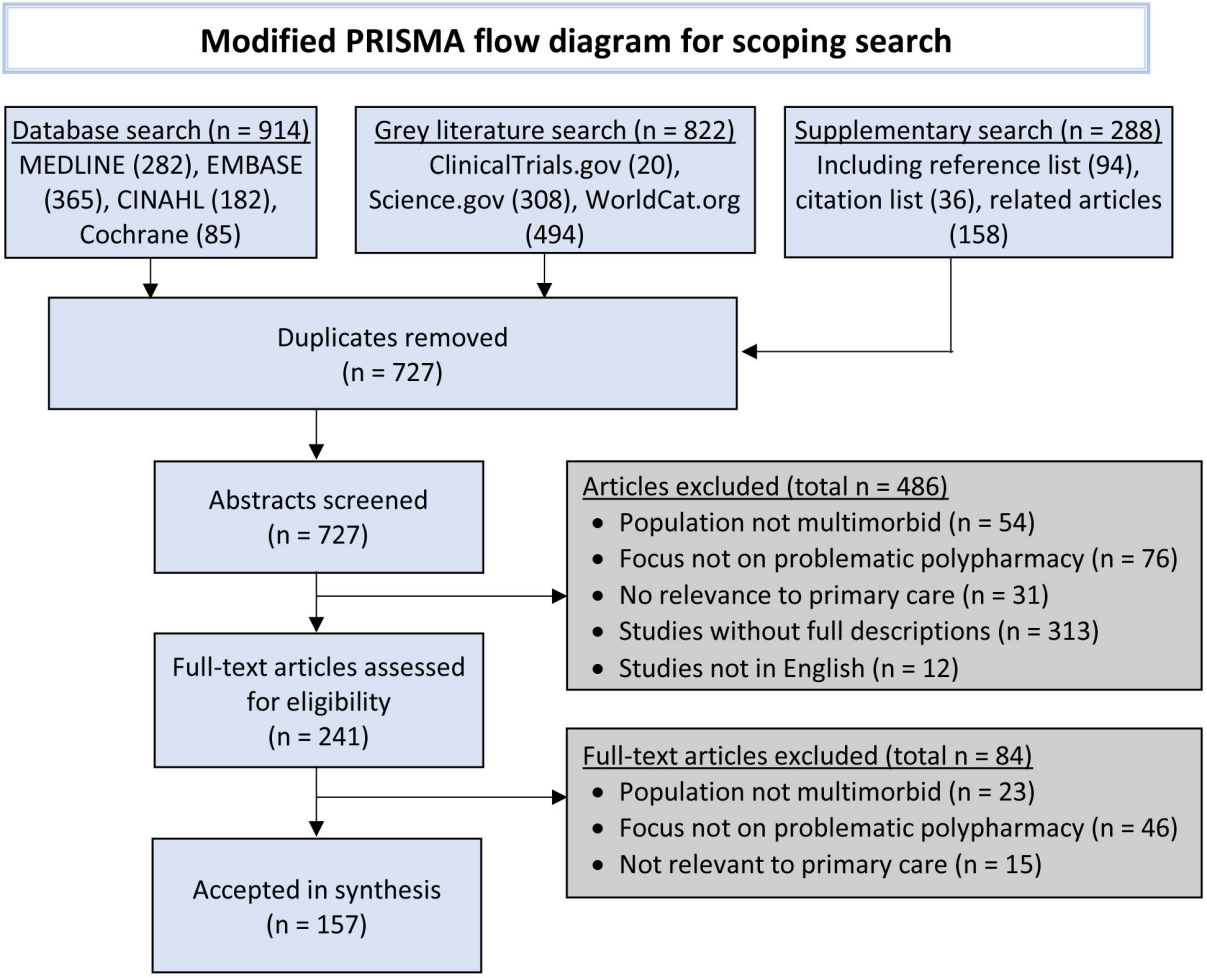
A Preferred Reporting Items for Systematic Reviews and Meta-Analyses flow diagram illustrating search results.

The search yielded 727 unique articles, with the process illustrated in figure 1. During eligibility screening, 486 were excluded after assessment of the abstract and 84 further full-text articles were excluded. A total of 157 articles were included in the final synthesis (online supplemental file 1), of which 19 were added during the updated search. This included 52 meta-analyses and reviews, 55 quantitative (including 9 RCTs and 19 longitudinal analyses), 36 qualitative studies (including 6 consensus studies and 2 RCT evaluations), 9 pilot or feasibility studies and 5 policy documents. The literature was varied with international articles covering a range of polypharmacy issues, from definitions to interventions, with some focussing on subpopulations with multimorbidity (eg, frailty) and subcategories within the broader context of primary care (eg, residential care facilities).

10.1136/bmjopen-2023-081698.supp1 Supplementary data

### Understanding how polypharmacy is defined

#### Numerous polypharmacy definitions

There is no consensus on a definition for polypharmacy, with significant variations in approaches to targeting problematic polypharmacy.^2 3 17^ Over 100 definitions of polypharmacy have been used, reflecting the discordance of approaches.^18 19^ Two main approaches to defining polypharmacy can be grouped into quantitative (using a form of medication count) and qualitative definitions (using descriptive notions of prescribing quality), with some studies using a combination of these definitions. Table 3 gives illustrative examples of these definitions.

**Table 3:** Illustrative list of examples for polypharmacy definitions

Definitions	Descriptions/examples
Quantitative definitions	
Single cut-offs of medication count	≥2, ≥3, ≥4, ≥5, ≥8, ≥10, ≥11 or ≥20 medications
Single cut-offs of a medication group	>2 anticholinergic medications >3 antipsychotic medications
Groups of medication counts	0–4 medications, 5–9 medications, 10–14 medications, ≥15 medications 0–5 medications, 6–8 medications, 9–11 medications, ≥12 medications 0–6 medications, 7–9 medications, 10–13 medications, ≥14 medications
Categorisation with levels or attributes	Mild polypharmacy 1–4 or 2–3 medications Minor polypharmacy 2–4 medications Major polypharmacy ≥5 medications Standard polypharmacy 5–9 or 6–9 medications Severe polypharmacy ≥6 or ≥10 medications Extreme polypharmacy ≥10 medications Hyperpolypharmacy ≥10 medications High-level polypharmacy ≥10 medications
Qualitative definitions	
Overprescribing	More medications than clinically indicated or unnecessary medications or presence of medications with no clinical indications or for which a safer alternative exists
Underprescribing	Lack of an indicated medication, or prescribed an inadequate amount or prescribed less frequent than appropriate
Drug-drug interactions	Any potential interaction, or harmful combination
Inappropriate medications	Defined by set criteria, for example, overprescribing, misprescribing and potential interactions
Prescribing cascade	Medication prescribed to treat the side effect of another medication
Absence of indication	Medication not matching the diagnosis
Therapeutic duplication	Same medicine used more than once or twice within the same therapeutic group used (eg, multiple antidepressants)
No therapeutic benefit	Medications with lack of effectiveness
Not cost-effective	Availability of an equally effective, lower cost alternative

Illustrative examples of wide range of definitions for polypharmacy used in the literature.^18 19^ Generally, quantitative definitions focus on operationalising medication count, regardless of whether polypharmacy is problematic whereas most qualitative definitions attach descriptors to describe scenarios where polypharmacy may be clinically problematic.

Quantitative definitions of polypharmacy were more frequent, with over 90% of publications using some form of medication count.^2 18–21^ For example, the WHO defines polypharmacy as four or more medicines, academic studies most commonly use 5 or more.^1 2^ Other quantitative definitions included categorisations rather than cut offs of medication count. These were frequently labelled levels (eg, mild, moderate and severe) or attributes (eg, excessive, extreme), yet counts within these categories were also inconsistent.^12 18 19 22–24^ Generally, quantitative definitions were easier to operationalise and more reproducible, with a focus on medication count, regardless of whether polypharmacy is problematic. In contrast, qualitative definitions largely required clinical judgement to evaluate prescribing quality, carrying a focus on when polypharmacy becomes problematic. This frequently highlighted the overuse or overprescribing of medications. But definitions also covered aspects of misprescribing, often through applying a list of defined prescribing criteria, and also underprescribing, though only a few studies emphasised this aspect. The terms ‘appropriate’, ‘inappropriate’ and ‘problematic’ polypharmacy were also commonly used to describe when multiple medications were justified compared with when the clinical indication was unclear.^3 18 19 25^ These definitions have now been expanded to cover further dimensions of polypharmacy, such as the increasing recognition of the importance of patient and carer input in determining the appropriateness of medications.^26 27^

#### The challenges of defining when polypharmacy is ‘problematic’

The understanding of polypharmacy has progressed over time, with an increasing shift to more clinically applicable definitions. This reflects the increasing complexity of decision-making for combinations of medicines tailored to individual needs. There is also recognition that it is not possible to account for clinical appropriateness through simple medication counts.^18 19^ Commonly people with multiple health needs may well be appropriately prescribed more than 10 medications for therapeutic and symptomatic benefit, which would be termed extreme polypharmacy in some studies and guidelines.^28 29^ Yet there is some validity to numeric approaches as increasing medications are strongly associated with drug-related problems, and very high counts of medication are usually questionable.^30^ There is also a need to improve the consistency of reporting medication exposure characteristics.^18 19 31–33^ Various definitions have been used to define temporality and ‘long-term’ use, with some publications including ‘acute’ and ‘as required’ medications as opposed to chronic medications, with varied definitions of time periods (ranging from 1 to 240 days).^18 19^ Terms such as problematic polypharmacy and inappropriate polypharmacy have been increasingly favoured, as they consider appropriateness and clinical decision-making.^34^ Yet qualitative research suggests that these labels were still insufficient to reflect the complexity of medicines management, with practitioners juggling terms such as ‘potentially inappropriate’ and ‘specifically appropriate’ and others considering them ‘judgemental’ and even ‘accusatory’.^35^

### Identifying problematic polypharmacy in practice

#### Targeting potentially high-risk populations

Various strategies target higher risk populations to try and identify problematic polypharmacy. One common approach uses simple cut offs of age (commonly ≥65 years) combined with cut offs of medications (frequently ≥5) and this was the main inclusion criterion for the majority of trials.^13^ Another approach adopted by multiple national recommendations advocate case finding through high-risk groups.^36–38^ For instance, both NICE guidelines and the Australian Commission on Safety and Quality in Healthcare recommend greater attention for older people with frailty, and complex multimorbidity and co-existing mental and physical health problems.^2 29 36^ Accordingly, several national indicators, initiatives and studies also use combinations of these approaches.^36–43^ Other approaches include risk scores to identify patients at high risk of particular outcomes (eg, hospitalisations or adverse drug reactions) but these require further development.^44 45^ Overall, strategies to identify potentially high-risk populations currently demonstrate variable validity in polypharmacy and are seldom comprehensive or holistic, as they are specific to the needs of particular groups.^2 36 46^

#### Targeting potentially inappropriate medicines

Evaluating the appropriateness of individual medications is a common approach both as a case-finding approach and as a surrogate measure of prescribing quality across polypharmacy. Various tools have been developed to identify potentially inappropriate medicines and these can be split into explicit and implicit tools, with some tools combining both (examples in table 4).^47–49^ The majority have been developed using expert opinion and consensus methodology, and originally were designed for evaluating individual medications, rather than polypharmacy as a whole.^47 50^ Explicit tools contain specific criteria or scenarios leading to potential adverse drug events and carry advantages of reproducibility and ease of automation.^51–55^ Implicit tools require judgement, which means they can be subjective and demand more time and clinical expertise. Nevertheless, explicit tools are limited to specific drugs and diseases, but implicit tools can be applied to any medication. This perhaps allows implicit tools greater applicability in polypharmacy, as explicit tools will miss out any medicines outside criteria.^56^

**Table 4:** Key examples of explicit and implicit tools of appropriate prescribing

Tool	Description	Strengths	Limitations
Beers criteria (*Explicit tool*)	First widely used explicit criteria Contains over 200 criteria (2023 version) including potentially inappropriate medications to be avoided such as drug disease and drug–drug interactions, particularly in older adults.	International studies have shown predictive validity for adverse drug reactions, falls, cognitive function, hospitalisation and death. Endorsed by the American Geriatric Society and updated approximately every 3–4 years. Easier to automate in drug records as criteria are specific	No positive clinical outcomes in RCTs to date No prioritisation of medications for review Can be challenging to use as long list of criteria Does not address underprescribing Focus is on individual medications rather than polypharmacy as a whole
Screening Tool of Older Person’s Prescriptions/ Screening Tool to Alert doctors to Right Treatment—STOPP/START (*Explicit tool, but newer versions also contain implicit measures*)	One of the most widely used explicit criteria globally for older adults Contains 133 criteria for potentially inappropriate medications, and 57 potential underprescribing criteria (version 3), organised according to medication and disease groups	Some positive outcomes shown in several RCTs Also addresses aspects of underprescribing in addition to overprescribing Easier to automate in computerised drug records as most criteria are specific	Misses out medications out of criteria Can be challenging to use as long list of criteria No prioritisation of medications for review Focus is on individual medications rather than polypharmacy as a whole
Medication Appropriateness Index—MAI (*Implicit tool*)	First widely used implicit criteria Lists 10 criteria that evaluate various aspects of medication appropriateness (eg, indication, effectiveness, dose)	Some positive outcomes shown in several RCTs Can be applied to all medicines	Time consuming to execute Requires clinical expertise and can be subjective Difficult to automate No prioritisation of medications for review Focus is seldom on polypharmacy as a whole or underprescribing
Drug Burden Index—DBI (*Implicit tool, as requires further judgement to evaluate appropriateness after calculating score*)	Widely researched risk score Calculates the cumulative exposure of sedatives and anticholinergics to give a score between 0 and 1.	International studies have shown predictive validity for falls, fractures, general practice visits and admission. Takes into account licenced doses to allow transferability between counties Easier to automate in drug records.	No positive clinical outcomes in RCTs to date No consideration for appropriateness or specific indication of medicines Only focused on sedatives, and anticholinergics Can be challenging to calculate at point of care unless computerised Does not address polypharmacy as a whole or underprescribing

A descriptive summary of selected examples of widely studied explicit and implicit tools.^48 54 174–177^

RCT, randomised controlled trial.

Several systematic reviews have revealed a high level of variability of included criteria within explicit tools.^47–50 54 57^ A review of 36 explicit tools reported criteria spanning 907 medications and medication classes, but only 44 medications and 4 classes were reported by the majority.^48^ This was despite over 85% of these tools being developed based on either the Beers or the Screening Tool of Older Person’s Prescriptions/Screening Tool to Alert doctors to Right Treatment (STOPP/START) criteria.^48^ Due to this, many studies combine several explicit criteria to complement the list of medications included.^47 48 50 58–62^ Only about a third of tools suggested alternative treatments to potentially inappropriate medicines, yet nearly 70% of suggested alternatives were deemed inappropriate by other tools.^47^ Implicit tools are also diverse in nature, with reviews identifying over 16 different tools incorporating implicit criteria.^54 63^ These ranged from risk scores to lists of questions specifying appropriate use or criteria to evaluate the administrative burden to patients.^54 63–65^ Several tools combine implicit and explicit indicators, including documents used for national guidance (eg, Australian Prescribing Indicators Tool).^63 66 67^

#### Key limitations in identifying problematic polypharmacy in practice

Current strategies to identify problematic polypharmacy demonstrate inadequate performance. At present, risk stratification tools remain too broad, and seldom consider the clinical appropriateness of individual medications.^34 68^ Though comprehensive explicit criteria are helpful in identifying potentially inappropriate medications, translation into everyday care remains elusive due to challenges in clinical application, and the omission of medications not included in criteria.^48 69^ For instance, previous studies have found that less than 25% of adverse drug reactions are caused by drugs listed by Beers criteria.^70 71^ Additionally, apart from STOPP/START, most widely used tools were not designed to also cover underprescribing (table 4), with some studies also choosing to omit many of the underprescribing criteria in its application.^47–50 54^ Furthermore, there have been questions as to the utility of long lists of medications as studies have shown a high prevalence of potentially inappropriate medications (over 30% of patients) but low variability within many criteria, potentially leaving little room for improvement.^72^ Studies also mention usability issues with such long lists, even with computerised integration, and the difficulties of making treatment decisions without prioritisation of criteria, particularly as their predictive validity is unknown.^47 59 68 73 74^ Still, as the majority of instruments were developed focussing on patients over 65 years old, the suitability for middle-aged adults is unknown, yet both polypharmacy and multimorbidity are increasing in this age group.^20 30 75^ Only a handful of criteria have been developed and validated (eg, Prescribing Optimally in Middle-aged People’s Treatments criteria), all including significantly fewer criteria for individual medications and medication classes.^54 56 63 76 77^ Again, this further limits applicability in problematic polypharmacy, where the whole of the medication regimen should be considered.

### Addressing problematic polypharmacy through interventions

#### Large variability in interventions addressing polypharmacy

Interventions to address problematic polypharmacy have covered a wide range of aims, such as reducing adverse drug reactions, increasing the appropriateness of medicines use, reducing falls, improving patient adherence and maintaining quality of life.^13 78–81^ To combat overprescribing specifically, deprescribing interventions have also received significant attention, though interventions that focus on underprescribing are much less.^82–86^ Several large reviews highlight good evidence of improving prescribing patterns, yet mixed and low certainty of evidence in improving patient-relevant outcome measures.^2 13–15 80 87–90^ Reviews covering over 150 primary studies reported no differences in all-cause mortality and no clear evidence of benefit in reduced hospitalisations, when comparing interventions to usual care.^13 80 88 91–94^ There were also no differences in quality of life, adverse drug reactions, readmission rates, primary care visits and emergency department visits.^13 80 92–94^ Two reviews have highlighted some economic benefits in reducing healthcare expenditure, but others highlight inconsistencies due to low-quality evidence.^92 93 95^ Overall, there is evidence that these interventions are safe and do not lead to harm, but may still be time and resource intensive for both patients and practitioners, as many require continuing input.^13 80 82^ Likewise, mixed evidence of improved clinical outcomes, such as falls, is also observed even in more focused populations, such as those with frailty and in long-term care facilities.^84–86 96^

#### Multiple intervention components to address polypharmacy, with unclear effectiveness

The majority of polypharmacy interventions were multimodal with a review revealing 14 different elements from 80 studies and an average of 2.5 elements per intervention.^13 97^ The most common elements included medication reviews, training for professionals and using tools, such as clinical decision support, checklists or audit and feedback.^13 43 74 97–100^ Other components strengthened interprofessional or multidisciplinary collaborations by involving clinical pharmacists, nurses or geriatricians.^13 92 94 97 100–107^ There were also patient-facing components, such as education and training for patients and patient interviews to seek their understanding and lived experiences with their medicines.^108–113^ Despite the growing literature on the importance of patient-centred care in medicines management, current literature highlights that patient priorities are seldom fully integrated into polypharmacy interventions.^13 82 91 97 114–121^ Patient-centred approaches also appear to be key to improving adherence, as a frequent discordance between practitioner and patient views is reported.^13 15 97 122–128^ More recent interventions that do adopt a patient-centred model show some mixed improvements in appropriate prescribing, but limited improvements in outcomes, reflecting some of the challenges of integrating patient priorities into routine medication reviews.^99 108–113 129 130^

In terms of effectiveness of individual intervention elements, similar effect sizes have been observed in reducing the number of potentially inappropriate medicines, with no particular components showing particular superiority.^13 80 97^ However, generalised professional education programmes appear to be less effective than individualised interventions.^13 131^ Medication reviews are also the most commonly adopted component, but as a single intervention, there remains insufficient evidence of medication reviews alone improve clinical outcomes.^84 132 133^ Despite the advantages of automation, electronic tools in trials demonstrate high variability in implementation within large pan-European and global trials, and no clear positive advantages on relevant patient outcomes have been reported.^13 134 135^ Pharmacists show promise as an extra resource for managing polypharmacy in individual studies, but two recent reviews revealed uncertain effects on optimising medicines.^92 94 102–106 136 137^ Community pharmacists can contribute to medication safety, but more in-depth management such as polypharmacy medication reviews was seen as outside the scope of community pharmacy.^105 138 139^

#### Key challenges in addressing problematic polypharmacy

In spite of the breadth of interventions targeting polypharmacy, it remains unclear which intervention components are more important.^13^ Theory-informed interventions are few and there are opportunities for improvements in intervention design through stronger foundations on theoretical frameworks and behaviour change techniques.^128 140–144^ Widespread variation exists in the everyday management of medicines and polypharmacy.^2 3 145–147^ These variations occur at patient, prescriber, regional and international levels, and indicate links between problematic polypharmacy and health inequalities.^1–3 39 145 146 148–150^ As such, multiple challenges to addressing problematic polypharmacy need to be overcome, going beyond the identification of individual barriers and facilitators and translating these into practice within the complexity of interlinked systems of care.^2 39 151 152^ The failure of the implementation of interventions is commonly down to the lack of consideration of integration into an already high-demand system in everyday primary care.^152–155^

For patients with polypharmacy and multimorbidity, prioritisation and decision-making are a challenge, given that they can receive 10 times the amount of information during consultations due to compounding health issues, interacting medications and complex social issues.^156^ Yet patient priorities and shared decision-making are vital to deciding the appropriateness of medications, so improvements need to be made to both the clarity of information provided and the integration of patient views into polypharmacy decisions.^2 26 27 114 118 121 128 130 156 157^ The majority of patients appear willing to discuss deprescribing medications, particularly if they have a good relationship with their doctor.^82 105 118 135 155^ However, they also have strong beliefs and attitudes of the value of their medicines, with inertia generated when feeling well on their current medication regimen.^82 118 120 152 158–160^

For health professionals, work and effort are required to even consider deprescribing, particularly as prescribing is so embedded in routine practice and finding an appropriate time to initiate the discussion is often difficult given competing priorities.^153 154 161–163^ A comprehensive polypharmacy medication review is described as ‘impossible’ to complete in 10 minutes, leading to practitioners defaulting to a swifter review and degrading medication reviews to being ‘mundane’ tasks.^158^ This is combined with the work to gain awareness (of new policies, guidelines and tools), overcome significant uncertainty in evidence (with ‘unmeasurable’ risk-benefit) and increase self-efficacy with limited resources and alternatives.^149 154 162–167^ On an organisational and systems level, fragmentation of care and poor coordination between healthcare teams and specialists often lead to deferring ownership of deprescribing, and miscommunication to patients, leading to medication-related problems.^149 151 161 166 168 169^ More comprehensive approaches and better resources are needed to support practitioners and organisations in pushing for improved polypharmacy decisions in a patient-centred manner, rather than simply maintaining the ‘status quo’.^35 82 148 162 164^

## Discussion

The evidence highlights significant challenges to optimising the targeting of patients with problematic polypharmacy for intervention. Despite the extensive number of studies, there is little evidence of improved patient outcomes even for higher risk populations, including individuals with frailty and those in long-term care facilities. This is highly suggestive that the targeting of patients with problematic polypharmacy needs to be more focused or even that the incorrect populations and medications are currently being targeted. Simple counts or ‘at-risk’ populations appear too broad as case-finding approaches. Though potentially inappropriate prescribing criteria can be helpful, this approach is also inadequate as it omits many patients not fitting criteria, lacks consistency across criteria and often overlooks underprescribing and multimorbidity. Furthermore, given the complexity of prescribing decisions in multimorbidity and the importance of considering patient values, potentially inappropriate criteria can rarely be used alone in assessing appropriateness. Due to the frequent confounding of multimorbidity observed in studies evaluating polypharmacy outcomes, coupled with the diverse combinations of medications involved in adverse drug reactions, there is a need for cross-cutting tools that can effectively capture the interplay of multiple health conditions in patients.^91 147^ Ultimately, the targeting of patients with problematic polypharmacy need to take into account patient perspectives, individual factors and clinical appropriateness.

### Implications for further research and practice

The approach to targeting patients needs to be improved as a first step, which may allow the identification of an optimal population for polypharmacy interventions. A next step to enhance clinical utility may be the routine adjustment of multimorbidity, as there is frequent confounding of polypharmacy outcomes within studies.^91^ In doing so, we may be able to identify patients who are both overprescribed and underprescribed medicines yet consider some degree of clinical appropriateness. An opportunity exists to produce a cross-cutting measure beyond single diseases and individual drug interactions to evaluate patients as a whole, with the aim of improving overall health.^68 164^

The multifactorial drivers of polypharmacy also mean that approaches to address problematic polypharmacy need to go beyond targeting patients and practitioners alone.^39 152^ Despite this, evidence of a systems approach encompassing policy-makers, organisations, practitioners, patients and carers is lacking.^2 39 151 152^ Both the growth of evidence-based medicine and desire to minimise all risk are significant drivers of increased medicines burden and problematic polypharmacy. Yet polypharmacy is rarely ‘evidence-based’, as it would be impossible to have a large enough sample size to perform drug trials and meta-analyses of the millions of combinations that patients with multimorbidity are taking.^6 170^ Studies examining exclusion criteria of RCTs estimate that over 90% of this population would be excluded from trials, questioning their representativeness.^171^ The emphasis on following guidelines and increasing treatment intensity should be balanced with the understanding that high-quality personalised healthcare can only be achieved through also carefully reducing, stopping or not initiating medication, with shared decision-making and agreed care objectives.^172 173^

### Strengths and limitations

This scoping review syntheses a wide breath of literature to explore the existing evidence. It allowed a systematic approach on an initial search strategy and was also adaptable to heterogeneous sources (eg, policy documents) and developing literature (eg, pilot studies) through related article, supplementary and grey literature searching. It examined the overlapping concepts of polypharmacy and multimorbidity concurrently, allowing synergies in evidence generation and critique.

There are several limitations of our review to consider. As with other scoping reviews, critical appraisal was not performed. Polypharmacy is an area that has received widespread attention, with hundreds of primary studies and dozens of systematic reviews. Hence, in our attempts to present generalisable findings, the nuances within primary studies may be lost, such as differences in study setting, population or intervention characteristics. While we made efforts to specifically extract primary care-related findings, it was not always possible to separate results in studies encompassing both primary and secondary care. Furthermore, by emphasising multimorbidity and primary care in our search, we may have overlooked research investigating more acute medication adjustments in polypharmacy patients.

## Conclusion

An optimal approach for targeting patients with problematic polypharmacy is yet to be determined. To address the challenges posed by confounding, it may be valuable to develop a cross-cutting measure of polypharmacy that consistently accounts for multimorbidity. The complexities of prescribing decisions in polypharmacy highlight the importance of improved approaches that consider patient perspectives, individual factors and clinical appropriateness.

## Supplementary Material

Reviewer comments

Author's manuscript

## Data Availability

All data relevant to the study are included in the article or uploaded as supplementary information.

## References

[1] World Health Organization . Medication without harm: world health organization. 2017.

[2] The Department of Health and Social Care . National Overprescribing review report: good for you, good for us, good for everybody. 2021.

[3] Duerden M , Avery T , Payne R . Polypharmacy and Medicines Optimisation. London: The King’s Fund, 2013.

[4] Hajjar ER , Cafiero AC , Hanlon JT . Polypharmacy in elderly patients. Am J Geriatr Pharmacother 2007;5:345–51. 10.1016/j.amjopharm.2007.12.002 18179993

[5] National Institute for Health and Care Excellence (NICE) . Acute coronary syndromes [Ng185]. 2020. 33301270

[6] Buffel du Vaure C , Dechartres A , Battin C , et al . Exclusion of patients with concomitant chronic conditions in ongoing randomised controlled trials targeting 10 common chronic conditions and registered at Clinicaltrials.Gov: a systematic review of registration details. BMJ Open 2016;6:e012265. 10.1136/bmjopen-2016-012265 PMC5051474 27678540

[7] Osanlou R , Walker L , Hughes DA , et al . Adverse drug reactions, Multimorbidity and Polypharmacy: a prospective analysis of 1 month of medical admissions. BMJ Open 2022;12:e055551. 10.1136/bmjopen-2021-055551 PMC9255409 35788071

[8] Gellad WF , Grenard JL , Marcum ZA . A systematic review of barriers to medication adherence in the elderly: looking beyond cost and regimen complexity. Am J Geriatr Pharmacother 2011;9:11–23. 10.1016/j.amjopharm.2011.02.004 21459305 PMC3084587

[9] Nordin Olsson I , Runnamo R , Engfeldt P . Medication quality and quality of life in the elderly, a cohort study. Health Qual Life Outcomes 2011;9:95. 10.1186/1477-7525-9-95 22054205 PMC3216839

[10] Davies EA , O’Mahony MS . Adverse drug reactions in special populations - the elderly: ADRs in the elderly. Br J Clin Pharmacol 2015;80:796–807. 10.1111/bcp.12596 25619317 PMC4594722

[11] Corsonello A , Pedone C , Incalzi RA . Age-related pharmacokinetic and pharmacodynamic changes and related risk of adverse drug reactions. Curr Med Chem 2010;17:571–84. 10.2174/092986710790416326 20015034

[12] Doherty AS , Boland F , Moriarty F , et al . Adverse drug reactions and associated patient characteristics in older community-dwelling adults: a 6-year prospective cohort study. Br J Gen Pract 2023;73:e211–9. 10.3399/BJGP.2022.0181 36823047 PMC9923764

[13] Cole JA , Gonçalves-Bradley DC , Alqahtani M , et al . Interventions to improve the appropriate use of Polypharmacy for older people. Cochrane Database Syst Rev 2023;10:CD008165. 10.1002/14651858.CD008165.pub5 37818791 PMC10565901

[14] Alldred DP , Kennedy M-C , Hughes C , et al . Interventions to Optimise prescribing for older people in care homes. Cochrane Database Syst Rev 2016;2:CD009095. 10.1002/14651858.CD009095.pub3 26866421 PMC7111425

[15] Cross AJ , Elliott RA , Petrie K , et al . Interventions for improving Medication‐Taking ability and adherence in older adults prescribed multiple medications. Cochrane Database Syst Rev 2020;5:CD012419. 10.1002/14651858.CD012419.pub2 32383493 PMC7207012

[16] Tricco AC , Lillie E , Zarin W , et al . PRISMA extension for Scoping reviews (PRISMA-SCR): checklist and explanation. Ann Intern Med 2018;169:467–73. 10.7326/M18-0850 30178033

[17] Taghy N , Cambon L , Cohen J-M , et al . Failure to reach a consensus in Polypharmacy definition: an obstacle to measuring risks and impacts—results of a literature review. Ther Clin Risk Manag 2020;16:57–73. 10.2147/TCRM.S214187 32103967 PMC7023902

[18] Masnoon N , Shakib S , Kalisch-Ellett L , et al . What is Polypharmacy? A systematic review of definitions. BMC Geriatr 2017;17:230. 10.1186/s12877-017-0621-2 29017448 PMC5635569

[19] Sirois C , Domingues NS , Laroche M-L , et al . Polypharmacy definitions for Multimorbid older adults need stronger foundations to guide research. Pharmacy 2019;7:126. 10.3390/pharmacy7030126 31470621 PMC6789889

[20] Vos R , Boesten J , van den Akker M . Fifteen-year Trajectories of Multimorbidity and Polypharmacy in Dutch primary care—A longitudinal analysis of age and sex patterns. PLOS ONE 2022;17:e0264343. 10.1371/journal.pone.0264343 35213615 PMC8880753

[21] Aubert CE , Streit S , Da Costa BR , et al . Polypharmacy and specific Comorbidities in university primary care settings. Eur J Intern Med 2016;35:35–42. 10.1016/j.ejim.2016.05.022 27289492

[22] Gutiérrez-Valencia M , Aldaz Herce P , Lacalle-Fabo E , et al . Prevalence of Polypharmacy and associated factors in older adults in Spain: data from the national health survey 2017. Med Clin (Barc) 2019;153:141–50. 10.1016/j.medcli.2018.12.013 30803798

[23] Rieckert A , Trampisch US , Klaaßen-Mielke R , et al . Polypharmacy in older patients with chronic diseases: a cross-sectional analysis of factors associated with excessive Polypharmacy. BMC Fam Pract 2018;19:113. 10.1186/s12875-018-0795-5 30021528 PMC6052592

[24] Payne RA , Avery AJ , Duerden M , et al . Prevalence of Polypharmacy in a Scottish primary care population. Eur J Clin Pharmacol 2014;70:575–81. 10.1007/s00228-013-1639-9 24487416

[25] Monégat M , Sermet C , Perronnin M , et al . Polypharmacy: definitions, measurement and stakes involved. Review of the Literature and Measurement Tests 2014;8.

[26] Mair A , Wilson M , Dreischulte T . Addressing the challenge of Polypharmacy. Annu Rev Pharmacol Toxicol 2020;60:661–81. 10.1146/annurev-pharmtox-010919-023508 31589822

[27] Heaton J , Britten N , Krska J , et al . Person-centred medicines Optimisation policy in England: an agenda for research on Polypharmacy. Prim Health Care Res Dev 2017;18:24–34. 10.1017/S1463423616000207 27306579

[28] Sirois C , Lunghi C , Laroche M-L , et al . The delicate choice of optimal basic therapy for Multimorbid older adults: A cross-sectional survey. Res Social Adm Pharm 2019;15:761–6. 10.1016/j.sapharm.2018.09.008 30249377

[29] National Institute for Health and Care Excellence (NICE) . Multimorbidity and Polypharmacy [Ktt18]. 2017.

[30] Guthrie B , Makubate B , Hernandez-Santiago V , et al . The rising tide of Polypharmacy and drug-drug interactions: population database analysis 1995–2010. BMC Med 2015;13:74. 10.1186/s12916-015-0322-7 25889849 PMC4417329

[31] McCarthy C , Flood M , Clyne B , et al . Medication changes and potentially inappropriate prescribing in older patients with significant Polypharmacy. Int J Clin Pharm 2023;45:191–200. 10.1007/s11096-022-01497-2 36385206

[32] von Buedingen F , Hammer MS , Meid AD , et al . Changes in prescribed medicines in older patients with Multimorbidity and Polypharmacy in general practice. BMC Fam Pract 2018;19:131. 10.1186/s12875-018-0825-3 30055583 PMC6064613

[33] Calderón-Larrañaga A , Gimeno-Feliu LA , González-Rubio F , et al . Polypharmacy patterns: Unravelling systematic associations between prescribed medications. PLoS ONE 2013;8:e84967. 10.1371/journal.pone.0084967 24376858 PMC3869920

[34] Kadam UT , Roberts I , White S , et al . Conceptualizing multiple drug use in patients with Comorbidity and Multimorbidity: proposal for standard definitions beyond the term Polypharmacy. J Clin Epidemiol 2019;106:98–107. 10.1016/j.jclinepi.2018.10.014 30385327

[35] Clyne B , Cooper JA , Hughes CM , et al . Potentially inappropriate or specifically appropriate?’ qualitative evaluation of general practitioners views on prescribing, Polypharmacy and potentially inappropriate prescribing in older people. BMC Fam Pract 2016;17:109. 10.1186/s12875-016-0507-y 27515854 PMC4982127

[36] Australian Commission on Safety and Quality in Health Care . Polypharmacy, 75 years and over. 2020.

[37] Scottish Government Polypharmacy Model of Care Group . Polypharmacy Guidance, Realistic Prescribing. 3rd edn. Scottish Government: Edinburgh, 2018.

[38] All Wales Medicines Strategy Group . Polypharmacy in older people: A guide for Healthcare professionals. 2023.

[39] McIntosh J , Alonso A , MacLure K , et al . A case study of Polypharmacy management in nine European countries: implications for change management and implementation. PLOS ONE 2018;13. 10.1371/journal.pone.0195232 PMC5905890 29668763

[40] Krüger C , Schäfer I , van den Bussche H , et al . Non-random relations in drug use expressed as patterns comprising prescription and over-the-counter drugs in Multimorbid elderly patients in primary care: data of the exploratory analysis of the Multicentre, observational cohort study Multicare. Eur J Gen Pract 2021;27:119–29. 10.1080/13814788.2021.1933425 34132623 PMC8211130

[41] Villén N , Guisado-Clavero M , Fernández-Bertolín S , et al . Multimorbidity patterns, Polypharmacy and their association with liver and kidney abnormalities in people over 65 years of age: a longitudinal study. BMC Geriatr 2020;20:206. 10.1186/s12877-020-01580-1 32532213 PMC7291454

[42] Monterde D , Vela E , Clèries M , et al . Multimorbidity as a Predictor of health service utilization in primary care: a Registry-based study of the Catalan population. BMC Fam Pract 2020;21:39. 10.1186/s12875-020-01104-1 32066377 PMC7026948

[43] Jäger C , Freund T , Steinhäuser J , et al . Impact of a tailored program on the implementation of evidence-based recommendations for Multimorbid patients with Polypharmacy in primary care practices—results of a cluster-randomized controlled trial. Implementation Sci 2017;12:8. 10.1186/s13012-016-0535-y PMC5237147 28086976

[44] Novella A , Elli C , Tettamanti M , et al . Relation between drug therapy-based Comorbidity indices, Charlson’s Comorbidity index, Polypharmacy and mortality in three samples of older adults. Arch Gerontol Geriatr 2022;100:S0167-4943(22)00030-9. 10.1016/j.archger.2022.104649 35149290

[45] Häppölä P , Havulinna AS , Tasa T , et al . A data-driven medication score predicts 10-year mortality among aging adults. Sci Rep 2020;10:15760. 10.1038/s41598-020-72045-z 32978407 PMC7519677

[46] Guisado-Clavero M , Violán C , López-Jimenez T , et al . Medication patterns in older adults with Multimorbidity: a cluster analysis of primary care patients. BMC Fam Pract 2019;20:82. 10.1186/s12875-019-0969-9 31195985 PMC6567459

[47] Schiavo G , Forgerini M , Lucchetta RC , et al . A comprehensive look at explicit screening tools for potentially inappropriate medication: A systematic Scoping review. Australas J Ageing 2022;41:357–82. 10.1111/ajag.13046 35226786

[48] Motter FR , Fritzen JS , Hilmer SN , et al . Potentially inappropriate medication in the elderly: a systematic review of validated explicit criteria. Eur J Clin Pharmacol 2018;74:679–700. 10.1007/s00228-018-2446-0 29589066

[49] Lucchetti G , Lucchetti ALG . Inappropriate prescribing in older persons: A systematic review of medications available in different criteria. Arch Gerontol Geriatr 2017;68:55–61. 10.1016/j.archger.2016.09.003 27649514

[50] Lee G , Lim J-F , Page AT , et al . Applicability of explicit potentially inappropriate medication lists to the Australian context: A systematic review. Australas J Ageing 2022;41:200–21. 10.1111/ajag.13038 35025135

[51] Vrdoljak D , Borovac JA . Medication in the elderly - considerations and therapy prescription guidelines. Acta Med Acad 2015;44:159–68. 10.5644/ama2006-124.142 26702910

[52] Lopez-Rodriguez JA , Rogero-Blanco E , Aza-Pascual-Salcedo M , et al . Potentially inappropriate prescriptions according to explicit and implicit criteria in patients with Multimorbidity and Polypharmacy. MULTIPAP: A cross-sectional study. PLOS ONE 2020;15:e0237186. 10.1371/journal.pone.0237186 32785232 PMC7423095

[53] Sánchez-Fidalgo S , Guzmán-Ramos MI , Galván-Banqueri M , et al . Prevalence of drug interactions in elderly patients with Multimorbidity in primary care. Int J Clin Pharm 2017;39:343–53. 10.1007/s11096-017-0439-1 28238102

[54] Masnoon N , Shakib S , Kalisch-Ellett L , et al . Tools for assessment of the appropriateness of prescribing and association with patient-related outcomes: A systematic review. Drugs Aging 2018;35:43–60. 10.1007/s40266-018-0516-8 29350335

[55] Tampaki M , Livada A , Fourka M-N , et al . Inappropriate prescribing in geriatric rural primary care: impact on adverse outcomes and relevant risk factors in a prospective observational cohort study. Aging Clin Exp Res 2023;35:1901–7. 10.1007/s40520-023-02475-y 37428424 PMC10460359

[56] Cooper JA , Ryan C , Smith SM , et al . The development of the PROMPT (prescribing Optimally in middle-aged people’s treatments) criteria. BMC Health Serv Res 2014;14:484. 10.1186/s12913-014-0484-6 25410615 PMC4229620

[57] Lee CS , Tan NC , Goh KLS , et al . Factors associated with potentially inappropriate prescribing among older persons in primary care settings: systematic review. Proceedings of Singapore Healthcare 2023;32. 10.1177/20101058231181478

[58] Troncoso-Mariño A , López-Jiménez T , Roso-Llorach A , et al . Medication‐Related problems in older people in Catalonia: A Real‐World data study. Pharmacoepidemiol Drug Saf 2021;30:220–8. 10.1002/pds.5149 33026123 PMC7839740

[59] Rogero-Blanco E , Del-Cura-González I , Aza-Pascual-Salcedo M , et al . Drug interactions detected by a computer-assisted prescription system in primary care patients in Spain: MULTIPAP study. Eur J Gen Pract 2021;27:90–6. 10.1080/13814788.2021.1917543 33982632 PMC8128212

[60] Rogero-Blanco E , López-Rodríguez JA , Sanz-Cuesta T , et al . Use of an electronic clinical decision support system in primary care to assess inappropriate Polypharmacy in young seniors with Multimorbidity: observational, descriptive, cross-sectional study. JMIR Med Inform 2020;8:e14130. 10.2196/14130 PMC7078622 32126005

[61] D’Aiuto C , Lunghi C , Guénette L , et al . Health care system costs related to potentially inappropriate medication use involving opioids in older adults in Canada. BMC Health Serv Res 2023;23:1295. 10.1186/s12913-023-10303-2 38001466 PMC10668473

[62] Manirajan P , Sivanandy P . Drug utilisation review among geriatric patients with Noncommunicable diseases in a primary care setting in Malaysia. Healthcare (Basel) 2023;11:1665. 10.3390/healthcare11121665 37372782 PMC10298635

[63] Kaufmann CP , Tremp R , Hersberger KE , et al . Inappropriate prescribing: a systematic overview of published assessment tools. Eur J Clin Pharmacol 2014;70:1–11. 10.1007/s00228-013-1575-8 24019054

[64] Burt J , Elmore N , Campbell SM , et al . Developing a measure of Polypharmacy appropriateness in primary care: systematic review and expert consensus study. BMC Med 2018;16:91. 10.1186/s12916-018-1078-7 29895310 PMC5998565

[65] George J , Phun Y-T , Bailey MJ , et al . Development and validation of the medication regimen complexity index. Ann Pharmacother 2004;38:1369–76. 10.1345/aph.1D479 15266038

[66] Basger BJ , Chen TF , Moles RJ . Validation of prescribing appropriateness criteria for older Australians using the RAND/UCLA appropriateness method. BMJ Open 2012;2:e001431. 10.1136/bmjopen-2012-001431 PMC3467596 22983875

[67] Scott I , Anderson K , Freeman C . Review of structured guides for Deprescribing. Eur J Hosp Pharm 2017;24:51–7. 10.1136/ejhpharm-2015-000864 31156899 PMC6451538

[68] Cadogan CA , Ryan C , Hughes CM . Appropriate Polypharmacy and medicine safety: when many is not too many. Drug Saf 2016;39:109–16. 10.1007/s40264-015-0378-5 26692396 PMC4735229

[69] Akyon SH , Akyon FC , Yılmaz TE . Artificial intelligence-supported web application design and development for reducing Polypharmacy side effects and supporting rational drug use in geriatric patients. Front Med (Lausanne) 2023;10:1029198. 10.3389/fmed.2023.1029198 36968816 PMC10030839

[70] Laroche M-L , Charmes J-P , Nouaille Y , et al . Is inappropriate medication use a major cause of adverse drug reactions in the elderly Br J Clin Pharmacol 2007;63:177–86. 10.1111/j.1365-2125.2006.02831.x 17166186 PMC2000580

[71] Miller GC , Valenti L , Britt H , et al . Drugs causing adverse events in patients aged 45 or older: a randomised survey of Australian general practice patients. BMJ Open 2013;3:e003701. 10.1136/bmjopen-2013-003701 PMC3796276 24114371

[72] Brown JD , Hutchison LC , Li C , et al . Predictive validity of the beers and screening tool of older persons’ potentially inappropriate prescriptions (STOPP) criteria to detect adverse drug events, hospitalizations, and emergency Department visits in the United States. J Am Geriatr Soc 2016;64:22–30. 10.1111/jgs.13884 26782849 PMC5287350

[73] Cahir C , Moriarty F , Teljeur C , et al . Potentially inappropriate prescribing and vulnerability and hospitalization in older community-dwelling patients. Ann Pharmacother 2014;48:1546–54. 10.1177/1060028014552821 25248541

[74] Del Cura-González I , López-Rodríguez JA , Leiva-Fernández F , et al . How to improve Healthcare for patients with Multimorbidity and Polypharmacy in primary care: A pragmatic cluster-randomized clinical trial of the MULTIPAP intervention. J Pers Med 2022;12:752. 10.3390/jpm12050752 35629175 PMC9144280

[75] O’Regan A , Glynn L , Niranjamn V , et al . How often do patients attend general practice, how often are they referred to hospital, and how do multi-morbidity and Polypharmacy affect general practice attendance and referral rates Rural Remote Health 2023;23:8106. 10.22605/RRH8106 36802742

[76] Khatter A , Moriarty F , Ashworth M , et al . Prevalence and predictors of potentially inappropriate prescribing in middle-aged adults: a repeated cross-sectional study. Br J Gen Pract 2021;71:e491–7. 10.3399/BJGP.2020.1048 33606659 PMC8136579

[77] Cooper JA , Moriarty F , Ryan C , et al . Potentially inappropriate prescribing in two populations with differing socio-economic profiles: a cross-sectional database study using the PROMPT criteria. Eur J Clin Pharmacol 2016;72:583–91. 10.1007/s00228-015-2003-z 26820292 PMC4834102

[78] Rankin A , Cadogan CA , Patterson SM , et al . Interventions to improve the appropriate use of Polypharmacy for older people. Cochrane Database Syst Rev 2018;9:CD008165. 10.1002/14651858.CD008165.pub4 30175841 PMC6513645

[79] Cooper JA , Cadogan CA , Patterson SM , et al . Interventions to improve the appropriate use of Polypharmacy in older people: a Cochrane systematic review. BMJ Open 2015;5:e009235. 10.1136/bmjopen-2015-009235 PMC4679890 26656020

[80] Keller MS , Qureshi N , Mays AM , et al . Cumulative update of a systematic overview evaluating interventions addressing Polypharmacy. JAMA Netw Open 2024;7:e2350963. 10.1001/jamanetworkopen.2023.50963 38198136 PMC10782233

[81] Johansson T , Abuzahra ME , Keller S , et al . Impact of strategies to reduce Polypharmacy on clinically relevant endpoints: a systematic review and meta-analysis: impact of strategies to reduce Polypharmacy. Br J Clin Pharmacol 2016;82:532–48. 10.1111/bcp.12959 27059768 PMC4972170

[82] Reeve J , Maden M , Hill R , et al . Deprescribing medicines in older people living with Multimorbidity and Polypharmacy: the TAILOR evidence synthesis. Health Technol Assess 2022;26:1–148. 10.3310/AAFO2475 PMC9376985 35894932

[83] Reeve E , Gnjidic D , Long J , et al . A systematic review of the emerging definition of ‘Deprescribing’ with network analysis: implications for future research and clinical practice.: the emerging definition of ‘Deprescribing. Br J Clin Pharmacol 2015;80:1254–68. 10.1111/bcp.12732 27006985 PMC4693477

[84] Seppala LJ , Kamkar N , van Poelgeest EP , et al . Medication reviews and Deprescribing as a single intervention in falls prevention: a systematic review and meta-analysis. Age Ageing 2022;51:afac191. 10.1093/ageing/afac191 36153749 PMC9509688

[85] Kua C-H , Mak VSL , Huey Lee SW . Health outcomes of Deprescribing interventions among older residents in nursing homes: A systematic review and meta-analysis. J Am Med Dir Assoc 2019;20:362–72. 10.1016/j.jamda.2018.10.026 30581126

[86] Shrestha S , Poudel A , Cardona M , et al . Impact of Deprescribing dual-purpose medications on patient-related outcomes for older adults near end-of-life: a systematic review and meta-analysis. Ther Adv Drug Saf 2021;12:20420986211052343. 10.1177/20420986211052343 34707802 PMC8543710

[87] Soler O , Barreto JOM . Community-level pharmaceutical interventions to reduce the risks of Polypharmacy in the elderly: overview of systematic reviews and economic evaluations. Front Pharmacol 2019;10:302. 10.3389/fphar.2019.00302 31001117 PMC6454558

[88] Anderson LJ , Schnipper JL , Nuckols TK , et al . A systematic overview of systematic reviews evaluating interventions addressing Polypharmacy. Am J Health Syst Pharm 2019;76:1777–87. 10.1093/ajhp/zxz196 31612924 PMC7170727

[89] Dills H , Shah K , Messinger-Rapport B , et al . Deprescribing medications for chronic diseases management in primary care settings: A systematic review of randomized controlled trials. J Am Med Dir Assoc 2018;19:923–35. 10.1016/j.jamda.2018.06.021 30108032

[90] Ulley J , Harrop D , Ali A , et al . Deprescribing interventions and their impact on medication adherence in community-dwelling older adults with Polypharmacy: a systematic review. BMC Geriatr 2019;19:15. 10.1186/s12877-019-1031-4 30658576 PMC6339421

[91] Ali MU , Sherifali D , Fitzpatrick-Lewis D , et al . Interventions to address Polypharmacy in older adults living with Multimorbidity: review of reviews. Can Fam Physician 2022;68:e215–26. 10.46747/cfp.6807e215 35831093 PMC9842141

[92] Riordan DO , Walsh KA , Galvin R , et al . The effect of pharmacist-led interventions in Optimising prescribing in older adults in primary care: A systematic review. SAGE Open Med 2016;4:2050312116652568. 10.1177/2050312116652568 27354917 PMC4910534

[93] Hill-Taylor B , Walsh KA , Stewart S , et al . Effectiveness of the STOPP/START (screening tool of older persons' potentially inappropriate prescriptions/screening tool to alert doctors to the right treatment) criteria: systematic review and meta-analysis of randomized controlled studies. J Clin Pharm Ther 2016;41:158–69. 10.1111/jcpt.12372 26990017

[94] Tasai S , Kumpat N , Dilokthornsakul P , et al . Impact of medication reviews delivered by community pharmacist to elderly patients on Polypharmacy: A meta-analysis of randomized controlled trials. J Patient Saf 2021;17:290–8. 10.1097/PTS.0000000000000599 30920431

[95] Laberge M , Sirois C , Lunghi C , et al . Economic evaluations of interventions to optimize medication use in older adults with Polypharmacy and Multimorbidity: A systematic review. Clin Interv Aging 2021;16:767–79. 10.2147/CIA.S304074 33981140 PMC8108125

[96] Ibrahim K , Cox NJ , Stevenson JM , et al . A systematic review of the evidence for Deprescribing interventions among older people living with frailty. BMC Geriatr 2021;21:258. 10.1186/s12877-021-02208-8 33865310 PMC8052791

[97] Lee JQ , Ying K , Lun P , et al . Intervention elements to reduce inappropriate prescribing for older adults with Multimorbidity receiving outpatient care: a Scoping review. BMJ Open 2020;10:e039543. 10.1136/bmjopen-2020-039543 PMC7440708 32819958

[98] Michiels-Corsten M , Gerlach N , Schleef T , et al . Generic instruments for drug discontinuation in primary care: A systematic review. Br J Clin Pharmacol 2020;86:1251–66. 10.1111/bcp.14287 32216066 PMC7319012

[99] Zechmann S , Senn O , Valeri F , et al . Effect of a patient-centred Deprescribing procedure in older Multimorbid patients in Swiss primary care - A cluster-randomised clinical trial. BMC Geriatr 2020;20:471. 10.1186/s12877-020-01870-8 33198634 PMC7670707

[100] Jäger C , Steinhäuser J , Freund T , et al . A tailored programme to implement recommendations for Multimorbid patients with Polypharmacy in primary care practices—process evaluation of a cluster randomized trial. Implement Sci 2017;12:31. 10.1186/s13012-017-0559-y 28264693 PMC5339959

[101] San-José A , Pérez-Bocanegra C , Agustí A , et al . Integrated health intervention on Polypharmacy and inappropriate prescribing in elderly people with Multimorbidity: results at the end of the intervention and at 6 months after the intervention. Med Clin (Barc) 2021;156:263–9. 10.1016/j.medcli.2020.04.030 32593414

[102] Cardwell K , Smith SM , Clyne B , et al . Evaluation of the general practice pharmacist (GPP) intervention to Optimise prescribing in Irish primary care: a non-randomised pilot study. BMJ Open 2020;10:e035087. 10.1136/bmjopen-2019-035087 PMC7322285 32595137

[103] Alaa Eddine N , Schreiber J , El-Yazbi AF , et al . A pharmacist-led medication review service with a Deprescribing focus guided by implementation science. Front Pharmacol 2023;14:1097238. 10.3389/fphar.2023.1097238 36794277 PMC9922726

[104] Benson M , Murphy D , Hall L , et al . Medication management for complex patients in primary care: application of a remote, Asynchronous clinical pharmacist model. Postgrad Med 2021;133:784–90. 10.1080/00325481.2021.1934492 34047254

[105] Uhl MC , Muth C , Gerlach FM , et al . Patient-perceived barriers and Facilitators to the implementation of a medication review in primary care: a qualitative thematic analysis. BMC Fam Pract 2018;19:3. 10.1186/s12875-017-0707-0 29304725 PMC5755323

[106] Bell HT , Granas AG , Enmarker I , et al . Nurses’ and pharmacists’ learning experiences from participating in Interprofessional medication reviews for elderly in primary health care - a qualitative study. BMC Fam Pract 2017;18:30. 10.1186/s12875-017-0598-0 28241789 PMC5330158

[107] Köberlein-Neu J , Mennemann H , Hamacher S , et al . Interprofessional medication management in patients with multiple morbidities. Deutsches Ärzteblatt International 2016. 10.3238/arztebl.2016.0741 PMC5159681 27890050

[108] Molist-Brunet N , Sevilla-Sánchez D , Puigoriol-Juvanteny E , et al . Improving individualized prescription in patients with Multimorbidity through medication review. BMC Geriatr 2022;22:417. 10.1186/s12877-022-03107-2 35549672 PMC9096338

[109] McCarthy C , Clyne B , Boland F , et al . GP-delivered medication review of Polypharmacy, Deprescribing, and patient priorities in older people with Multimorbidity in Irish primary care (Sppire study): A cluster randomised controlled trial. PLOS Med 2022;19:e1003862. 10.1371/journal.pmed.1003862 34986166 PMC8730438

[110] McCarthy C , Pericin I , Smith SM , et al . Patient and general practitioner experiences of implementing a medication review intervention in older people with Multimorbidity: process evaluation of the Sppire trial. Health Expectations 2022;25:3225–37. 10.1111/hex.13630 36245339 PMC9700182

[111] McCarthy C , Moriarty F , Wallace E , et al . The evolution of an evidence based intervention designed to improve prescribing and reduce Polypharmacy in older people with Multimorbidity and significant Polypharmacy in primary care (Sppire). J Comorb 2020;10:2235042X20946243. 10.1177/2235042X20946243 PMC7493276 32974211

[112] Schäfer I , Kaduszkiewicz H , Mellert C , et al . Narrative medicine-based intervention in primary care to reduce Polypharmacy: results from the cluster-randomised controlled trial Multicare AGENDA. BMJ Open 2018;8:e017653. 10.1136/bmjopen-2017-017653 PMC5786138 29362248

[113] Mann N-K , Schmiedl S , Mortsiefer A , et al . Development of a Deprescribing manual for frail older people for use in the COFRAIL study and in primary care. Ther Adv Drug Saf 2022;13:20420986221122684. 10.1177/20420986221122684 36091625 PMC9452796

[114] Mangin D , Risdon C , Lamarche L , et al . I think this medicine actually killed my wife: patient and family perspectives on shared decision-making to optimize medications and safety. Ther Adv Drug Saf 2019;10:2042098619838796. 10.1177/2042098619838796 31057788 PMC6452573

[115] Schöpf AC , von Hirschhausen M , Farin E , et al . Elderly patients’ and Gps’ perspectives of patient–GP communication concerning Polypharmacy: a qualitative interview study. Prim Health Care Res Dev 2018;19:355–64. 10.1017/S1463423617000883 29277160 PMC6452947

[116] Knowles S , Hays R , Senra H , et al . Empowering people to help speak up about safety in primary care: using Codesign to involve patients and professionals in developing new interventions for patients with Multimorbidity. Health Expect 2018;21:539–48. 10.1111/hex.12648 29266797 PMC5867321

[117] Noël PH , Frueh BC , Larme AC , et al . Collaborative care needs and preferences of primary care patients with Multimorbidity. Health Expect 2005;8:54–63. 10.1111/j.1369-7625.2004.00312.x 15713171 PMC5060269

[118] Maidment I , Lawson S , Wong G , et al . Towards an understanding of the burdens of medication management affecting older people: the MEMORABLE realist synthesis. BMC Geriatr 2020;20:183. 10.1186/s12877-020-01568-x 32498672 PMC7272211

[119] Reeve E , Low L-F , Hilmer SN . Beliefs and attitudes of older adults and Carers about Deprescribing of medications: a qualitative focus group study. Br J Gen Pract 2016;66:e552–60. 10.3399/bjgp16X685669 27266865 PMC4979944

[120] Zechmann S , Trueb C , Valeri F , et al . Barriers and Enablers for Deprescribing among older, Multimorbid patients with Polypharmacy: an Explorative study from Switzerland. BMC Fam Pract 2019;20:64. 10.1186/s12875-019-0953-4 31088397 PMC6518702

[121] Adamson J , Hanson H , Todd A , et al . Medication work among Nonagenarians: a qualitative study of the Newcastle 85+ cohort participants at 97 years old. Br J Gen Pract 2023;73:e267–75. 10.3399/BJGP.2022.0188 36997216 PMC9997653

[122] Reeve E , Shakib S , Hendrix I , et al . Review of Deprescribing processes and development of an evidence-based, patient-centred Deprescribing process: patient-centred Deprescribing process. Brit J Clinical Pharma 2014;78:738–47. 10.1111/bcp.12386 PMC4239968 24661192

[123] Lozano-Hernández CM , López-Rodríguez JA , Leiva-Fernández F , et al . Social support, social context and Nonadherence to treatment in young senior patients with Multimorbidity and Polypharmacy followed-up in primary care. MULTIPAP study. PLOS ONE 2020;15:e0235148. 10.1371/journal.pone.0235148 32579616 PMC7314051

[124] Ose D , Mahler C , Vogel I , et al . Lets talk about medication: concordance in rating medication adherence among Multimorbid patients and their general practitioners. Patient Prefer Adherence 2012;6:839–45. 10.2147/PPA.S35498 23226007 PMC3514069

[125] Weir KR , Naganathan V , Carter SM , et al . The role of older patients’ goals in GP decision-making about medicines: a qualitative study. BMC Fam Pract 2021;22:13. 10.1186/s12875-020-01347-y 33419389 PMC7796626

[126] Junius-Walker U , Viniol A , Michiels-Corsten M , et al . Mediquit, an electronic Deprescribing tool for patients on Polypharmacy: results of a feasibility study in German general practice. Drugs Aging 2021;38:725–33. 10.1007/s40266-021-00861-7 34251594 PMC8342343

[127] Neuner-Jehle S , Zechmann S , Grundmann Maissen D , et al . Patient-provider concordance in the perception of illness and disease: a cross-sectional study among Multimorbid patients and their general practitioners in Switzerland. Patient Prefer Adherence 2017;11:1451–8. 10.2147/PPA.S137388 28860728 PMC5572955

[128] Ie K , Machino R , Albert SM , et al . Deprescribing as an opportunity to facilitate patient-centered care: A qualitative study of general practitioners and pharmacists in Japan. Int J Environ Res Public Health 2023;20:3543. 10.3390/ijerph20043543 36834238 PMC9962748

[129] Michiels-Corsten M , Gerlach N , Junius-Walker U , et al . Mediquit – an electronic Deprescribing tool: a pilot study in German primary care; Gps’ and patients’ perspectives. BMC Prim Care 2022;23:252. 10.1186/s12875-022-01852-2 36162994 PMC9511770

[130] Mangin D , Lamarche L , Agarwal G , et al . Team approach to Polypharmacy evaluation and reduction: feasibility randomized trial of a structured clinical pathway to reduce Polypharmacy. Pilot Feasibility Stud 2023;9:84. 10.1186/s40814-023-01315-0 37202822 PMC10193598

[131] Page AT , Clifford RM , Potter K , et al . The feasibility and effect of Deprescribing in older adults on mortality and health: a systematic review and Meta‐Analysis. Br J Clin Pharmacol 2016;82:583–623. 10.1111/bcp.12975 27077231 PMC5338123

[132] Verma A , Saha S , Jarl J , et al . An overview of systematic reviews and meta-analyses on the effect of medication interventions targeting Polypharmacy for frail older adults. J Clin Med 2023;12:1379. 10.3390/jcm12041379 36835915 PMC9960328

[133] Anderson LJ , Schnipper JL , Nuckols TK , et al . Effect of medication reconciliation interventions on outcomes: A systematic overview of systematic reviews. Am J Health Syst Pharm 2019;76:2028–40. 10.1093/ajhp/zxz236 31789354 PMC6885740

[134] Rieckert A , Reeves D , Altiner A , et al . Use of an electronic decision support tool to reduce Polypharmacy in elderly people with chronic diseases: cluster randomised controlled trial. BMJ 2020;369:m1822. 10.1136/bmj.m1822 32554566 PMC7301164

[135] Muth C , Uhlmann L , Haefeli WE , et al . Effectiveness of a complex intervention on Prioritising Multimedication in Multimorbidity (PRIMUM) in primary care: results of a pragmatic cluster randomised controlled trial. BMJ Open 2018;8:e017740. 10.1136/bmjopen-2017-017740 PMC5855483 29478012

[136] Hasan Ibrahim AS , Barry HE , Hughes CM . A systematic review of general practice-based pharmacists’ services to optimize medicines management in older people with Multimorbidity and Polypharmacy. Fam Pract 2021;38:509–23. 10.1093/fampra/cmaa146 33506870

[137] Croke A , Cardwell K , Clyne B , et al . The effectiveness and cost of integrating pharmacists within general practice to optimize prescribing and health outcomes in primary care patients with Polypharmacy: a systematic review. BMC Prim Care 2023;24:41. 10.1186/s12875-022-01952-z 36747132 PMC9901090

[138] Collier A , Balmer D , Dai L , et al . Older people, medication safety, and the role of the community pharmacist: a longitudinal Ethnographic study. Pharmacy Practice and Res 2023;53:18–25. 10.1002/jppr.1839

[139] Fudge N , Swinglehurst D . It’s all about patient safety’: an Ethnographic study of how Pharmacy staff construct medicines safety in the context of Polypharmacy. BMJ Open 2021;11:e042504. 10.1136/bmjopen-2020-042504 PMC7925910 33550250

[140] Hansen CR , O’Mahony D , Kearney PM , et al . Identification of behaviour change techniques in Deprescribing interventions: a systematic review and meta-analysis: behaviour change techniques in Deprescribing interventions. Br J Clin Pharmacol 2018;84:2716–28. 10.1111/bcp.13742 30129139 PMC6255994

[141] Gorman A , Rankin A , Hughes C , et al . Theoretically derived interventions aimed at improving appropriate Polypharmacy in primary care: A systematic review. Explor Res Clin Soc Pharm 2022;7:100166. 10.1016/j.rcsop.2022.100166 36039374 PMC9418988

[142] Rankin A , Gorman A , Cole J , et al . An external pilot cluster randomised controlled trial of a theory-based intervention to improve appropriate Polypharmacy in older people in primary care (Polyprime). Pilot Feasibility Stud 2022;8:203. 10.1186/s40814-022-01161-6 36088445 PMC9463515

[143] Kirwan C , Hynes L , Hart N , et al . The Multimorbidity collaborative medication review and decision making (Mycomrade) study: a pilot cluster randomised trial in two Healthcare systems. Pilot Feasibility Stud 2022;8:225. 10.1186/s40814-022-01107-y 36195963 PMC9531225

[144] Cadogan CA , Ryan C , Francis JJ , et al . Improving appropriate Polypharmacy for older people in primary care: selecting components of an evidence-based intervention to target prescribing and dispensing. Implementation Sci 2015;10:161. 10.1186/s13012-015-0349-3 PMC4647274 26573745

[145] Khezrian M , McNeil CJ , Murray AD , et al . An overview of prevalence, determinants and health outcomes of Polypharmacy. Ther Adv Drug Saf 2020;11:2042098620933741. 10.1177/2042098620933741 32587680 PMC7294476

[146] Ong SM , Lim YMF , Sivasampu S , et al . Variation of Polypharmacy in older primary care Attenders occurs at Prescriber level. BMC Geriatr 2018;18:59. 10.1186/s12877-018-0750-2 29471806 PMC5824493

[147] Fahmi A , Wong D , Walker L , et al . Combinations of medicines in patients with Polypharmacy aged 65-100 in primary care: large variability in risks of adverse drug related and emergency hospital admissions. PLoS ONE 2023;18:e0281466. 10.1371/journal.pone.0281466 36753492 PMC9907844

[148] Kardas P , Mair A , Stewart D , et al . Optimizing Polypharmacy management in the elderly: a comprehensive European Benchmarking survey and the development of an innovative online Benchmarking application. Front Pharmacol 2023;14:1254912. 10.3389/fphar.2023.1254912 37915419 PMC10616468

[149] King E , Bazargan M , Entsuah N , et al . Potentially inappropriate medication use among Underserved older Latino adults. JCM 2023;12:3067. 10.3390/jcm12093067 37176508 PMC10179006

[150] Barrio-Cortes J , Benito-Sánchez B , Villimar-Rodriguez AI , et al . Differences in Healthcare service utilization in patients with Polypharmacy according to their risk level by adjusted morbidity groups: a population-based cross-sectional study. J of Pharm Policy and Pract 2023;16:161. 10.1186/s40545-023-00665-7 38017572 PMC10683272

[151] Sawan M , Reeve E , Turner J , et al . A systems approach to identifying the challenges of implementing Deprescribing in older adults across different health-care settings and countries: a narrative review. Expert Rev Clin Pharmacol 2020;13:233–45. 10.1080/17512433.2020.1730812 32056451 PMC7309419

[152] Doherty AJ , Boland P , Reed J , et al . Barriers and Facilitators to Deprescribing in primary care: a systematic review. BJGP Open 2020;4:bjgpopen20X101096. 10.3399/bjgpopen20X101096 PMC7465575 32723784

[153] Wallis KA , Andrews A , Henderson M . Swimming against the tide: primary care physicians’ views on Deprescribing in everyday practice. Ann Fam Med 2017;15:341–6. 10.1370/afm.2094 28694270 PMC5505453

[154] Anderson K , Stowasser D , Freeman C , et al . Prescriber barriers and Enablers to minimising potentially inappropriate medications in adults: a systematic review and thematic synthesis. BMJ Open 2014;4:e006544. 10.1136/bmjopen-2014-006544 PMC4265124 25488097

[155] Lee JW , Jeong S , Han H-R , et al . Barriers and Facilitators to Deprescribing before surgery: A qualitative study of providers and older adults. Geriatr Nurs 2023;53:135–40. 10.1016/j.gerinurse.2023.07.018 37540907 PMC10528381

[156] Bujold M , Pluye P , Légaré F , et al . Decision-making and related outcomes of patients with complex care needs in primary care settings: a systematic literature review with a case-based qualitative synthesis. BMC Prim Care 2022;23:279. 10.1186/s12875-022-01879-5 36352376 PMC9644584

[157] Engels LWS , van Merode T , Heijmans M , et al . n.d. Measurement of treatment burden in patients with Multimorbidity in the Netherlands: translation and validation of the Multimorbidity treatment burden questionnaire (NL-MTBQ). Fam Pract. 10.1093/fampra/cmad100 PMC11636562 37878729

[158] Swinglehurst D , Hogger L , Fudge N . Negotiating the Polypharmacy paradox: a Video-Reflexive Ethnography study of Polypharmacy and its practices in primary care. BMJ Qual Saf 2023;32:150–9. 10.1136/bmjqs-2022-014963 PMC9985753 36854488

[159] Swinglehurst D , Fudge N . Organising Polypharmacy: unpacking medicines, unpacking meanings—an Ethnographic study. BMJ Open 2021;11:e049218. 10.1136/bmjopen-2021-049218 PMC8395269 34446490

[160] Reeve E , To J , Hendrix I , et al . Patient barriers to and Enablers of Deprescribing: a systematic review. Drugs Aging 2013;30:793–807. 10.1007/s40266-013-0106-8 23912674

[161] Cairo Notari S , Sader J , Caire Fon N , et al . Understanding Gps’ clinical reasoning processes involved in managing patients suffering from Multimorbidity: A systematic review of qualitative and quantitative research. Int J Clin Pract 2021;75:e14187. 10.1111/ijcp.14187 33783098 PMC8459259

[162] Sinnott C , Hugh SM , Boyce MB , et al . What to give the patient who has everything? A qualitative study of prescribing for Multimorbidity in primary care. Br J Gen Pract 2015;65:e184–91. 10.3399/bjgp15X684001 25733440 PMC4337307

[163] Carrier H , Zaytseva A , Bocquier A , et al . Gps’ management of Polypharmacy and therapeutic dilemma in patients with Multimorbidity: a cross-sectional survey of Gps in France. Br J Gen Pract 2019;69:e270–8. 10.3399/bjgp19X701801 30803978 PMC6428490

[164] Anderson K , Foster M , Freeman C , et al . “Negotiating “Unmeasurable harm and benefit”: perspectives of general practitioners and consultant pharmacists on Deprescribing in the primary care setting”. Qual Health Res 2017;27:1936–47. 10.1177/1049732316687732 29088989

[165] Mc Namara KP , Breken BD , Alzubaidi HT , et al . Health professional perspectives on the management of Multimorbidity and Polypharmacy for older patients in Australia. Age Ageing 2016. 10.1093/ageing/afw200 27836856

[166] Turner JP , Edwards S , Stanners M , et al . What factors are important for Deprescribing in Australian long-term care facilities? perspectives of residents and health professionals. BMJ Open 2016;6:e009781. 10.1136/bmjopen-2015-009781 PMC4800122 26966056

[167] Sinnige J , Korevaar JC , van Lieshout J , et al . Medication management strategy for older people with Polypharmacy in general practice: a qualitative study on prescribing behaviour in primary care. Br J Gen Pract 2016;66:e540–51. 10.3399/bjgp16X685681 27266862 PMC4979928

[168] Smith SM , O’Kelly S , O’Dowd T . Gps' and pharmacists' experiences of managing Multimorbidity: a ‘Pandora’s box. Br J Gen Pract 2010;60:285–94. 10.3399/bjgp10X514756 20594430 PMC2894403

[169] Laursen J , Kornholt J , Betzer C , et al . General practitioners’ barriers toward medication reviews in Polymedicated Multimorbid patients: how can a focus on the Pharmacotherapy in an outpatient clinic support Gps? Health Serv Res Manag Epidemiol 2018;5:2333392818792169. 10.1177/2333392818792169 30246058 PMC6144514

[170] Tsoi CS , Chow JY , Choi KS , et al . Medical characteristics of the oldest old: retrospective chart review of patients aged 85+ in an academic primary care centre. BMC Res Notes 2014;7:340. 10.1186/1756-0500-7-340 24897943 PMC4061508

[171] Tan YY , Papez V , Chang WH , et al . Comparing clinical trial population Representativeness to real-world populations: an external validity analysis encompassing 43 895 trials and 5 685 738 individuals across 989 unique drugs and 286 conditions in England. Lancet Healthy Longev 2022;3:e674–89. 10.1016/S2666-7568(22)00186-6 36150402

[172] Wallace E , Salisbury C , Guthrie B , et al . Managing patients with Multimorbidity in primary care. BMJ 2015;350:h176. 10.1136/bmj.h176 25646760

[173] Scott IA , Anderson K , Freeman CR , et al . First do no harm: a real need to Deprescribe in older patients. Med J Aust 2014;201:390–2. 10.5694/mja14.00146 25296059

[174] Liu BM , Kouladjian O’Donnell L , Redston MR , et al . Association of the drug burden index (DBI) exposure with outcomes: A systematic review. J American Geriatrics Society 2024;72:589–603. 10.1111/jgs.18691 38006299

[175] Hanlon JT , Schmader KE . The medication appropriateness index: A Clinimetric measure. Psychother Psychosom 2022;91:78–83. 10.1159/000521699 35158365

[176] O’Mahony D , Cherubini A , Guiteras AR , et al . STOPP/START criteria for potentially inappropriate prescribing in older people: version 3. Eur Geriatr Med 2023;14:625–32. 10.1007/s41999-023-00777-y 37256475 PMC10447584

[177] By the 2023 American Geriatrics Society Beers Criteria® Update Expert Panel . American Geriatrics society 2023 updated AGS beers criteria® for potentially inappropriate medication use in older adults. J American Geriatrics Society 2023;71:2052–81. 10.1111/jgs.18372 PMC12478568 37139824

