# Supplementary material for: Defining, identifying and addressing problematic polypharmacy within multimorbidity in primary care: a scoping review
Source: BMJ Open. 2024 May 24;14(5):e081698. doi: 10.1136/bmjopen-2023-081698 (PMC11129052; doi:10.1136/bmjopen-2023-081698)
Supplement: Supplementary data [file bmjopen-2023-081698supp001.pdf]

Supplementary file 1: Descriptions of the included 157 papers in the synthesis of the scoping review

Author & Year	Title	Country	Publication type	Key Polypharmacy definitions	Population	Intervention (if applicable)	Key findings/ conclusions
Ali et al. 2022	Interventions to address polypharmacy in older adults living with multimorbidity.	Canada	Review of reviews	• Concurrent use of multiple medications<br>• ≥5 chronic medicines	Older adults (all 64+ years) with chronic conditions taking 5 or more medication across 5 reviews	Any polypharmacy intervention in the primary care setting	• Polypharmacy interventions are associated with reductions in potentially inappropriate prescribing and improvements in medication adherence.<br>• However, there is limited evidence of their effectiveness for clinical and intermediate outcomes.
Anderson et al. 2019	A systematic overview of systematic reviews evaluating interventions addressing polypharmacy.	USA	Review of reviews	• Use of multiple medications daily<br>• Appropriate and inappropriate polypharmacy	Mainly older adults (≥ 65 years) from 6 systematic reviews (in a range of primary care, community, nursing home and secondary care settings)	Any intervention addressing polypharmacy	• Despite the low quality of evidence in the underlying primary studies, there was evidence polypharmacy interventions improved medication appropriateness.<br>• However, there was no consistent evidence of any impact on downstream patient-centred outcomes such as healthcare utilization, morbidity, or mortality.
Anderson et al. 2019	Effect of medication reconciliation interventions on outcomes: A systematic overview of systematic reviews.	USA	Review of reviews	• Many medications<br>• Focus on medication discrepancies	Adult or paediatric populations	Interventions that included medication reconciliation as a core part	• An overview of systematic reviews of medication reconciliation interventions found 9 high-quality systematic reviews.<br>• A minority of those reviews' conclusions were consistent with medication reconciliation alone having a measurable impact, and such conclusions were almost all based on very low-quality evidence.
Soler et al. 2019	Community-Level Pharmaceutical Interventions to Reduce the Risks of Polypharmacy in the Elderly: Overview of Systematic Reviews and Economic Evaluations	Brazil	Review of reviews	• Prescription of multiple drugs to the individual<br>• Adequate polypharmacy and inadequate polypharmacy	Mainly older adults (≥ 65 years) from 13 systematic reviews and 3 economic analyses (range of primary/secondary care settings)	Any pharmaceutical intervention to reduce risks of polypharmacy	• There is evidence of improvement in clinical, epidemiological, humanistic, and economic outcomes for various types of community-level pharmaceutical interventions,<br>• Differences in observed outcomes may be due to study designs, primary study sample sizes, risk of bias, difficulty in aggregating data, heterogeneity of indicators and quality of evidence
Page et al. 2016	The feasibility and effect of deprescribing in older adults on	Australia	Review and meta-analysis	• Use of many medications	34 143 older adults (≥65 years,	Any deprescribing intervention	• Although nonrandomized data suggested that deprescribing reduces mortality, deprescribing

	mortality and health: a systematic review and meta-analysis			• Focus on deprescribing	mean age 73.8) across 132 papers		was not shown to alter mortality in randomized studies.<br>• Mortality was significantly reduced when applying patient-specific interventions in randomized studies.
Hill-Taylor et al. 2016	Effectiveness of the STOPP/START (Screening Tool of Older Persons' potentially inappropriate Prescriptions/Screening Tool to Alert doctors to the Right Treatment) criteria: systematic review and meta-analysis of randomized controlled studies	Canada	Review & meta-analysis	• Focus on potentially inappropriate prescribing	1925 older adults (≥65 years) from 4 RCTs across four countries (both acute and long-term care settings)	Interventions involving prospective application of the STOPP and/or START criteria on medication profiles	• Meta-analysis found that the STOPP criteria reduced PIM rates in all four studies, but study heterogeneity (I2 = 86·7%) prevented the calculation of a meaningful statistical summary.<br>• We found evidence that use of the criteria reduces falls, delirium episodes, hospital length-of-stay, care visits (primary and emergency) and medication costs, but no evidence of improvements in quality of life or mortality.
Johansson et al. 2016	Impact of strategies to reduce polypharmacy on clinically relevant endpoints: a systematic review and meta-analysis	Austria	Review & meta-analysis	• ≥4 or ≥5 drugs<br>• Use of potentially inappropriate medication	10 980 older adults (65+ years) across 25 controlled trials (multiple settings)	Any intervention aimed at reducing inappropriate polypharmacy	• There is no convincing evidence that the strategies assessed in the present review are effective in reducing polypharmacy<br>• These strategies to reduce polypharmacy had no effect on all-cause mortality (odds ratio 1.02; 95% confidence interval 0.84, 1.23). Only single studies found improvements, in terms of reducing the number of hospital admissions, in favour of the intervention group.
Keller et al. 2024	Cumulative Update of a Systematic Overview Evaluating Interventions Addressing Polypharmacy	USA (multiple countries included)	Review & meta-analysis	• ≥5 medications<br>• Use of potentially inappropriate medication (PIMs) or potential prescribing omissions (PPOs)	Mainly older adults (mainly ≥65 years) across 179 trials (multiple settings)	Any intervention aimed at addressing polypharmacy	• There is little evidence of an association between polypharmacy-related interventions and reduced important clinical and health care use outcomes.<br>• More evidence is needed regarding which interventions are most useful and which populations would benefit most.
Kua et al. 2019	Health outcomes of deprescribing interventions among older residents in nursing homes: a systematic review and meta-analysis.	Malaysia	Review & meta-analysis	• Concurrent use of multiple medications<br>• Potentially inappropriate medications<br>• Focus on deprescribing	Older Residents (≥ 60 years) in Nursing Homes across 41 RCTs	Any deprescribing intervention (defined as either medication discontinuation, substitution, or reduction)	• Deprescribing interventions significantly reduced the number of residents with potentially inappropriate medications by 59%<br>• Compared to other deprescribing interventions, medication review–directed deprescribing had significant benefits on older residents in nursing homes. Further research is required to elicit

							other clinical benefits of medication review–directed deprescribing practice.
Seppala et al. 2022	Medication reviews and deprescribing as a single intervention in falls prevention: a systematic review and meta-analysis.	Netherlands	Review & meta-analysis	• Use of multiple medications<br>• Focus on deprescribing	Older adults (aged ≥ 60 years) across 49 studies (separate analyses for community, hospital, and long-term care settings)	Any medication review or deprescribing intervention as a single intervention in falls prevention	• In meta-analyses, no significant associations between medication reviews and fall outcomes were found in any of the settings.<br>• There was a trend for a lower number of fallers in the meta-analysis assessing medication reviews in long-term care.<br>• In a frail subgroup, medication review might be effective even as a single intervention.
Shrestha et al. 2021	Impact of deprescribing dual-purpose medications on patient-related outcomes for older adults near end-of-life: a systematic review and meta-analysis.	Australia	Review & meta-analysis	• Potentially inappropriate medications<br>• Focus on deprescribing	689 Older adults (mean 81.6-85.7 years) with at least one life-limiting illness and at least one potentially inappropriate dual-purpose medication (disease prevention and symptom control) across 5 studies	Any deprescribing intervention	• The deprescribing of DPMs lowered the risk of mortality (risk ratio (RR) = 0.59, 95% confidence interval (CI) = 0.44-0.79) and referral to acute care facilities (RR = 0.40, 95% CI = 0.22-0.73), but did not have a significant impact on the risk of falls, non-vertebral fracture, emergency presentation, unplanned hospital admission, or general practitioner visits<br>• Insufficient good-quality studies powered to confirm a benefit in terms of quality of life, physical or cognitive function, health service utilisation and adverse events.
Tasai et al. 2021	Impact of Medication Reviews Delivered by Community Pharmacist to Elderly Patients on Polypharmacy: A Meta-analysis of Randomized Controlled Trials	Thailand	Review & meta-analysis	• ≥4 prescribed medications	4633 older adults (65+ years) across 4 studies	Interventions incorporating medication reviews delivered by community pharmacist	• When compared with usual care, medication reviews provided by community pharmacist significantly reduced risk of ED visits (risk ratio = 0.68; 95% CI = 0.48–0.96)
Alldred et al. 2016	Interventions to optimise prescribing for older people in care homes.	UK	Review	• ≥4 regular medicines<br>• Focus on appropriate & inappropriate polypharmacy (Beers, STOPP/START, MAI)	10,953 older residents (≥ 65 years) in 355 care homes in 10 countries.	Any intervention to optimise overall prescribing	• Could not draw robust conclusions from the evidence due to variability in design, outcomes and results<br>• The interventions implemented in the studies in this review led to the identification and resolution of medication-related problems and improvements in medication appropriateness

							• Evidence of a consistent effect on resident-related outcomes was not found.
Anderson et al. 2014	Prescriber barriers and enablers to minimising potentially inappropriate medications in adults: a systematic review and thematic synthesis	Australi a	Review	• Use of multiple medications concurrently<br>• ≥5 prescription, over-the-counter or complementary medicines every day<br>• potentially inappropriate medications	Adults of all ages, with some focus on older adults with chronically prescribed potent ially inappropriate medications across 21 studies (18 in primary care, 2 in residential care, 1 in secondary care)	N/A	• Barriers and enablers to minimising PIMs emerged within four analytical themes: problem awareness; inertia secondary to lower perceived value proposition for ceasing versus continuing PIMs; self-efficacy regarding personal ability to alter prescribing; and feasibility of altering prescribing in routine care given external constraints.<br>• The first 3 themes are intrinsic to the prescriber (e.g., beliefs, attitudes, knowledge, skills, behaviour) and the fourth is extrinsic (e.g., patient, work setting, health system and cultural factors).
Cadogan et al. 2013	Appropriate Polypharmacy and Medicine Safety: When Many is not Too Many	UK (NI), Ireland	Review	• Use of multiple medicines<br>• Balancing 'too little' and 'too many'<br>• Focus on appropriate polypharmacy	Middle-aged and older population with multimorbidity	N/A	• Differentiating between 'many' drugs and 'too many' drugs is proving ever more complex.<br>• Conceptualising polypharmacy as a numerical threshold is unhelpful because it fails to consider that the appropriate number of medicines varies according to individual patients' clinical needs<br>• Increased use of the term 'appropriate polypharmacy' could encourage greater consideration of the clinical context underlying prescribing, as well as increased acceptance that the prescribing of multiple medicines is 'potentially problematic rather than always inappropriate'.
Cairo Notari et al. 2021	Understanding GPs' clinical reasoning processes involved in managing patients suffering from multimorbidity: A systematic review of qualitative and quantitative research	Switzerl and	Review	• Multiple medicines and associated harmful effects<br>• Focus on deprescribing	Adults with multimorbidity (≥2 chronic conditions) across 32 studies	N/A	• In the absence of guidelines adapted to multimorbidity, there is no single correct plan, but competing priorities and unavoidable uncertainties.<br>• Thus, GPs have to consider and weigh multiple factors simultaneously. In the context of multimorbidity,<br>• GPs describe their reasoning as essentially intuitive and seem to perceive it as less accurate.<br>• These clinical reasoning processes are nevertheless more analytical than they might think and rooted in deep knowledge of the individual patient.

Cole et al. 2023	Interventions to improve the appropriate use of polypharmacy for older people	UK (Ireland), included trials from 19 countries	Review	• Polypharmacy (4 or more medicines), which used a validated tool to assess prescribing appropriateness<br>• Use of potentially inappropriate medication (PIMs) or potential prescribing omissions (PPOs)	18,073 older adults (aged ≥ 65) from 38 studies	Any intervention to improve appropriate polypharmacy	• It is unclear whether interventions to improve appropriate polypharmacy resulted in clinically significant improvement (including medication appropriateness, PIMs, PPOs, hospital admissions, quality of life, medication related problems).<br>• Since the last update in 2018, there appears to have been an increase in the number of studies seeking to address potential prescribing omissions and more interventions being delivered by multidisciplinary teams.
Cooper et al. 2015	Interventions to improve the appropriate use of polypharmacy in older people: a Cochrane systematic review	UK (Northern Ireland)	Review	• ≥4 regular medicines<br>• Focus on appropriate & inappropriate polypharmacy (Beers, STOPP/START, MAI)	Older adults (≥ 65 years) with ≥ 1 long-term condition who were receiving polypharmacy (≥ 4 regular medicines) from 12 studies (5 in community care) across 5 countries	All interventions improving appropriate polypharmacy.	• The included 12 interventions demonstrated improvements in appropriate polypharmacy based on reductions in inappropriate prescribing.<br>• However, it was unclear if interventions resulted in clinically significant improvements
Croke et al. 2023	The effectiveness and cost of integrating pharmacists within general practice to optimize prescribing and health outcomes in primary care patients with polypharmacy: a systematic review	Ireland	Review	• ≥5 regular medications<br>• Potentially inappropriate prescribing (any explicit/implicit criteria)	23,516 community dwelling adults (≥18 years) and in the primary care setting with polypharmacy (23 studies in 3 continents)	Any intervention involving pharmacist integration within General Practice (Including remote integration)	• Pharmacist integration probably reduced PIP and number of medications however, there was no clear effect on other patient outcomes<br>• While interventions in a small number of studies appeared to be cost-effective, further robust, well-designed cluster RCTs with economic evaluations are required to determine cost-effectiveness of pharmacist integration.
Dills et al. 2018	Deprescribing Medications for Chronic Diseases Management in Primary Care Settings: A Systematic Review of Randomized Controlled Trials	USA	Review	• Use of multiple prescription drugs<br>• Focus on deprescribing	Adults in a range of settings (primary care, assisted living, nursing home, outpatient and acute care settings only if medications for chronic disease	Deprescribing interventions involving chronic medical and mental health conditions managed by primary care professionals	• Deprescription may be successful and effective in select classes of drugs, with collaboration of clinical pharmacists for patient and provider education, and patient-specific drug recommendations, complemented by close clinical follow-up to detect early signs of exacerbation of chronic diseases.<br>• Deprescription may (1) require expensive intensive, ongoing interventions by clinical teams; (2) not lead to expected outcomes such as improved falls rate, cognition, and quality of

					were deprescribed		life, or a lower admission rate; and (3) have unexpected adverse outcomes affecting patients' quality of life.
Doherty et al. 2020	Barriers and facilitators to deprescribing in primary care: a systematic review	UK	Review	• Multiple concurrent medications<br>• ≥4 of any type of medications<br>• Focus on deprescribing	Adults (aged ≥18 years) with multimorbidity and polypharmacy (≥2 long-term health conditions), across 40 studies from 14 countries	Any deprescribing intervention	A whole system, patient-centred approach to safe deprescribing interventions is required, involving key decision-makers, healthcare professionals, patients, and carers.
Hasan Ibrahim et al. 2021	A systematic review of general practice-based pharmacists' services to optimize medicines management in older people with multimorbidity and polypharmacy	UK (Northern Ireland)	Review	• Concomitant use of ≥4 medicines<br>• Focus on medicines optimisation	Older adults (≥65 years) with multimorbidity (≥2 long-term conditions) across 7 studies in 5 countries	Practice-based pharmacists optimizing medicines management for older people with both multimorbidity and polypharmacy	• All studies employed pharmacist-led medication reviews accompanied by recommendations agreed and implemented by GPs.<br>• The limited available evidence suggested that, in collaboration with other practice team members, had mixed effects on outcomes focused on optimizing medicines management for older people.<br>• Most included studies were of poor quality and data to estimate the risk of bias often missing.
Heaton et al.	Person-centred medicines optimisation policy in England: an agenda for research on polypharmacy.	UK	Review	• Use of multiple medications<br>• 'Appropriate' and 'problematic' polypharmacy	Patients using multiple medicines in the UK	N/A	Reports varied in their inclusion of patient perspectives and person-centred care values, and in the extent to which they drew on evidence from research on patients' experiences of polypharmacy and medicines use.
Ibrahim et al. 2021	A systematic review of the evidence for deprescribing interventions among older people living with frailty.	UK	Review	• ≥5 regular medications<br>• Focus on deprescribing	657 older adults with frailty (mean age 79–87 years)	Any deprescribing medication review intervention accounting for at least 50% of changes	• All studies described medication-related outcomes and reported a reduction in potentially inappropriate medications and total number of medications per-patient.<br>• Feasibility of deprescribing in 4 studies which showed that 72–91% of recommendations made were implemented.<br>• 2 studies evaluated and reported acceptability of their interventions and 2 described cost savings.<br>• But there was a paucity of research about the impact of deprescribing in older people living with frailty

Kaufmann et al. 2014	Inappropriate prescribing: a systematic overview of published assessment tools.	Switzerland	Review	• Use of multiple medicines<br>• Focus on appropriate and inappropriate prescribing	Adults (majority older adults) with potentially inappropriate prescribing	N/A	• Out of 46 tools to assess inappropriate prescribing. 28 (61%) were explicit, 8 (17%) were implicit and 10 (22%) used a mixed approach.<br>• 36 tools named older people as target patients and 10 (22%) tools did not specify the target age group.<br>• Only 9 tools (19.5%) focused on patients in ambulatory care and 6 (13%) were developed for use in long-term care. 27 (59%) tools did not specify the health care setting.
Khezrian et al. 2020	An overview of prevalence, determinants and health outcomes of polypharmacy.	UK (Scotland)	Review	• No generally accepted definition<br>• Numerical (e.g. ≥ 5 medications concurrently or excessive polypharmacy ≥ 10 medications concurrently)<br>• Descriptive methods (patients visiting multiple pharmacies to obtain medications' and 'use of additional medications to correct adverse effects)<br>• Appropriate vs inappropriate polypharmacy	Older adults	N/A	• Polypharmacy was most often defined in terms of the number of medications that are being taken by an individual at any given time.<br>• Our review showed that the prevalence of polypharmacy varied between 10% to as high as around 90% in different populations.<br>• Chronic conditions, demographics, socioeconomics and self-assessed health factors were independent predictors of polypharmacy.<br>• Polypharmacy was reported to be associated with various adverse outcomes after adjusting for health conditions.
Laberge et al. 2021	Economic Evaluations of Interventions to Optimize Medication Use in Older Adults with Polypharmacy and Multimorbidity: A Systematic Review	Canada	Review	• Consumption of multiple medications simultaneously<br>• use of ≥5 medications<br>• potentially inappropriate medications and adverse drug reactions	6375 older adults (≥65 years) with multimorbidity (2+ conditions) from 11 studies across 8 countries (settings included primary care, nursing home, pharmacies, and secondary care)	Any intervention aimed at optimizing medication use	• Interventions were generally associated with a reduction in medication expenditure. The benefits of the intervention in terms of clinical outcomes remain limited.<br>• Five studies were cost-benefit analyses, which had a net benefit that was either null or positive.<br>• However, the quality of the studies was generally low.

Lee et al. 2020	Intervention elements to reduce inappropriate prescribing for older adults with multimorbidity receiving outpatient care: a scoping review.	Singapore	Review	• ≥5 medications daily<br>• Focus on potentially inappropriate prescribing	Older adults with multimorbidity	Interventions to reduce inappropriate prescribing	• 4 intervention elements were identified. An average of 2-3 to three elements were adopted in each intervention. The three most frequently adopted intervention elements were medication review (70%), training (26.3%) and tool/instrument(s) (22.5%).<br>• The 14 intervention elements were mapped onto five intervention functions: 'education', 'persuasion', 'training', 'environmental restructuring' and 'enablement'
Lee et al. 2022	Applicability of explicit potentially inappropriate medication lists to the Australian context: A systematic review.	Australia	Review	Focus on potentially inappropriate medications	Older adults prescribed potentially inappropriate medications	N/A	• Applicability of each explicit list ranged from 50-96% according to medications available in Australia and 25-83% according to medications available under subsidy.<br>• Pooling data from different lists may help to identify potentially inappropriate medications that may be applicable to local settings.
Lee et al. 2023	Factors associated with potentially inappropriate prescribing among older persons in primary care settings: Systematic review	Singapore	Review	• Focus on Potentially inappropriate prescribing (PIP)	2,893,925 older adults (≥60 years, mean 70-84 years) from 25 studies	N/A	• Risk factors of PIP could be classified into patient, physician and system factors.<br>• Patient factors were related to patient demographics (advanced age, lower education level and lower socioeconomic status), medical comorbidities (polypharmacy and multimorbidity) and lifestyle factors (unhealthy habits and use of over-the-counter medications).<br>• Physician and system factors included older, male, solo general practitioner (GP), higher number of visits of pharmaceutical sales representatives to GP, centrally located GP practice, and smaller number of older patients following up with GP, and medication source from public health system.
Lucchetti et al. 2017	Inappropriate prescribing in older persons: A systematic review of medications available in different criteria	Brazil	Review	Focus on potentially inappropriate medications	Older adults prescribed potentially inappropriate medications	N/A	• More than 85% used a Delphi method.<br>• There were 729 different medications/classes reported in all criteria.<br>• Diazepam was included in all 14 criteria followed by amitriptyline (13 criteria) and doxepin (12 criteria).<br>• Benzodiazepines, NSAIDs, antihistamines and antipsychotics were the most common drugs reported as potentially inappropriate for older persons.

Maidment et al. 2020	Towards an understanding of the burdens of medication management affecting older people: the MEMORABLE realist synthesis	UK	Review	• ≥5 medications<br>• Or if less – a complex regime	Older adults (≥ 60) years with multimorbidity (≥2 long term conditions) and polypharmacy	Medication management as a extended, complex process	• Older people and family carers often find medication management challenging and burdensome particularly for complex regimens.<br>• Practitioners need to be aware of this potential challenge, and work with older people and their carers to minimise the burden associated with medication management.
Mair et al. 2020	Addressing the Challenge of Polypharmacy.	UK (Scotland)	Review	• Use of multiple medications<br>• Appropriate and inappropriate polypharmacy	Older adults with multimorbidity and polypharmacy	N/A	• Guidance is needed to support patients and clinicians in defining and achieving realistic goals of drug treatment, and system change is necessary to aid implementation.
Masnoon et al 2018	Tools for Assessment of the Appropriateness of Prescribing and Association with Patient-Related Outcomes: A Systematic Review.	Australia	Review	• Multiple medicines prescribed together<br>• Focus on appropriate and inappropriate prescribing	Adults (mainly ≥45 years) with multimorbidity and polypharmacy	N/A	• Out of the 42 tools, 78.6% (n = 33) provided guidance around stopping inappropriate medications, 28.6% (n = 12) around starting appropriate medications, 61.9% (n = 26) were explicit (criteria based) and 31.0% (n = 13) had been externally validated, with hospitalisation being the most commonly used patient-related outcome (n = 9, 21.4%).<br>• Less than 50% of available tools have been externally validated, limiting their use in clinical practice.
Masnoon et al.	What is polypharmacy? A systematic review of definitions.	Australia	Review	• 138 definitions of polypharmacy and associated terms were obtained.<br>• 111 numerical only definitions (80.4% of all definitions), 15 numerical definitions which incorporated a duration of therapy or healthcare setting (10.9%) and 12 descriptive definitions (8.7%).	Older adults with multimorbidity and polypharmacy	N/A	• Polypharmacy definitions were variable.<br>• Numerical definitions of polypharmacy did not account for specific comorbidities present and make it difficult to assess safety and appropriateness of therapy in the clinical setting.
Michiels-Corsten et al. 2020	Generic instruments for drug discontinuation in primary care: A systematic review	Germany	Review	• Concurrent use of several (mostly ≥5) long-term medications	Adults in primary care	Any generic guiding instruments for drug discontinuation in	• Instruments revealed diverging emphases on the stages of deprescribing, i.e. preparation, drug evaluation, decision-making and implementation. Accordingly, 3 types of instruments emerged: general frameworks,

				• Potentially inappropriate medications<br>• Focus on discontinuation/ deprescribing		patients with polypharmacy in the primary care setting.	detailed drug assessment tools and comprehensive discontinuation guidelines.<br>• There is still a need for practical and user-friendly tools that support physicians in communicational aspects, visualise trade-offs and also enhance patient involvement.
Monégat et al, 2014	Polypharmacy: definitions, measurement and stakes involved	France	Review	Simultaneous, cumulative, and continuous polypharmacy) examined according to different thresholds – mainly ≥ 5 medications	69,324 older adults (aged 75+) from French GPs	N/A	• These definitions can used together, provide a broader overview of the use of medication.<br>• However, if polypharmacy is generally related to inappropriate prescribing, it is not sufficient on its own to identify them. Counting the number of medications does not allow distinguishing between those that are justified for a given pathology and those that are not.
Rankin et al. 2018	Interventions to improve the appropriate use of polypharmacy for older people.	UK (Ireland), trials from 12 countri es	Review	Polypharmacy (4 or more medicines), which used a validated tool to assess prescribing appropriateness	28,672 older adults (aged 65+) from 32 studies	Any intervention to improve appropriate polypharmacy	• Unclear whether interventions resulted in clinically significant improvements<br>• May be slightly beneficial in terms of reducing potential prescribing omissions; but this effect estimate is based on only two studies, which had serious limitations in terms of risk bias.
Reeve et al. 2022	Deprescribing medicines in older people living with multimorbidity and polypharmacy: the TAILOR evidence synthesis	UK	Review	• Concurrent use of multiple medicines in a single person<br>• Use of ≥5 medications<br>• Appropriate and problematic polypharmacy<br>• Focus on deprescribing	Adults (aged ≥ 50 years) with multimorbidity (≥2 conditions)	Deprescribing interventions	• Deprescribing is a complex intervention<br>• Need to integrate patient-centred and contextual factors into best practice models.<br>• 34 context-mechanism-outcome configurations describe the knowledge work of tailored prescribing under eight headings related to organisational, health-care professional and patient factors, and interventions to improve deprescribing.<br>• Robust tailored deprescribing requires attention to providing an enabling infrastructure, access to data, tailored explanations, and trust.
Reeve et al. 2013	Patient barriers to and enablers of deprescribing: a systematic review	Australi a	Review	• Many medicines<br>• Focus on potentially inappropriate medication and deprescribing	Mainly adult with some paediatric studies across 21 studies.	Stopping a medication/ deprescribing	• Three themes: disagreement/ agreement with 'appropriateness' of cessation, absence/presence of a 'process' for cessation, and negative/positive 'influences' to cease medication, were identified as both potential barriers and enablers, with 'fear' of cessation and 'dislike' of medications as a fourth barrier and enabler, respectively.<br>• The most common barrier/enabler identified was 'appropriateness' of cessation, with 15

							studies identifying this as a barrier and 18 as an enabler.
Reeve et al. 2014	Review of deprescribing processes and development of an evidence-based, patient-centred deprescribing process	Australia	Review	• Many medicines<br>• Focus on inappropriate medication use and deprescribing	Mainly focussed on older adults (≥65 years) with one study range 25-75) with polypharmacy across 10 studies	Deprescribing	• The developed patient-centred deprescribing process, which is a 5-step cycle: gaining a comprehensive medication history, identifying potentially inappropriate medications, determining whether the potentially inappropriate medication can be ceased, planning the withdrawal regimen (e.g. tapering where necessary) and provision of monitoring, support and documentation.<br>• This process focuses on engaging patients throughout the process, with the aim of improving long-term health outcomes.
Reeve et al. 2015	A systematic review of the emerging definition of 'deprescribing' with network analysis: implications for future research and clinical practice	Australia	Review	• Use of multiple medications<br>• Focus on deprescribing	Older adults prescribed inappropriate medications	N/A	• 8 characteristics of definitions: stop/withdraw/cease/discontinue, aspect of prescribing included e.g. long term therapy/ inappropriate medications, use of the term 'process' or 'structured', withdrawal is planned/supervised/judicious, involving multiple steps, includes dose reduction/ substitution/tapering, desired goals/ outcomes.<br>• "Deprescribing is the process of withdrawal of an inappropriate medication, supervised by a health care professional with the goal of managing polypharmacy and improving outcomes."
Riordan et al. 2016	The effect of pharmacist-led interventions in optimising prescribing in older adults in primary care: A systematic review	Ireland	Review	• Focus on potentially inappropriate prescribing and medication related problems	Community-dwelling older adults (≥65 years) across 5 studies	Any pharmacist-led intervention designed to reduce potentially inappropriate prescribing or improve medication appropriateness in primary care.	• Overall, this review demonstrates that pharmacist-led interventions may improve prescribing appropriateness in community-dwelling older adults.<br>• However, the quality of evidence is low.
Sanchez-Fidalgo et al. 2017	Prevalence of drug interactions in elderly patients with multimorbidity in primary care.	Spain	Review	• Use of multiple medications<br>• Focus on drug interactions	Older adults (aged 65+) in primary care	N/A	• The prevalence of drug-drug interactions in the elderly with multimorbidity is high, with ACEIs, diuretics and NSAID being the most common therapeutic groups involved

Sawan et al. 2020	A systems approach to identifying the challenges of implementing deprescribing in older adults across different health-care settings and countries: a narrative review	Australia	Review	• Medications for which the risk outweighs the benefit in the individual<br>• ≥5 regularly prescribed medications<br>• Focus on deprescribing	Older adults (≥ 65 years) with multimorbidity (≥2 chronic diseases) and polypharmacy	N/A	• Deprescribing intervention studies are inherently heterogeneous because of the complexity of interventions employed and often do not reflect the real-world.<br>• Process evaluations in deprescribing intervention studies are needed to determine the contextual factors that are important to the translation of the interventions in the real-world.<br>• Deprescribing interventions may need to be individually tailored to target the unique barriers and opportunities to deprescribing in different clinical settings.<br>• Introduction of national policies to encourage deprescribing may be beneficial but need to be evaluated to determine if there are any unintended consequences.
Schiavo et al. 2022	A comprehensive look at explicit screening tools for potentially inappropriate medication: A systematic scoping review.	Brazil	Review	Focus on potentially inappropriate medications	Older adults prescribed potentially inappropriate medications	N/A	• Fifty-eight tools reported 614 PIMs and 747 PIMs–interactions.<br>• Limited overlap between the tools was observed: 123 (69.1%) of 178 therapeutic alternatives proposed by the tools were considered inappropriate by other tools, and 222 (36.1%) of the 614 PIMs identified were named as being inappropriate only once.<br>• Only 21 tools were developed by a Delphi panel technique associated with systematic review.<br>• The PIMs listed as essential medication in Brazil and by the WHO were 30.6% and 23.3% of the total reported, respectively.
Scott et al. 2017	Review of structured guides for deprescribing	Australia	Review	• Appropriate and inappropriate polypharmacy<br>• Focus on deprescribing	Older adults prescribed potentially inappropriate medications in both secondary and primary care	N/A	• The 7 included guides had considerable heterogeneity, with some guides constituting little more than a set of principles while others entail detailed processes and sub-steps which addressed multiple determinants of drug appropriateness<br>• Evidence of effectiveness for each guide was limited in that none have been evaluated in RCTs, and pilot or feasibility studies have involved relatively small samples<br>• More research is needed for determining effectiveness and ease of use in routine clinical practice, especially in primary care settings.

Sirois et al.	Polypharmacy definitions for multimorbid older adults need stronger foundations to guide research, clinical practice and public health	Canada	Review	• > 46 definitions of polypharmacy<br>• Several thresholds based on count<br>• The majority of the publications (58%) used a minimal threshold of 5 medications.<br>• Heterogeneous qualitative definitions, mostly stating "more drugs than needed".	Older adults with multimorbidity and polypharmacy	N/A	• The wide variety of definitions for polypharmacy precludes comparisons, appropriate identification, and management of polypharmacy in multimorbid older adults.<br>• Standardized definitions would allow more coherent judgments regarding the individual and collective stakes of polypharmacy.
Taghy et al. 2020	Failure to Reach a Consensus in Polypharmacy Definition: An Obstacle to Measuring Risks and Impacts—Results of a Literature Review.	France	Review	2 main approaches:<br>• Quantitative, applying varying thresholds and types of polypharmacy based on the medication number<br>• Qualitative, based on the clinical indications and effects of a given drug regimen, with a growing number of descriptive characteristics	Polypharmacy in the elderly and the general population (1 included review in children also).	N/A	• The term “inappropriate” is increasingly associated with polypharmacy especially in studies that aimed to use this definition to identify possible solutions for healthcare providers in the field related to aging.<br>• High variability and an evolution in the approaches defining “polypharmacy” in the absence of a consensus following standardized criteria. That makes it very difficult to estimate and measure the outcomes associated with this phenomenon.
Ulley et al. 2019	Deprescribing interventions and their impact on medication adherence in community-dwelling older adults with polypharmacy: a systematic review	UK	Review	• Use of any potentially inappropriate medication<br>• Focus on deprescribing<br>• Focus on adherence	Adults (46-97 years) that are community dwelling from 22 studies in 13 countries	Any deprescribing intervention	• There is insufficient evidence to show that deprescribing improves medication adherence. Only 13 studies (of 22) reported adherence of which only 5 were randomised controlled trials.<br>• Adherence was reported as a secondary outcome in all but one study.
Verma et al. 2023	An Overview of Systematic Reviews and Meta-Analyses on the Effect of Medication Interventions Targeting	Sweden	Review	• Use of multiple medications<br>• ≥5 or ≥10 medications for more than 90 days	Frail older patients (≥60 years)	Any strategy targeting polypharmacy	• Six systematic reviews reported a statistically significant reduction in the number of inappropriately prescribed medications.<br>• Medication reviews help in reducing the use of inappropriate medications in frail older adults

	Polypharmacy for Frail Older Adults						• But there is insufficient evidence in terms of frailty score and hospital admissions.
Vrdoljak et al. 2015	Medication in the elderly - considerations and therapy prescription guidelines.	Croatia	Review	More than a few medications potentially inappropriate medications	Older adults with multimorbidity and polypharmacy	N/A	• Lists (e.g. START/STOPP and Beers) are an important practical support in a GP's everyday work.<br>• Implementation of such therapeutic aids reduces the possibility of medical error and minimizes the chance of an inappropriate prescription for this vulnerable population stratum.
Del Cura-González et al. 2022	How to Improve Healthcare for Patients with Multimorbidity and Polypharmacy in Primary Care: A Pragmatic Cluster-Randomized Clinical Trial of the MULTIPAP Intervention	Spain	RCT (cluster)	• Simultaneous consumption of ≥ 5 drugs<br>• Potentially inappropriate medication<br>• Focus on appropriateness (MAI)	593 older adults (between 65-74 years) with multimorbidity (≥3 diseases) and polypharmacy (≥5 drugs) during the last three months	MULTIPAP intervention: GPs e-training and GP-patient-centred interview.	• The intervention significantly improved medication appropriateness.<br>• The observed quality of life improvement was not significant.<br>• GPs e-training in multimorbidity has shown to be feasible and well accepted by the professionals.
Jager et al. 2017	Impact of a tailored program on the implementation of evidence-based recommendations for multimorbid patients with polypharmacy in primary care practices-results of a cluster-randomized controlled trial.	German y	RCT (Cluster)	More than 4 drugs	273 adults (aged >50 years, suffering from at least 3 chronic diseases, receiving more than 4 drugs, and being at high risk for medication-related events according to the assessment of the treating GP)	The tailored program consisted of a workshop for GPs and health care assistants, educational materials and reminders for patients, and the elaboration of implementation action plans.	• The tailored program may improve implementation of medication counselling and brown bag review, whereas the use of medication lists and medication reviews did not improve.<br>• No effect of the tailored program on the combined primary outcome could be substantiated
Jungo et al. 2023	Optimising prescribing in older adults with multimorbidity and polypharmacy in primary care (OPTICA): cluster randomised clinical trial.	Switzerl and	RCT (cluster)	• ≥5 long-term medications<br>• Focus on appropriate polypharmacy and potentially inappropriate medications – STOPP/START	323 older adults (≥ 65 years) with ≥3 conditions in Swiss primary care	eCDSS – with STOPP/START and STRIPA – a 6-step medication review conducted by GPs, followed by shared decision making	• No evidence of improvement in appropriateness of medication or a reduction in prescribing omissions at 12 months<br>• On average, one recommendation to stop or start a medication was reported to be implemented per patient.<br>• At 12 months, the results of the intention-to-treat analysis of the improvement in appropriateness of medication (odds ratio 1.05, 95% confidence interval 0.59 to 1.87) and the

							number of prescribing omissions (0.90, 0.41 to 1.96) were inconclusive.
McCarthy et al. 2022	GP-delivered medication review of polypharmacy, deprescribing, and patient priorities in older people with multimorbidity in Irish primary care (SPPiRE Study): A cluster randomised controlled trial	Ireland	RCT (Cluster)	• Multiple medicines use<br>• Higher levels of polypharmacy ≥ 15 medicines<br>• Focus on potentially inappropriate prescribing (STOPP/START)	404 older adults (≥65 years) taking ≥15 regular medicines from 51 GP practices	SPPiRE website: (educational module; template for an individualised patient med review that identified PIP, deprescribing opportunities, and patient priorities	The SPPiRE intervention resulted in a small but significant reduction in the number of medicines but no evidence of a clear effect on PIP.<br>At 6-month follow-up, both intervention and control groups had reductions in the numbers of medicines with a small but significantly greater reduction in the intervention group<br>Less than 2% of drug withdrawals in the intervention group led to a reported adverse drug events.
Muth et al. 2018	Effectiveness of a complex intervention on Prioritising Multimedication in Multimorbidity (PRIMUM) in primary care: results of a pragmatic cluster randomised controlled trial	Germany	RCT (Cluster)	≥5 long-term drug prescriptions with systemic effects	505 older adults (≥60 years) with multimorbidity (≥3 chronic conditions) across 72 general practices in Hesse, Germany.	Healthcare assistant conducted a checklist-based interview with patients on medication-related problems and reconciled their medications. Then using a computerised decision support system, the GP optimised medication	• This study found the complex intervention to have no significant effects in older patients with multimorbidity and polypharmacy in general practice.<br>• At baseline, many patients already received appropriate prescriptions and enjoyed good quality of life and functional status.
Rieckert et al. 2020	Use of an electronic decision support tool to reduce polypharmacy in elderly people with chronic diseases: cluster randomised controlled trial.	Germany	RCT (Cluster)	• ≥8 drugs<br>• Focus on inappropriate polypharmacy and adverse drug events	3904 older adults (≥75 years) using ≥8 medications across 359 practices (4 countries)	An electronic decision support tool comprising a comprehensive drug review to support general practitioners in deprescribing potentially inappropriate and non-evidence-based drugs.	• In intention-to-treat analysis, a computerised decision support tool for comprehensive drug review of elderly people with polypharmacy showed no conclusive effects on the composite of unplanned hospital admission or death by 24 months.<br>• A reduction in drugs was achieved without detriment to patient outcomes.

Schafer et al. 2018	Narrative medicine-based intervention in primary care to reduce polypharmacy: results from the cluster-randomised controlled trial MultiCare AGENDA	Germany	RCT (Cluster)	• Co-prescription of multiple medications<br>• Focus on the patient's perspectives	604 older adults (mean age 73 years) from 55 practices in 3 areas in Germany	GPs had 3x 30 min consultations.<br>1. aimed at identifying treatment targets and priorities of the patient.<br>2. 'brown bag' review<br>3. discuss goal attainment and future treatment targets.	Intensifying the doctor–patient dialogue and discussing the patient's agenda and personal needs did not lead to a reduction of medication intake and did not alter health-related quality of life.
Zechmann et al. 2020	Effect of a patient-centred deprescribing procedure in older multimorbid patients in Swiss primary care - A cluster-randomised clinical trial	Switzerland	RCT (cluster)	• Multiple medications<br>• ≥5 drugs for ≥6 months<br>• Focus on number, but also patient safety and QoL	334 older adults (≥60 years, mean 76.2) with multimorbidity across 46 GPs in North Switzerland	GP training encouraging the use of a validated deprescribing-algorithm including shared-decision-making	• The patient-centred deprescribing procedure is effective immediately after the intervention, but not after 6 and 12 months.<br>• Further research needs to determine the optimal interval of repeated deprescribing interventions for a sustainable effect on polypharmacy at mid- and long-term.
Koberlein-Neu et al. 2016	Interprofessional medication management in patients with multiple morbidities.	Germany	RCT (cluster, stepped wedge)	• Many medications<br>• ≥5 long-term medications (>3months)<br>• Focus on drug related problems	142 older adults (mean age 76.8 years) with multimorbidity (≥ 3 conditions) in 2 regions in Germany	Medication management and case management (including specialist home visit and pharmacist input)	• Interprofessional collaboration increased medication safety.<br>• Working across disciplinary boundaries allowed for a decrease in drug-related problems and brought up aspects outside the purview of the primary care physician.
Adamson et al. 2023	Medication work among nonagenarians: a qualitative study of the Newcastle 85+ cohort participants at 97 years old.	UK	Qualitative study (interviews)	• Use of multiple medicines	296 older adults (≥ 65 years)	Semi-structured interviews	• This study has shown a high level of acceptance of the work associated with medications among this group and trust in the prescribers to provide the most appropriate care.<br>• Medicines optimisation should build on this trust and be presented as personalised, evidence-based care.
Ie et al. 2023	Deprescribing as an Opportunity to Facilitate Patient-Centered Care: A Qualitative Study of	Japan	Qualitative study (focus group interviews)	• Focus on deprescribing	Older adults with multimorbidity and polypharmacy	8 Interview focus groups to 36 professionals (including 19 GPs	• Healthcare providers acted on the basis of their attitudes and beliefs on deprescribing, the influence of subjective norms, and perceived behavioural control for deprescribing.

	General Practitioners and Pharmacists in Japan.					and 16 clinical pharmacists)	• These processes are influenced by factors such as drug class, prescribers, patients, deprescribing experience, and environment/education.<br>• Healthcare providers' attitudes, beliefs, and behavioural control (along with deprescribing strategies) evolve in a dynamic interplay with experience, environment, and education
Knowles et al. 2018	Empowering people to help speak up about safety in primary care: Using codesign to involve patients and professionals in developing new interventions for patients with multimorbidity	UK	Qualitative (workshop)	• Large number of medications<br>• Complex medication schedule	11 patients with multimorbidity or carers (plus a second workshop with five HCPs)	An intervention to empower patients and carers to raise safety issues in primary care	• Both patients and professionals prioritized polypharmacy as a threat to safety.<br>• Findings emphasized the limited capacity of patients with multimorbidity and the need for services to proactively offer support to reduce the burden of managing complex treatment regimes.<br>• There is a need for accessible reminders to support medication adherence and medication reviews for particularly vulnerable patients conducted with pharmacists within GP practices.
Rozsnyai et al. 2020	What do older adults with multimorbidity and polypharmacy think about deprescribing? The LESS study - a primary care-based survey	Switzerl and	Qualitative (questionnai re)	• ≥5 chronic medications<br>• Focus on inappropriate medications and deprescribing	300 older adults (≥70 years,) with multimorbidity (≥3 chronic conditions) and polypharmacy	Deprescribing	• The majority of participants (77%) were willing to deprescribe one or more of their medicines if their doctor said it was possible. There was no association with sex, age or the number of medicines and willingness to deprescribe.<br>• Increased willingness to deprescribe was linked to a good relationship with their GP and that they would feel that deprescribing was safe and if new studies showed an avoidable risk.<br>• The most common barriers towards deprescribing were patients feeling well on their current medicines and being convinced that they need all their medicines.
Engels et al. 2023	Measurement of treatment burden in patients with multimorbidity in the Netherlands: translation and validation of the Multimorbidity Treatment Burden Questionnaire (NL-MTBQ).	Netherl ands	Qualitative (questionnai re with interviews)	• Focus on treatment burden<br>• Inappropriate polypharmacy	959 adults (17 - 96 years, mean = 69.9 years)	Multimorbidity Treatment Burden Questionnaire	• Median global NL-MTBQ score was 3.85 (interquartile range 0–9.62), representing low treatment burden.<br>• Factor analysis supported a single-factor structure.<br>• The Dutch version of the 13-item MTBQ is a single-structured, valid, and compact patient-reported outcome measure to assess treatment burden in primary care patients with multimorbidity.

Anthierens et al. 2010	Qualitative insights into general practitioners views on polypharmacy	Belgium	Qualitative (interviews)	• concomitant use of ≥3 drugs<br>• use of more drugs than indicated	People with polypharmacy	Interviews with 65 GPs	• It is a problem in their older patient population, especially because of the risk of adverse drug reactions, interactions and lowered adherence.<br>• Difficulties in keeping an overview of the exact medication intake<br>• Patients' strong belief in their medication and self-medication are seen as important barriers<br>• According to the respondents, prevention and evidence based medicine guidelines often induce polypharmacy.
Clyne et al. 2016	"Potentially inappropriate or specifically appropriate?" Qualitative evaluation of general practitioners' views on prescribing, polypharmacy and potentially inappropriate prescribing in older people	Ireland	Qualitative (interviews)	• Multiple medications<br>• Focus on Potentially inappropriate prescribing (PIP)	196 older adults (≥70 years) with multimorbidity (≥2 chronic medical conditions)	OPTI-SCRIPT (academic detailing with a pharmacist on conducting GP-led medicines review with participating patients; medicines reviews supported by web-based algorithms for GPs providing alternatives for specific PIP meds identified by the research pharmacist; and tailored patient information leaflets)	• 3 main, inter-related themes emerged (complex prescribing environment, paternalistic doctor-patient relationship, and relevance of PIP concept).<br>• Patient complexity (e.g. polypharmacy, multimorbidity), as well as prescriber complexity (e.g. multiple prescribers, poor communication, restricted autonomy) were all identified as factors contributing to a complex prescribing environment where PIP could occur, as was a paternalistic-doctor patient relationship.<br>• The concept of PIP was perceived to be of variable usefulness to GPs and the criteria to measure it may be at odds with the complex processes of prescribing for this patient population.
Laursen et al. 2018	General Practitioners' Barriers Toward Medication Reviews in Polymedicated Multimorbid Patients: How can a Focus on the Pharmacotherapy in an Outpatient Clinic Support GPs?	Denmar k	Qualitative (interviews)	• concomitant use of ≥2 drugs<br>• often arbitrarily set at cut-off value ≥5	People with polypharmacy and multimorbidity (≥2 chronic diseases)	Interviews with 14 GPs	• The primary barriers toward multimorbid patients with polypharmacy were the need for communication and teamwork with specialists<br>• Often, GPs felt that the specialists were more concerned about following standards and guidelines regarding specific diseases instead of a more holistic patient approach.<br>• To improve management of polypharmacy patients, the GPs suggest that a joint force is

							necessary, a partner-like relationship with greater transparency regarding information transfer, feedback, and shared decision-making.
Lee et al. 2023	Barriers and facilitators to deprescribing before surgery: A qualitative study of providers and older adults.	USA	Qualitative (interviews)	• ≥5 medications	8 older adults (mean 74 years) with polypharmacy (mean 8 medications)	Interviews with 8 primary care physicians in Maryland	• Facilitators and barriers both followed the following themes:<br>• Attitudes towards deprescribing before surgery, perceived benefits of deprescribing before surgery, patient-provider relationship and shared decision-making, hope for surgery, barriers to deprescribing before surgery, and preferences for deprescribing follow-up.
McNamara et al. 2017	Health professional perspectives on the management of multimorbidity and polypharmacy for older patients in Australia	Australi a	Qualitative (interviews)	• Using multiple medications<br>• Focus on appropriate medications management	Older adults (≥65 years) with multimorbidity and polypharmacy	Interviews with a range of 26 health professionals (14 prescribers and 12 non-prescribers, with 5 GPs, 5 hospital professionals, 6 nursing, 6 pharmacy staff)	• Most participants did not routinely use structured approaches to incorporate patients' preferences in clinical decision-making, address conflicting prescriber advice, assess patients' adherence to treatment plans or seek to optimise care plans.<br>• Challenges with coordination and continuity of care, pressures of workload and poorly defined individual responsibilities for care, all contributed to participants' avoiding ownership of multimorbidity management.
Schöpf et al. 2018	Elderly patients' and GPs' perspectives of patient–GP communication concerning polypharmacy: a qualitative interview study	German y	Qualitative (interviews)	• Concurrent prescription of at least 4 or 5 medications<br>• Appropriate medication therapy	6 older adults (≥65 years) along with 3 GPs in a single primary care centre in Germany	Communication about polypharmacy, safety and empowerment	• Patients' awareness of the significance of their active role in addressing polypharmacy needs to be increased.<br>• This includes understanding that trusting the doctor does not preclude asking questions or seeking more information.<br>• GPs might support patients by 'inviting' their contribution.<br>• We need interventions which improve patients' communication skills and address specific issues of polypharmacy, particularly in elderly patients
Sinnott et al. 2015	What to give the patient who has everything? A qualitative study of prescribing for multimorbidity in primary care	Ireland	Qualitative (interviews)	• Appropriate and inappropriate polypharmacy	51 adults (median 75 years, range 39-92 years) with multimorbidity (mean 8.3 conditions)	GP interviews	• Difficulties arose when recommendations or preferences conflicted, to which GPs responded by 'satisficing': accepting care that they deemed satisfactory and sufficient for a particular patient.<br>• Satisficing was manifest as relaxing targets for disease control, negotiating compromise with the patient, or making 'best guesses' about the most appropriate course of action.

							• In patients perceived as stable, GPs preferred to 'maintain the status quo' rather than rationalise medications, even in cases with significant polypharmacy.<br>• Proactive changes in medications were facilitated by continuity of care, sufficient consultation time, and open lines of communication.
Wallis et al. 2017	Swimming Against the Tide: Primary Care Physicians' Views on Deprescribing in Everyday Practice	New Zealand	Qualitative (interviews)	• Multiple medicines<br>• Focus on potentially inappropriate prescribing and deprescribing	People with polypharmacy and multimorbidity (many chronic conditions)	Interviews with 24 GPs	• Deprescribing was "swimming against the tide" of patient expectations, the medical culture of prescribing, and organizational constraints.<br>• It came with inherent risks for both themselves and patients and conveyed a sense of vulnerability in practice.<br>• The only incentive to deprescribing was the duty to do what was right for the patient.<br>• Physicians recommended organizational changes to support safer prescribing, including targeted funding for annual medicines review, computer prompts, improved information flows between prescribers, improved access to expert advice and user-friendly decision support, increased availability of non-pharmaceutical therapies, and enhanced patient engagement in medicines management.
Weir et al. 2021	The role of older patients' goals in GP decision-making about medicines: a qualitative study	Australi a	Qualitative (interviews)	• Taking ≥ 5 medicines<br>• Over-prescribing/ under-prescribing<br>• Inappropriate selection of a medication<br>• Avoidable adverse drug reactions	GPs caring for older patients with polypharmacy	N/A	• Most GPs recognised some value in understanding older patients' goals and preferences regarding their medicines.<br>• GPs differed on the following main themes: 1) definition and perception of patients' goals, 2) relationship with the patient, 3) approach to medicines management and prioritisation.<br>• We observed that GPs preferred one of three different practice patterns in their approach to patients' goals in medicines decisions: 1) goals and preferences considered lower priority – 'Directive'; 2) goals seen as central – 'Goal-oriented'; 3) goals and preferences considered but not explicitly elicited – 'Tacit'.
Zechmann et al. 2019	Barriers and enablers for deprescribing among older,	Switzerl and	Qualitative (interviews)	• > 5 drugs/day<br>• Inappropriate polypharmacy	19 older adults (mean age 76.9 years) with	Interviews	• We identified patient involvement in deprescribing and coordination of care as key issues for deprescribing

	multimorbid patients with polypharmacy: an explorative study from Switzerland			• Focus on benefit and harm ratio, admissions and mortality	multimorbidity taking 8.9 mean drugs per day.		• Conservatism/inertia and fragmented medical care were the main barriers towards deprescribing. No patient felt devalued as a consequence of the deprescribing offer.<br>• GPs concerns regarding patients' devaluation should not prevent them from actively discussing the reduction of drugs.
Anderson et al. 2017	Negotiating "Unmeasurable Harm and Benefit": Perspectives of General Practitioners and Consultant Pharmacists on Deprescribing in the Primary Care Setting	Australi a	Qualitative (focus groups)	• Use of more medicines than are clinically indicated or concurrent use of multiple medicines<br>• no consensus, but ≥5 commonly cited<br>• Focus on potentially inappropriate polypharmacy and deprescribing	Older adults (≥65 years) with polypharmacy who were still residing in the community	Focus group (32 GPs and 15 clinical pharmacists)	• Deprescribing is an inherently uncertain venture<br>• The option to deprescribe is shaped by many factors, including a clinician's perception of the risk/benefit ratio of persisting with the status quo versus deprescribing.<br>• It is more likely to occur in the presence of a continuous therapeutic relationship between the GP and patient and in response to a clear clinical trigger or finding of "low-hanging fruit."<br>• However, poorly developed interprofessional relationships and a lack of dedicated time and tacit knowledge/familiarity with patients, respectively, are important barriers to deprescribing.
Mangin et al. 2019	"I think this medicine actually killed my wife": patient and family perspectives on shared decision-making to optimize medications and safety	Canada	Qualitative (focus groups)	• ≥5 long-term medications<br>• Inappropriate medications<br>• Focus on benefit vs harms	16 adults (55-90 years) with multimorbidity (≥ 2 chronic conditions) from Southern Ontario and British Columbia (convenience sample)	How patient/family preferences and priorities are considered in medication-related discussions and decisions within the healthcare system	• Shared decision-making resulted from both recognition and integration of the personal expertise of the patient and family in medications, and perceived patient-centredness.<br>• It is complex, dynamic, and nonlinear, and patient priorities are not as integrated into shared decision-making about medications as we would hope.<br>• This is broadly consistent with the current conceptualization of evidence-based medicine.<br>• This suggests the need for developing a systematic process to elicit, record, and integrate patient preferences and priorities about medications to create space for a more patient-centred conversation.
Noel et al. 2005	Collaborative care needs and preferences of primary care patients with multimorbidity	USA	Qualitative (focus groups)	• Multiple medications<br>• Medication side effects and burdens	60 patients with ≥2 chronic illnesses (taking 8-27 medications) across 8 primary care clinics within	Any care need or preference for primary care patients with multiple chronic illnesses	• Polypharmacy was a major concern.<br>• Problematic interactions with providers and the health care system were also mentioned, often in relation to specialty care and included incidents in which providers had ignored concerns or provided conflicting advice

					the Veterans Health Administration.		• Knowledge and skills deficits interfered with self-management.<br>• Participants were willing to use technology for monitoring or educational purposes if it did not preclude human contact
Sinnige et al. 2016	Medication management strategy for older people with polypharmacy in general practice: a qualitative study on prescribing behaviour in primary care.	Netherlands	Qualitative (Focus groups)	• Chronic use of ≥5 medications<br>• appropriate polypharmacy	Older adults (aged 68–84 years) diagnosed with multiple, highly prevalent chronic diseases, often part of a cluster of diseases	Focus groups with 12 GPs	• Similar strategy regarding the patients' medication management: defining treatment goals; determining primary goals; and adjusting medications based on the treatment effect, GPs' and patients' preferences, and patient characteristics.<br>• The GPs would like to discuss their choices with other professionals, and they valued structured medication reviews with the patient, as well as quick and practical support tools that work on demand.
Smith et al. 2010	GPs' and pharmacists' experiences of managing multimorbidity: a "Pandora's box"	Ireland	Qualitative (Focus groups)	• Multiple medications use<br>• ≥5 medications	People with multimorbidity	3 focus groups with 13 GPs and 7 pharmacists	• Themes included 1) the concept of multimorbidity and the link to polypharmacy and ageing; 2) health systems issues relating to lack to time, inter-professional communication difficulties, and fragmentation of care; 3) individual issues from clinicians relating to professional roles, clinical uncertainty, and avoidance; 4) patient issues; and 5) potential management solutions.
Uhl et al. 2018	Patient-perceived barriers and facilitators to the implementation of a medication review in primary care: a qualitative thematic analysis.	Germany	Qualitative (focus groups and telephone interviews)	• ≥5 drugs per day<br>• Inappropriate polypharmacy	31 older adults (range 62–88 years), ≥3 chronic diseases, from 17 General practices in Frankfurt (convenience sample)	Medication review	• Barriers to patient participation concerned patient autonomy, while facilitators involved patient awareness of medication-related problems.<br>• Barriers to GP-led reviews concerned GP's lack of resources while facilitators related to the trusting relationship between patient and GP.<br>• Pharmacist-led reviews might be hindered by a lack of patients' confidence in pharmacists' expertise, but facilitated by pharmacies' digital records of the patients' medications.
Reeve et al. 2016	Beliefs and attitudes of older adults and carers about deprescribing of medications: a qualitative focus group study	Australia	Qualitative (focus group)	• Many medicines<br>• Focus on inappropriate medication and deprescribing	14 older adults (mean age 75 years) and 14 carers (mean age 79) across 4 focus groups	Deprescribing	• Main factors: Their perception of the appropriateness of that medication; fear of outcomes of withdrawal; dislike of taking medications; and the availability of a process for withdrawal (including a discussion with a

							healthcare professional and knowing that the medication could be restarted if necessary).<br>• A patient's regular GP was identified as a strong influence both for and against.<br>• A theme unique to the carers was the complexity involved in making decisions about medications for their care recipients.
Bell et al. 2017	Nurses' and pharmacists' learning experiences from participating in interprofessional medication reviews for elderly in primary health care - a qualitative study	Norway	Qualitative (focus group and telephone interviews)	• ≥4 drugs<br>• Inappropriate drug use	Elderly patients with polypharmacy and multimorbidity in primary care	Interprofessional medication reviews	• Although experiencing challenges in conducting IMRs, the nurses and pharmacists had learning experiences they said improved both their own practice and the quality of drug management.<br>• There are some challenges concerning how to ensure participation of all three professions and how to get thorough information about the patient.
Collier et al. 2023	Older people, medication safety, and the role of the community pharmacist: a longitudinal ethnographic study	Australia	Qualitative (ethnography)	• ≥5 regular or 'as required', prescription or non-prescription medications<br>• Focus on medication safety	20 older adults (≥65 years) with frailty and multimorbidity	Community pharmacist involvement	• Community pharmacists play a significant role in the medication safety of older people with frailty and polypharmacy. Analysis resulted in three main themes:<br>1) the older person–determined role of the pharmacist,<br>2) the 'taken for granted' safety work of the pharmacist<br>3) collective agency and medication safety.
Fudge et al 2021	'It's all about patient safety': an ethnographic study of how pharmacy staff construct medicines safety in the context of polypharmacy	UK	Qualitative (ethnography)	• Concurrent use of ≥5 medications<br>• Focus on medication safety	33 pharmacy staff (counter staff, technicians, dispensers, pharmacists)	Community pharmacist involvement	• 'Safety' in the performance of practices relating to polypharmacy was not a fixed, defined notion, but an ongoing, collaborative accomplishment, emerging within an organisational narrative of 'care'.<br>• Despite meticulous attention to 'safety', carefully guarded professional boundaries meant that addressing polypharmacy per se in the context of community pharmacy was beyond reach.
Swinglehurst et al. 2021	Organising polypharmacy: unpacking medicines, unpacking meanings—an ethnographic study	UK	Qualitative (ethnography)	• Multiple medications<br>• ≥10 'higher-risk' polypharmacy<br>• Appropriate and inappropriate polypharmacy	24 older adults (≥65 years) with multimorbidity and ≥10 regular medications from 3 NHS GP Practices	Medication reviews	• Polypharmacy demands careful organising.<br>• All patients had developed strategies and routines for organising medicines into their lives, negotiating medicine taking to enable acceptable adherence and make their medicines manageable.

					(purposive sampling), ethnography also covered 4 community pharmacies		• Strategies adopted by patients often involved the use of 'do-it-yourself' dosette boxes.<br>• Patients incorporated a range of approaches to manage supplies and flex their regimens to align with personal values and priorities. Practices of organising medicines are effortful, creative and often highly collaborative.<br>• Patients strive for adherence, but their organisational efforts privilege 'living with medicines' over taking medicines strictly 'as prescribed'.
Swinglehurst et al. 2023	Negotiating the polypharmacy paradox: a video-reflexive ethnography study of polypharmacy and its practices in primary care	UK	Qualitative (ethnography)	• Multiple medications<br>• ≥10 'higher-risk' polypharmacy<br>• Appropriate and inappropriate polypharmacy	24 older adults (≥65 years) with multimorbidity and ≥10 regular medications from 3 NHS GP Practices (purposive sampling), ethnography also covered 4 community pharmacies	Medication reviews	• Participants rarely referenced biomedical aspects of prescribing (eg, drug-drug interactions, 'Numbers Needed to Treat/Harm') focussing instead on polypharmacy as an emotional and relational challenge. Clinicians initially denigrated their medication review work as mundane. Through VRE they reframed their work as complex, identifying polypharmacy as a delicate matter to negotiate.<br>• Medication review was identified as an ongoing process, rather than a discrete 'one-off' activity. Meaningful progress towards tackling polypharmacy was only possible through small, incremental, carefully supported changes in which both patient and clinician negotiated a sharing of responsibility, best supported by continuity of care.
McIntosh et al. 2018	A case study of polypharmacy management in nine European countries: Implications for change management and implementation.	Spain (authors consortium includes 8 EU countries)	Qualitative (case study)	• ≥5 medications<br>• Appropriate and inappropriate polypharmacy	Older adults (varied but all ≥50 years old), some specific to geriatric departments, or care homes	Varied identified initiatives across 5 sites: Spain (Catalonia), Germany (Lower Saxony), Sweden (Uppsala), and two UK sites (Northern Ireland and Scotland).	• Within the studied EU countries, polypharmacy management was not widely addressed.<br>• Results highlight the importance of change management and theory-based implementation strategies.
All Wales Medicines	Polypharmacy in older people: A guide for	UK (Wales)	Policy	• Use of multiple medications	Adults with polypharmacy	N/A	• Assessing polypharmacy and patient adherence to medication is imperative.

Strategy Group. 2023	healthcare professionals.			• Appropriate and problematic polypharmacy<br>• Particular high risk medications			• To support medicines optimisation in older patients who may be subject to inappropriate polypharmacy, deprescribing high risk medicines is practical
Australian Commission on Safety and Quality in Health Care. 2020	Polypharmacy, 75 years and over	Australia	Policy	≥5 medications at the same time	Older adults (≥75 years) with polypharmacy	N/A	• There are widespread variations in polypharmacy prevalence across the country<br>• Further guidance is needed for communication between medical teams and residential homes, medication management reviews and system changes including establishment, governance and advisory committees.
King's Fund	Polypharmacy and medicines optimisation.	UK	Policy	• Appropriate polypharmacy: prescribing for an individual for complex conditions or for multiple conditions in circumstances where medicines use has been optimised and where the medicines are prescribed according to best evidence.<br>• Problematic polypharmacy: prescribing multiple medications inappropriately, or where the intended benefit of the medication is not realised.	Adults with polypharmacy	N/A	• For many people, appropriate polypharmacy will extend life expectancy and improve quality of life. With meds optimised and prescribed according to best evidence.<br>• Problematic polypharmacy can cause an increased risk of drug interactions and adverse drug reactions, together with impaired adherence and QoL<br>• Many clinical trials and practice guidelines do not consider polypharmacy in the context of multi-morbidity.
Scottish Government Polypharmacy Model of Care Group. 2018	Polypharmacy guidance, realistic prescribing. 3rd Edition.	UK (Scotland)	Policy	• Many medications<br>• Appropriate and inappropriate polypharmacy	Adults with polypharmacy	N/A	• The case for effective polypharmacy management is quite clear, but in a complex healthcare setting with many competing priorities it is useful to outline the quality and economic reasons why it should be prioritised.

							• Need to address polypharmacy management as a public health issue, as multi-morbidities do not just affect the elderly.<br>• Greater emphasis has been placed on shared-decision making to actively engage the patient with the 7-Step medication review.<br>• The Drug Efficacy (NNT) tables help discussion regarding the relative potential benefits of a range of common therapeutic interventions.
The Department of Health and Social Care 2021	National overprescribing review report: Good for you, good for us, good for everybody	UK	Policy	• Overprescribing – the use of a medicine where there is a better non-medicine alternative, or the use is inappropriate for that patients' circumstances and wishes.<br>• Polypharmacy – the concurrent use of multiple medicines for one person. There is currently no consensus on a definition for polypharmacy.<br>• Prevalence calculated at 5+ and 8+.	Adults with polypharmacy	N/A	• Overprescribing is a serious problem in health systems internationally that has grown dramatically over the last 25 years, with systemic and cultural causes.<br>• Estimates at least 10% of medicines overprescribed
McCarthy et al 2020	The evolution of an evidence based intervention designed to improve prescribing and reduce polypharmacy in older people with multimorbidity and significant polypharmacy in primary care (SPPiRE)	Ireland	Pilot and protocol	• Multiple medicines use<br>• Higher levels of polypharmacy ≥ 15 medicines<br>• Focus on potentially inappropriate prescribing (STOPP/START)	10 older adults (≥65 years) taking ≥15 regular medicines	SPPiRE website: educational module template for an individualised patient medication review that identified PIP, deprescribing opportunities, and patient priorities	• A framework was used to systematically describe how and why the original intervention was modified, allowing the new intervention to build upon an effective and robustly developed intervention but also to be relevant in the context of the current evidence base.

McCarthy et al. 2017	Supporting prescribing in older people with multimorbidity and significant polypharmacy in primary care (SPPiRE): a cluster randomised controlled trial protocol and pilot.	Ireland	Pilot and protocol	• Multiple medicines use<br>• Higher levels of polypharmacy ≥ 15 medicines<br>• Focus on potentially inappropriate prescribing (STOPP/START)	10 older adults (≥65 years) taking ≥15 regular medicines	SPPiRE website: educational module template for an individualised patient medication review that identified PIP, deprescribing opportunities, and patient priorities	• The feasibility of the finder tool and intervention were assessed through GP feedback and analysis of use of the online material. Overall, the intervention was well received by the GPs and their patients many of whom reported feeling reassured that their medicines were being reviewed and rationalised.<br>• When the higher prevalence, lower risk proton pump inhibitor PIP was excluded, 90% of patients had at least 1 PIP and the mean number of PIP per patient was 1.1. Identified instances of PIP were acted on in 44% of cases and 45% when the proton pump inhibitor PIP was excluded.<br>• The most common patient priorities were treating pain, followed by fatigue, and reducing the number of repeat medicines.
Kirwan et al. 2022	The multimorbidity collaborative medication review and decision making (MyComrade) study: a pilot cluster randomised trial in two healthcare systems	UK and Ireland	Pilot (Cluster RCT)	• ≥10 medications	121 adults (≥18 years and over, with multi morbidity and ≥10 medications living in the community across 15 GP practices in Northern Ireland and Ireland	Training (Face-to-face or pre-recorded) and inclusion of practice-based pharmacists in NI as collaborative reviewers using the NO TEARS tool to encourage shared decision making	• Both practice staff and patients found the intervention acceptable and reported strong fidelity to the My Comrade intervention components.<br>• Some staff highlighted concerns such as poor communication of the reviews to patients, dissatisfaction regarding incentivisation and in ROI the sustainability of two GPs collaboratively conducting the medication reviews.<br>• Pairing of GP and pharmacist may be more sustainable to implement in routine practice.
Rankin et al. 2022	An external pilot cluster randomised controlled trial of a theory-based intervention to improve appropriate polypharmacy in older people in primary care (PolyPrime).	UK and Ireland	Pilot (Cluster RCT)	• ≥ 5 medicines<br>• ≥ 4 medications for ≥3 months<br>• Focus on potentially inappropriate prescribing (STOPP/START and NO TEARS)	120 older adults (≥ 70 years old) prescribed ≥4 medications for ≥3 months living in the community across 12 GP practices in Northern Ireland and Ireland	An online video (incorporating behaviour change) and scheduled medication reviews with patients on 2 occasions.	• The intervention was successfully delivered as intended; it was acceptable to GPs, practice staff, and patients; and potential mechanisms of action have been identified.<br>• It may be feasible to conduct an intervention to improve appropriate polypharmacy in older people in primary care across two healthcare jurisdictions.
Cardwell et al. 2020	Evaluation of the General Practice Pharmacist (GPP) intervention to	Ireland	Pilot	• Many medications<br>• Focus on potentially inappropriate prescribing, high risk	786 older adults (mean age 69.8 years) from 4 General practices	A pharmacist joined the practice team for 6 months and	An intervention involving pharmacists, working within general practices is feasible to implement and has potential to improve prescribing quality.

	optimise prescribing in Irish primary care: a non-randomised pilot study.			medications and deprescribing		undertook medication reviews (face to face or chart based), provided prescribing advice and facilitated practice-based education	
Michiels-Corsten et al. 2022	MediQuit – an electronic deprescribing tool: a pilot study in German primary care; GPs' and patients' perspectives	Germany	Pilot	• Multiple medications<br>• ≥ 5 drugs<br>• Excessive polypharmacy = ≥ 10 drugs<br>• Inappropriate polypharmacy<br>• Focus on overprescribing and deprescribing	41 older adults (mean age 77 years) with at least 3 chronic diseases (mean was 8) with ≥ 10 drugs in 2 regions of Germany	MediQuit - Electronic Deprescribing Tool (3 steps: 1. Med review with algorithm to flag PIM for deprescribing. 2. Communcation prompts, and risk-benefits. 3. Information on discontinuation process).	• Identification (step 1) and implementation elements (Step 3) were perceived most helpful by GPs. Whereas, shared-decision making elements (step 2) revealed room for improvement.<br>• Patients were broadly satisfied with the deprescribing consultation (85%) and with their decision made regarding their medication (90%).<br>• GPs were satisfied tool and gave important hints for future development.
Benson et al. 2021	Medication management for complex patients in primary care: application of a remote, asynchronous clinical pharmacist model	USA	Feasibility	• ≥ 5 medicines<br>• Focus on drug related problems (Indication, Effectiveness, Safety, and Compliance)	202 adults (aged 40+) with multimorbidity (≥2 chronic conditions) and high health expenditure	A remotely delivered Comprehensive Medication Management allowing pharmacists to make prescription changes (for 6 months)	• A clinical pharmacist found that 86% of participants had a drug therapy problem according to classification criteria. Seventy-nine percent of all drug therapy problems identified were resolved upon completion of the study.<br>• A service model using remote pharmacist services may be an effective means of improving team-based primary care medication management for this population.
Junius-Walker et al. 2021	MediQuit, an Electronic Deprescribing Tool for Patients on Polypharmacy: Results of a Feasibility Study in German General Practice	Germany	Feasibility	• Multiple medications<br>• ≥ 5 drugs<br>• Excessive polypharmacy = ≥ 10 drugs<br>• Inappropriate polypharmacy	41 older adults (mean age 77 years) with at least 3 chronic diseases (mean was 8) with ≥ 10 drugs in 2 regions of Germany	MediQuit - Electronic Deprescribing Tool (3 steps: 1. Med review with algorithm to flag PIM for deprescribing. 2.	• Deprescribing was achieved in 70% of consultations in agreement with patients.<br>• Drugs deprescribed were symptom-lowering and preventive drugs<br>• GPs found MediQuit useful in initiating communication on this issue and enhancing deliberations for a deprescribing decision.

				• Focus on overprescribing and deprescribing		Communication prompts, and risk-benefits. 3. Information on discontinuation process).	• GPs rated patient involvement higher than did patients themselves.
Mangin et al. 2023	Team approach to polypharmacy evaluation and reduction: feasibility randomized trial of a structured clinical pathway to reduce polypharmacy.	Canada	Feasibility	• ≥5 long-term medications<br>• Focus on appropriateness and patient preferences.	37 older adults (≥ 70 years) on ≥ 5 long-term medications	TAPER – a team based clinical pathway for a complete medication review by the pharmacist and the physician aimed at reducing medication burden	• Results from this feasibility study indicate that TAPER as a clinical pathway is feasible to implement in a primary care team setting and in an RCT research framework.<br>• Outcome trends suggest effectiveness.
Cahir et al. 2014	Potentially inappropriate prescribing and vulnerability and hospitalization in older community-dwelling patients	Ireland	Longitudinal study (retrospective cohort)	• Multiple medications<br>• Focus on potentially inappropriate prescribing (compares Beers and STOPP criteria)	931 community-dwelling patients aged ≥70 years in 15 general practices in Ireland	N/A	• The prevalence by the Beers 2012 and STOPP criteria was 28% (n = 246) and 42% (n = 377), respectively. Patients with ≥2 PIP indicators were almost twice as likely to be classified as vulnerable.<br>• STOPP is a more sensitive measure of PIP than the Beers 2012 criteria and of clinical benefit in primary care settings, particularly for hospital visits.
Doherty et al. 2023	Adverse drug reactions and associated patient characteristics in older community-dwelling adults: a 6-year prospective cohort study	Ireland	Longitudinal study (prospective cohort)	• ≥5 regular prescribed medications<br>• major polypharmacy (≥10 drug classes)	592 older adults (aged ≥ 70 years) across 15 practices in Ireland	N/A	• A total of 211 ADRs were recorded for 159 participants, resulting in a cumulative incidence of 26.9% over 6 years.<br>• The majority of ADRs detected were mild (89.1%), with the remainder classified as moderate (10.9%). Eight moderate ADRs, representing 34.8% of moderate ADRs and 3.8% of all ADRs, required an emergency hospital admission.<br>• ADRs were independently associated with female sex, polypharmacy (5–9 drug classes) and major polypharmacy (≥10 drug classes)
McCarthy et al. 2023	Medication changes and potentially inappropriate prescribing in older	Ireland	Longitudinal study (secondary analysis of	• Multiple medicines use<br>• Focus on potentially inappropriate	404 older adults (aged ≥ 65 years), prescribed ≥ 15 repeat medicines,	N/A	• There were reductions in the prescription of most drug groups with the largest reduction in antiplatelet prescriptions.

	patients with significant polypharmacy.		RCT at 2 time points)	prescriptions (SPPiRE PIP criteria)	from 51 different general practices		• Considering medication discontinuations, initiations and switches, there was a median of 5 medication changes per person<br>• A high proportion of benzodiazepine, anticholinergic and diuretic prescriptions were potentially inappropriate suggesting these higher risk groups may warrant specific attention.
O'Regan et al. 2023	How often do patients attend general practice, how often are they referred to hospital, and how do multi-morbidity and polypharmacy affect general practice attendance and referral rates?	Ireland	Longitudinal study (retrospective cohort)	• ≥5 regular medications	6603 older adults (50+ years) from 72 GP practices	N/A	• Increasing age, number of chronic illnesses and number of medications were associated with increased attendance rates to the GP and practice nurse and home visits but did not significantly increase the ratio of attendance to referral rate.<br>• General practice must be supported to provide person centred care to an ageing population with rising rates of multi-morbidity and polypharmacy.
Tampaki et al. 2023	Inappropriate prescribing in geriatric rural primary care: impact on adverse outcomes and relevant risk factors in a prospective observational cohort study.	Greece	Longitudinal study (prospective cohort)	• Use of potentially inappropriate medication (PIMs) or potential prescribing omissions (PPOs). [using STOPP/START]	104 older adults (≥ 65 years, median age 78 years) receiving a median of 6 drugs	N/A	• PPO was found in 78% and PIMs in 61%. PIM was multivariately correlated with multimorbidity (p = 0.029) and polypharmacy (p < 0,001), while drug-PPO was only associated with multimorbidity (p = 0.039).<br>• The number of PIM predicted emergency department visits and hospitalizations at 6-month follow-up (p value 0.011), independent of age, sex, frailty, comorbidities, and total medication number.
Muller et al. 2020	Development and internal validation of prognostic models to predict negative health outcomes in older patients with multimorbidity and polypharmacy in general practice.	Germany	Longitudinal study (prognostic modelling)	• Multiple medicines use<br>• Appropriate & inappropriate polypharmacy<br>• Focus on Quality of life	592 958 older adults (≥60 years, ≥5 drugs, ≥3 chronic diseases, excluding dementia) in Germany	N/A	• Best trial data-based model predicted HRQoL after 6 months well and included parameters of well-being not found in claims.<br>• Performance of claims data-based models and models predicting long-term outcomes was relatively weak.
Kruger et al. 2021	Non-random relations in drug use expressed as patterns comprising prescription and over-the-counter drugs in	Germany	Longitudinal study (retrospective cohort study)	Chronic co-prescription or co-application of different drugs at the same time	3189 older adults (65-85 years) with at least 3 chronic diseases	N/A	There are strong associations between drug patterns and multimorbidity clusters, which enrich the knowledge about the treatment of multimorbid elderly patients in primary care.

	multimorbid elderly patients in primary care: Data of the exploratory analysis of the multicentre, observational cohort study MultiCare.						
Carr et al 2021	A multidimensional measure of polypharmacy for older adults using the Health and Retirement Study	UK	Longitudinal study	• Concurrent use of multiple medications by one individual<br>• Added dimensions e.g. temporality, dosage, appropriateness, anticholinergic properties, potential drug interactions<br>• Cut offs of 5+ and 9+ used in comparative analyses	2141 adults (aged 50-80) living in the USA	N/A	• A four-class model was selected based on fit statistics and clinical interpretability.<br>• Participants in the 'low risk' class tended to be male, cohabitating, and reported fewer health conditions, compared to 'high risk' classes.<br>• The three 'high risk' classes overlapped with the groups concurrently taking 5+ and 9+ medications per month.
D'Aiuto et al. 2023	Health care system costs related to potentially inappropriate medication use involving opioids in older adults in Canada	Canada	Longitudinal study	• Potentially inappropriate medication (PIMs) – Beer's criteria<br>• Potentially inappropriate medication use involving opioids (PIOU)	1201 older adults (≥ 65 years)	N/A	• Potentially inappropriate medication use involving opioids is associated with higher costs compared to those observed with opioid use and no use.<br>• There is a need for more effective use of health care resources to reduce costs for the health care system.
Fahmi et al. 2023	Combinations of medicines in patients with polypharmacy aged 65-100 in primary care: Large variability in risks of adverse drug related and emergency hospital admissions.	UK	Longitudinal study	• Use of multiple medicines<br>• Use of ≥5 drugs (within 84 days prior)	89,235 older adults (≥ 65 years)	N/A	• There were over 112,000 different combinations of the 50 medicine classes most implicated in ADR-related hospital admission in the RF models, with the most important medicine classes being loop diuretics, domperidone and/or metoclopramide, medicines, and sulfonamides and/or trimethoprim.<br>• Polypharmacy involves a very large number of different combinations of medicines, with substantial differences in risks of ADR-related and emergency hospital admissions.<br>• Simple tools based on few medicine classes may not be effective in identifying high risk patients.

Häppölä et al. 2020	A data-driven medication score predicts 10-year mortality among aging adults.	Finland	Longitudinal study	Use of numerous potentially interacting medications.	20,078 adults (aged 46-74) in community sites across Finland (mainly North)	N/A	• The resulting score is strongly associated with all-cause mortality (HR 1.18 per point increase in score; 95% CI 1.14–1.22)<br>• When combined with Charlson comorbidity index, individuals had over 6x risk (HR 6.30; 95% CI 3.84–10.3) compared to individuals with a protective score profile.<br>• Alone, the medication score performs similarly to the Charlson comorbidity index
Monterde et al. 2020	Multimorbidity as a predictor of health service utilization in primary care: a registry-based study of the Catalan population.	Spain	Longitudinal study	Prescription of more than 8 drugs over one year	6,102,595 adults (≥18 years) in Catalonia	N/A	• Multimorbidity assessment enhanced prediction of use of healthcare resources at community level<br>• Clinical risk groups had a higher predictive performance for polypharmacy over Charlson Index and Adjusted Morbidity Groups
Villén et al. 2020	Multimorbidity patterns, polypharmacy and their association with liver and kidney abnormalities in people over 65 years of age: a longitudinal study.	Spain	Longitudinal study	concomitant consumption of ≥5 medications	743,827 older adults (65–99 years) in 285 primary health care centres (PHCCs) in Catalonia	N/A	• The most frequently prescribed medicines were related to multimorbidity patterns and their consumption was maintained throughout the follow-up period.<br>• A higher risk of abnormal kidney and liver function was observed in specific multimorbidity patterns.
Guthrie et al. 2015	The rising tide of polypharmacy and drug-drug interactions: population database analysis 1995-2010	UK (Scotland)	Longitudinal analysis (retrospective cohort)	• concomitant prescription of ≥5 or ≥10 drugs<br>• Major or excessive polypharmacy<br>• 3 levels of analysis for polypharmacy as ≥5, ≥10, and ≥15 drugs dispensed in the previous 84 days.<br>• Focus on drug-drug interactions	310,000 adults (≥20 years, mean 48 years) in one region of Scotland	N/A	• Between 1995 and 2010, the proportion of adults dispensed ≥5 drugs doubled to 20.8%, and the proportion dispensed ≥10 tripled to 5.8%.<br>• Receipt of ≥10 drugs was strongly associated with increasing age but was also in people living in more deprived areas or in a care home<br>• The proportion with potentially serious drug-drug interactions more than doubled to 13% of adults in 2010, and the number of drugs was most strongly associated with this
Payne et al. 2014	Is polypharmacy always hazardous? A retrospective cohort analysis using linked electronic health records from primary and secondary care	UK	Longitudinal analysis (retrospective cohort)	• Prescription of multiple medications<br>• Focus on appropriate vs inappropriate polypharmacy	180 815 adults with long-term clinical conditions and numbers of regular medications	N/A	• Admissions were more common in patients on multiple medications, but admission risk varied with the number of conditions.<br>• Unplanned hospitalization is strongly associated with the number of regular medications. However, the effect is reduced in patients with multiple conditions, in whom only the most

							extreme levels of polypharmacy are associated with increased admissions.<br>• Assumptions that polypharmacy is always hazardous and represents poor care should be tempered by clinical assessment of the conditions for which those drugs are being prescribed.
von Buedingen et al. 2018	Changes in prescribed medicines in older patients with multimorbidity and polypharmacy in general practice	German y	Longitudinal analysis (secondary analysis of RCT data)	• Regular use of five or more drugs<br>• Inappropriate prescriptions	505 older adults (median age 72 years) with multimorbidity (≥3 chronic conditions) across 72 general practices in Hesse, Germany.	PRIMUM (The healthcare assistant conducted a checklist-based interview with patients on medication-related problems and reconciled their medications. Then, using a computerised decision support system, the GP optimised medication, with patients)	• Medication regimens in older patients with multimorbidity and polypharmacy changed frequently.<br>• These are mostly due to discontinuations and dosage alterations, followed by additions and restarts.<br>• These findings cast doubt on the effectiveness of cross-sectional assessments of medication and support longitudinal assessments where possible.
Alaa Eddine et al. 2020	A pharmacist-led medication review service with a deprescribing focus guided by implementation science	Lebano n	Longitudinal analysis	• ≥ 5 medicines<br>• Drug related problems and potentially inappropriate prescribing<br>• Focus on deprescribing	143 older adults (≥ 65 years) at a single site (mixed GP/physicians in primary care facility) in Lebanon for low-income patients (receiving free medication)	An pharmacist-led medication review, with recommendation s placed with GPs/ physicians at the facility.	• The intervention pharmacist provided 221 recommendations to physicians, of which 52% were to discontinue one or more medications.<br>• Patients in the intervention group showed significantly higher satisfaction compared to the ones in the control group ($p < 0.001$, effect size = 1.75).<br>• Of all recommendations, 30% were accepted by the physicians.
Vos et al. 2022	Fifteen-year trajectories of multimorbidity and polypharmacy in Dutch primary care—A longitudinal analysis of age and sex patterns.	Netherl ands	Longitudinal analysis	≥5 or more different medications in one year	10,037 patients, all ages from GP practices in South Netherlands	N/A	• Multimorbidity and polypharmacy are common, and their prevalence is accelerating, with a relatively rapid increase in younger groups.<br>• This underlines the need for a longitudinal approach and a life course perspective in patient care.

Novella et al. 2022	Relation between drug therapy-based comorbidity indices, Charlson's comorbidity index, polypharmacy and mortality in three samples of older adults.	Italy	Longitudinal analysis (retrospective cohort)	• Polypharmacy: ≥5 medications<br>• Excessive polypharmacy: ≥10 medications<br>• This definition included systemic and topical drugs	Older adults (all 65+ years) including 2,389 nursing home residents, 4,765 and 633 older adults admitted acutely to geriatric or internal medicine wards	N/A	On the whole, comorbidity indices did not perform well in our three settings, although the highest level of each index was associated with higher mortality.
McCarthy et al. 2022	Patient and general practitioner experiences of implementing a medication review intervention in older people with multimorbidity: Process evaluation of the SPPiRE trial	Ireland	Evaluation (RCT)	• Multiple medicines use<br>• Higher levels of polypharmacy ≥ 15 medicines<br>• Focus on potentially inappropriate prescribing (STOPP/START)	404 older adults (≥65 years) taking ≥15 regular medicines from 51 GP practices	SPPiRE website: educational module template for an individualised patient medication review that identified PIP, deprescribing opportunities, and patient priorities	• The SPPiRE intervention had a small effect in reducing the number of medicines and this was primarily mediated through the brown bag review.<br>• The context of resource shortages and deep-seated views around medical decision-making influenced intervention implementation.<br>• Intervention delivery varied among practices and 45 patients (28%) had no review, primarily due to insufficient GP time. GPs and patients responded positively to the intervention but most GPs did not engage with the patient priority-setting process.<br>• GPs identified a lack of integration into practice software and resources as barriers to future implementation.
Jager et al. 2017	A tailored programme to implement recommendations for multimorbid patients with polypharmacy in primary care practices-process evaluation of a cluster randomized trial	Germany	Evaluation (RCT)	• ≥4 drugs	273 adults (>50 years) suffering from at least 3 chronic diseases, receiving more than 4 drugs, and being at high risk for medication-related events according to the assessment of the treating GP	The tailored programme consisted of a workshop for practice teams, elaboration of implementation action plans, aids for medication reviews, a multilingual info-tool for patients on a tablet PC, posters and	• The most frequently reported effect of the tailored programme was the increase of awareness for the health problem and the recommendations, while implementation of routine processes was only reported for structured medication counselling.<br>• Several modifications of the tailored programme may enhance its effectiveness such as conducting outreach visits instead of a workshop, improved targeting, provision of evidence, integration of tools into the practice software and information materials in tailored formats.

						brown paper bags as reminders for patients.	
Akyon et al. 2023	Artificial intelligence-supported web application design and development for reducing polypharmacy side effects and supporting rational drug use in geriatric patients	Turkiye	Cross-sectional study	• simultaneous use of ≥5 drugs<br>• Focus on potentially inappropriate medications	296 older adults (≥ 65 years)	AI supported web application - auxiliary reference tool for drug–drug, and drug-disease interactions	• While the PIM coverage rate with the proposed tool was 75.3%, the PIM coverage rate of EU(7)-PIM, US-FORTA, TIME-to-STOPP, Beers 2019, STOPP, Priscus criteria in the web application database respectively(63.5%–19.5%) from the highest to the lowest.<br>• PIM criteria alone are insufficient to include actively used medicines and it shows heterogeneity.
Aubert et al. 2016	Polypharmacy and specific comorbidities in university primary care settings.	Switzerland	Cross-sectional study	• ≥ 5 long-term prescribed drugs<br>• potentially inappropriate prescribing and potential prescribing omission (using STOPP/START)	1002 patients aged 50–80 years followed in Swiss university primary care settings	N/A	Polypharmacy is common in university primary care settings, is strongly associated with hypertension, diabetes mellitus, chronic kidney disease and cardiovascular diseases, and increases potentially inappropriate prescribing.
Barrio-Cortes et al. 2023	Differences in healthcare service utilization in patients with polypharmacy according to their risk level by adjusted morbidity groups: a population-based cross-sectional study.	Spain	Cross-sectional study	• ≥ 6 medicines	1598 adults (mean age 82.7)	N/A	• Polypharmacy population compared to non-polypharmacy was characterized by a more advanced age, predominance of women, high-risk, complexity, numerous comorbidities, dependency and remarkable healthcare utilization.<br>• Factors associated with a greater primary care utilization in patients with polypharmacy were elevated complexity, high risk level and dysrhythmia.
Bužančić et al. 2023	Deprescribing in a multimorbid older adult: A case vignette study among community pharmacists and primary care physicians.	Croatia	Cross-sectional study	• ≥5 regular medications concomitantly<br>• ≥10 medications (Hyperpolypharmacy)<br>• Focus on deprescribing	Older adult with taking 16 medications	Case vignette (comparing pharmacist and physician deprescribing acceptance)	• Physicians would accept rationales to deprescribe a median of 10 medicines, while pharmacist recommend a median of six medicines.<br>• Most difference lays in deprescribing of preventative medicines.<br>• Action is needed to improve pharmacists' skills in recognizing deprescribing targets and confidence in making suggestions, which could lead to opening of possibilities for joint patient care.

Calderon-Larranaga et al. 2013	Polypharmacy patterns: unravelling systematic associations between prescribed medications	Spain	Cross-sectional study	• Multiple medicines<br>• Focus on polypharmacy patterns or clusters	79,089 people (≥15 years, mean 47 years)	N/A	• 7 patterns of polypharmacy were identified, which may be classified depending on the type of disease they are intended to treat: cardiovascular, depression-anxiety, acute respiratory infection, chronic obstructive pulmonary disease, rhinitis-asthma, pain, and menopause.<br>• Almost all of the patterns included drugs for preventing or treating potential side effects of other drugs in the same pattern.<br>• It demonstrated the existence of non-random associations in drug prescriptions.
Guisado-Clavero et al. 2019	Medication patterns in older adults with multimorbidity: a cluster analysis of primary care patients.	Spain	Cross-sectional study	Multiple drug use (examined in categories of 1-4, 5-9, and 10+ medications)	164,513 older adults (64-94 years) with multimorbidity (≥2 chronic diseases) within 50 primary healthcare centres in Barcelona	N/A	• Six medication patterns were identified, 5 of which were related to one or more anatomical group, with associations among drugs from different systems.<br>• Overall, guidelines do not accurately reflect the situation of the elderly multimorbid, new strategies for managing multiple drug uses are needed to optimize prescribing in these patients.
Kardas et al. 2023	Optimizing polypharmacy management in the elderly: a comprehensive European benchmarking survey and the development of an innovative online benchmarking application.	Poland, UK and Qatar (Study across all EU)	Cross-sectional study	• Multiple medications<br>• ≥5 medications	Older adults (≥ 65 years)	911 professionals (mostly pharmacists, GPs, consultants nurses and other stakeholders) from all but 2 EU countries	• Out of the survey participants, 496 (54.4%) reported availability of various activities or formal programs targeting polypharmacy in the elderly that were known to them.<br>• These programs had multiple goals, of which improved patient safety was indicated as the most common objective (65.1% of the cases).<br>• The most typical settings for such programs was primary care (49.4%), with pharmacists and primary care doctors being indicated most often as those providing the programs (61.7% and 35.5% of cases, respectively).<br>• Vast majority of programs applied diverse forms of drug reviews.
King et al. 2023	Potentially Inappropriate Medication Use among Underserved Older Latino Adults.	USA	Cross-sectional study	• ≥5 medications<br>• Focus on potentially inappropriate medication (PIM) – Beer's criteria	126 older adults (≥ 65 years) living in 14 mostly Latino senior	N/A	• One-third of participants had at least one PIM.<br>• Polypharmacy (≥5 medications) was observed in 55% of our sample. In addition, 46% took drugs to be used with caution (UWC).

					centers and housing units		• In total, 16% were taking between 9 and 24 medications, whereas 39% and 46% were taking 5 to 8 and 1 to 4 prescription medications.<br>• The multinomial logit regression analysis showed that (controlling for demographic variables) increased PIM use was associated with an increased number of prescription medications, number of chronic conditions, sleep difficulty, lack of access to primary care, financial strains, and poor self-rated health.
Lopez-Rodriguez et al. 2015	Potentially inappropriate prescriptions according to explicit and implicit criteria in patients with multimorbidity and polypharmacy. MULTIPAP: A cross-sectional study	Spain	Cross-sectional study	• Simultaneous use of several medicines.<br>• Potentially inappropriate medications (using both explicit and implicit criteria)	593 community-dwelling elderly aged 65 to 74 years, with multimorbidity and polypharmacy	N/A	• Potentially inappropriate prescribing was detected in 57.7%, 43.6%, 68.8% and 71% of 50 patients according to the explicit criteria STOPP 2014, STOPP 2008, Beers 2019 and Beers 2015 respectively.<br>• The MAI criteria detected greater inappropriateness than did the explicit criteria, but their application was more complex and difficult to automate.<br>• For every new drug taken by a patient, the MAI score increased by 2.41 (95% CI 1.46; 3.35) points.<br>• Diabetes, ischaemic heart disease and asthma were independently associated with lower summated MAI scores.
Lozano-Hernandez et al. 2020	Social support, social context and nonadherence to treatment in young senior patients with multimorbidity and polypharmacy followed-up in primary care. MULTIPAP Study	Spain	Cross-sectional study	• Simultaneous consumption of ≥ 5 drugs<br>• Potentially inappropriate medication<br>• Focus on adherence	593 older adults (between 65-74 years) with multimorbidity (≥3 diseases) and polypharmacy (≥5 drugs) during the last three months	MULTIPAP intervention: GPs e-training and GP-patient-centred interview.	• Among patients 65-74 years of age with multimorbidity and polypharmacy, lower functional support was related to nonadherence to treatment<br>• The nonadherence decreased in those patients with higher functional support, lower urban vulnerability, and higher perceived health status according to the visual analog scale of health-related quality of life
Manirajan et al. 2023	Drug Utilisation Review among Geriatric Patients with Noncommunicable Diseases in a Primary Care Setting in Malaysia.	Malaysi a	Cross-sectional study	• ≥5 more medications<br>• ≥10 = excessive medications	310 older adults (≥ 65 years) in one primary care clinic in Malaysia	N/A	• Combination therapy was prescribed to more than 97% (n = 302) of the elderly, whereas cardiovascular and endocrine medications were the most common.<br>• 10 prescriptions were found to have drug-related problems, prescribing cascade (80%), lack of medicine optimisation (10%), and inappropriate prescription (10%).

Moreira et al. 2020	Polypharmacy among adult and older adult users of primary care services delivered through the Unified Health System in Minas Gerais, Brazil.	Brazil	Cross-sectional study	≥5 medications	1,159 interviewees distributed across 104 cities and 253 primary healthcare services	N/A	Understanding the medication use profile in primary health care allows identifying groups of people that are more likely to experience polypharmacy, and planning measures to mitigate potential polypharmacy-related problems.
Neuner-Jehle et al. 2017	Patient–provider concordance in the perception of illness and disease: a cross-sectional study among multimorbid patients and their general practitioners in Switzerland	Switzerland	Cross-sectional study	• Multiple medications<br>• Focus on concordance, complexity and patient-centeredness	334 older adults (≥60 years, mean 76.2) with multimorbidity across 46 GPs in North Switzerland	N/A	• A majority of GPs perceive the CCs of the multimorbid patients correctly, but there is room for improvement.<br>• Concordance between CCs and diagnosis was 53.6%.<br>• The younger age and higher intake of drugs were significantly associated with an increased concordance between CCs and diagnosis.
Ong et al. 2018	Variation of polypharmacy in older primary care attenders occurs at prescriber level	Malaysia	Cross-sectional study	• Concomitant use of≥ 5 medications<br>• Inappropriate medication use	22,832 Older adults (≥65 years) attending 2914 primary care clinics in Malaysia (nationwide)	N/A	• A total of 20.3% of the older primary care attenders experienced polypharmacy (26.7%% in public and 11.0% in private practice).<br>• The adjusted odds ratio (OR) of polypharmacy were 6.37 times greater in public practices. Polypharmacy was associated with patients of female gender (OR 1.49), primary education level (OR 1.61) and multimorbidity (OR 14.21).<br>• The variation in rate of polypharmacy was mainly found at prescriber level.
Ose et al. 2012	Let's talk about medication: concordance in rating medication adherence among multimorbid patients and their general practitioners	Germany	Cross-sectional study	• Multiple medications use<br>• Focus on adherence (self-rated)	92 patients with multiple chronic conditions at high-risk of hospitalisation (subjective) across 10 primary care practices from a region in Germany	N/A	• The percentage of concordance ranges between 40% (forgot to take medication) and 61% (deliberately omitted a dose).<br>• Talking about medication on a regular basis and better continuity of care may enhance patient – provider concordance in rating medication adherence as a prerequisite for shared decisions concerning medication in patients with multiple chronic conditions.
Rieckert et al. 2018	Polypharmacy in older patients with chronic diseases: a cross-sectional analysis of	Austria, Germany, Italy, and UK	Cross-sectional study	excessive polypharmacy (taking ≥10 substances daily).	3904 older adults (aged 75+)	N/A	Frailty, multimorbidity, obesity, and decreased physical as well as mental health status are risk factors for excessive polypharmacy. Sex,

	factors associated with excessive polypharmacy.						educational level, and smoking apparently do not seem to be related to excessive polypharmacy.
Rogero-Blanco et al. 2020	Use of an Electronic Clinical Decision Support System in Primary Care to Assess Inappropriate Polypharmacy in Young Seniors With Multimorbidity: Observational, Descriptive, Cross-Sectional Study	Spain	Cross-sectional study	Focus on potentially inappropriate medications (Beers and STOPP/START)	593 older adults (aged 65-75) with multimorbidity (≥3 diseases) and polypharmacy (≥5 medications)	N/A	• There is a high prevalence of potentially inappropriate medications<br>• Females, taking a greater number of medicines, working in the primary sector, and being prescribed drugs for the central nervous system were related to a higher frequency<br>• The most common potentially inappropriate medications included benzodiazepines and prolonged use of proton pump inhibitors
Rogero-Blanco et al. 2021	Drug interactions detected by a computer-assisted prescription system in primary care patients in Spain: MULTIPAP study	Spain	Cross-sectional study	• Simultaneous consumption of ≥ 5 drugs<br>• Potentially inappropriate medication<br>• Focus on drug-drug interactions	593 older adults (between 65-74 years) with multimorbidity (≥3 diseases) and polypharmacy (≥5 drugs) during the last three months	MULTIPAP intervention: GPs e-training and GP-patient-centred interview.	• Half of the patients had at least one relevant drug interaction<br>• Factors associated with drug interactions were the use of more than 10 drugs (OR 11.86) and having anxiety/depressive disorder (OR 1.98), with protective factors against as hypertension, diabetes, and ischaemic heart disease
Sirois et al. 2019	The delicate choice of optimal basic therapy for multimorbid older adults: A cross-sectional survey.	Canada	Cross-sectional study	• Many medications<br>• ≥10 medications	Older adults (65–75 years) with type 2 diabetes, chronic obstructive pulmonary disease and heart failure	N/A	• At least half of the participants (pharmacists and geriatricians) considered polypharmacy (≥10 medications) inevitable for an optimal basic treatment of DM, COPD and HF.<br>• The heterogeneity of responses raises issues when considering quality indicators in population-based studies.
Troncoso-Marino et al. 2021	Medication-related problems in older people in Catalonia: A real-world data study.	Spain	Cross-sectional study	Focus on medication-related problems<br>• Duplicate therapy<br>• Drug-drug interactions<br>• Potentially inappropriate medications (Beers, STOPP/START, PRISCUS)	853 085 older adults (aged 65-99) with multimorbidity in 284 primary health care centres in Catalonia	N/A	• The coexistence of multimorbidity and polypharmacy is associated with an elevated risk of medication-related problems<br>• The most common potentially inappropriate drugs were those that increase the risk of fall (66.8%), antiulcer agents without criteria for gastroprotection (40.6%), and the combination of drugs with anticholinergic effects (39.7%).

				• Drugs contraindicated in chronic kidney disease and in liver diseases			
Carrier et al. 2019	GPs' management of polypharmacy and therapeutic dilemma in patients with multimorbidity: a cross-sectional survey of GPs in France	France	Cross-sectional study	• Many drugs at the same time or an excessive number<br>• No consensus on threshold or temporality<br>• Focus on unfavourable risk–benefit and potentially inappropriate prescribing	Older adults (54-82 years) with multimorbidity	Survey amongst 1266 GPs	• Nearly all (91.4%) responders felt comfortable or fairly comfortable deprescribing inappropriate medications, but only 34.7% decided to do so often or very often<br>• In therapeutic dilemmas, some GPs choose to prioritise patients' requests over iatrogenic risks.<br>• GPs need pragmatic implementation tools for handling therapeutic dilemmas, and to improve their skills in medication management and patient engagement in such situations.
Cooper et al. 2016	Potentially inappropriate prescribing in two populations with differing socio-economic profiles: a cross-sectional database study using the PROMPT criteria.	UK (NI), Ireland	Cross-sectional study	• ≥4 repeat medicines<br>• Focus on Potentially inappropriate prescribing (PROMPT)	>750,000 middle-aged adults (45–64 years) in 2 populations with differing socio-economic profiles in Ireland	N/A	• Age group, female gender and polypharmacy were significantly associated with PIP in both populations and polypharmacy had the strongest association.<br>• PIP is common amongst middle-aged people with the risk of PIP increasing with polypharmacy.<br>• Differences in the prevalence of polypharmacy and PIP between the two populations may relate to heterogeneity in healthcare services and different socio-economic profiles, with higher rates of multimorbidity and associated polypharmacy in more deprived groups.
Gutierrez-Valencia et al. 2019	Prevalence of polypharmacy and associated factors in older adults in Spain: Data from the National Health Survey 2017	Spain	Cross-sectional study	• polypharmacy (≥ 5 medications)<br>• hyperpolypharmacy (≥10)	7023 older adults (aged 65+) from National Health Survey of Spain	N/A	• The prevalence of polypharmacy in the elderly in primary care continues to increase and could be widely underestimated.<br>• Factors such as functional capacity or geriatric syndromes, fundamental in elderly people, modulate the habits of consumption and prescription of drugs in this population.
Payne et al 2014	Prevalence of polypharmacy in a Scottish primary care population	UK (Scotland)	Cross-sectional	• multiple medications by a single patient<br>•	180,815 adult (≥20 years) patients permanently registered in 40	N/A	• Polypharmacy is common and significantly associated with multimorbidity, although considerable variation exists between different conditions.

					Scottish GP surgeries.		• The impact of clinical conditions on the number of medicines is generally less in the presence of co-existing concordant conditions.<br>•
Khatter et al. 2020	Prevalence and predictors of potentially inappropriate prescribing in middle-aged adults: a repeated cross-sectional study	UK	Cross sectional study	• ≥4 repeat medicines<br>• Focus on Potentially inappropriate prescribing (PROMPT)	>50,000 middle aged adults (45–64 years taking ≥1 medicine.	N/A	• The prevalence of PIP decreased from 20% in 2014 to 18% in 2019.<br>• The most prevalent PROMPT criteria were the use of ≥2 drugs from the same pharmacological class (7.6%), use of non-steroidal anti-inflammatory drugs for >3 months (7.1%) and use of proton pump inhibitors above recommended maintenance dosages for >8 weeks (3.1%).<br>• Over the study period, the prevalence of multimorbidity increased (47–52%) and polypharmacy was stable (27%).<br>• Polypharmacy, multimorbidity, deprivation, and age were independently associated with PIP. Sex was the only variable not associated.
Molist-Brunet et al. 2022	Improving individualized prescription in patients with multimorbidity through medication review	Spain	Before and after study	• ≥5 medications continuously<br>• severe or excessive polypharmacy: ≥10 chronic medications	428 older adults (≥65 years, mean age 85.5) with multimorbidity (≥2 morbidities) across 3 primary care centres and 3 nursing homes.	Medication review by an interdisciplinary team (primary care team, consultant geriatrician and clinical pharmacist) by applying the Patient-Centered Prescription model (based on CGA) to align the treatment with care goals.	• An individualized medication review in frail older patients, applying the Patient-Centered Prescription model, decreases pharmacological parameters related to adverse drug effects, such as polypharmacy, therapeutical complexity, and anticholinergic and, or sedative burden.<br>• A decrease in polypharmacy, medication regimen complexity index, and drug burden index was more frequent among frail patients, especially those with severe frailty
San-Jose et al. 2021	Integrated health intervention on polypharmacy and inappropriate prescribing in elderly people with multimorbidity: Results	Spain	Before and after study	• ≥ 5 medicines<br>• Focus on potentially inappropriate prescribing (STOPP/START)	100 older adults (≥ 65 years) with high multimorbidi ty according to Adjusted Morbidity Groups (AMG 3-4) with a	A CGA and appropriate prescribing review of medicines were performed by the team (one GP,	• An integrated health intervention centred on polypharmacy in elderly people improves inappropriate prescribing that persists beyond the intervention.<br>• The proportion of patients with two or more STOPP criteria reduced from 37% at the beginning of the intervention to 18% at the end,

	at the end of the intervention and at 6 months after the intervention				recent unplanned hospital visit (including ED)	one medical doctor, an ANP and a day nurse), then a care plan was developed.	and the proportion of those with START criteria from 13% to 6%. These differences persisted at 6 months.
Basger et al. 2012	Validation of prescribing appropriateness criteria for older Australians using the RAND/UCLA appropriateness method	Australi a	Consensus study	• Use of multiple medications<br>• Focus on prescribing appropriateness	N/A	N/A	• A set of 41 Australian prescribing appropriateness criteria were validated by an expert panel.<br>• Use of these criteria, together with clinical judgement and other medication review processes such as patient interview, is intended to assist in improving patient care by efficiently detecting potential drug related problems related to commonly occurring medicines
Burt et al. 2018	Developing a measure of polypharmacy appropriateness in primary care: systematic review and expert consensus study.	UK	Consensus study (with review)	• Use of multiple medications in a single individual<br>• Focus on polypharmacy appropriateness	N/A	N/A	• Produced a set of 12 indicators of clinical importance considered relevant to polypharmacy appropriateness.<br>• Panel members particularly valued indicators concerned with adverse drug reactions, contraindications, drug-drug interactions, and the conduct of medication reviews.
Cooper et al. 2014	The development of the PROMPT (PRescribing Optimally in Middle-aged People's Treatments) criteria	UK and Ireland	Consensus study (with review)	Focus on potentially inappropriate prescribing	Middle-aged adults (45-64 years)	N/A	• PROMPT is the first set of prescribing criteria developed for use in middle-aged adults<br>• A final list of 22 criteria reached consensus after two rounds
George et al. 2004	Development and Validation of the Medication Regimen Complexity Index	Australi a	Consensus study (with review)	• Use of multiple medications<br>• Focus on complexity	N/A	N/A	• A 65–item Medication Regimen Complexity Index (MRCI) was developed.<br>• The total MRCI score had significant correlation with the number of drugs in the regimen, but not with the age and gender of the patients.
Mann et al. 2022	Development of a deprescribing manual for frail older people for use in the COFRAIL study and in primary care	German y	Consensus study	• ≥5 medications<br>• Focus on deprescribing	Frail older patients (≥65 years	Structured manual to support deprescribing of medicines in frail, older adults in primary care	• After piloting and revisions, the deprescribing manual now covers 11 indications/topics.<br>• In each chapter, patient- and medication-related deprescribing criteria, monitoring and communication strategies, and information about concerns related to the use of specific drugs in older patients are provided.
Turner et al. 2016	What factors are important for	Australi a	Consensus study	• ≥5 medications	Residents of long-term care	Nominal group methods with 19	• No two groups had the same priorities. GPs ranked 'evidence for deprescribing' and

	deprescribing in Australian long-term care facilities? Perspectives of residents and health professionals			• Appropriate and inappropriate prescribing<br>• Focus on deprescribing of unnecessary or potentially inappropriate medications	facilities with multimorbidity and polypharmacy across metropolitan and regional South Australia.	GPs, 12 nurses, 14 pharmacists, and 11 patients and carers	'communication with family/resident' as most important factors<br>• Pharmacists ranked 'clinical appropriateness of therapy' and 'identifying residents' goals of care' as most important.<br>• The multidisciplinary groups prioritised both 'adequacy of medical and medication history' and 'identifying goals of care'.
