# Supplementary material for: Defining, identifying and addressing problematic polypharmacy within multimorbidity in primary care: a scoping review
Source: BMJ Open. 2024 May 24;14(5):e081698. doi: 10.1136/bmjopen-2023-081698 (PMC11129052; doi:10.1136/bmjopen-2023-081698)
Supplement: Author's manuscript [file bmjopen-2023-081698.draft_revisions.pdf]

BMJ Open is committed to open peer review. As part of this commitment we make the peer review history of every article we publish publicly available.

When an article is published we post the peer reviewers' comments and the authors' responses online. We also post the versions of the paper that were used during peer review. These are the versions that the peer review comments apply to.

The versions of the paper that follow are the versions that were submitted during the peer review process. They are not the versions of record or the final published versions. They should not be cited or distributed as the published version of this manuscript.

BMJ Open is an open access journal and the full, final, typeset and author-corrected version of record of the manuscript is available on our site with no access controls, subscription charges or pay-per-view fees (http://bmjopen.bmj.com).

If you have any questions on BMJ Open's open peer review process please email
info.bmjopen@bmj.com

BMJ Open

Defining, identifying, and addressing problematic polypharmacy within multimorbidity in primary care: A scoping review

Journal:	*BMJ Open*
Manuscript ID	bmjopen-2023-081698
Article Type:	Original research
Date Submitted by the Author:	03-Nov-2023

Complete List of Authors:	Tsang, Jung Yin; The University of Manchester Division of Population Health Health Services Research and Primary Care, Centre for Primary Care and Health Services Research, School of Health Sciences; The University of Manchester, NIHR Greater Manchester Patient Safety Research Collaboration (GMPSRC), Faculty of Biology, Medicine and Health, Manchester Academic Health Sciences Centre (MAHSC)<br>Sperrin, Matthew; The University of Manchester, Division of Informatics, Imaging and Data Sciences, School of Health Sciences; The University of Manchester, NIHR Greater Manchester Patient Safety Research Collaboration (GMPSRC), Faculty of Biology, Medicine and Health, Manchester Academic Health Sciences Centre (MAHSC)<br>Blakeman, Thomas; The University of Manchester Division of Population Health Health Services Research and Primary Care, Centre for Primary Care and Health Services Research, School of Health Sciences; The University of Manchester, NIHR Greater Manchester Patient Safety Research Collaboration (GMPSRC), Faculty of Biology, Medicine and Health, Manchester Academic Health Sciences Centre (MAHSC)<br>Payne , Rupert A ; University of Exeter Medical School, Department of Health and Community Sciences<br>Ashcroft, Darren; The University of Manchester Faculty of Biology Medicine and Health, Division of Pharmacy and Optometry, School of Health Sciences; The University of Manchester, NIHR Greater Manchester Patient Safety Research Collaboration (GMPSRC), Faculty of Biology, Medicine and Health, Manchester Academic Health Sciences Centre (MAHSC)

Keywords:	Drug Combinations, Chronic Disease, Adverse events < THERAPEUTICS, Primary Health Care, Primary Prevention, Medication Adherence

SCHOLARONE™ Manuscripts

I, the Submitting Author has the right to grant and does grant on behalf of all authors of the Work (as defined in the below author licence), an exclusive licence and/or a non-exclusive licence for contributions from authors who are: i) UK Crown employees; ii) where BMJ has agreed a CC-BY licence shall apply, and/or iii) in accordance with the terms applicable for US Federal Government officers or employees acting as part of their official duties; on a worldwide, perpetual, irrevocable, royalty-free basis to BMJ Publishing Group Ltd ("BMJ") its licensees and where the relevant Journal is co-owned by BMJ to the co-owners of the Journal, to publish the Work in this journal and any other BMJ products and to exploit all rights, as set out in our licence.

The Submitting Author accepts and understands that any supply made under these terms is made by BMJ to the Submitting Author unless you are acting as an employee on behalf of your employer or a postgraduate student of an affiliated institution which is paying any applicable article publishing charge ("APC") for Open Access articles. Where the Submitting Author wishes to make the Work available on an Open Access basis (and intends to pay the relevant APC), the terms of reuse of such Open Access shall be governed by a Creative Commons licence – details of these licences and which Creative Commons licence will apply to this Work are set out in our licence referred to above.

Other than as permitted in any relevant BMJ Author's Self Archiving Policies, I confirm this Work has not been accepted for publication elsewhere, is not being considered for publication elsewhere and does not duplicate material already published. I confirm all authors consent to publication of this Work and authorise the granting of this licence.

For peer review only

Defining, identifying, and addressing problematic polypharmacy within multimorbidity in primary care: A scoping review

Jung Yin Tsang [1,2,3]
Matthew Sperrin [2,3]
Thomas Blakeman [1,2]
Rupert A Payne [4]
Darren M Ashcroft [2,5]

Authors and affiliations:
[1]Centre for Primary Care and Health Services Research, School of Health Sciences, University of Manchester, UK
[2]NIHR Greater Manchester Patient Safety Research Collaboration (GMPSRC), Faculty of Biology, Medicine and Health, Manchester Academic Health Sciences Centre (MAHSC), University of Manchester, UK
[3]Division of Informatics, Imaging and Data Sciences, School of Health Sciences, University of Manchester, UK
[4]Department of Health and Community Sciences, University of Exeter Medical School, Exeter, United Kingdom
[5]Division of Pharmacy and Optometry, School of Health Sciences, University of Manchester, UK

Corresponding author:
Jung Yin Tsang
jungyin.tsang@manchester.ac.uk
Centre for Primary Care and Health Services Research, 6th Floor Williamson building, Oxford Road, University of Manchester, Manchester, UK, M13 9PL
Tel: 0161 275 0752

Word count: 3998 words
Key words: Polypharmacy; multimorbidity; Overtreatment; Inappropriate Prescribing; Deprescriptions; Adverse Drug Reaction; Drug Side Effects.

For peer review only

ABSTRACT

Introduction

Polypharmacy and multimorbidity pose escalating challenges. Despite numerous attempts, interventions have yet to show consistent improvements in health outcomes. A key factor may be varied approaches to targeting patients for intervention.

Objectives

To explore how patients are targeted for intervention by examining the literature with respect to understanding how polypharmacy is defined, identifying problematic polypharmacy in practice, and addressing problematic polypharmacy through interventions

Design

Scoping review as defined by the Joanna Briggs Institute (JBI)

Setting

Primary care

Data sources

Medline, Embase, CINAHL, and Cochrane along with ClinicalTrials.gov, Science.gov and WorldCat from January 2004 to December 2022

Eligibility criteria

We included all articles that had a focus on problematic polypharmacy in multimorbidity and primary care, incorporating multiple types of evidence, such as reviews, quantitative trials, qualitative studies, and policy documents. Articles focussing on a single index disease or not written in English were excluded.

For peer review only

Extraction and Analysis

We performed a narrative synthesis, comparing themes and findings across the collective evidence to draw contextualised insights and conclusions.

Results

In total, 138 articles were included. Though there is no consensus on a definition for polypharmacy, there has been a shift towards more clinically relevant definitions such as 'appropriate', 'inappropriate' and 'problematic' polypharmacy. Case-finding methods often rely on basic medication counts (often 5 or more) without considering past medical history or whether individual medications are clinically appropriate. Other approaches highlight specific drug indicators or interactions as potentially inappropriate prescribing, failing to capture a proportion of patients not fitting criteria. This may hinder the identification of the precise population requiring intervention.

Conclusions

The targeting of patients with polypharmacy should consider patient perspectives, routine practice, system-related factors, and organisational challenges. The development of a comprehensive measure of problematic polypharmacy that consistently incorporates adjustment for multimorbidity may be a valuable next step to address frequent confounding.

For peer review only

For peer review only - http://bmjopen.bmj.com/site/about/guidelines.xhtml

STRENGTHS AND LIMITATIONS OF THIS STUDY

- This is the first scoping review to explore and conceptualise how patients with problematic polypharmacy are targeted for intervention
- It includes multiple types of evidence, including systematic reviews, quantitative, qualitative, and mixed methods studies, along with policy documents.
- Our synthesis capitalises on the shared challenges involved in managing both polypharmacy and multimorbidity with a greater focus on articles regarding polypharmacy in chronic conditions. rather than acute medication adjustments.
- It was not always possible to separate results in studies encompassing both primary and secondary care

For peer review only

INTRODUCTION

Polypharmacy in multimorbidity is an increasing global priority.[1] With an ageing population, over a quarter of the population are living with Multiple Long Term Conditions (MLTC), i.e. multimorbidity, often associated with polypharmacy, which is broadly defined as the use of multiple medications.[2] Medications carry clear benefits, yet the use of multiple medicines can be linked to adverse consequences, including increased treatment burden, unplanned hospitalisation and death.[3,4] For single conditions, people with more severe disease often require more medications. For example, the National Institute for Health and Care Excellence (NICE) guidelines recommend six medicines to be initiated post-myocardial infarction for secondary prevention.[5] Yet in multimorbidity, the number of medicines quickly add up, with limited evidence of benefit over risk as this population is frequently excluded in trials.[6] As the number of medicines prescribed increases, so does the direct risk of adverse drug reactions, increasing health service costs and utilisation, reducing adherence, and decreasing quality of life.[7–9] This can be particularly problematic for older patients, for whom prescribing is more common and thus associated with greater possibility of prescribing errors. Moreover the risks of harms are increased due to changes in pharmacokinetics (e.g. impaired drug metabolism, changes in drug binding) and pharmacodynamics (e.g. increased sensitivity to adverse effects).[10,11] Problematic polypharmacy has previously been defined as “the prescribing of multiple medications inappropriately, or where the intended benefit of the medication is not realised.”[3]

Despite numerous interventions targeting polypharmacy, there remains little evidence of improvement of health outcomes, such as hospitalisations and death.[12–14] However, some reductions in inappropriate prescribing have been observed. Successes of these interventions were highly variable and greatly affected by differences in implementation and targeting of

For peer review only - http://bmjopen.bmj.com/site/about/guidelines.xhtml

patients.[12–14] Further conceptualising the complex and varied approaches to targeting patients with problematic polypharmacy and multimorbidity may inform empirical research and improve future intervention design.[2] Therefore, a scoping review was performed, as this is an effective approach for assessing a broad evidence base. This review centres on considering the pivotal role of primary care professionals and capitalises on the shared challenges involved in managing polypharmacy and multimorbidity. The overarching aim of the review was to explore how patients are targeted for intervention by examining the literature with respect to 1) understanding how polypharmacy is defined; 2) identifying problematic polypharmacy in practice; and 3) addressing problematic polypharmacy through interventions.

METHODS

A scoping review as defined by the Joanna Briggs Institute (JBI) was performed consistent with the Preferred Reporting Items for Systematic Reviews extension for Scoping Reviews (PRISMA-ScR) guidance.[15] This allowed an exploration of both breadth and depth of the topic, which was imperative given the complexity and heterogeneity of evidence. We purposely retained multiple types of evidence (e.g. randomised controlled trials (RCT), consensus trials, qualitative video ethnography) to allow learning through quantitative, qualitative, mixed methods studies, as well as policy and grey literature, to increase relevance and examine the latest evidence base to date.

Search Strategy

A literature search was conducted within MEDLINE, Embase, CINAHL and Cochrane Database of Systematic Reviews in Jan 2023. Search terms covering our population, concept and context of interest are provided in table 1. This included the population of people with

For peer review only

multimorbidity, the concept of problematic polypharmacy, and the context of primary care. We limited our final search strategy to include only articles from 2004 onwards based on the earliest date of relevant articles from a preliminary search. Three additional databases were then searched for grey literature: ClinicalTrials.gov, Science.gov and WorldCat in Feb 2023. We then followed an iterative process of snowballing through a supplementary search of references, citation lists and related articles using Google Scholar. Consistent with scoping reviews guidance, critical appraisal was not undertaken.

Table 1: Search terms used

Category	Search terms used
Multimorbidity	Multimorbid* or multiple long-term conditions or multiple health conditions
Polypharmacy	Polypharmacy or polypharmacotherapy or hyperpolypharmacy or polymedicine* or polimedicin* or multiple medic* or multimedic* or inappropriate prescrib* or overprescrib* or underprescrib* or deprescrib*
Primary Care	Primary care or primary healthcare or general practi*

Eligibility criteria

The eligibility criteria with typical exclusion examples are presented in table 2, guided by the Population, Concept and Context framework recommended by PRISMA-ScR:

- Population – Adults living with multimorbidity
- Concept – Problematic polypharmacy
- Context – Primary Care

For peer review only

Table 2: Eligibility criteria and typical exclusion examples

Inclusion criteria	Typical exclusion examples
Population<br>• Studies must include adults (18 years and older)<br>• Studies must focus on those with multimorbidity – defined as 2 or more long-term conditions, not linked to an "index disease"	• Studies focussing on diabetic patients with renovascular disease (I.e. has an index condition of diabetes)
Concept<br>• Studies focussing on polypharmacy – defined as the concurrent use of multiple medications<br>• Studies that consider the long-term clinical impact of multiple medicines<br>• Studies that consider the consequences of multiple medicines or the 'problematic' element of polypharmacy	• Studies focussed on single medications<br>• Studies based on prescribing of antibiotics for acute presentations only<br>• Studies that are simply descriptive of the number of tablets taken and do not report any risk factors, outcomes, or consequences
Context<br>• Studies with relevance to primary care, including studies which crossed the primary-secondary care interface.	• Studies solely on hospital-based pharmacists
Study type<br>• Studies written in English<br>• Studies presenting full descriptions of the research (e.g. research studies, systematic reviews, randomised controlled trials, pilot studies, and policy documents)	• Letters, comments, conference abstracts, protocols, proceedings, etc.

Study selection

Studies meeting the inclusion criteria were initially selected, based on screening the titles, abstracts, and subsequent full papers by one researcher (JT). A random selection of 10% the records were analysed independently by a second researcher (TB) with 97% agreement of inclusion. Disagreements were resolved through discussion with the wider team.

For peer review only

Data Extraction and Analysis

The data was extracted from eligible studies using a standardised data extraction form and included the author, year of publication, country of origin, type of the publication, polypharmacy definitions, type of participants, descriptions of interventions (if applicable), and key findings (see additional file 1). Further elaboration of the extracted data involved grouping studies according to their focus on either defining, identifying, and addressing polypharmacy, with some spanning multiple elements. The main analysis took the form of a narrative synthesis, using mainly qualitative descriptive data consistent with PRISMA-ScR guidance.[18] This compared themes and findings from grouped studies and using the collective evidence to draw contextualised insights and conclusions.

RESULTS

The search yielded 667 unique articles, with the process illustrated in figure 1. During eligibility screening 457 were excluded after assessment of the abstract and 72 further full-text articles were excluded. 138 articles were included in the final synthesis (supplementary file 1). This included 49 meta-analyses and reviews, 43 quantitative (including 8 RCTs, and 14 longitudinal analyses), 33 qualitative studies (including 6 consensus studies and 2 RCT evaluations), 8 pilot or feasibility studies, and 5 policy documents. The literature was varied with international articles covering a range of polypharmacy issues, from definitions to interventions, with some focussing on sub-populations with multimorbidity (e.g. frailty) and subcategories within the broader context of primary care (e.g. residential care facilities).

[INSERT FIGURE 1]

For peer review only

Understanding how polypharmacy is defined

<u>Numerous polypharmacy definitions</u>

There is no consensus on a definition for polypharmacy, with significant variations in approaches to targeting problematic polypharmacy.[2,3,16] Over 100 definitions of polypharmacy have been used, reflecting the discordance of approaches.[17,18] Two main approaches to defining polypharmacy can be grouped into quantitative (utilising a form of medication count) and qualitative definitions (utilising descriptive notions of prescribing quality), with some studies utilising a combination of these definitions. Table 3 gives illustrative examples of these definitions.

Table 3: Illustrative list of examples for polypharmacy definitions

Definitions	Descriptions/ Examples
**Quantitative definitions**	
Single cut-offs of medication count	≥2, ≥3, ≥4, ≥5, ≥8, ≥10, ≥11 or ≥20 medications
Single cut-offs of a medication group	>2 anticholinergic medications<br>>3 antipsychotic medications
Groups of medication counts	0–4 medications, 5–9 medications, 10–14 medications, ≥15 medications<br>0–5 medications, 6–8 medications, 9–11 medications, ≥12 medications<br>0–6 medications, 7–9 medications, 10–13 medications, ≥14 medications
Categorisation with levels or attributes	Mild polypharmacy 1–4 or 2–3 medications<br>Minor polypharmacy 2–4 medications<br>Major polypharmacy ≥5 medications<br>Standard polypharmacy 5–9 or 6-9 medications<br>Severe polypharmacy ≥6 or ≥10 medications<br>Extreme polypharmacy ≥10 medications<br>Hyper-polypharmacy ≥10 medications<br>High-level polypharmacy ≥10 medications
**Qualitative definitions**	
Overprescribing	More medications than clinically indicated or unnecessary medications or presence of medications with no clinical indications or for which a safer alternative exists
Underprescribing	Lack of an indicated medication, or prescribed an inadequate amount or prescribed less frequent than appropriate
Drug-drug interactions	Any potential interaction, or harmful combination
Inappropriate medications	Defined by set criteria, for example overprescribing, mis-prescribing and potential interactions
Prescribing cascade	Medication prescribed to treat the side effect of another medication
Absence of indication	Medication not matching the diagnosis
Therapeutic duplication	Same medicine used more than once or two within same therapeutic group used (e.g. multiple antidepressants)

For peer review only

No therapeutic benefit	Medications with lack of effectiveness
Not cost-effective	Availability of an equally effective, lower-cost alternative

Illustrative examples of wide range of definitions for polypharmacy used in the literature.[17,18] Generally, quantitative definitions focus on operationalising medication count, regardless of whether polypharmacy is problematic. Whereas most qualitative definitions attach descriptors to describe scenarios where polypharmacy may be clinically problematic.

Quantitative definitions of polypharmacy were more frequent, with over 90% of publications utilising some form of medication count.[2,17–20] For example, the World Health Organization defines polypharmacy as four or more medicines, academic studies most commonly use five or more.[1,2] Other quantitative definitions included categorisations rather than cut offs of medication count. These were frequently labelled levels (e.g. mild, moderate and severe) or attributes (e.g. excessive, extreme), yet counts within these categories were also inconsistent.[17,18,21,22] Generally, quantitative definitions were easier to operationalise and more reproducible, with a focus on medication count, regardless of whether polypharmacy is problematic. In contrast, qualitative definitions largely required clinical judgement to evaluate prescribing quality, carrying a focus on when polypharmacy becomes problematic. This frequently highlighted the overuse or overprescribing of medications. But definitions also covered aspects of mis-prescribing, often through applying a list of defined prescribing criteria, and also under-prescribing, though only a few studies emphasised this aspect. The terms ‘appropriate’, ‘inappropriate’ and ‘problematic’ polypharmacy were also commonly used to describe when multiple medications were justified compared to when the clinical indication was unclear.[3,17,18,23] These definitions have now been expanded to cover further dimensions of polypharmacy, such as the increasing recognition of the importance of patient and carer input in determining the appropriateness of medications.[24,25]

For peer review only

The challenges of defining when polypharmacy is 'problematic'

The understanding of polypharmacy has progressed over time, with an increasing shift to more clinically applicable definitions. This reflects the increasing complexity of decision making for combinations of medicines tailored to individual needs. There is also recognition that it is not possible to account for clinical appropriateness through simple medication counts.[17,18,26] Commonly people with multiple health needs may well be appropriately prescribed more than 10 medications for therapeutic and symptomatic benefit, which would be termed extreme polypharmacy in some studies and guidelines.[27,28] Yet there is some validity to numeric approaches as increasing medications are strongly associated with drug related problems, and very high counts of medication are usually questionable.[29] There is also a need to improve the consistency of reporting medication exposure characteristics.[17,18,30–32] Various definitions have been used to define temporality and 'long-term' use, with some publications including 'acute' and 'as required' medications as opposed to chronic medications, with varied definition of time periods (ranging from 1-240 days).[17,18] Terms such as problematic polypharmacy and inappropriate polypharmacy have been increasingly favoured, as they consider appropriateness and clinical decision-making.[33] Yet qualitative research suggest these labels were still insufficient to reflect the complexity of medicines management, with practitioners juggling terms such as "potentially inappropriate" and "specifically appropriate" and others considering them "judgemental" and even "accusatory".[34]

Identifying problematic polypharmacy in practice

Targeting potentially high-risk populations

Various strategies target higher risk populations to try and identify problematic polypharmacy. One common approach use simple cut offs of age (commonly ≥ 65 years) combined with cut

For peer review only - http://bmjopen.bmj.com/site/about/guidelines.xhtml

offs of medications (frequently ≥ 5) and this was the main inclusion criteria for the majority of trials.[12] Another approach adopted by multiple national recommendations advocate case finding through high risk groups.[35–37] For instance, both NICE guidelines and the Australian Commission on Safety and Quality in Healthcare recommend greater attention for older people with frailty, and complex multimorbidity and co-existing mental and physical health problems.[2,28,35] Accordingly, several national indicators, initiatives and studies also use combinations of these approaches.[36–42] Other approaches include risk scores to identify patients at high-risk of particular outcomes (e.g. hospitalisations, or adverse drug reactions) but these require further development.[43,44] Overall, strategies to identify potentially high-risk populations currently demonstrate variable validity in polypharmacy and are seldom comprehensive or holistic, as they are specific to the needs of particular groups.[2,35,45]

Targeting potentially inappropriate medicines

Evaluating the appropriateness of individual medications is a common approach both as a case-finding approach and as a surrogate measure of prescribing quality across polypharmacy. Various tools have been developed to identify potentially inappropriate medicines and these can be split into explicit and implicit tools, with some tools combining both (examples in table 4).[46–48] The majority have been developed using expert opinion and consensus methodology, and originally were designed for evaluating individual medications, rather than polypharmacy as a whole.[46,49] Explicit tools contain specific criteria or scenarios leading to potential adverse drug events and carry advantages of reproducibility and ease of automation.[50–52] Implicit tools require judgement, which means they can be subjective and demand more time and clinical expertise. Nevertheless, explicit tools are limited to specific drugs and diseases, but implicit tools can be applied to any medication. This perhaps allows implicit tools greater applicability in polypharmacy, as explicit tools will miss out any medicines outside criteria.[53]

For peer review only

268

269 *Table 4: Key examples of explicit and implicit tools of appropriate prescribing*

Tool	Description	Strengths	Limitations
**Beers Criteria** *(Explicit tool)*	- First widely used explicit criteria<br>- Contains 84 criteria (2019 version) including potentially inappropriate medications to be avoided such as drug-disease and drug-drug interactions	- International studies have shown predictive validity for adverse drug reactions, falls, cognitive function, hospitalisation and death.<br>- Endorsed by the American Geriatric Society and updated approximately every 3 years.<br>- Easier to automate in drug records as criteria are very specific	- No positive clinical outcomes in RCTs to date<br>- No prioritisation of medications for review<br>- Can be challenging to use as long list of criteria<br>- Does not address under-prescribing<br>- Focus is on individual medications rather than polypharmacy as a whole
**Screening Tool of Older Person's Prescriptions/ Screening Tool to Alert doctors to Right Treatment – STOPP/START** *(Explicit tool, but newer versions also contain implicit measures)*	- One of the most widely used explicit criteria globally<br>- Contains 80 criteria for potentially inappropriate medications, and 34 potential under-prescribing criteria (version 2), organised according to medication and disease groups	- Some positive outcomes shown in several RCTs<br>- Also addresses aspects of under-prescribing in addition to overprescribing<br>- Easier to automate in computerised drug records as most criteria are very specific	- Misses out medications out of criteria<br>- Potentially usability issues with such a long list<br>- No prioritisation of medications for review<br>- Can be challenging to use as long list of criteria<br>- Focus is on individual medications rather than polypharmacy as a whole
**Medication Appropriateness Index – MAI** *(Implicit tool)*	First widely used implicit criteria<br>Lists 10 criteria that evaluate various aspects of medication appropriateness (e.g. indication, effectiveness, dose)	- Some positive outcomes shown in several RCTs<br>- Can be applied to all medicines	- Time-consuming to execute<br>- Requires clinical expertise and can be subjective<br>- Difficult to automate<br>- No prioritisation of medications for review<br>- Focus is seldom on polypharmacy as a whole or under-prescribing
**Drug Burden Index – DBI** *(Implicit tool, as requires further judgement to evaluate appropriateness after calculating score)*	- Widely researched risk score<br>- Calculates the cumulative exposure of sedatives and anticholinergics to give a score between 0 and 1.	- International studies have shown predictive validity for falls, fractures, GP visits and admission.<br>- Takes into account licenced doses to allow transferability between counties<br>- Easier to automate in drug records.	- No positive clinical outcomes in RCTs to date<br>- No consideration for appropriateness or specific indication of medicines<br>- Only focussed on sedatives, and anticholinergics<br>- Can be challenging to calculate at point of care unless computerised<br>- Does not address polypharmacy as a whole or under-prescribing

270 A descriptive summary of selected examples of widely studied explicit and implicit tools.[47,54]

271

For peer review only - http://bmjopen.bmj.com/site/about/guidelines.xhtml

Several systematic reviews have revealed a high level of variability of included criteria within explicit tools.[46–49] A review of 36 explicit tools reported criteria spanning 907 medications and medication classes, but only 44 medications and 4 classes were reported by the majority.[47] This was despite over 85% of these tools being developed based on either the Beers or the STOPP/START (Screening Tool of Older Person's Prescriptions/Screening Tool to Alert doctors to Right Treatment) criteria).[47] Due to this, many studies combine several explicit criteria to complement the list of medications included.[46,47,49,55,56] Only about a third of tools suggested alternative treatments to potentially inappropriate medicines, yet nearly 70% of suggested alternatives were deemed inappropriate by other tools.[46] Implicit tools are also diverse in nature, with reviews identifying over 16 different tools incorporating implicit criteria.[54,57,58] These ranged from risk scores to lists of questions specifying appropriate use or criteria to evaluate the administrative burden to patients.[54,57,59] Several tools combine implicit and explicit indicators, including documents used for national guidance (e.g. Australian Prescribing Indicators Tool).[57,60–62]

Key limitations in identifying problematic polypharmacy in practice

Current strategies to identify problematic polypharmacy demonstrate inadequate performance. At present, risk stratification tools remain too broad, and seldom consider the clinical appropriateness of individual medications.[33,63] Though comprehensive explicit criteria are helpful in identifying potentially inappropriate medications, translation into everyday care remains elusive due to challenges in clinical application, and the omission of medications not included in criteria. For instance, previous studies have found that less than 25% of adverse drug reactions are caused by drugs listed by Beers criteria.[64,65] Additionally, apart from STOPP/START, most widely used tools were not designed to also cover under-prescribing (Table 4), with some studies also choosing to omit many of the under-prescribing criteria in its

For peer review only - http://bmjopen.bmj.com/site/about/guidelines.xhtml

application.[46–49] Furthermore, there has been questions as to the utility of long lists of medications as studies have shown a high prevalence of potentially inappropriate medications (over 30% of patients) but low variability within many criteria, potentially leaving little room for improvement.[66] Studies also mention usability issues with such long lists, even with computerised integration, and the difficulties of making treatment decisions without prioritisation of criteria, particularly as their predictive validity is unknown.[46,63,67–69] Still, as the majority of instruments were developed focussing on patients over 65 years old, the suitability for middle-aged adults is unknown, yet both polypharmacy and multimorbidity are increasing in this age-group.[19,29] Only a handful of criteria have been developed and validated (e.g. PRescribing Optimally in Middle-aged People's Treatments - PROMPT criteria), all including significantly fewer criteria for individual medications and medication classes.[53,54,57,70,71] Again, this further limits applicability in problematic polypharmacy, where the whole of the medication regimen should be considered.

Addressing problematic polypharmacy through interventions

Large variability in interventions addressing polypharmacy

Interventions to address problematic polypharmacy have covered a wide range of aims, such as reducing adverse drug reactions, increasing the appropriateness of medicines use, reducing falls, improving patient adherence, and maintaining quality of life.[12,13,72] To combat overprescribing specifically, deprescribing interventions have also received significant attention, though interventions that focus on under-prescribing are much less.[73–77] Several large reviews highlight good evidence of improving prescribing patterns, yet mixed and low certainty of evidence in improving patient-relevant outcome measures.[3,12–14,78–81] Reviews covering over 150 primary studies reported no differences in all-cause mortality and no clear evidence of benefit in reduced hospitalisations, when comparing interventions to usual care.[12,72,79,82–85]

For peer review only - http://bmjopen.bmj.com/site/about/guidelines.xhtml

There were also no differences in quality of life, adverse drug reactions, readmission rates, primary care visits and emergency department visits.[12,72,83–85] Two reviews have highlighted some economic benefits in reducing healthcare expenditure, but others highlight inconsistencies due to low quality evidence.[83,84,86] Overall, there is evidence that these interventions are safe and do not lead to harm, but may be time and resource intensive for both patients and practitioners, as many require continuing input.[80,82] Likewise, mixed evidence of improved clinical outcomes, such as falls, is also observed even in more focussed populations, such as those with frailty and in long-term care facilities.[75–77,87]

<u>Multiple intervention components to address polypharmacy, with unclear effectiveness</u>

The majority of polypharmacy interventions were multimodal with a review revealing fourteen different elements from 80 studies and an average of 2.5 elements per intervention.[12,88] The most common elements included medication reviews and training for professionals and utilising tools, such as clinical decision support, checklists or audit and feedback.[12,69,88–91] Other components strengthened inter-professional or multidisciplinary collaborations by involving clinical pharmacists, nurses or geriatricians.[12,83,85,88,89,92–98] There were also patient facing components, such as education and training for patients and patient interviews to seek their understanding and lived experiences with their medicines.[99–104] Despite the growing literature on the importance of patient-centred care in medicines management, current literature highlights that patient priorities are seldom fully integrated into polypharmacy interventions.[12,74,82,88,105–111] Patient-centred approaches also appear to be key to improving adherence, as a frequent discordance between practitioner and patient views is reported.[12,88,112–117] More recent interventions that do adopt a patient centred model, show some mixed improvements in appropriate prescribing and limited improvements in outcomes, reflecting

For peer review only - http://bmjopen.bmj.com/site/about/guidelines.xhtml

some of the challenges of integrating patient priorities into routine medication reviews.[91,99–104,118]

In terms of effectiveness of individual intervention elements, similar effect sizes have been observed in reducing the number of potentially inappropriate medicines, with no particular components showing particular superiority.[12,62] However, generalised professional education programmes appear to be less effective than individualised interventions.[12,119] Medication reviews are also the most commonly adopted component, but as a single intervention, there remains insufficient evidence of medication reviews alone improving clinical outcome.[75,120,121] Despite the advantages of automation, electronic tools in trials demonstrate high variability in implementation within large pan-European and global trials, and no clear positive advantages on relevant patient outcomes have been reported.[12,122,123] Pharmacists show promise as an extra resource for managing polypharmacy in individual studies, but two recent reviews revealed uncertain effects on optimising medicines.[83,85,93–97,124,125] Community pharmacists can contribute to medication safety, but more in-depth management such as polypharmacy medication reviews were seen as outside the scope of community pharmacy.[96,126,127]

<u>Key challenges in addressing problematic polypharmacy</u>

In spite of the breadth of interventions targeting polypharmacy, it remains unclear which intervention components are more important.[12] Theory informed interventions are few and there are opportunities for improvements in intervention design through stronger foundations on theoretical frameworks and behaviour change techniques.[128–132] Widespread variation exists in the everyday management of medicines and polypharmacy.[2,3,133] These variations occur at patient, prescriber, regional and international levels, and indicate links between problematic polypharmacy and health inequalities.[1–3,133,134] As such, multiple challenges to addressing

For peer review only

problematic polypharmacy need to be overcome, going beyond the identification of individual barriers and facilitators and translating these into practice within the complexity of interlinked systems of care.[2,38,135,136] The failure of the implementation of interventions are commonly down to the lack of consideration of integration into an already high demand system in everyday primary care.[136–138]

For patients with polypharmacy and multimorbidity, prioritisation and decision making is a challenge, given that they can receive ten times the amount of information during consultations due to compounding health issues, interacting medications and complex social issues.[139] Yet patient priorities and shared decision making is vital to deciding the appropriateness of medications, so improvements need to be made to both the clarity of information provided and the integration of patient views into polypharmacy decisions.[2,24,25,105,109,139] The majority of patients appear willing to discuss deprescribing medications, particularly if they have a good relationship with their doctor.[74,111,140] However, they also have strong beliefs and attitudes of the value of their medicines, with inertia generated when feeling well on their current medication regimen.[74,111,136,141–144]

For health professionals, work and effort are required to even consider deprescribing, particularly as prescribing is so embedded in routine practice and finding an appropriate time to initiate the discussion is often difficult given competing priorities.[96,137,138,145–147] A comprehensive polypharmacy medication review is described as 'impossible' to complete in 10 minutes, leading to practitioners defaulting to a swifter review and degrading medication reviews to being 'mundane' tasks.[96,141] This is combined with the work to gain awareness (of new policies, guidelines and tools), overcome significant uncertainty in evidence (with 'unmeasurable' risk-benefit), and increase self-efficacy with limited resources and

For peer review only - http://bmjopen.bmj.com/site/about/guidelines.xhtml

alternatives.[138,146–151] On an organisational and systems level, fragmentation of care and poor co-ordination between healthcare teams and specialists often lead to deferring ownership of deprescribing attempts, and miscommunication to patients, leading to medication related problems.[108,145,149,151–153] More comprehensive approaches and better resources are needed to support practitioners and organisations in pushing for improved polypharmacy decisions in a patient centred manner, rather than simply maintaining the 'status quo'.[34,74,146,148]

DISCUSSION

There are significant challenges to targeting patients with problematic polypharmacy and multimorbidity for intervention. Varying definitions exist and reflect the complexity of evaluating problematic polypharmacy, making it difficult to define and measure outcomes and hindering the identification of an optimal population for trials.[3] Overall, the lack of improvements in health outcomes from polypharmacy interventions may have been due to the heterogeneity and complexity of interventions across studies, with a lack of consistency in measuring outcomes. This may also have been attributable upstream to the inconsistent definitions and methods used to identify patients with problematic polypharmacy.[12,78,82,84] Many studies also demonstrate confounding of the outcomes associated with polypharmacy, through insufficient adjustment for multimorbidity.[82] With the bulk of evidence solely focussing on overprescribing and deprescribing, there is a paucity of interventions that also consider under-prescribing, which is also highly prevalent in patients with problematic polypharmacy.[3,13] Variable implementation of these complex interventions also play a major role, particularly for studies incorporating computerised tools.[12,89,101,122,123,135] For future approaches to be successful, it is imperative to consider consistency in defining polypharmacy,

For peer review only

clarity in identifying the population at risk, and evaluating which individual intervention components are more effective.

Implications for further research and practice

Current ways to target patients with problematic polypharmacy fall short. Case-finding methods including many national comparators either focus on limited aspects of prescribing or use a simple count of medications, ignoring appropriateness of medications and disregarding individual factors. Other approaches adopt a relatively narrow approach through highlighting specific drug indicators or interactions, failing to capture a large proportion of patients not fitting criteria. Large lists of explicit criteria remain difficult to implement even with electronic support and are frequently subject to alert fatigue due to variable clinical relevance. The approach to targeting patients may need to be improved as a first step, which may in turn improve how interventions target and evaluate problematic polypharmacy. A next step to enhance clinical utility may be the routine adjustment of multimorbidity, as there is frequent confounding of polypharmacy outcomes within studies. In doing so, we may be able to identify patients who are both over-prescribed and under-prescribed medicines yet consider some degree of clinical appropriateness. An opportunity exists to produce a cross-cutting measure beyond single diseases and individual drug interactions to evaluate patients as a whole, with the aim of improving overall health.[63,148]

The multifactorial drivers of polypharmacy also mean that approaches to address problematic polypharmacy need to go beyond targeting patients and practitioners alone.[38,136] Despite this, evidence of a systems approach encompassing policy makers, organisations, practitioners, patients and carers is lacking.[2,38,135,136] Both the growth of evidence-based medicine and desire to minimise all risk are significant drivers of increased medicines burden and

For peer review only - http://bmjopen.bmj.com/site/about/guidelines.xhtml

problematic polypharmacy. Yet polypharmacy is rarely 'evidence-based', as it would be impossible to have a large enough sample size to perform drug trials and meta-analyses of the millions of combinations that patients with multimorbidity are taking.[154] Studies examining exclusion criteria of RCTs estimates that over 90% of this population would be excluded from trials, questioning their representativeness.[155] The emphasis on following guidelines and increasing treatment intensity should be balanced with the understanding that high quality personalised healthcare can only be achieved through also carefully reducing, stopping, or not initiating medication, with shared decision-making and agreed care objectives.[156,157]

Strengths and Limitations

This scoping review syntheses a wide breath of literature to explore the existing evidence. It allowed a systematic approach on an initial search strategy, but was also adaptable to heterogeneous sources (e.g. policy documents) and developing literature (e.g. pilot studies) through related article, supplementary and grey literature searching. It examined the overlapping concepts of polypharmacy and multimorbidity concurrently, allowing synergies in evidence generation and critique.

There are several limitations of our review to consider. As with other scoping reviews, critical appraisal was not performed. Polypharmacy is an area that has received widespread attention, with hundreds of primary studies and dozens of systematic reviews. Hence, in our attempts to present generalisable findings, the nuances within primary studies may be lost, such as differences in study setting, population, or intervention characteristics. While we made efforts to specifically extract primary care-related findings, it was not always possible to separate results in studies encompassing both primary and secondary care. Furthermore, by

For peer review only - http://bmjopen.bmj.com/site/about/guidelines.xhtml

emphasising multimorbidity and primary care in our search, we may have overlooked research investigating more acute medication adjustments in polypharmacy patients.

CONCLUSION

An optimal approach for targeting patients with problematic polypharmacy for intervention is yet to be determined. To address the challenges posed by confounding, it may be valuable to develop a cross-cutting measure of polypharmacy that consistently accounts for multimorbidity. The complex drivers of polypharmacy and the variable implementation of complex interventions, highlight the importance of considering routine practices, patient perspectives, system-related factors, and organisational challenges within intervention designs.

Author's contributions

All authors were involved in the conceptualisation and design of the study. JT performed the search, selection, extraction and synthesis with contributions from TB. JT wrote the first draft of the manuscript. All authors contributed to the review and approval of the final manuscript.

Competing interests

D.M.A. reports research funding from Abbvie, Almirall, Celgene, Eli Lilly, Novartis, UCB, and the Leo Foundation outside the submitted work. The other authors have no conflicts of interest to disclose.

For peer review only - http://bmjopen.bmj.com/site/about/guidelines.xhtml

Funding statement

JT is funded by the NIHR Doctoral Fellowship Programme (Ref: NIHR302624) for this research project. TB and DMA are supported by the NIHR Greater Manchester Patient Safety Research Collaboration. The views expressed in this document are those of the authors and not necessarily those of the NIHR, NHS or the UK Department of Health and Social Care.

Patient and public involvement

Four members of patients and the public are involved in a steering group and provide invaluable contribution to the design, or conduct, or reporting and dissemination plans of our research.

Data sharing statement

All data relevant to the study are included in the article or uploaded as supplementary information.

REFERENCES

1. World Health Organization. *Medication without Harm*. World Health Organization; 2017.
2. The Department of Health and Social Care. National overprescribing review report: Good for you, good for us, good for everybody. 2021. https://www.gov.uk/government/publications/national-overprescribing-review-report.
3. Duerden M, Avery T, Payne R. Polypharmacy and medicines optimisation. *King's Fund*. 2013.
4. Hajjar ER, Cafiero AC, Hanlon JT. Polypharmacy in elderly patients. *Am J Geriatr Pharmacother*. 2007;5(4):345-351. doi:https://doi.org/10.1016/j.amjopharm.2007.12.002
5. National Institute for Health and Care Excellence (NICE). Acute Coronary Syndromes [NG185]. 2020. https://www.nice.org.uk/guidance/ng185.
6. Buffel du Vaure C, Dechartres A, Battin C, Ravaud P, Boutron I. Exclusion of patients

For peer review only

For peer review only - http://bmjopen.bmj.com/site/about/guidelines.xhtml

with concomitant chronic conditions in ongoing randomised controlled trials targeting 10 common chronic conditions and registered at ClinicalTrials.gov: a systematic review of registration details. *BMJ Open*. 2016;6(9):e012265. doi:10.1136/bmjopen-2016-012265
7. Avery AJ, Ghaleb M, Barber N, et al. *Investigating the Prevalence and Causes of Prescribing Errors in General Practice: The Practice Study*. Vol 21.; 2012.
8. Gellad WF, Grenard JL, Marcum ZA. A Systematic Review of Barriers to Medication Adherence in the Elderly: Looking Beyond Cost and Regimen Complexity. *Am J Geriatr Pharmacother*. 2011;9(1):11-23. doi:https://doi.org/10.1016/j.amjopharm.2011.02.004
9. Olsson IN, Runnamo R, Engfeldt P. Medication quality and quality of life in the elderly, a cohort study. *Health Qual Life Outcomes*. 2011;9(1):95.
10. Davies EA, O'Mahony MS. Adverse drug reactions in special populations – the elderly. *Br J Clin Pharmacol*. 2015;80(4):796-807. doi:https://doi.org/10.1111/bcp.12596
11. Corsonello A, Pedone C, Incalzi RA. Age-related pharmacokinetic and pharmacodynamic changes and related risk of adverse drug reactions. *Curr Med Chem*. 2010;17(6):571-584.
12. Rankin A, Cadogan CA, Patterson SM, et al. Interventions to improve the appropriate use of polypharmacy for older people. *Cochrane database Syst Rev*. 2018;9(9):CD008165. doi:10.1002/14651858.CD008165.pub4
13. Cooper JA, Cadogan CA, Patterson SM, et al. Interventions to improve the appropriate use of polypharmacy in older people: a Cochrane systematic review. *BMJ Open*. 2015;5(12):e009235. doi:10.1136/bmjopen-2015-009235
14. Alldred DP, Kennedy M, Hughes C, Chen TF, Miller P. Interventions to optimise prescribing for older people in care homes. *Cochrane Database Syst Rev*. 2016;(2).
15. Tricco AC, Lillie E, Zarin W, et al. PRISMA Extension for Scoping Reviews (PRISMA-ScR): Checklist and Explanation. *Ann Intern Med*. 2018;169(7):467-473. doi:10.7326/M18-0850
16. Taghy N, Cambon L, Cohen J-M, Dussart C. Failure to Reach a Consensus in Polypharmacy Definition: An Obstacle to Measuring Risks and Impacts—Results of a Literature Review. *Ther Clin Risk Manag*. 2020;16:57.
17. Masnoon N, Shakib S, Kalisch-Ellett L, Caughey GE. What is polypharmacy? A systematic review of definitions. *BMC Geriatr*. 2017;17(1):230. doi:10.1186/s12877-017-0621-2
18. Sirois C, Domingues NS, Laroche M-L, et al. Polypharmacy Definitions for Multimorbid Older Adults Need Stronger Foundations to Guide Research, Clinical Practice and Public Health. *Pharm (Basel, Switzerland)*. 2019;7(3). doi:10.3390/pharmacy7030126
19. Vos R, Boesten J, van den Akker M. Fifteen-year trajectories of multimorbidity and polypharmacy in Dutch primary care—A longitudinal analysis of age and sex patterns. *PLoS One*. 2022;17(2):e0264343. https://doi.org/10.1371/journal.pone.0264343.
20. Aubert CE, Streit S, Da Costa BR, et al. Polypharmacy and specific comorbidities in university primary care settings. *Eur J Intern Med*. 2016;35:35-42.
21. Gutierrez-Valencia M, Aldaz Herce P, Lacalle-Fabo E, Contreras Escamez B, Cedeno-Veloz B, Martinez-Velilla N. Prevalence of polypharmacy and associated factors in older adults in Spain: Data from the National Health Survey 2017. *Med Clin*. 2019;153(4):141-150. doi:https://dx.doi.org/10.1016/j.medcli.2018.12.013 PT - Journal Article

For peer review only

22. Rieckert A, Trampisch US, Klaaßen-Mielke R, et al. Polypharmacy in older patients with chronic diseases: a cross-sectional analysis of factors associated with excessive polypharmacy. *BMC Fam Pract*. 2018;19(1):113. doi:10.1186/s12875-018-0795-5

23. Monégat M, Sermet C, Perronnin M, Rococo E. Polypharmacy: definitions, measurement and stakes involved. *Rev Lit Meas tests*. 2014;8.

24. Mair A, Wilson M, Dreischulte T. Addressing the Challenge of Polypharmacy. *Annu Rev Pharmacol Toxicol*. 2020;60(1):661-681. doi:10.1146/annurev-pharmtox-010919-023508

25. Heaton J, Britten N, Krska J, Reeve J. Person-centred medicines optimisation policy in England: an agenda for research on polypharmacy. *Prim Health Care Res Dev*. 2017;18(1):24-34. doi:10.1017/S1463423616000207

26. Payne RA, Abel GA, Avery AJ, Mercer SW, Roland MO. Is polypharmacy always hazardous? A retrospective cohort analysis using linked electronic health records from primary and secondary care. *Br J Clin Pharmacol*. 2014;77(6):1073-1082. doi:10.1111/bcp.12292

27. Sirois C, Lunghi C, Laroche M-L, Maheux A, Frini A. The delicate choice of optimal basic therapy for multimorbid older adults: A cross-sectional survey. *Res Soc Adm Pharm*. 2019;15(6):761-766. doi:https://doi.org/10.1016/j.sapharm.2018.09.008

28. National Institute for Health and Care Excellence (NICE). Multimorbidity and polypharmacy [KTT18]. 2017.

29. Guthrie B, Makubate B, Hernandez-Santiago V, Dreischulte T. The rising tide of polypharmacy and drug-drug interactions: population database analysis 1995-2010. *BMC Med*. 2015;13:74. doi:10.1186/s12916-015-0322-7

30. McCarthy C, Flood M, Clyne B, et al. Medication changes and potentially inappropriate prescribing in older patients with significant polypharmacy. *Int J Clin Pharm*. 2023;45(1):191-200.

31. von Buedingen F, Hammer MS, Meid AD, Muller WE, Gerlach FM, Muth C. Changes in prescribed medicines in older patients with multimorbidity and polypharmacy in general practice. *BMC Fam Pract*. 2018;19(1):131. doi:https://dx.doi.org/10.1186/s12875-018-0825-3 PT - Journal Article, Research Support, Non-U.S. Gov't

32. Calderon-Larranaga A, Gimeno-Feliu LA, Gonzalez-Rubio F, et al. Polypharmacy patterns: unravelling systematic associations between prescribed medications. *PLoS One*. 2013;8(12):e84967. doi:https://dx.doi.org/10.1371/journal.pone.0084967 PT - Journal Article, Research Support, Non-U.S. Gov't

33. Kadam UT, Roberts I, White S, et al. Conceptualizing multiple drug use in patients with comorbidity and multimorbidity: proposal for standard definitions beyond the term polypharmacy. *J Clin Epidemiol*. 2019;106:98-107. doi:10.1016/j.jclinepi.2018.10.014

34. Clyne B, Cooper JA, Hughes CM, Fahey T, Smith SM. "Potentially inappropriate or specifically appropriate?" Qualitative evaluation of general practitioners views on prescribing, polypharmacy and potentially inappropriate prescribing in older people. *BMC Fam Pract*. 2016;17(1):109. doi:10.1186/s12875-016-0507-y

35. Australian Commission on Safety and Quality in Health Care. Polypharmacy, 75 years and over. 2020. https://www.safetyandquality.gov.au/sites/default/files/2021-04/fourth_atlas_2021_-_6.1_polypharmacy_75_years_and_over.pdf.

36. Scottish Government Polypharmacy Model of Care Group. Polypharmacy guidance,

For peer review only

For peer review only - http://bmjopen.bmj.com/site/about/guidelines.xhtml

realistic prescribing. 3rd Edn. 2018.
37. All Wales Medicines Strategy Group. Polypharmacy in older people: A guide for healthcare professionals. 2023. https://awttc.nhs.wales/files/guidelines-and-pils/polypharmacy-in-older-people-a-guide-for-healthcare-professionalspdf/.
38. McIntosh J, Alonso A, MacLure K, et al. A case study of polypharmacy management in nine European countries: Implications for change management and implementation. *PLoS One*. 2018;13(4):e0195232. https://doi.org/10.1371/journal.pone.0195232.
39. Kruger C, Schafer I, van den Bussche H, et al. Non-random relations in drug use expressed as patterns comprising prescription and over-the-counter drugs in multimorbid elderly patients in primary care: Data of the exploratory analysis of the multicentre, observational cohort study MultiCare. *Eur J Gen Pract*. 2021;27(1):119-129.
40. Villén N, Guisado-Clavero M, Fernández-Bertolín S, et al. Multimorbidity patterns, polypharmacy and their association with liver and kidney abnormalities in people over 65 years of age: a longitudinal study. *BMC Geriatr*. 2020;20(1):206. doi:10.1186/s12877-020-01580-1
41. Monterde D, Vela E, Cleries M, Garcia-Eroles L, Roca J, Perez-Sust P. Multimorbidity as a predictor of health service utilization in primary care: a registry-based study of the Catalan population. *BMC Fam Pract*. 2020;21(1):39.
42. Jager C, Freund T, Steinhauser J, et al. Impact of a tailored program on the implementation of evidence-based recommendations for multimorbid patients with polypharmacy in primary care practices-results of a cluster-randomized controlled trial. *Implement Sci*. 2017;12(1):8. doi:https://dx.doi.org/10.1186/s13012-016-0535-y PT - Journal Article, Randomized Controlled Trial, Research Support, Non-U.S. Gov't
43. Novella A, Elli C, Tettamanti M, et al. Relation between drug therapy-based comorbidity indices, Charlson's comorbidity index, polypharmacy and mortality in three samples of older adults. *Arch Gerontol Geriatr*. 2022;100:104649. doi:https://doi.org/10.1016/j.archger.2022.104649
44. Häppölä P, Havulinna AS, Tasa T, et al. A data-driven medication score predicts 10-year mortality among aging adults. *Sci Rep*. 2020;10(1):15760. doi:10.1038/s41598-020-72045-z
45. Guisado-Clavero M, Violan C, Lopez-Jimenez T, et al. Medication patterns in older adults with multimorbidity: a cluster analysis of primary care patients. *BMC Fam Pract*. 2019;20(1):82.
46. Schiavo G, Forgerini M, Lucchetta RC, Mastroianni P de C. A comprehensive look at explicit screening tools for potentially inappropriate medication: A systematic scoping review. *Australas J Ageing*. 2022;41(3):357-382. doi:https://doi.org/10.1111/ajag.13046
47. Motter FR, Fritzen JS, Hilmer SN, Paniz ÉV, Paniz VMV. Potentially inappropriate medication in the elderly: a systematic review of validated explicit criteria. *Eur J Clin Pharmacol*. 2018;74(6):679-700. doi:10.1007/s00228-018-2446-0
48. Lucchetti G, Lucchetti ALG. Inappropriate prescribing in older persons: A systematic review of medications available in different criteria. *Arch Gerontol Geriatr*. 2017;68:55-61. doi:10.1016/j.archger.2016.09.003
49. Lee G, Lim J-F, Page AT, Etherton-Beer C, Clifford R, Wang K. Applicability of explicit potentially inappropriate medication lists to the Australian context: A systematic review. *Australas J Ageing*. 2022;41(2):200-221.

For peer review only

doi:https://doi.org/10.1111/ajag.13038
50. Vrdoljak D, Borovac JA. Medication in the elderly - considerations and therapy prescription guidelines. *Acta Med Acad*. 2015;44(2):159-168. http://ovidsp.ovid.com/ovidweb.cgi?T=JS&PAGE=reference&D=med12&NEWS=N&AN=26702910.
51. Lopez-Rodriguez JA, Rogero-Blanco E, Aza-Pascual-Salcedo M, et al. Potentially inappropriate prescriptions according to explicit and implicit criteria in patients with multimorbidity and polypharmacy. MULTIPAP: A cross-sectional study. *PLoS One*. 2020;15(8):e0237186. https://doi.org/10.1371/journal.pone.0237186.
52. Sanchez-Fidalgo S, Guzman-Ramos MI, Galvan-Banqueri M, Bernabeu-Wittel M, Santos-Ramos B. Prevalence of drug interactions in elderly patients with multimorbidity in primary care. *Int J Clin Pharm*. 2017;39(2):343-353. doi:https://dx.doi.org/10.1007/s11096-017-0439-1 PT - Journal Article, Review
53. Cooper JA, Ryan C, Smith SM, et al. The development of the PROMPT (PRescribing Optimally in Middle-aged People's Treatments) criteria. *BMC Health Serv Res*. 2014;14(1):484. doi:10.1186/s12913-014-0484-6
54. Masnoon N, Shakib S, Kalisch-Ellett L, Caughey GE. Tools for Assessment of the Appropriateness of Prescribing and Association with Patient-Related Outcomes: A Systematic Review. *Drugs Aging*. 2018;35(1):43-60. doi:10.1007/s40266-018-0516-8
55. Troncoso-Marino A, Lopez-Jimenez T, Roso-Llorach A, et al. Medication-related problems in older people in Catalonia: A real-world data study. *Pharmacoepidemiol Drug Saf*. 2021;30(2):220-228. http://ovidsp.ovid.com/ovidweb.cgi?T=JS&PAGE=reference&D=med19&NEWS=N&AN=33026123.
56. Rogero-Blanco E, Lopez-Rodriguez JA, Sanz-Cuesta T, Aza-Pascual-Salcedo M, Bujalance-Zafra MJ, Cura-Gonzalez I. Use of an Electronic Clinical Decision Support System in Primary Care to Assess Inappropriate Polypharmacy in Young Seniors With Multimorbidity: Observational, Descriptive, Cross-Sectional Study. *JMIR Med informatics*. 2020;8(3):e14130. doi:10.2196/14130
57. Kaufmann CP, Tremp R, Hersberger KE, Lampert ML. Inappropriate prescribing: a systematic overview of published assessment tools. *Eur J Clin Pharmacol*. 2014;70(1):1-11. doi:10.1007/s00228-013-1575-8
58. Burt J, Elmore N, Campbell SM, Rodgers S, Avery AJ, Payne RA. Developing a measure of polypharmacy appropriateness in primary care: systematic review and expert consensus study. *BMC Med*. 2018;16(1):91. doi:10.1186/s12916-018-1078-7
59. George J, Phun Y-T, Bailey MJ, Kong DCM, Stewart K. Development and Validation of the Medication Regimen Complexity Index. *Ann Pharmacother*. 2004;38(9):1369-1376. doi:10.1345/aph.1D479
60. Basger BJ, Chen TF, Moles RJ. Validation of prescribing appropriateness criteria for older Australians using the RAND/UCLA appropriateness method. *BMJ Open*. 2012;2(5):e001431.
61. Haque R. ARMOR: a tool to evaluate polypharmacy in elderly persons. *Ann Long-Term Care*. 2009;17(6):26-30.
62. Scott I, Anderson K, Freeman C. Review of structured guides for deprescribing. *Eur J Hosp Pharm Sci Pract*. 2017;24(1):51-57. doi:10.1136/ejhpharm-2015-000864
63. Cadogan CA, Ryan C, Hughes CM. Appropriate Polypharmacy and Medicine Safety: When Many is not Too Many. *Drug Saf*. 2016;39(2):109-116. doi:10.1007/s40264-

For peer review only

015-0378-5

64. Laroche M-L, Charmes J-P, Nouaille Y, Picard N, Merle L. Is inappropriate medication use a major cause of adverse drug reactions in the elderly? *Br J Clin Pharmacol*. 2007;63(2):177-186. doi:10.1111/j.1365-2125.2006.02831.x

65. Miller GC, Valenti L, Britt H, Bayram C. Drugs causing adverse events in patients aged 45 or older: a randomised survey of Australian general practice patients. *BMJ Open*. 2013;3(10):e003701. doi:10.1136/bmjopen-2013-003701

66. Brown JD, Hutchison LC, Li C, Painter JT, Martin BC. Predictive Validity of the Beers and Screening Tool of Older Persons' Potentially Inappropriate Prescriptions (STOPP) Criteria to Detect Adverse Drug Events, Hospitalizations, and Emergency Department Visits in the United States. *J Am Geriatr Soc*. 2016;64(1):22-30. doi:10.1111/jgs.13884

67. Cahir C, Moriarty F, Teljeur C, Fahey T, Bennett K. Potentially inappropriate prescribing and vulnerability and hospitalization in older community-dwelling patients. *Ann Pharmacother*. 2014;48(12):1546-1554. doi:10.1177/1060028014552821

68. Rogero-Blanco E, Del-Cura-Gonzalez I, Aza-Pascual-Salcedo M, et al. Drug interactions detected by a computer-assisted prescription system in primary care patients in Spain: MULTIPAP study. MULTIPAP group, ed. *Eur J Gen Pract*. 2021;27(1):90-96. http://ovidsp.ovid.com/ovidweb.cgi?T=JS&PAGE=reference&D=med20&NEWS=N&AN=33982632.

69. Del Cura-González I, López-Rodríguez JA, Leiva-Fernández F, et al. How to Improve Healthcare for Patients with Multimorbidity and Polypharmacy in Primary Care: A Pragmatic Cluster-Randomized Clinical Trial of the MULTIPAP Intervention. *J Pers Med*. 2022;12(5). doi:10.3390/jpm12050752

70. Khatter A, Moriarty F, Ashworth M, Durbaba S, Redmond P. Prevalence and predictors of potentially inappropriate prescribing in middle-aged adults: a repeated cross-sectional study. *Br J Gen Pract*. 2021;71(708):e491-e497. doi:https://dx.doi.org/10.3399/BJGP.2020.1048 PT - Journal Article

71. Cooper JA, Moriarty F, Ryan C, et al. Potentially inappropriate prescribing in two populations with differing socio-economic profiles: a cross-sectional database study using the PROMPT criteria. *Eur J Clin Pharmacol*. 2016;72(5):583-591. doi:https://dx.doi.org/10.1007/s00228-015-2003-z PT - Journal Article, Research Support, Non-U.S. Gov't

72. Johansson T, Abuzahra ME, Keller S, et al. Impact of strategies to reduce polypharmacy on clinically relevant endpoints: a systematic review and meta-analysis. *Br J Clin Pharmacol*. 2016;82(2):532-548. doi:10.1111/bcp.12959

73. Reeve E, Gnjidic D, Long J, Hilmer S. A systematic review of the emerging definition of "deprescribing" with network analysis: implications for future research and clinical practice. *Br J Clin Pharmacol*. 2015;80(6):1254-1268. doi:10.1111/bcp.12732

74. Reeve J, Maden M, Hill R, et al. Deprescribing medicines in older people living with multimorbidity and polypharmacy: the TAILOR evidence synthesis. *Health Technol Assess*. 2022;26(32):1-148. doi:10.3310/AAFO2475

75. Seppala LJ, Kamkar N, van Poelgeest EP, et al. Medication reviews and deprescribing as a single intervention in falls prevention: a systematic review and meta-analysis. *Age Ageing*. 2022;51(9):afac191. doi:10.1093/ageing/afac191

76. Kua C-H, Mak VSL, Huey Lee SW. Health Outcomes of Deprescribing Interventions Among Older Residents in Nursing Homes: A Systematic Review and Meta-analysis. *J*

For peer review only

Am Med Dir Assoc. 2019;20(3):362-372.e11. doi:https://doi.org/10.1016/j.jamda.2018.10.026

77. Shrestha S, Poudel A, Cardona M, Steadman KJ, Nissen LM. Impact of deprescribing dual-purpose medications on patient-related outcomes for older adults near end-of-life: a systematic review and meta-analysis. *Ther Adv drug Saf*. 2021;12:20420986211052344. doi:10.1177/20420986211052343
78. Soler O, Barreto JOM. Community-Level Pharmaceutical Interventions to Reduce the Risks of Polypharmacy in the Elderly: Overview of Systematic Reviews and Economic Evaluations. *Front Pharmacol*. 2019;10. doi:10.3389/fphar.2019.00302
79. Anderson LJ, Schnipper JL, Nuckols TK, et al. A systematic overview of systematic reviews evaluating interventions addressing polypharmacy. *Am J Heal Pharm*. 2019;76(21):1777-1787. doi:10.1093/ajhp/zxz196
80. Dills H, Shah K, Messinger-Rapport B, Bradford K, Syed Q. Deprescribing Medications for Chronic Diseases Management in Primary Care Settings: A Systematic Review of Randomized Controlled Trials. *J Am Med Dir Assoc*. 2018;19(11):923-935.e2. doi:10.1016/j.jamda.2018.06.021
81. Ulley J, Harrop D, Ali A, Alton S, Fowler Davis S. Deprescribing interventions and their impact on medication adherence in community-dwelling older adults with polypharmacy: a systematic review. *BMC Geriatr*. 2019;19(1):15. doi:10.1186/s12877-019-1031-4
82. Ali MU, Sherifali D, Fitzpatrick-Lewis D, et al. Interventions to address polypharmacy in older adults living with multimorbidity. *Can Fam Physician*. 2022;68(7):e215 LP-e226. doi:10.46747/cfp.6807e215
83. Riordan DO, Walsh KA, Galvin R, Sinnott C, Kearney PM, Byrne S. The effect of pharmacist-led interventions in optimising prescribing in older adults in primary care: A systematic review. *SAGE open Med*. 2016;4:2050312116652568. doi:10.1177/2050312116652568
84. Hill-Taylor B, Walsh KA, Stewart S, Hayden J, Byrne S, Sketris IS. Effectiveness of the STOPP/START (Screening Tool of Older Persons' potentially inappropriate Prescriptions/Screening Tool to Alert doctors to the Right Treatment) criteria: systematic review and meta-analysis of randomized controlled studies. *J Clin Pharm Ther*. 2016;41(2):158-169. doi:10.1111/jcpt.12372
85. Tasai S, Kumpat N, Dilokthornsakul P, Chaiyakunapruk N, Saini B, Dhippayom T. Impact of Medication Reviews Delivered by Community Pharmacist to Elderly Patients on Polypharmacy: A Meta-analysis of Randomized Controlled Trials. *J Patient Saf*. 2021;17(4).
86. Laberge M, Sirois C, Lunghi C, et al. Economic Evaluations of Interventions to Optimize Medication Use in Older Adults with Polypharmacy and Multimorbidity: A Systematic Review. *Clin Interv Aging*. 2021;16:767-779. doi:10.2147/CIA.S304074
87. Ibrahim K, Cox NJ, Stevenson JM, Lim S, Fraser SDS, Roberts HC. A systematic review of the evidence for deprescribing interventions among older people living with frailty. *BMC Geriatr*. 2021;21(1):258. doi:10.1186/s12877-021-02208-8
88. Lee JQ, Ying K, Lun P, et al. Intervention elements to reduce inappropriate prescribing for older adults with multimorbidity receiving outpatient care: a scoping review. *BMJ Open*. 2020;10(8):e039543. doi:10.1136/bmjopen-2020-039543
89. Jager C, Steinhauser J, Freund T, Kuse S, Szecsenyi J, Wensing M. A tailored programme to implement recommendations for multimorbid patients with

For peer review only

polypharmacy in primary care practices-process evaluation of a cluster randomized trial. *Implement Sci*. 2017;12(1):31. doi:https://dx.doi.org/10.1186/s13012-017-0559-y PT - Journal Article, Randomized Controlled Trial, Research Support, Non-U.S. Gov't

90. Michiels-Corsten M, Gerlach N, Schleef T, Junius-Walker U, Donner-Banzhoff N, Viniol A. Generic instruments for drug discontinuation in primary care: A systematic review. *Br J Clin Pharmacol*. 2020;86(7):1251-1266.

91. Zechmann S, Senn O, Valeri F, et al. Effect of a patient-centred deprescribing procedure in older multimorbid patients in Swiss primary care - A cluster-randomised clinical trial. *BMC Geriatr*. 2020;20(1):471. doi:10.1186/s12877-020-01870-8

92. San-Jose A, Perez-Bocanegra C, Agusti A, et al. Integrated health intervention on polypharmacy and inappropriate prescribing in elderly people with multimorbidity: Results at the end of the intervention and at 6 months after the intervention. *Med Clin (Barc)*. 2021;156(6):263-269. doi:https://dx.doi.org/10.1016/j.medcli.2020.04.030 PT - Journal Article

93. Cardwell K, Smith SM, Clyne B, et al. Evaluation of the General Practice Pharmacist (GPP) intervention to optimise prescribing in Irish primary care: a non-randomised pilot study. *BMJ Open*. 2020;10(6):e035087.

94. Alaa Eddine N, Schreiber J, El-Yazbi AF, Shmaytilli H, Amin MEK. A pharmacist-led medication review service with a deprescribing focus guided by implementation science. *Front Pharmacol*. 2023;14:1097238. doi:10.3389/fphar.2023.1097238

95. Benson M, Murphy D, Hall L, Vande Kamp P, Cook DJ. Medication management for complex patients in primary care: application of a remote, asynchronous clinical pharmacist model. *Postgrad Med*. 2021;133(7):784-790. doi:https://dx.doi.org/10.1080/00325481.2021.1934492 PT - Journal Article, Observational Study

96. Uhl MC, Muth C, Gerlach FM, Schoch G-G, Muller BS. Patient-perceived barriers and facilitators to the implementation of a medication review in primary care: a qualitative thematic analysis. *BMC Fam Pract*. 2018;19(1):3. doi:https://dx.doi.org/10.1186/s12875-017-0707-0 PT - Journal Article, Research Support, Non-U.S. Gov't

97. Bell HT, Granas AG, Enmarker I, Omli R, Steinsbekk A. Nurses' and pharmacists' learning experiences from participating in interprofessional medication reviews for elderly in primary health care - a qualitative study. *BMC Fam Pract*. 2017;18(1):30. doi:https://dx.doi.org/10.1186/s12875-017-0598-0 PT - Journal Article, Multicenter Study

98. Koberlein-Neu J, Mennemann H, Hamacher S, et al. Interprofessional Medication Management in Patients With Multiple Morbidities. *Dtsch Arztebl Int*. 2016;113(44):741-748.

99. Molist-Brunet N, Sevilla-Sanchez D, Puigoriol-Juvanteny E, Barneto-Soto M, Gonzalez-Bueno J, Espaulella-Panicot J. Improving individualized prescription in patients with multimorbidity through medication review. *BMC Geriatr*. 2022;22(1):417.

100. McCarthy C, Clyne B, Boland F, et al. GP-delivered medication review of polypharmacy, deprescribing, and patient priorities in older people with multimorbidity in Irish primary care (SPPiRE Study): A cluster randomised controlled trial. team SppS, ed. *PLoS Med*. 2022;19(1):e1003862.

101. McCarthy C, Pericin I, Smith SM, et al. Patient and general practitioner experiences of implementing a medication review intervention in older people with multimorbidity:

For peer review only

Process evaluation of the SPPiRE trial. *Heal Expect*. 2022;25(6):3225-3237. doi:https://doi.org/10.1111/hex.13630

102. Schafer I, Kaduszkiewicz H, Mellert C, et al. Narrative medicine-based intervention in primary care to reduce polypharmacy: results from the cluster-randomised controlled trial MultiCare AGENDA. *BMJ Open*. 2018;8(1):e017653.
103. McCarthy C, Moriarty F, Wallace E, Smith SM. The evolution of an evidence based intervention designed to improve prescribing and reduce polypharmacy in older people with multimorbidity and significant polypharmacy in primary care (SPPiRE). *J Comorbidity*. 2020;10:2235042X20946243. doi:10.1177/2235042X20946243
104. Mann N-K, Schmiedl S, Mortsiefer A, et al. Development of a deprescribing manual for frail older people for use in the COFRAIL study and in primary care. *Ther Adv drug Saf*. 2022;13:20420986221122684. doi:10.1177/20420986221122684
105. Mangin D, Risdon C, Lamarche L, et al. "I think this medicine actually killed my wife": patient and family perspectives on shared decision-making to optimize medications and safety. *Ther Adv drug Saf*. 2019;10:2042098619838796. doi:10.1177/2042098619838796
106. Schöpf AC, von Hirschhausen M, Farin E, Maun A. Elderly patients' and GPs' perspectives of patient–GP communication concerning polypharmacy: a qualitative interview study. *Prim Health Care Res Dev*. 2018;19(4):355-364. doi:DOI: 10.1017/S1463423617000883
107. Knowles S, Hays R, Senra H, et al. Empowering people to help speak up about safety in primary care: Using codesign to involve patients and professionals in developing new interventions for patients with multimorbidity. *Health Expect*. 2018;21(2):539-548. doi:https://dx.doi.org/10.1111/hex.12648 PT - Journal Article, Research Support, Non-U.S. Gov't
108. Noel PH, Frueh BC, Larme AC, Pugh JA. Collaborative care needs and preferences of primary care patients with multimorbidity. *Health Expect*. 2005;8(1):54-63. http://ovidsp.ovid.com/ovidweb.cgi?T=JS&PAGE=reference&D=med6&NEWS=N&AN=15713171.
109. Maidment I, Lawson S, Wong G, et al. Towards an understanding of the burdens of medication management affecting older people: the MEMORABLE realist synthesis. *BMC Geriatr*. 2020;20(1):183. doi:10.1186/s12877-020-01568-x
110. Reeve E, Low L-F, Hilmer SN. Beliefs and attitudes of older adults and carers about deprescribing of medications: a qualitative focus group study. *Br J Gen Pract J R Coll Gen Pract*. 2016;66(649):e552-60. doi:10.3399/bjgp16X685669
111. Zechmann S, Trueb C, Valeri F, Streit S, Senn O, Neuner-Jehle S. Barriers and enablers for deprescribing among older, multimorbid patients with polypharmacy: an explorative study from Switzerland. *BMC Fam Pract*. 2019;20(1):64. doi:10.1186/s12875-019-0953-4
112. Reeve E, Shakib S, Hendrix I, Roberts MS, Wiese MD. Review of deprescribing processes and development of an evidence-based, patient-centred deprescribing process. *Br J Clin Pharmacol*. 2014;78(4):738-747. doi:10.1111/bcp.12386
113. Lozano-Hernandez CM, Lopez-Rodriguez JA, Leiva-Fernandez F, et al. Social support, social context and nonadherence to treatment in young senior patients with multimorbidity and polypharmacy followed-up in primary care. MULTIPAP Study. *PLoS One*. 2020;15(6):e0235148.
114. Ose D, Mahler C, Vogel I, Ludt S, Szecsenyi J, Freund T. Let's talk about medication:

For peer review only

For peer review only - http://bmjopen.bmj.com/site/about/guidelines.xhtml

concordance in rating medication adherence among multimorbid patients and their general practitioners. *Patient Prefer Adherence*. 2012;6:839-845. doi:10.2147/PPA.S35498

115. Weir KR, Naganathan V, Carter SM, et al. The role of older patients' goals in GP decision-making about medicines: a qualitative study. *BMC Fam Pract*. 2021;22(1):13.

116. Junius-Walker U, Viniol A, Michiels-Corsten M, Gerlach N, Donner-Banzhoff N, Schleef T. MediQuit, an Electronic Deprescribing Tool for Patients on Polypharmacy: Results of a Feasibility Study in German General Practice. *Drugs Aging*. 2021;38(8):725-733. doi:10.1007/s40266-021-00861-7

117. Neuner-Jehle S, Zechmann S, Grundmann Maissen D, Rosemann T, Senn O. Patient-provider concordance in the perception of illness and disease: a cross-sectional study among multimorbid patients and their general practitioners in Switzerland. *Patient Prefer Adherence*. 2017;11:1451-1458. doi:10.2147/PPA.S137388

118. Michiels-Corsten M, Gerlach N, Junius-Walker U, Schleef T, Donner-Banzhoff N, Viniol A. MediQuit - an electronic deprescribing tool: a pilot study in German primary care; GPs' and patients' perspectives. *BMC Prim care*. 2022;23(1):252. http://ovidsp.ovid.com/ovidweb.cgi?T=JS&PAGE=reference&D=med22&NEWS=N&AN=36162994.

119. Page AT, Clifford RM, Potter K, Schwartz D, Etherton-Beer CD. The feasibility and effect of deprescribing in older adults on mortality and health: a systematic review and meta-analysis. *Br J Clin Pharmacol*. 2016;82(3):583-623. doi:10.1111/bcp.12975

120. Verma A, Saha S, Jarl J, Conlon E, McGuinness B, Trépel D. An Overview of Systematic Reviews and Meta-Analyses on the Effect of Medication Interventions Targeting Polypharmacy for Frail Older Adults. *J Clin Med*. 2023;12(4). doi:10.3390/jcm12041379

121. Anderson LJ, Schnipper JL, Nuckols TK, et al. Effect of medication reconciliation interventions on outcomes: A systematic overview of systematic reviews. *Am J Heal Pharm*. 2019;76(24):2028-2040. doi:10.1093/ajhp/zxz236

122. Rieckert A, Reeves D, Altiner A, et al. Use of an electronic decision support tool to reduce polypharmacy in elderly people with chronic diseases: cluster randomised controlled trial. *BMJ*. 2020;369:m1822. doi:10.1136/bmj.m1822

123. Muth C, Uhlmann L, Haefeli WE, et al. Effectiveness of a complex intervention on Prioritising Multimedication in Multimorbidity (PRIMUM) in primary care: results of a pragmatic cluster randomised controlled trial. *BMJ Open*. 2018;8(2):e017740. doi:https://dx.doi.org/10.1136/bmjopen-2017-017740 PT - Journal Article, Pragmatic Clinical Trial, Research Support, Non-U.S. Gov't

124. Hasan Ibrahim AS, Barry HE, Hughes CM. A systematic review of general practice-based pharmacists' services to optimize medicines management in older people with multimorbidity and polypharmacy. *Fam Pract*. 2021;38(4):509-523.

125. Croke A, Cardwell K, Clyne B, Moriarty F, McCullagh L, Smith SM. The effectiveness and cost of integrating pharmacists within general practice to optimize prescribing and health outcomes in primary care patients with polypharmacy: a systematic review. *BMC Prim care*. 2023;24(1):41. doi:10.1186/s12875-022-01952-z

126. Collier A, Balmer D, Dai L, Hikaka J, Bloomfield K, Boyd M. Older people, medication safety, and the role of the community pharmacist: a longitudinal ethnographic study. *J Pharm Pract Res*. 2023;53(1):18-25. doi:https://doi.org/10.1002/jppr.1839

127. Fudge N, Swinglehurst D. 'It's all about patient safety': an ethnographic study of how

For peer review only

pharmacy staff construct medicines safety in the context of polypharmacy. *BMJ Open*. 2021;11(2):e042504. doi:10.1136/bmjopen-2020-042504

128. Hansen CR, O'Mahony D, Kearney PM, et al. Identification of behaviour change techniques in deprescribing interventions: a systematic review and meta-analysis. *Br J Clin Pharmacol*. 2018;84(12):2716-2728. doi:10.1111/bcp.13742
129. Gorman A, Rankin A, Hughes C, O'Dwyer M, Ryan C. Theoretically derived interventions aimed at improving appropriate polypharmacy in primary care: A systematic review. *Explor Res Clin Soc Pharm*. 2022;7:100166. doi:https://doi.org/10.1016/j.rcsop.2022.100166
130. Rankin A, Gorman A, Cole J, et al. An external pilot cluster randomised controlled trial of a theory-based intervention to improve appropriate polypharmacy in older people in primary care (PolyPrime). *Pilot Feasibility Stud*. 2022;8(1):203. doi:10.1186/s40814-022-01161-6
131. Kirwan C, Hynes L, Hart N, et al. The multimorbidity collaborative medication review and decision making (MyComrade) study: a pilot cluster randomised trial in two healthcare systems. *Pilot Feasibility Stud*. 2022;8(1):225.
132. Cadogan CA, Ryan C, Francis JJ, et al. Improving appropriate polypharmacy for older people in primary care: selecting components of an evidence-based intervention to target prescribing and dispensing. *Implement Sci*. 2015;10(1):161. doi:10.1186/s13012-015-0349-3
133. Khezrian M, McNeil CJ, Murray AD, Myint PK. An overview of prevalence, determinants and health outcomes of polypharmacy. *Ther Adv drug Saf*. 2020;11:2042098620933741.
134. Ong SM, Lim YMF, Sivasampu S, Khoo EM. Variation of polypharmacy in older primary care attenders occurs at prescriber level. *BMC Geriatr*. 2018;18(1):59. doi:https://dx.doi.org/10.1186/s12877-018-0750-2 PT - Journal Article, Research Support, Non-U.S. Gov't
135. Sawan M, Reeve E, Turner J, et al. A systems approach to identifying the challenges of implementing deprescribing in older adults across different health-care settings and countries: a narrative review. *Expert Rev Clin Pharmacol*. 2020;13(3):233-245. doi:10.1080/17512433.2020.1730812
136. Doherty AJ, Boland P, Reed J, et al. Barriers and facilitators to deprescribing in primary care: a systematic review. *BJGP Open*. 2020;4(3):bjgpopen20X101096. doi:10.3399/bjgpopen20X101096
137. Wallis KA, Andrews A, Henderson M. Swimming Against the Tide: Primary Care Physicians' Views on Deprescribing in Everyday Practice. *Ann Fam Med*. 2017;15(4):341-346. doi:10.1370/afm.2094
138. Anderson K, Stowasser D, Freeman C, Scott I. Prescriber barriers and enablers to minimising potentially inappropriate medications in adults: a systematic review and thematic synthesis. *BMJ Open*. 2014;4(12):e006544. doi:10.1136/bmjopen-2014-006544
139. Bujold M, Pluye P, Legare F. Decision-making and related outcomes of patients with complex care needs in primary care settings: a systematic literature review with a case-based qualitative synthesis. *BMC Prim care*. 2022;23(1):279.
140. Rozsnyai Z, Jungo KT, Reeve E, et al. What do older adults with multimorbidity and polypharmacy think about deprescribing? The LESS study - a primary care-based survey. *BMC Geriatr*. 2020;20(1):435. doi:https://dx.doi.org/10.1186/s12877-020-

For peer review only - http://bmjopen.bmj.com/site/about/guidelines.xhtml

01843-x PT - Journal Article, Research Support, Non-U.S. Gov't

141. Swinglehurst D, Hogger L, Fudge N. Negotiating the polypharmacy paradox: a video-reflexive ethnography study of polypharmacy and its practices in primary care. *BMJ Qual Saf*. 2023;32(3):150-159. doi:https://dx.doi.org/10.1136/bmjqs-2022-014963 PT - Journal Article
142. Swinglehurst D, Fudge N. Organising polypharmacy: unpacking medicines, unpacking meanings—an ethnographic study. *BMJ Open*. 2021;11(8):e049218. doi:10.1136/bmjopen-2021-049218
143. Hasler S, Senn O, Rosemann T, Neuner-Jehle S. Effect of a patient-centered drug review on polypharmacy in primary care patients: study protocol for a cluster-randomized controlled trial. *Trials*. 2015;16:380. doi:https://dx.doi.org/10.1186/s13063-015-0915-7 PT - Journal Article, Multicenter Study, Randomized Controlled Trial, Research Support, Non-U.S. Gov't
144. Reeve E, To J, Hendrix I, Shakib S, Roberts MS, Wiese MD. Patient barriers to and enablers of deprescribing: a systematic review. *Drugs Aging*. 2013;30(10):793-807. doi:10.1007/s40266-013-0106-8
145. Cairo Notari S, Sader J, Caire Fon N, et al. Understanding GPs' clinical reasoning processes involved in managing patients suffering from multimorbidity: A systematic review of qualitative and quantitative research. *Int J Clin Pract*. 2021;75(9):e14187. doi:10.1111/ijcp.14187
146. Sinnott C, Hugh SM, Boyce MB, Bradley CP. What to give the patient who has everything? A qualitative study of prescribing for multimorbidity in primary care. *Br J Gen Pract*. 2015;65(632):e184-91. doi:https://dx.doi.org/10.3399/bjgp15X684001 PT - Journal Article, Research Support, Non-U.S. Gov't
147. Carrier H, Zaytseva A, Bocquier A, et al. GPs' management of polypharmacy and therapeutic dilemma in patients with multimorbidity: a cross-sectional survey of GPs in France. *Br J Gen Pract*. 2019;69(681):e270-e278. doi:https://dx.doi.org/10.3399/bjgp19X701801 PT - Journal Article
148. Anderson K, Foster M, Freeman C, Luetsch K, Scott I. Negotiating "Unmeasurable Harm and Benefit": Perspectives of General Practitioners and Consultant Pharmacists on Deprescribing in the Primary Care Setting. *Qual Health Res*. 2017;27(13):1936-1947.
149. Mc Namara KP, Breken BD, Alzubaidi HT, et al. Health professional perspectives on the management of multimorbidity and polypharmacy for older patients in Australia. *Age Ageing*. 2017;46(2):291-299. http://ovidsp.ovid.com/ovidweb.cgi?T=JS&PAGE=reference&D=med14&NEWS=N&AN=27836856.
150. Sinnige J, Korevaar JC, van Lieshout J, Westert GP, Schellevis FG, Braspenning JC. Medication management strategy for older people with polypharmacy in general practice: a qualitative study on prescribing behaviour in primary care. *Br J Gen Pract*. 2016;66(649):e540-51. doi:https://dx.doi.org/10.3399/bjgp16X685681 PT - Journal Article
151. Turner JP, Edwards S, Stanners M, Shakib S, Bell JS. What factors are important for deprescribing in Australian long-term care facilities? Perspectives of residents and health professionals. *BMJ Open*. 2016;6(3):e009781. doi:https://dx.doi.org/10.1136/bmjopen-2015-009781 PT - Journal Article, Research Support, Non-U.S. Gov't

For peer review only

152. Smith SM, O'Kelly S, O'Dowd T. GPs' and pharmacists' experiences of managing multimorbidity: a "Pandora's box". *Br J Gen Pract*. 2010;60(576):285-294.
153. Laursen J, Kornholt J, Betzer C, Petersen TS, Christensen MB. General Practitioners' Barriers Toward Medication Reviews in Polymedicated Multimorbid Patients: How can a Focus on the Pharmacotherapy in an Outpatient Clinic Support GPs? *Heal Serv Res Manag Epidemiol*. 2018;5:2333392818792169. doi:10.1177/2333392818792169
154. Tsoi CS, Chow JY, Choi KS, et al. Medical characteristics of the oldest old: retrospective chart review of patients aged 85+ in an academic primary care centre. *BMC Res Notes*. 2014;7:340.
155. Tan YY, Papez V, Chang WH, Mueller SH, Denaxas S, Lai AG. Comparing clinical trial population representativeness to real-world populations: an external validity analysis encompassing 43 895 trials and 5 685 738 individuals across 989 unique drugs and 286 conditions in England. *Lancet Heal Longev*. 2022;3(10):e674-e689. doi:10.1016/S2666-7568(22)00186-6
156. Wallace E, Salisbury C, Guthrie B, Lewis C, Fahey T, Smith SM. Managing patients with multimorbidity in primary care. *BMJ Br Med J*. 2015;350:h176. doi:10.1136/bmj.h176
157. Scott IA, Anderson K, Freeman CR, Stowasser DA. First do no harm: a real need to deprescribe in older patients. *Med J Aust*. 2014;201(7):390-392. doi:10.5694/mja14.00146

For peer review only

For peer review only - http://bmjopen.bmj.com/site/about/guidelines.xhtml

List of figures and supplementary files.

Figure 1: A PRIMSA Flow Diagram illustrating search results

Supplementary file 1: Descriptions of included papers in the synthesis

For peer review only

Modified PRISMA flow diagram for scoping search

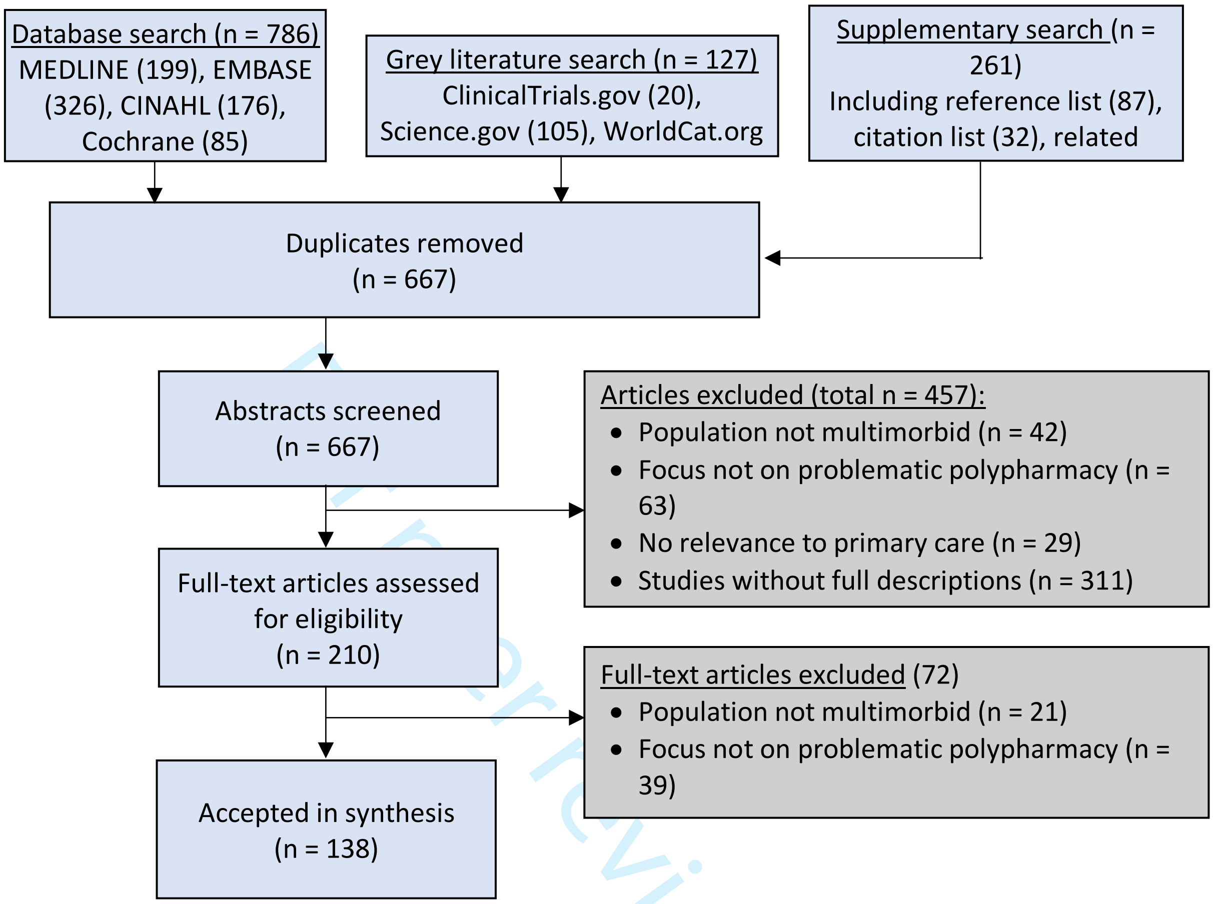

For peer review only

Author & Year	Title	Country	Publication type	Key Polypharmacy definitions	Population	Intervention (if applicable)	Key findings/ conclusions
Ali et al. 2022	Interventions to address polypharmacy in older adults living with multimorbidity.	Canada	Review of reviews	• Concurrent use of multiple medications<br>• ≥5 chronic medicines	Older adults (all 64+ years) with chronic conditions taking 5 or more medication across 5 reviews	Any polypharmacy intervention in the primary care setting	• Polypharmacy interventions are associated with reductions in potentially inappropriate prescribing and improvements in medication adherence.<br>• However, there is limited evidence of their effectiveness for clinical and intermediate outcomes.
Anderson et al. 2019	A systematic overview of systematic reviews evaluating interventions addressing polypharmacy.	USA	Review of reviews	• Use of multiple medications daily<br>• Appropriate and inappropriate polypharmacy	Mainly older adults (≥ 65 years) from 6 systematic reviews (in a range of primary care, community, nursing home and secondary care settings)	Any intervention addressing polypharmacy	• Despite the low quality of evidence in the underlying primary studies, there was evidence polypharmacy interventions improved medication appropriateness.<br>• However, there was no consistent evidence of any impact on downstream patient-centred outcomes such as healthcare utilization, morbidity, or mortality.
Anderson et al. 2019	Effect of medication reconciliation interventions on outcomes: A systematic overview of systematic reviews.	USA	Review of reviews	• Many medications<br>• Focus on medication discrepancies	Adult or paediatric populations	Interventions that included medication reconciliation as a core part	• An overview of systematic reviews of medication reconciliation interventions found 9 high-quality systematic reviews.<br>• A minority of those reviews' conclusions were consistent with medication reconciliation alone having a measurable impact, and such conclusions were almost all based on very low-quality evidence.
Soler et al. 2019	Community-Level Pharmaceutical Interventions to Reduce the Risks of Polypharmacy in the Elderly: Overview of Systematic Reviews and Economic Evaluations	Brazil	Review of reviews	• Prescription of multiple drugs to the individual<br>• Adequate polypharmacy and inadequate polypharmacy	Mainly older adults (≥ 65 years) from 13 systematic reviews and 3 economic analyses (range of primary/secondary care settings)	Any pharmaceutical intervention to reduce risks of polypharmacy	• There is evidence of improvement in clinical, epidemiological, humanistic, and economic outcomes for various types of community-level pharmaceutical interventions,<br>• Differences in observed outcomes may be due to study designs, primary study sample sizes, risk of bias, difficulty in aggregating data, heterogeneity of indicators and quality of evidence
Page et al. 2016	The feasibility and effect of deprescribing in older adults on	Australia	Review and meta-analysis	• Use of many medications	34 143 older adults (≥65 years,	Any deprescribing intervention	• Although nonrandomized data suggested that deprescribing reduces mortality, deprescribing

For peer review only

	mortality and health: a systematic review and meta-analysis			• Focus on deprescribing	mean age 73.8) across 132 papers		• was not shown to alter mortality in randomized studies.<br>• Mortality was significantly reduced when applying patient-specific interventions in randomized studies.
Hill-Taylor et al. 2016	Effectiveness of the STOPP/START (Screening Tool of Older Persons' potentially inappropriate Prescriptions/Screening Tool to Alert doctors to the Right Treatment) criteria: systematic review and meta-analysis of randomized controlled studies	Canada	Review & meta-analysis	• Focus on potentially inappropriate prescribing	1925 older adults (≥65 years) from 4 RCTs across four countries (both acute and long-term care settings)	Interventions involving prospective application of the STOPP and/or START criteria on medication profiles	• Meta-analysis found that the STOPP criteria reduced PIM rates in all four studies, but study heterogeneity (I2 = 86·7%) prevented the calculation of a meaningful statistical summary.<br>• We found evidence that use of the criteria reduces falls, delirium episodes, hospital length-of-stay, care visits (primary and emergency) and medication costs, but no evidence of improvements in quality of life or mortality.
Johansson et al. 2016	Impact of strategies to reduce polypharmacy on clinically relevant endpoints: a systematic review and meta-analysis.	Austria	Review & meta-analysis	• ≥4 or ≥5 drugs<br>• Use of potentially inappropriate medication	10 980 older adults (65+ years) across 25 controlled trials (multiple settings)	Any intervention aimed at reducing inappropriate polypharmacy	• There is no convincing evidence that the strategies assessed in the present review are effective in reducing polypharmacy<br>• These strategies to reduce polypharmacy had no effect on all-cause mortality (odds ratio 1.02; 95% confidence interval 0.84, 1.23). Only single studies found improvements, in terms of reducing the number of hospital admissions, in favour of the intervention group.
Kua et al. 2019	Health outcomes of deprescribing interventions among older residents in nursing homes: a systematic review and meta-analysis.	Malaysia	Review & meta-analysis	• Concurrent use of multiple medications<br>• Potentially inappropriate medications<br>• Focus on deprescribing	Older Residents (≥ 60 years) in Nursing Homes across 41 RCTs	Any deprescribing intervention (defined as either medication discontinuation, substitution, or reduction)	• Deprescribing interventions significantly reduced the number of residents with potentially inappropriate medications by 59%<br>• Compared to other deprescribing interventions, medication review–directed deprescribing had significant benefits on older residents in nursing homes. Further research is required to elicit other clinical benefits of medication review–directed deprescribing practice.
Seppala et al. 2022	Medication reviews and deprescribing as a single intervention in falls prevention: a	Netherlands	Review & meta-analysis	• Use of multiple medications<br>• Focus on deprescribing	Older adults (aged ≥ 60 years) across 49 studies (separate analyses for	Any medication review or deprescribing intervention as a single	• In meta-analyses, no significant associations between medication reviews and fall outcomes were found in any of the settings.

For peer review only - http://bmjopen.bmj.com/site/about/guidelines.xhtml

	systematic review and meta-analysis.				community, hospital, and long-term care settings)	intervention in falls prevention	• There was a trend for a lower number of fallers in the meta-analysis assessing medication reviews in long-term care.<br>• In a frail subgroup, medication review might be effective even as a single intervention.
Shrestha et al. 2021	Impact of deprescribing dual-purpose medications on patient-related outcomes for older adults near end-of-life: a systematic review and meta-analysis.	Australia	Review & meta-analysis	• Potentially inappropriate medications<br>• Focus on deprescribing	689 Older adults (mean 81.6-85.7 years) with at least one life-limiting illness and at least one potentially inappropriate dual-purpose medication (disease prevention and symptom control) across 5 studies	Any deprescribing intervention	• The deprescribing of DPMs lowered the risk of mortality (risk ratio (RR) = 0.59, 95% confidence interval (CI) = 0.44-0.79) and referral to acute care facilities (RR = 0.40, 95% CI = 0.22-0.73), but did not have a significant impact on the risk of falls, non-vertebral fracture, emergency presentation, unplanned hospital admission, or general practitioner visits<br>• Insufficient good-quality studies powered to confirm a benefit in terms of quality of life, physical or cognitive function, health service utilisation and adverse events.
Tasai et al. 2021	Impact of Medication Reviews Delivered by Community Pharmacist to Elderly Patients on Polypharmacy: A Meta-analysis of Randomized Controlled Trials	Thailand	Review & meta-analysis	• ≥4 prescribed medications	4633 older adults (65+ years) across 4 studies	Interventions incorporating medication reviews delivered by community pharmacist	• When compared with usual care, medication reviews provided by community pharmacist significantly reduced risk of ED visits (risk ratio = 0.68; 95% CI = 0.48–0.96)
Alldred et al. 2016	Interventions to optimise prescribing for older people in care homes.	UK	Review	• ≥4 regular medicines<br>• Focus on appropriate & inappropriate polypharmacy (Beers, STOPP/START, MAI)	10,953 older residents (≥ 65 years) in 355 care homes in 10 countries.	Any intervention to optimise overall prescribing	• Could not draw robust conclusions from the evidence due to variability in design, outcomes and results<br>• The interventions implemented in the studies in this review led to the identification and resolution of medication-related problems and improvements in medication appropriateness<br>• Evidence of a consistent effect on resident-related outcomes was not found.
Anderson et al. 2014	Prescriber barriers and enablers to minimising potentially	Australia	Review	• Use of multiple medications concurrently	Adults of all ages, with some focus on older adults	N/A	• Barriers and enablers to minimising PIMs emerged within four analytical themes: problem awareness; inertia secondary to lower perceived

	inappropriate medications in adults: a systematic review and thematic synthesis			• ≥5 prescription, over-the-counter or complementary medicines every day<br>• potentially inappropriate medications	with chronically prescribed potent ially inappropriate medications across 21 studies (18 in primary care, 2 in residential care, 1 in secondary care)		value proposition for ceasing versus continuing PIMs; self-efficacy regarding personal ability to alter prescribing; and feasibility of altering prescribing in routine care given external constraints.<br>• The first 3 themes are intrinsic to the prescriber (eg, beliefs, attitudes, knowledge, skills, behaviour) and the fourth is extrinsic (eg, patient, work setting, health system and cultural factors).
Cadogan et al. 2013	Appropriate Polypharmacy and Medicine Safety: When Many is not Too Many	UK (NI), Ireland	Review	• Use of multiple medicines<br>• Balancing 'too little' and 'too many'<br>• Focus on appropriate polypharmacy	Middle-aged and older population with multimorbidity	N/A	• Differentiating between 'many' drugs and 'too many' drugs is proving ever more complex.<br>• Previous assumptions that polypharmacy is synonymous with 'too many' medicines or inappropriate prescribing have been undermined because when patients' clinical context is taken into consideration, the prescribing of 'many' medicines can, in fact, be entirely appropriate.<br>• Conceptualising polypharmacy as a numerical threshold is unhelpful because it fails to consider that the appropriate number of medicines varies according to individual patients' clinical needs and may overlook the omission of potentially beneficial medications, which can equally pose risks to patients' safety and well-being<br>• Increased use of the term 'appropriate polypharmacy' could encourage greater consideration of the clinical context underlying prescribing, as well as increased acceptance that the prescribing of multiple medicines is 'potentially problematic rather than always inappropriate'.
Cairo Notari et al. 2021	Understanding GPs' clinical reasoning processes involved in managing patients suffering from multimorbidity: A systematic review of	Switzerl and	Review	• Multiple medicines and associated harmful effects<br>• Focus on deprescribing	Adults with multimorbidity (≥2 chronic conditions) across 32 studies	N/A	• In the absence of guidelines adapted to multimorbidity, there is no single correct plan, but competing priorities and unavoidable uncertainties.<br>• Thus, GPs have to consider and weigh multiple factors simultaneously. In the context of multimorbidity,

For peer review only

	qualitative and quantitative research						• GPs describe their reasoning as essentially intuitive and seem to perceive it as less accurate.<br>• These clinical reasoning processes are nevertheless more analytical than they might think and rooted in deep knowledge of the individual patient.
Cooper et al. 2015	Interventions to improve the appropriate use of polypharmacy in older people: a Cochrane systematic review	UK (Northern Ireland)	Review	• ≥4 regular medicines<br>• Focus on appropriate & inappropriate polypharmacy (Beers, STOPP/START, MAI)	Older adults (≥ 65 years) with ≥ 1 long-term condition who were receiving polypharmacy (≥ 4 regular medicines) from 12 studies (5 in community care) across 5 countries	All interventions improving appropriate polypharmacy.	• The included 12 interventions demonstrated improvements in appropriate polypharmacy based on reductions in inappropriate prescribing.<br>• However, it was unclear if interventions resulted in clinically significant improvements
Croke et al. 2023	The effectiveness and cost of integrating pharmacists within general practice to optimize prescribing and health outcomes in primary care patients with polypharmacy: a systematic review	Ireland	Review	• ≥5 regular medications<br>• Potentially inappropriate prescribing (any explicit/implicit criteria)	23,516 community dwelling adults (≥18 years) and in the primary care setting with polypharmacy (23 studies in 3 continents)	Any intervention involving pharmacist integration within General Practice (Including remote integration)	• Pharmacist integration probably reduced PIP and number of medications however, there was no clear effect on other patient outcomes<br>• While interventions in a small number of studies appeared to be cost-effective, further robust, well-designed cluster RCTs with economic evaluations are required to determine cost-effectiveness of pharmacist integration.
Dills et al. 2018	Deprescribing Medications for Chronic Diseases Management in Primary Care Settings: A Systematic Review of Randomized Controlled Trials	USA	Review	• Use of multiple prescription drugs<br>• Focus on deprescribing	Adults in a range of settings (primary care, assisted living, nursing home, outpatient and acute care settings only if medications for chronic disease were deprescribed	Deprescribing interventions involving chronic medical and mental health conditions managed by primary care professionals	• Deprescription may be successful and effective in select classes of drugs, with collaboration of clinical pharmacists for patient and provider education, and patient-specific drug recommendations, complemented by close clinical follow-up to detect early signs of exacerbation of chronic diseases.<br>• Deprescription may (1) require expensive intensive, ongoing interventions by clinical teams; (2) not lead to expected outcomes such as improved falls rate, cognition, and quality of life, or a lower admission rate; and (3) have unexpected adverse outcomes affecting patients' quality of life.

For peer review only

Doherty et al. 2020	Barriers and facilitators to deprescribing in primary care: a systematic review	UK	Review	• Multiple concurrent medications<br>• ≥4 of any type of medications<br>• Focus on deprescribing	Adults (aged ≥18 years) with multimorbidity and polypharmacy (≥2 long-term health conditions), across 40 studies from 14 countries	Any deprescribing intervention	A whole systems, patient-centred approach to safe deprescribing interventions is required, involving key decision-makers, healthcare professionals, patients, and carers.
Hasan Ibrahim et al. 2021	A systematic review of general practice-based pharmacists' services to optimize medicines management in older people with multimorbidity and polypharmacy	UK (Northern Ireland)	Review	• Concomitant use of ≥4 medicines<br>• Focus on medicines optimisation	Older adults (≥65 years) with multimorbidity (≥2 long-term conditions) across 7 studies in 5 countries	Practice-based pharmacists optimizing medicines management for older people with both multimorbidity and polypharmacy	• All studies employed pharmacist-led medication reviews accompanied by recommendations agreed and implemented by GPs.<br>• The limited available evidence suggested that, in collaboration with other practice team members, had mixed effects on outcomes focused on optimizing medicines management for older people.<br>• Most included studies were of poor quality and data to estimate the risk of bias often missing.
Heaton et al.	Person-centred medicines optimisation policy in England: an agenda for research on polypharmacy.	UK	Review	• Use of multiple medications<br>• 'Appropriate' and 'problematic' polypharmacy	Patients using multiple medicines in the UK	N/A	Reports varied in their inclusion of patient perspectives and person-centred care values, and in the extent to which they drew on evidence from research on patients' experiences of polypharmacy and medicines use.
Ibrahim et al. 2021	A systematic review of the evidence for deprescribing interventions among older people living with frailty.	UK	Review	• ≥5 regular medications<br>• Focus on deprescribing	657 older adults with frailty (mean age 79–87 years)	Any deprescribing medication review intervention accounting for at least 50% of changes	• All studies described medication-related outcomes and reported a reduction in potentially inappropriate medications and total number of medications per-patient.<br>• Feasibility of deprescribing in 4 studies which showed that 72–91% of recommendations made were implemented.<br>• 2 studies evaluated and reported acceptability of their interventions and further 2 described cost saving.<br>• But there was a paucity of research about the impact of deprescribing in older people living with frailty

For peer review only

Kaufmann et al. 2014	Inappropriate prescribing: a systematic overview of published assessment tools.	Switzerland	Review	• Use of multiple medicines<br>• Focus on appropriate and inappropriate prescribing	Adults (majority older adults) with potentially inappropriate prescribing	N/A	• Out of 46 tools to assess inappropriate prescribing. 28 (61%) were explicit, 8 (17%) were implicit and 10 (22%) used a mixed approach.<br>• 36 tools named older people as target patients and 10 (22%) tools did not specify the target age group.<br>• Only 9 tools (19.5%) focused on patients in ambulatory care and 6 (13%) were developed for use in long-term care. 27 (59%) tools did not specify the health care setting.
Khezrian et al. 2020	An overview of prevalence, determinants and health outcomes of polypharmacy.	UK (Scotland)	Review	• No generally accepted definition<br>• Major and minor polypharmacy criteria<br>• Numerical (e.g. ≥ 5 medications concurrently or excessive polypharmacy ≥ 10 medications concurrently)<br>• Descriptive methods (patients visiting multiple pharmacies to obtain medications' and 'use of additional medications to correct adverse effects)<br>• Appropriate vs inappropriate polypharmacy	Older adults	N/A	• Polypharmacy was most often defined in terms of the number of medications that are being taken by an individual at any given time.<br>• Our review showed that the prevalence of polypharmacy varied between 10% to as high as around 90% in different populations.<br>• Chronic conditions, demographics, socioeconomics and self-assessed health factors were independent predictors of polypharmacy.<br>• Polypharmacy was reported to be associated with various adverse outcomes after adjusting for health conditions.
Laberge et al. 2021	Economic Evaluations of Interventions to Optimize Medication Use in Older Adults with Polypharmacy and	Canada	Review	• Consumption of multiple medications simultaneously<br>• use of ≥5 medications	6375 older adults (≥65 years) with multimorbidity (2+ conditions) from 11 studies across 8 countries	Any intervention aimed at optimizing medication use	• Interventions were generally associated with a reduction in medication expenditure. The benefits of the intervention in terms of clinical outcomes remain limited.<br>• Five studies were cost-benefit analyses, which had a net benefit that was either null or positive.

For peer review only

	Multimorbidity: A Systematic Review			• potentially inappropriate medications and adverse drug reactions	(settings included primary care, nursing home, pharmacies, and secondary care)		• However, the quality of the studies was generally low.
Lee et al. 2020	Intervention elements to reduce inappropriate prescribing for older adults with multimorbidity receiving outpatient care: a scoping review.	Singapore	Review	• ≥5 medications daily<br>• Focus on potentially inappropriate prescribing	Older adults with multimorbidity	Interventions to reduce inappropriate prescribing	• 4 intervention elements were identified. An average of 2-3 to three elements were adopted in each intervention. The three most frequently adopted intervention elements were medication review (70%), training (26.3%) and tool/instrument(s) (22.5%).<br>• The 14 intervention elements were mapped onto five intervention functions: 'education', 'persuasion', 'training', 'environmental restructuring' and 'enablement'
Lee et al. 2022	Applicability of explicit potentially inappropriate medication lists to the Australian context: A systematic review.	Australia	Review	Focus on potentially inappropriate medications	Older adults prescribed potentially inappropriate medications	N/A	• Applicability of each explicit list ranged from 50-96% according to medications available in Australia and 25-83% according to medications available under subsidy.<br>• Pooling data from different lists may help to identify potentially inappropriate medications that may be applicable to local settings.
Lucchetti et al. 2017	Inappropriate prescribing in older persons: A systematic review of medications available in different criteria	Brazil	Review	Focus on potentially inappropriate medications	Older adults prescribed potentially inappropriate medications	N/A	• More than 85% used a Delphi method.<br>• There were 729 different medications/classes reported in all criteria.<br>• Diazepam was included in all 14 criteria followed by amitriptyline (13 criteria) and doxepin (12 criteria).<br>• Benzodiazepines, NSAIDs, antihistamines and antipsychotics were the most common drugs reported as potentially inappropriate for older persons.
Maidment et al. 2020	Towards an understanding of the burdens of medication management affecting older people: the MEMORABLE realist synthesis	UK	Review	• ≥5 medications<br>• Or if less – a complex regime	Older adults (≥ 60) years with multimorbidity (≥2 long term conditions) and polypharmacy	Medication management as a extended, complex process	• Older people and family carers often find medication management challenging and burdensome particularly for complex regimens.<br>• Practitioners need to be aware of this potential challenge, and work with older people and their carers to minimise the burden associated with medication management.

Mair et al. 2020	Addressing the Challenge of Polypharmacy.	UK (Scotland)	Review	• Use of multiple medications<br>• Appropriate and inappropriate polypharmacy	Older adults with multimorbidity and polypharmacy	N/A	Guidance is needed to support patients and clinicians in defining and achieving realistic goals of drug treatment, and system change is necessary to aid implementation.
Masnoon et al 2018	Tools for Assessment of the Appropriateness of Prescribing and Association with Patient-Related Outcomes: A Systematic Review.	Australia	Review	• Multiple medicines prescribed together<br>• Focus on appropriate and inappropriate prescribing	Adults (mainly ≥45 years) with multimorbidity and polypharmacy	N/A	• Out of the 42 tools, 78.6% (n = 33) provided guidance around stopping inappropriate medications, 28.6% (n = 12) around starting appropriate medications, 61.9% (n = 26) were explicit (criteria based) and 31.0% (n = 13) had been externally validated, with hospitalisation being the most commonly used patient-related outcome (n = 9, 21.4%).<br>• Less than 50% of available tools have been externally validated, limiting their use in clinical practice.
Masnoon et al.	What is polypharmacy? A systematic review of definitions.	Australia	Review	• 138 definitions of polypharmacy and associated terms were obtained.<br>• 111 numerical only definitions (80.4% of all definitions), 15 numerical definitions which incorporated a duration of therapy or healthcare setting (10.9%) and 12 descriptive definitions (8.7%).	Older adults with multimorbidity and polypharmacy	N/A	• Polypharmacy definitions were variable.<br>• Numerical definitions of polypharmacy did not account for specific comorbidities present and make it difficult to assess safety and appropriateness of therapy in the clinical setting.
Michiels-Corsten et al. 2020	Generic instruments for drug discontinuation in primary care: A systematic review	Germany	Review	• Concurrent use of several (mostly ≥5) long-term medications<br>• Potentially inappropriate medications<br>• Focus on discontinuation/ deprescribing	Adults in primary care	Any generic guiding instruments for drug discontinuation in patients with polypharmacy in the primary care setting.	• Instruments revealed diverging emphases on the stages of deprescribing, i.e. preparation, drug evaluation, decision-making and implementation. Accordingly, 3 types of instruments emerged: general frameworks, detailed drug assessment tools and comprehensive discontinuation guidelines.<br>• There is still a need for practical and user-friendly tools that support physicians in communicational aspects, visualise trade-offs and also enhance patient involvement.

For peer review only

Monégat et al, 2014	Polypharmacy: definitions, measurement and stakes involved	France	Review	Simultaneous, cumulative, and continuous polypharmacy) examined according to different thresholds – mainly ≥ 5 medications	69,324 older adults (aged 75+) from French GPs	N/A	• These definitions can used together, provide a broader overview of the use of medication.<br>• However, if polypharmacy is generally related to inappropriate prescribing, it is not sufficient on its own to identify them. Counting the number of medications does not allow distinguishing between those that are justified for a given pathology and those that are not.
Rankin et al. 2018	Interventions to improve the appropriate use of polypharmacy for older people.	UK (Ireland), include d trials from 12 countri es	Review	Polypharmacy (4 or more medicines), which used a validated tool to assess prescribing appropriateness	28,672 older adults (aged 65+) from 32 studies	Any intervention to improve appropriate polypharmacy	• Unclear whether interventions resulted in clinically significant improvements<br>• May be slightly beneficial in terms of reducing potential prescribing omissions; but this effect estimate is based on only two studies, which had serious limitations in terms of risk bias.
Reeve et al. 2022	Deprescribing medicines in older people living with multimorbidity and polypharmacy: the TAILOR evidence synthesis	UK	Review	• Concurrent use of multiple medicines in a single person<br>• Use of ≥5 medications<br>• Appropriate and problematic polypharmacy<br>• Focus on deprescribing	Adults (aged ≥ 50 years) with multimorbidity (≥2 conditions)	Deprescribing interventions	• Deprescribing is a complex intervention<br>• Need to integrate patient-centred and contextual factors into best practice models.<br>• 34 context-mechanism-outcome configurations describe the knowledge work of tailored prescribing under eight headings related to organisational, health-care professional and patient factors, and interventions to improve deprescribing.<br>• Robust tailored deprescribing requires attention to providing an enabling infrastructure, access to data, tailored explanations, and trust.
Reeve et al. 2013	Patient barriers to and enablers of deprescribing: a systematic review	Australi a	Review	• Many medicines<br>• Focus on potentially inappropriate medication and deprescribing	Mainly adult with some paediatric studies across 21 studies.	Stopping a medication/ deprescribing	• Three themes: disagreement/ agreement with 'appropriateness' of cessation, absence/presence of a 'process' for cessation, and negative/positive 'influences' to cease medication, were identified as both potential barriers and enablers, with 'fear' of cessation and 'dislike' of medications as a fourth barrier and enabler, respectively.<br>• The most common barrier/enabler identified was 'appropriateness' of cessation, with 15 studies identifying this as a barrier and 18 as an enabler.

For peer review only

Reeve et al. 2014	Review of deprescribing processes and development of an evidence-based, patient-centred deprescribing process	Australia	Review	• Many medicines<br>• Focus on inappropriate medication use and deprescribing	Mainly focussed on older adults (≥65 years) with one study range 25-75) with polypharmacy across 10 studies	Deprescribing	• The developed patient-centred deprescribing process, which is a 5-step cycle: gaining a comprehensive medication history, identifying potentially inappropriate medications, determining whether the potentially inappropriate medication can be ceased, planning the withdrawal regimen (e.g. tapering where necessary) and provision of monitoring, support and documentation.<br>• This process focuses on engaging patients throughout the process, with the aim of improving long-term health outcomes.<br>• Despite a comprehensive review of the literature, there is still a lack in the evidence base on which to conduct deprescribing.
Reeve et al. 2015	A systematic review of the emerging definition of 'deprescribing' with network analysis: implications for future research and clinical practice	Australia	Review	• Use of multiple medications<br>• Focus on deprescribing	Older adults prescribed inappropriate medications	N/A	• 8 characteristics of definitions: stop/withdraw/cease/discontinue, aspect of prescribing included e.g. long term therapy/ inappropriate medications, use of the term 'process' or 'structured', withdrawal is planned/supervised /judicious, involving multiple steps, includes dose reduction/ substitution/tapering, desired goals/ outcomes described.<br>• "Deprescribing is the process of withdrawal of an inappropriate medication, supervised by a health care professional with the goal of managing polypharmacy and improving outcomes."
Riordan et al. 2016	The effect of pharmacist-led interventions in optimising prescribing in older adults in primary care: A systematic review	Ireland	Review	• Focus on potentially inappropriate prescribing and medication related problems	Community-dwelling older adults (≥65 years) across 5 studies	Any pharmacist-led intervention designed to reduce potentially inappropriate prescribing or improve medication appropriateness in primary care.	• Overall, this review demonstrates that pharmacist-led interventions may improve prescribing appropriateness in community-dwelling older adults.<br>• However, the quality of evidence is low.

For peer review only

Sanchez-Fidalgo et al. 2017	Prevalence of drug interactions in elderly patients with multimorbidity in primary care.	Spain	Review	• Use of multiple medications<br>• Focus on drug interactions	Older adults (aged 65+) in primary care	N/A	• The prevalence of drug-drug interactions in the elderly with multimorbidity is high, with ACEIs, diuretics and NSAID being the most common therapeutic groups involved
Sawan et al. 2020	A systems approach to identifying the challenges of implementing deprescribing in older adults across different health-care settings and countries: a narrative review	Australia	Review	• Medications for which the risk outweighs the benefit in the individual<br>• ≥5 regularly prescribed medications<br>• Focus on deprescribing	Older adults (≥ 65 years) with multimorbidity (≥2 chronic diseases) and polypharmacy	N/A	• Deprescribing intervention studies are inherently heterogeneous because of the complexity of interventions employed and often do not reflect the real-world.<br>• Process evaluations in deprescribing intervention studies are needed to determine the contextual factors that are important to the translation of the interventions in the real-world.<br>• Deprescribing interventions may need to be individually tailored to target the unique barriers and opportunities to deprescribing in different clinical settings.<br>• Introduction of national policies to encourage deprescribing may be beneficial but need to be evaluated to determine if there are any unintended consequences.
Schiavo et al. 2022	A comprehensive look at explicit screening tools for potentially inappropriate medication: A systematic scoping review.	Brazil	Review	Focus on potentially inappropriate medications	Older adults prescribed potentially inappropriate medications	N/A	• Fifty-eight tools reported 614 PIMs and 747 PIMs–interactions.<br>• Limited overlap between the tools was observed: 123 (69.1%) of 178 therapeutic alternatives proposed by the tools were considered inappropriate by other tools, and 222 (36.1%) of the 614 PIMs identified were named as being inappropriate only once.<br>• Only 21 tools were developed by a Delphi panel technique associated with systematic review.<br>• The PIMs listed as essential medication in Brazil and by the WHO were 30.6% and 23.3% of the total reported, respectively.
Scott et al. 2017	Review of structured guides for deprescribing	Australia	Review	• Appropriate and inappropriate polypharmacy<br>• Focus on deprescribing	Older adults prescribed potentially inappropriate medications in	N/A	• The 7 included guides had considerable heterogeneity, with some guides constituting little more than a set of principles while others entail detailed processes and sub-steps which addressed multiple determinants of drug appropriateness

					both secondary and primary care		• Evidence of effectiveness for each guide was limited in that none have been evaluated in RCTs, and pilot or feasibility studies have involved relatively small samples<br>• More research is needed for determining effectiveness and ease of use in routine clinical practice, especially in primary care settings.
Sirois et al.	Polypharmacy definitions for multimorbid older adults need stronger foundations to guide research, clinical practice and public health	Canada	Review	• > 46 definitions of polypharmacy<br>• Several thresholds based on the number of medications<br>• The majority of the publications (58%) used a minimal threshold of 5 medications.<br>• Heterogeneous qualitative definitions were identified, mostly stating that polypharmacy is "more drugs than needed".	Older adults with multimorbidity and polypharmacy	N/A	• The wide variety of definitions for polypharmacy precludes comparisons, appropriate identification, and management of polypharmacy in multimorbid older adults.<br>• Standardized definitions would allow more coherent judgments regarding the individual and collective stakes of polypharmacy.
Taghy et al. 2020	Failure to Reach a Consensus in Polypharmacy Definition: An Obstacle to Measuring Risks and Impacts—Results of a Literature Review.	France	Review	2 main approaches:<br>• Quantitative, applying varying thresholds and types of polypharmacy based on the number of medications being taken by the patient<br>• Qualitative, based on the clinical indications and effects of a given drug regimen, with a	Polypharmacy in the elderly and the general population (1 included review in children also).	N/A	• The term "inappropriate" is increasingly associated with polypharmacy especially in studies that aimed to use this definition to identify possible solutions for healthcare providers in the field related to aging.<br>• High variability and an evolution in the approaches defining "polypharmacy" in the absence of a consensus following standardized criteria. That makes it very difficult to estimate and measure the outcomes associated with this phenomenon.

For peer review only

				growing number of characteristics to describe polypharmacy			
Ulley et al. 2019	Deprescribing interventions and their impact on medication adherence in community-dwelling older adults with polypharmacy: a systematic review	UK	Review	• Use of any potentially inappropriate medication<br>• Focus on deprescribing<br>• Focus on adherence	Adults (46-97 years) that are community dwelling from 22 studies in 13 countries	Any deprescribing intervention	• There is insufficient evidence to show that deprescribing improves medication adherence. Only 13 studies (of 22) reported adherence of which only 5 were randomised controlled trials.<br>• Adherence was reported as a secondary outcome in all but one study.
Verma et al. 2023	An Overview of Systematic Reviews and Meta-Analyses on the Effect of Medication Interventions Targeting Polypharmacy for Frail Older Adults	Sweden	Review	• Use of multiple medications<br>• ≥5 or ≥10 medications for more than 90 days	Frail older patients (≥60 years)	Any strategy targeting polypharmacy	• Six systematic reviews reported a statistically significant reduction in the number of inappropriately prescribed medications.<br>• Medication reviews help in reducing the use of inappropriate medications in frail older adults<br>• But there is insufficient evidence in terms of frailty score and hospital admissions.
Vrdoljak et al. 2015	Medication in the elderly - considerations and therapy prescription guidelines.	Croatia	Review	More than a few medications potentially inappropriate medications	Older adults with multimorbidity and polypharmacy	N/A	• Lists (e.g. START/STOPP and Beers) are an important practical support in a GP's everyday work.<br>• Implementation of such therapeutic aids reduces the possibility of medical error and minimizes the chance of an inappropriate prescription for this vulnerable population stratum.
Koberlein-Neu et al. 2016	Interprofessional medication management in patients with multiple morbidities.	German y	RCT (cluster, stepped wedge)	• Many medications<br>• ≥5 long-term medications (>3months)<br>• Focus on drug related problems	142 older adults (mean age 76.8 years) with multimorbidity (≥ 3 conditions) in 2 regions in Germany	Medication management and case management (including specialist home visit and pharmacist input)	• Interprofessional collaboration increased medication safety.<br>• Working across disciplinary boundaries allowed for a decrease in drug-related problems and brought up aspects outside the purview of the primary care physician.
Del Cura-González et al. 2022	How to Improve Healthcare for Patients with Multimorbidity and Polypharmacy in Primary Care: A Pragmatic Cluster-	Spain	RCT (cluster)	• Simultaneous consumption of ≥ 5 drugs<br>• Potentially inappropriate medication	593 older adults (between 65-74 years) with multimorbidity (≥3 diseases) and polypharmacy (≥5	MULTIPAP intervention: GPs e-training and GP-patient-centred interview.	• The intervention significantly improved medication appropriateness.<br>• The observed quality of life improvement was not significant.<br>• GPs e-training in multimorbidity has shown to be feasible and well accepted by the professionals.

For peer review only

	Randomized Clinical Trial of the MULTIPAP Intervention			• Focus on appropriateness (MAI)	drugs) during the last three months		
Jager et al. 2017	Impact of a tailored program on the implementation of evidence-based recommendations for multimorbid patients with polypharmacy in primary care practices-results of a cluster-randomized controlled trial.	Germany	RCT (Cluster)	More than 4 drugs	273 adults (aged >50 years, suffering from at least 3 chronic diseases, receiving more than 4 drugs, and being at high risk for medication-related events according to the assessment of the treating GP)	The tailored program consisted of a workshop for GPs and health care assistants, educational materials and reminders for patients, and the elaboration of implementation action plans.	• The tailored program may improve implementation of medication counselling and brown bag review, whereas the use of medication lists and medication reviews did not improve. • No effect of the tailored program on the combined primary outcome could be substantiated
McCarthy et al. 2022	GP-delivered medication review of polypharmacy, deprescribing, and patient priorities in older people with multimorbidity in Irish primary care (SPPiRE Study): A cluster randomised controlled trial	Ireland	RCT (Cluster)	• Multiple medicines use • Higher levels of polypharmacy ≥ 15 medicines • Focus on potentially inappropriate prescribing (STOPP/START)	404 older adults (≥65 years) taking ≥15 regular medicines from 51 GP practices	SPPiRE website: (educational module; template for an individualised patient med review that identified PIP, deprescribing opportunities, and patient priorities	The SPPiRE intervention resulted in a small but significant reduction in the number of medicines but no evidence of a clear effect on PIP. At 6-month follow-up, both intervention and control groups had reductions in the numbers of medicines with a small but significantly greater reduction in the intervention group Less than 2% of drug withdrawals in the intervention group led to a reported adverse drug events.
Muth et al. 2018	Effectiveness of a complex intervention on Prioritising Multimedication in Multimorbidity (PRIMUM) in primary care: results of a pragmatic cluster randomised controlled trial	Germany	RCT (Cluster)	≥5 long-term drug prescriptions with systemic effects	505 older adults (≥60 years) with multimorbidity (≥3 chronic conditions) across 72 general practices in Hesse, Germany.	Healthcare assistant conducted a checklist-based interview with patients on medication-related problems and reconciled their medications. Then using a computerised decision support	• This study found the complex intervention to have no significant effects in older patients with multimorbidity and polypharmacy in general practice. • At baseline, many patients already received appropriate prescriptions and enjoyed good quality of life and functional status.

For peer review only

						system, the GP optimised medication	
Rieckert et al. 2020	Use of an electronic decision support tool to reduce polypharmacy in elderly people with chronic diseases: cluster randomised controlled trial.	Germany	RCT (Cluster)	• ≥8 drugs<br>• Focus on inappropriate polypharmacy and adverse drug events	3904 older adults (≥75 years) using ≥8 medications across 359 practices (4 countries)	An electronic decision support tool comprising a comprehensive drug review to support general practitioners in deprescribing potentially inappropriate and non-evidence-based drugs.	• In intention-to-treat analysis, a computerised decision support tool for comprehensive drug review of elderly people with polypharmacy showed no conclusive effects on the composite of unplanned hospital admission or death by 24 months.<br>• A reduction in drugs was achieved without detriment to patient outcomes.
Schafer et al. 2018	Narrative medicine-based intervention in primary care to reduce polypharmacy: results from the cluster-randomised controlled trial MultiCare AGENDA	Germany	RCT (Cluster)	• Co-prescription of multiple medications<br>• Focus on the patient's perspectives	604 older adults (mean age 73 years) from 55 practices in 3 areas in Germany	GPs had 3x 30 min consultations.<br>1. aimed at identifying treatment targets and priorities of the patient.<br>2. 'brown bag' review<br>3. discuss goal attainment and future treatment targets.	Intensifying the doctor–patient dialogue and discussing the patient's agenda and personal needs did not lead to a reduction of medication intake and did not alter health-related quality of life.
Zechmann et al. 2020	Effect of a patient-centred deprescribing procedure in older multimorbid patients in Swiss primary care - A cluster-randomised clinical trial	Switzerland	RCT (cluster)	• Multiple medications<br>• ≥5 drugs for ≥6 months<br>• Focus on number, but also patient safety and QoL	334 older adults (≥60 years, mean 76.2) with multimorbidity across 46 GPs in North Switzerland	GP training encouraging the use of a validated deprescribing-algorithm including shared-decision-making	• The patient-centred deprescribing procedure is effective immediately after the intervention, but not after 6 and 12 months.<br>• Further research needs to determine the optimal interval of repeated deprescribing interventions for a sustainable effect on polypharmacy at mid- and long-term.
Knowles et al. 2018	Empowering people to help speak up about safety in primary care: Using codesign to	UK	Qualitative (workshop)	• Large number of medications	11 patients with multimorbidity or carers (plus a	An intervention to empower patients and carers to raise	• Both patients and professionals prioritized polypharmacy as a threat to safety.

	involve patients and professionals in developing new interventions for patients with multimorbidity			• Complex medication schedule	second workshop with five HCPs)	safety issues in primary care	• Findings emphasized the limited capacity of patients with multimorbidity and the need for services to proactively offer support to reduce the burden of managing complex treatment regimes.<br>• There is a need for accessible reminders to support medication adherence and medication reviews for particularly vulnerable patients conducted with pharmacists within GP practices.
Rozsnyai et al. 2020	What do older adults with multimorbidity and polypharmacy think about deprescribing? The LESS study - a primary care-based survey	Switzerland	Qualitative (questionnaire)	• ≥5 chronic medications<br>• Focus on inappropriate medications and deprescribing	300 older adults (≥70 years,) with multimorbidity (≥3 chronic conditions) and polypharmacy	Deprescribing	• The majority of participants (77%) were willing to deprescribe one or more of their medicines if their doctor said it was possible. There was no association with sex, age or the number of medicines and willingness to deprescribe.<br>• Increased willingness to deprescribe was linked to a good relationship with their GP and that they would feel that deprescribing was safe and if new studies showed an avoidable risk.<br>• The most common barriers towards deprescribing were patients feeling well on their current medicines and being convinced that they need all their medicines.
Anthierens et al. 2010	Qualitative insights into general practitioners views on polypharmacy	Belgium	Qualitative (interviews)	• concomitant use of ≥3 drugs<br>• use of more drugs than indicated	People with polypharmacy	Interviews with 65 GPs	• It is a problem in their older patient population, especially because of the risk of adverse drug reactions, interactions and lowered adherence.<br>• Difficulties in keeping an overview of the exact medication intake<br>• Patients' strong belief in their medication and self-medication are seen as important barriers<br>• According to the respondents, prevention and evidence based medicine guidelines often induce polypharmacy.
Clyne et al. 2016	"Potentially inappropriate or specifically appropriate?" Qualitative evaluation of general practitioners' views on prescribing,	Ireland	Qualitative (interviews)	• Multiple medications<br>• Focus on Potentially inappropriate prescribing (PIP)	196 older adults (≥70 years) with multimorbidity (≥2 chronic medical conditions)	OPTI-SCRIPT (academic detailing with a pharmacist on conducting GP-led medicines review with participating	• 3 main, inter-related themes emerged (complex prescribing environment, paternalistic doctor-patient relationship, and relevance of PIP concept).<br>• Patient complexity (e.g. polypharmacy, multimorbidity), as well as prescriber complexity (e.g. multiple prescribers, poor communication, restricted autonomy) were all identified as

For peer review only

	polypharmacy and potentially inappropriate prescribing in older people					patients; medicines reviews supported by web-based algorithms for GPs providing alternatives for specific PIP meds identified by the research pharmacist; and tailored patient information leaflets)	factors contributing to a complex prescribing environment where PIP could occur, as was a paternalistic-doctor patient relationship.<br>• The concept of PIP was perceived to be of variable usefulness to GPs and the criteria to measure it may be at odds with the complex processes of prescribing for this patient population.
Laursen et al. 2018	General Practitioners' Barriers Toward Medication Reviews in Polymedicated Multimorbid Patients: How can a Focus on the Pharmacotherapy in an Outpatient Clinic Support GPs?	Denmar k	Qualitative (interviews)	• concomitant use of ≥2 drugs<br>• often arbitrarily set at cut-off value ≥5	People with polypharmacy and multimorbidity (≥2 chronic diseases)	Interviews with 14 GPs	• The primary barriers toward multimorbid patients with polypharmacy were the need for communication and teamwork with specialists<br>• Often, GPs felt that the specialists were more concerned about following standards and guidelines regarding specific diseases instead of a more holistic patient approach.<br>• To improve management of polypharmacy patients, the GPs suggest that a joint force is necessary, a partner-like relationship with greater transparency regarding information transfer, feedback, and shared decision-making.
McNamara et al. 2017	Health professional perspectives on the management of multimorbidity and polypharmacy for older patients in Australia	Australi a	Qualitative (interviews)	• Using multiple medications<br>• Focus on appropriate medications management	Older adults (≥65 years) with multimorbidity and polypharmacy	Interviews with a range of 26 health professionals (14 prescribers and 12 non-prescribers, with 5 GPs, 5 hospital professionals, 6 nursing, 6 pharmacy staff)	• Most participants did not routinely use structured approaches to incorporate patients' preferences in clinical decision-making, address conflicting prescriber advice, assess patients' adherence to treatment plans or seek to optimise care plans.<br>• Challenges with coordination and continuity of care, pressures of workload and poorly defined individual responsibilities for care, all contributed to participants' avoiding ownership of multimorbidity management.
Schöpf et al. 2018	Elderly patients' and GPs' perspectives of patient–GP	German y	Qualitative (interviews)	• Concurrent prescription of at	6 older adults (≥65 years) along with 3 GPs in a	Communication about polypharmacy,	• Patients' awareness of the significance of their active role in addressing polypharmacy needs to be increased.

For peer review only

	communication concerning polypharmacy: a qualitative interview study			least 4 or 5 medications<br>• Appropriate medication therapy	single primary care centre in Germany	safety and empowerment	• This includes understanding that trusting the doctor does not preclude asking questions or seeking more information.<br>• GPs might support patients by 'inviting' their contribution.<br>• We need interventions which improve patients' communication skills and address specific issues of polypharmacy, particularly in elderly patients
Sinnott et al. 2015	What to give the patient who has everything? A qualitative study of prescribing for multimorbidity in primary care	Ireland	Qualitative (interviews)	• Appropriate and inappropriate polypharmacy	51 adults (median 75 years, range 39-92 years) with multimorbidity (mean 8.3 conditions)	GP interviews	• Difficulties arose when recommendations or preferences conflicted, to which GPs responded by 'satisficing': accepting care that they deemed satisfactory and sufficient for a particular patient.<br>• Satisficing was manifest as relaxing targets for disease control, negotiating compromise with the patient, or making 'best guesses' about the most appropriate course of action.<br>• In patients perceived as stable, GPs preferred to 'maintain the status quo' rather than rationalise medications, even in cases with significant polypharmacy.<br>• Proactive changes in medications were facilitated by continuity of care, sufficient consultation time, and open lines of communication.
Wallis et al. 2017	Swimming Against the Tide: Primary Care Physicians' Views on Deprescribing in Everyday Practice	New Zealand	Qualitative (interviews)	• Multiple medicines<br>• Focus on potentially inappropriate prescribing and deprescribing	People with polypharmacy and multimorbidity (many chronic conditions)	Interviews with 24 GPs	• Deprescribing was "swimming against the tide" of patient expectations, the medical culture of prescribing, and organizational constraints.<br>• It came with inherent risks for both themselves and patients and conveyed a sense of vulnerability in practice.<br>• The only incentive to deprescribing was the duty to do what was right for the patient.<br>• Physicians recommended organizational changes to support safer prescribing, including targeted funding for annual medicines review, computer prompts, improved information flows between prescribers, improved access to expert advice and user-friendly decision support, increased availability of non-pharmaceutical therapies, and

For peer review only

							enhanced patient engagement in medicines management.
Weir et al. 2021	The role of older patients' goals in GP decision-making about medicines: a qualitative study	Australi a	Qualitative (interviews)	• Taking ≥ 5 medicines<br>• Over-prescribing/ under-prescribing<br>• Inappropriate selection of a medication<br>• Avoidable adverse drug reactions	GPs caring for older patients with polypharmacy	N/A	• Most GPs recognised some value in understanding older patients' goals and preferences regarding their medicines.<br>• GPs differed on the following main themes: 1) definition and perception of patients' goals, 2) relationship with the patient, 3) approach to medicines management and prioritisation.<br>• We observed that GPs preferred one of three different practice patterns in their approach to patients' goals in medicines decisions: 1) goals and preferences considered lower priority – 'Directive'; 2) goals seen as central – 'Goal-oriented'; 3) goals and preferences considered but not explicitly elicited – 'Tacit'.
Zechmann et al. 2019	Barriers and enablers for deprescribing among older, multimorbid patients with polypharmacy: an explorative study from Switzerland	Switzerl and	Qualitative (interviews)	• > 5 drugs/day<br>• Inappropriate polypharmacy<br>• Focus on benefit and harm ratio, admissions and mortality	19 older adults (mean age 76.9 years) with multimorbidity taking 8.9 mean drugs per day.	Interviews	• We identified patient involvement in deprescribing and coordination of care as key issues for deprescribing<br>• Conservatism/inertia and fragmented medical care were the main barriers towards deprescribing. No patient felt devalued as a consequence of the deprescribing offer.<br>• GPs concerns regarding patients' devaluation should not prevent them from actively discussing the reduction of drugs.
Anderson et al. 2017	Negotiating "Unmeasurable Harm and Benefit": Perspectives of General Practitioners and Consultant Pharmacists on Deprescribing in the Primary Care Setting	Australi a	Qualitative (focus groups)	• Use of more medicines than are clinically indicated or concurrent use of multiple medicines<br>• no consensus, but ≥5 commonly cited<br>• Focus on potentially inappropriate polypharmacy and deprescribing	Older adults (≥65 years) with polypharmacy who were still residing in the community	Focus group (32 GPs and 15 clinical pharmacists)	• Deprescribing is an inherently uncertain venture<br>• The option to deprescribe is shaped by many factors, including a clinician's perception of the risk/benefit ratio of persisting with the status quo versus deprescribing.<br>• It is more likely to occur in the presence of a continuous therapeutic relationship between the GP and patient and in response to a clear clinical trigger or finding of "low-hanging fruit."<br>• However, poorly developed interprofessional relationships and a lack of dedicated time and tacit knowledge/familiarity with patients, respectively, are important barriers to deprescribing.

For peer review only

Mangin et al. 2019	"I think this medicine actually killed my wife": patient and family perspectives on shared decision-making to optimize medications and safety	Canada	Qualitative (focus groups)	• ≥5 long-term medications<br>• Inappropriate medications<br>• Focus on benefit vs harms	16 adults (55-90 years) with multimorbidity (≥ 2 chronic conditions) from Southern Ontario and British Columbia (convenience sample)	How patient/family preferences and priorities are considered in medication-related discussions and decisions within the healthcare system	• Shared decision-making resulted from both recognition and integration of the personal expertise of the patient and family in medications, and perceived patient-centredness.<br>• It is complex, dynamic, and nonlinear, and patient priorities are not as integrated into shared decision-making about medications as we would hope.<br>• This is broadly consistent with the current conceptualization of evidence-based medicine.<br>• This suggests the need for developing a systematic process to elicit, record, and integrate patient preferences and priorities about medications to create space for a more patient-centred conversation.
Noel et al. 2005	Collaborative care needs and preferences of primary care patients with multimorbidity	USA	Qualitative (focus groups)	• Multiple medications<br>• Medication side effects and burdens	60 patients with ≥2 chronic illnesses (taking 8-27 medications) across 8 primary care clinics within the Veterans Health Administration.	Any care need or preference for primary care patients with multiple chronic illnesses	• Polypharmacy was a major concern.<br>• Problematic interactions with providers and the health care system were also mentioned, often in relation to specialty care and included incidents in which providers had ignored concerns or provided conflicting advice<br>• Knowledge and skills deficits interfered with self-management.<br>• Participants were willing to use technology for monitoring or educational purposes if it did not preclude human contact
Sinnige et al. 2016	Medication management strategy for older people with polypharmacy in general practice: a qualitative study on prescribing behaviour in primary care.	Netherlands	Qualitative (Focus groups)	• Chronic use of ≥5 medications<br>• appropriate polypharmacy	Older adults (aged 68–84 years) diagnosed with multiple, highly prevalent chronic diseases, often part of a cluster of diseases	Focus groups with 12 GPs	• Similar strategy regarding the patients' medication management: defining treatment goals; determining primary goals; and adjusting medications based on the treatment effect, GPs' and patients' preferences, and patient characteristics.<br>• The GPs would like to discuss their choices with other professionals, and they valued structured medication reviews with the patient, as well as quick and practical support tools that work on demand.
Smith et al. 2010	GPs' and pharmacists' experiences of managing	Ireland	Qualitative (Focus groups)	• Multiple medications use<br>• ≥5 medications	People with multimorbidity	3 focus groups with 13 GPs and 7 pharmacists	• Themes included 1) the concept of multimorbidity and the link to polypharmacy and ageing; 2) health systems issues relating to lack

For peer review only - http://bmjopen.bmj.com/site/about/guidelines.xhtml

	multimorbidity: a "Pandora's box"						to time, inter-professional communication difficulties, and fragmentation of care; 3) individual issues from clinicians relating to professional roles, clinical uncertainty, and avoidance; 4) patient issues; and 5) potential management solutions.
Uhl et al. 2018	Patient-perceived barriers and facilitators to the implementation of a medication review in primary care: a qualitative thematic analysis.	Germany	Qualitative (focus groups and telephone interviews)	• ≥5 drugs per day<br>• Inappropriate polypharmacy	31 older adults (range 62–88 years), ≥3 chronic diseases, from 17 General practices in Frankfurt (convenience sample)	Medication review	• Barriers to patient participation concerned patient autonomy, while facilitators involved patient awareness of medication-related problems.<br>• Barriers to GP-led reviews concerned GP's lack of resources while facilitators related to the trusting relationship between patient and GP.<br>• Pharmacist-led reviews might be hindered by a lack of patients' confidence in pharmacists' expertise, but facilitated by pharmacies' digital records of the patients' medications.
Reeve et al. 2016	Beliefs and attitudes of older adults and carers about deprescribing of medications: a qualitative focus group study	Australia	Qualitative (focus group)	• Many medicines<br>• Focus on inappropriate medication and deprescribing	14 older adults (mean age 75 years) and 14 carers (mean age 79) across 4 focus groups	Deprescribing	• Main factors: Their perception of the appropriateness of that medication; fear of outcomes of withdrawal; dislike of taking medications; and the availability of a process for withdrawal (including a discussion with a healthcare professional and knowing that the medication could be restarted if necessary).<br>• A patient's regular GP was identified as a strong influence both for and against medication withdrawal.<br>• A theme unique to the carers was the complexity involved in making decisions about medications for their care recipients.
Bell et al. 2017	Nurses' and pharmacists' learning experiences from participating in interprofessional medication reviews for elderly in primary health care - a qualitative study	Norway	Qualitative (focus group and telephone interviews)	• ≥4 drugs<br>• Inappropriate drug use	Elderly patients with polypharmacy and multimorbidity in primary care	Interprofessional medication reviews	• Although experiencing challenges in conducting IMRs, the nurses and pharmacists had learning experiences they said improved both their own practice and the quality of drug management.<br>• There are some challenges concerning how to ensure participation of all three professions and how to get thorough information about the patient.

For peer review only

Collier et al. 2023	Older people, medication safety, and the role of the community pharmacist: a longitudinal ethnographic study	Australia	Qualitative (ethnography)	• ≥5 regular or 'as required', prescription or non-prescription medications<br>• Focus on medication safety	20 older adults (≥65 years) with frailty and multimorbidity	Community pharmacist involvement	• Community pharmacists play a significant role in the medication safety of older people with frailty and polypharmacy. Analysis resulted in three main themes:<br>1) the older person–determined role of the pharmacist,<br>2) the 'taken for granted' safety work of the pharmacist<br>3) collective agency and medication safety.
Fudge et al 2021	'It's all about patient safety': an ethnographic study of how pharmacy staff construct medicines safety in the context of polypharmacy	UK	Qualitative (ethnography)	• Concurrent use of ≥5 medications<br>• Focus on medication safety	33 pharmacy staff (counter staff, technicians, dispensers, pharmacists)	Community pharmacist involvement	• 'Safety' in the performance of practices relating to polypharmacy was not a fixed, defined notion, but an ongoing, collaborative accomplishment, emerging within an organisational narrative of 'care'.<br>• Despite meticulous attention to 'safety', carefully guarded professional boundaries meant that addressing polypharmacy per se in the context of community pharmacy was beyond reach.
Swinglehurst et al. 2021	Organising polypharmacy: unpacking medicines, unpacking meanings—an ethnographic study	UK	Qualitative (ethnography)	• Multiple medications<br>• ≥10 'higher-risk' polypharmacy<br>• Appropriate and inappropriate polypharmacy	24 older adults (≥65 years) with multimorbidity and ≥10 regular medications from 3 NHS GP Practices (purposive sampling), ethnography also covered 4 community pharmacies	Medication reviews	• Polypharmacy demands careful organising.<br>• All patients had developed strategies and routines for organising medicines into their lives, negotiating medicine taking to enable acceptable adherence and make their medicines manageable.<br>• Strategies adopted by patients often involved the use of 'do-it-yourself' dosette boxes.<br>• Patients incorporated a range of approaches to manage supplies and flex their regimens to align with personal values and priorities. Practices of organising medicines are effortful, creative and often highly collaborative.<br>• Patients strive for adherence, but their organisational efforts privilege 'living with medicines' over taking medicines strictly 'as prescribed'.
Swinglehurst et al. 2023	Negotiating the polypharmacy paradox: a video-reflexive ethnography study of	UK	Qualitative (ethnography)	• Multiple medications<br>• ≥10 'higher-risk' polypharmacy	24 older adults (≥65 years) with multimorbidity and ≥10 regular	Medication reviews	• Participants rarely referenced biomedical aspects of prescribing (eg, drug-drug interactions, 'Numbers Needed to Treat/Harm') focussing instead on polypharmacy as an

	polypharmacy and its practices in primary care			• Appropriate and inappropriate polypharmacy	medications from 3 NHS GP Practices (purposive sampling), ethnography also covered 4 community pharmacies		emotional and relational challenge. Clinicians initially denigrated their medication review work as mundane. Through VRE they reframed their work as complex, identifying polypharmacy as a delicate matter to negotiate.<br>• Medication review was identified as an ongoing process, rather than a discrete 'one-off' activity. Meaningful progress towards tackling polypharmacy was only possible through small, incremental, carefully supported changes in which both patient and clinician negotiated a sharing of responsibility, best supported by continuity of care.
Haque. 2009	ARMOR: a tool to evaluate polypharmacy in elderly persons.	USA	Qualitative (case study)	• Use of multiple medications<br>• ≥9 medications	Elderly adults who are receiving nine or more medications; with an assessment for falls and admitted for rehabilitation.	N/A	• The ARMOR tool (Assess, Review, Minimize, Optimize, Reassess) is an attempt to consolidate these recommendations into a functional and interactive tool.<br>• It takes into account the patient's clinical profile and functional status and tries to balance evidence-based practice with altered physiological reserves.<br>• The application of this tool has led to significant reduction in polypharmacy, reduced cost of care, and marked decrease in hospitalization at our facility
McIntosh et al. 2018	A case study of polypharmacy management in nine European countries: Implications for change management and implementation.	Spain (authors consortium includes 8 EU countries)	Qualitative (case study)	• ≥5 medications<br>• Appropriate and inappropriate polypharmacy	Older adults (varied but all ≥50 years old), some specific to geriatric departments, or care homes	Varied identified initiatives across 5 sites: Spain (Catalonia), Germany (Lower Saxony), Sweden (Uppsala), and two UK sites (Northern Ireland and Scotland).	• Within the studied EU countries, polypharmacy management was not widely addressed.<br>• Results highlight the importance of change management and theory-based implementation strategies.
All Wales Medicines Strategy Group. 2023	Polypharmacy in older people: A guide for healthcare professionals.	UK (Wales)	Policy	• Use of multiple medications<br>• Appropriate and problematic polypharmacy	Adults with polypharmacy	N/A	• Assessing polypharmacy and patient adherence to medication is imperative.<br>• To support medicines optimisation in older patients who may be subject to inappropriate

For peer review only

				• Particular high risk medications			polyphramacy, deprescribing high risk medicines is practical
Australian Commission on Safety and Quality in Health Care. 2020	Polypharmacy, 75 years and over	Australi a	Policy	≥5 medications at the same time	Older adults (≥75 years) with polypharmacy	N/A	• There are widespread variations in polypharmacy prevalence across the country<br>• Further guidance is needed for communication between medical teams and residential homes, medication management reviews and system changes including establishment, governance and advisory committees.
King's Fund	Polypharmacy and medicines optimisation.	UK	Policy	• Appropriate polypharmacy: prescribing for an individual for complex conditions or for multiple conditions in circumstances where medicines use has been optimised and where the medicines are prescribed according to best evidence.<br>• Problematic polypharmacy: prescribing multiple medications inappropriately, or where the intended benefit of the medication is not realised.	Adults with polypharmacy	N/A	• For many people, appropriate polypharmacy will extend life expectancy and improve quality of life. With meds optimised and prescribed according to best evidence.<br>• Problematic polypharmacy can cause an increased risk of drug interactions and adverse drug reactions, together with impaired adherence and QoL<br>• Many clinical trials and practice guidelines do not consider polypharmacy in the context of multi-morbidity.
Scottish Governmen t Polypharma cy Model of Care Group. 2018	Polypharmacy guidance, realistic prescribing. 3rd Edn.	UK (Scotlan d)	Policy	• Many medications<br>• Appropriate and inappropriate polypharmacy	Adults with polypharmacy	N/A	• The case for effective polypharmacy management is quite clear, but in a complex healthcare setting with many competing priorities it is useful to outline the quality and economic reasons why it should be prioritised.<br>• Need to address polypharmacy management as a public health issue, as multi-morbidities do not just affect the elderly.

For peer review only

							• Greater emphasis has been placed on shared-decision making to actively engage the patient with the 7-Step medication review.<br>• The Drug Efficacy (NNT) tables help discussion regarding the relative potential benefits of a range of common therapeutic interventions.
The Department of Health and Social Care 2021	National overprescribing review report: Good for you, good for us, good for everybody	UK	Policy	• Overprescribing – the use of a medicine where there is a better non-medicine alternative, or the use is inappropriate for that patients' circumstances and wishes.<br>• Polypharmacy – the concurrent use of multiple medicines for one person. There is currently no consensus on a definition for polypharmacy.<br>• Prevalence calculated at 5+ and 8+.	Adults with polypharmacy	N/A	• Overprescribing is a serious problem in health systems internationally that has grown dramatically over the last 25 years, with systemic and cultural causes.<br>• Estimates at least 10% of medicines overprescribed
McCarthy et al 2020	The evolution of an evidence based intervention designed to improve prescribing and reduce polypharmacy in older people with multimorbidity and significant polypharmacy in primary care (SPPiRE)	Ireland	Pilot and protocol	• Multiple medicines use<br>• Higher levels of polypharmacy ≥ 15 medicines<br>• Focus on potentially inappropriate prescribing (STOPP/START)	10 older adults (≥65 years) taking ≥15 regular medicines	SPPiRE website: educational module<br>template for an individualised patient<br>medication review that identified PIP, deprescribing opportunities, and patient priorities	• A framework was used to systematically describe how and why the original intervention was modified, allowing the new intervention to build upon an effective and robustly developed intervention but also to be relevant in the context of the current evidence base.

McCarthy et al. 2017	Supporting prescribing in older people with multimorbidity and significant polypharmacy in primary care (SPPiRE): a cluster randomised controlled trial protocol and pilot.	Ireland	Pilot and protocol	• Multiple medicines use<br>• Higher levels of polypharmacy ≥ 15 medicines<br>• Focus on potentially inappropriate prescribing (STOPP/START)	10 older adults (≥65 years) taking ≥15 regular medicines	SPPiRE website: educational module<br>template for an individualised patient<br>medication review that identified PIP, deprescribing opportunities, and patient priorities	• The feasibility of the finder tool and intervention were assessed through GP feedback and analysis of use of the online material. Overall, the intervention was well received by the GPs and their patients many of whom reported feeling reassured that their medicines were being reviewed and rationalised.<br>• When the higher prevalence, lower risk proton pump inhibitor PIP was excluded, 90% of patients had at least 1 PIP and the mean number of PIP per patient was 1.1. Identified instances of PIP were acted on in 44% of cases and 45% when the proton pump inhibitor PIP was excluded.<br>• The most common patient priorities were treating pain, followed by fatigue, and reducing the number of repeat medicines.
Kirwan et al. 2022	The multimorbidity collaborative medication review and decision making (MyComrade) study: a pilot cluster randomised trial in two healthcare systems	UK and Ireland	Pilot (Cluster RCT)	• ≥10 medications	121 adults (≥18 years and over, with multi morbidity and ≥10 medications living in the community across 15 GP practices in Northern Ireland and Ireland	Training (Face-to-face or pre-recorded) and inclusion of practice-based pharmacists in NI as collaborative reviewers using the NO TEARS tool to encourage shared decision making	• Both practice staff and patients found the intervention acceptable and reported strong fidelity to the My Comrade intervention components.<br>• Some staff highlighted concerns such as poor communication of the reviews to patients, dissatisfaction regarding incentivisation and in ROI the sustainability of two GPs collaboratively conducting the medication reviews.<br>• Pairing of GP and pharmacist may be more sustainable to implement in routine practice.
Rankin et al. 2022	An external pilot cluster randomised controlled trial of a theory-based intervention to improve appropriate polypharmacy in older people in primary care (PolyPrime).	UK and Ireland	Pilot (Cluster RCT)	• ≥ 5 medicines<br>• ≥ 4 medications for ≥3 months<br>• Focus on potentially inappropriate prescribing (STOPP/START and NO TEARS)	120 older adults (≥ 70 years old) prescribed ≥4 medications for ≥3 months living in the community across 12 GP practices in Northern Ireland and Ireland	An online video (incorporating behaviour change) and scheduled medication reviews with patients on 2 occasions.	• The intervention was successfully delivered as intended; it was acceptable to GPs, practice staff, and patients; and potential mechanisms of action have been identified.<br>• It may be feasible to conduct an intervention to improve appropriate polypharmacy in older people in primary care across two healthcare jurisdictions.
Cardwell et al. 2020	Evaluation of the General Practice Pharmacist (GPP)	Ireland	Pilot	• Many medications<br>• Focus on potentially inappropriate	786 older adults (mean age 69.8	A pharmacist joined the practice team for	An intervention involving pharmacists, working within general practices is feasible to implement and has potential to improve prescribing quality.

	intervention to optimise prescribing in Irish primary care: a non-randomised pilot study.			prescribing, high risk medications and deprescribing	years) from 4 General practices	6 months and undertook medication reviews (face to face or chart based), provided prescribing advice and facilitated practice-based education	
Michiels-Corsten et al. 2022	MediQuit – an electronic deprescribing tool: a pilot study in German primary care; GPs' and patients' perspectives	Germany	Pilot	• Multiple medications<br>• ≥ 5 drugs<br>• Excessive polypharmacy = ≥ 10 drugs<br>• Inappropriate polypharmacy<br>• Focus on overprescribing and deprescribing	41 older adults (mean age 77 years) with at least 3 chronic diseases (mean was 8) with ≥ 10 drugs in 2 regions of Germany	MediQuit - Electronic Deprescribing Tool (3 steps: 1. Med review with algorithm to flag PIM for deprescribing. 2. Communcation prompts, and risk-benefits. 3. Information on discontinuation process).	• Identification (step 1) and implementation elements (Step 3) were perceived most helpful by GPs. Whereas, shared-decision making elements (step 2) revealed room for improvement.<br>• Patients were broadly satisfied with the deprescribing consultation (85%) and with their decision made regarding their medication (90%).<br>• GPs were satisfied tool and gave important hints for future development.
McCarthy et al. 2023	Medication changes and potentially inappropriate prescribing in older patients with significant polypharmacy.	Ireland	Longitudinal study (secondary analysis of RCT at 2 time points)	• Multiple medicines use<br>• Focus on potentially inappropriate prescriptions (SPPiRE PIP critieria)	404 older adults (aged ≥ 65 years), prescribed ≥ 15 repeat medicines, from 51 different general practices	N/A	• There were reductions in the prescription of most drug groups with the largest reduction in antiplatelet prescriptions.<br>• Considering medication discontinuations, initiations and switches, there was a median of 5 medication changes per person<br>• A high proportion of benzodiazepine, anticholinergic and diuretic prescriptions were potentially inappropriate suggesting these higher risk groups may warrant specific attention.
Muller et al. 2020	Development and internal validation of prognostic models to predict negative health outcomes in older patients with	Germany	Longitudinal study (prognostic modelling)	• Multiple medicines use<br>• Appropriate & inappropriate polypharmacy	592 958 older adults (≥60 years, ≥5 drugs, ≥3 chronic diseases, excluding	N/A	• Best trial data-based model predicted HRQoL after 6 months well and included parameters of well-being not found in claims.<br>• Performance of claims data-based models and models predicting long-term outcomes was relatively weak.

For peer review only

	multimorbidity and polypharmacy in general practice.			• Focus on Quality of life	dementia) in Germany		
Kruger et al. 2021	Non-random relations in drug use expressed as patterns comprising prescription and over-the-counter drugs in multimorbid elderly patients in primary care: Data of the exploratory analysis of the multicentre, observational cohort study MultiCare.	Germany	Longitudinal study (observational cohort study)	Chronic co-prescription or co-application of different drugs at the same time	3189 older adults (65-85 years) with at least 3 chronic diseases	N/A	There are strong associations between drug patterns and multimorbidity clusters, which enrich the knowledge about the treatment of multimorbid elderly patients in primary care.
Carr et al 2021	A multidimensional measure of polypharmacy for older adults using the Health and Retirement Study	UK	Longitudinal study	• Concurrent use of multiple medications by one individual<br>• Added dimensions e.g. temporality, dosage, appropriateness, anticholinergic properties, potential drug interactions<br>• Cut offs of 5+ and 9+ used in comparative analyses	2141 adults (aged 50-80) living in the USA	N/A	• A four-class model was selected based on fit statistics and clinical interpretability.<br>• Participants in the 'low risk' class tended to be male, cohabitating, and reported fewer health conditions, compared to 'high risk' classes.<br>• The three 'high risk' classes overlapped with the groups concurrently taking 5+ and 9+ medications per month.
Häppölä et al. 2020	A data-driven medication score predicts 10-year mortality among aging adults.	Finland	Longitudinal study	Use of numerous potentially interacting medications.	20,078 adults (aged 46-74) in community sites across Finland (mainly North)	N/A	• The resulting score is strongly associated with all-cause mortality (HR 1.18 per point increase in score; 95% CI 1.14–1.22)<br>• When combined with Charlson comorbidity index, individuals had over 6x risk (HR 6.30; 95% CI 3.84–10.3) compared to individuals with a protective score profile.<br>• Alone, the medication score performs similarly to the Charlson comorbidity index
Monterde et al. 2020	Multimorbidity as a predictor of health service utilization in primary care: a registry-	Spain	Longitudinal study	Prescription of more than 8 drugs over one year	6,102,595 adults (≥18 years) in Catalonia	N/A	• Multimorbidity assessment enhanced prediction of use of healthcare resources at community level

For peer review only

	based study of the Catalan population.						• Clinical risk groups had a higher predictive performance for polypharmacy over Charlson Index and Adjusted Morbidity Groups
Villén et al. 2020	Multimorbidity patterns, polypharmacy and their association with liver and kidney abnormalities in people over 65 years of age: a longitudinal study.	Spain	Longitudinal study	concomitant consumption of ≥5 medications	743,827 older adults (65–99 years) in 285 primary health care centres (PHCCs) in Catalonia	N/A	• The most frequently prescribed medicines were related to multimorbidity patterns and their consumption was maintained throughout the follow-up period.<br>• A higher risk of abnormal kidney and liver function was observed in specific multimorbidity patterns.
Guthrie et al. 2015	The rising tide of polypharmacy and drug-drug interactions: population database analysis 1995-2010	UK (Scotland)	Longitudinal analysis (retrospective cohort)	• concomitant prescription of ≥5 or ≥10 drugs<br>• Major or excessive polypharmacy<br>• 3 levels of analysis for polypharmacy as ≥5, ≥10, and ≥15 drugs dispensed in the previous 84 days.<br>• Focus on drug-drug interactions	310,000 adults (≥20 years, mean 48 years) in one region of Scotland	N/A	• Between 1995 and 2010, the proportion of adults dispensed ≥5 drugs doubled to 20.8%, and the proportion dispensed ≥10 tripled to 5.8%.<br>• Receipt of ≥10 drugs was strongly associated with increasing age but was also in people living in more deprived areas or in a care home<br>• The proportion with potentially serious drug-drug interactions more than doubled to 13% of adults in 2010, and the number of drugs was most strongly associated with this
Payne et al. 2014	Is polypharmacy always hazardous? A retrospective cohort analysis using linked electronic health records from primary and secondary care	UK	Longitudinal analysis (retrospective cohort)	• Prescription of multiple medications<br>• Focus on appropriate vs inappropriate polypharmacy	180 815 adults with long-term clinical conditions and numbers of regular medications	N/A	• Admissions were more common in patients on multiple medications, but admission risk varied with the number of conditions.<br>• Unplanned hospitalization is strongly associated with the number of regular medications. However, the effect is reduced in patients with multiple conditions, in whom only the most extreme levels of polypharmacy are associated with increased admissions.<br>• Assumptions that polypharmacy is always hazardous and represents poor care should be tempered by clinical assessment of the conditions for which those drugs are being prescribed.
von Buedingen et al. 2018	Changes in prescribed medicines in older patients with	Germany	Longitudinal analysis (of RCT data)	• Regular use of five or more drugs	505 older adults (median age 72 years) with	PRIMUM (The healthcare assistant	• Medication regimens in older patients with multimorbidity and polypharmacy changed frequently.

For peer review only

	multimorbidity and polypharmacy in general practice			• Inappropriate prescriptions	multimorbidity (≥3 chronic conditions) across 72 general practices in Hesse, Germany.	conducted a checklist-based interview with patients on medication-related problems and reconciled their medications. Then, using a computerised decision support system, the GP optimised medication, with patients)	• These are mostly due to discontinuations and dosage alterations, followed by additions and restarts.<br>• These findings cast doubt on the effectiveness of cross-sectional assessments of medication and support longitudinal assessments where possible.
Alaa Eddine et al. 2020	A pharmacist-led medication review service with a deprescribing focus guided by implementation science	Lebanon	Longitudinal analysis (cohort)	• ≥ 5 medicines<br>• Drug related problems and potentially inappropriate prescribing<br>• Focus on deprescribing	143 older adults (≥ 65 years) at a single site (mixed GP/physicians in primary care facility) in Lebanon for low-income patients (receiving free medication)	An pharmacist-led medication review, with recommendations placed with GPs/ physicians at the facility.	• The intervention pharmacist provided 221 recommendations to physicians, of which 52% were to discontinue one or more medications.<br>• Patients in the intervention group showed significantly higher satisfaction compared to the ones in the control group ($p < 0.001$, effect size = 1.75).<br>• Of all recommendations, 30% were accepted by the physicians.
Vos et al. 2022	Fifteen-year trajectories of multimorbidity and polypharmacy in Dutch primary care—A longitudinal analysis of age and sex patterns.	Netherlands	Longitudinal analysis	≥5 or more different medications in one year	10,037 patients, all ages from GP practices in South Netherlands	N/A	• Multimorbidity and polypharmacy are common, and their prevalence is accelerating, with a relatively rapid increase in younger groups.<br>• This underlines the need for a longitudinal approach and a life course perspective in patient care.
Novella et al. 2022	Relation between drug therapy-based comorbidity indices, Charlson's comorbidity index, polypharmacy and mortality in three samples of older adults.	Italy	Longitudinal analyses (retrospective cohort)	• Polypharmacy: ≥5 medications<br>• Excessive polypharmacy: ≥10 medications<br>• This definition included systemic and topical drugs	Older adults (all 65+ years) including 2,389 nursing home residents, 4,765 and 633 older adults admitted acutely to geriatric or	N/A	On the whole, comorbidity indices did not perform well in our three settings, although the highest level of each index was associated with higher mortality.

For peer review only - http://bmjopen.bmj.com/site/about/guidelines.xhtml

					internal medicine wards		
Cahir et al. 2014	Potentially inappropriate prescribing and vulnerability and hospitalization in older community-dwelling patients	Ireland	Longitudinal (retrospective cohort)	• Multiple medications<br>• Focus on potentially inappropriate prescribing (compares Beers and STOPP criteria)	931 community-dwelling patients aged ≥70 years in 15 general practices in Ireland	N/A	• The prevalence by the Beers 2012 and STOPP criteria was 28% (n = 246) and 42% (n = 377), respectively. Patients with ≥2 PIP indicators were almost twice as likely to be classified as vulnerable.<br>• STOPP is a more sensitive measure of PIP than the Beers 2012 criteria and of clinical benefit in primary care settings, particularly for hospital visits.
Benson et al. 2021	Medication management for complex patients in primary care: application of a remote, asynchronous clinical pharmacist model	USA	Feasibility	• ≥ 5 medicines<br>• Focus on drug related problems (Indication, Effectiveness, Safety, and Compliance)	202 adults (aged 40+) with multimorbidity (≥2 chronic conditions) and high health expenditure	A remotely delivered Comprehensive Medication Management allowing pharmacists to make prescription changes (for 6 months)	• A clinical pharmacist found that 86% of participants had a drug therapy problem according to classification criteria. Seventy-nine percent of all drug therapy problems identified were resolved upon completion of the study.<br>• A service model using remote pharmacist services may be an effective means of improving team-based primary care medication management for this population.
Junius-Walker et al. 2021	MediQuit, an Electronic Deprescribing Tool for Patients on Polypharmacy: Results of a Feasibility Study in German General Practice	Germany	Feasibility	• Multiple medications<br>• ≥ 5 drugs<br>• Excessive polypharmacy = ≥ 10 drugs<br>• Inappropriate polypharmacy<br>• Focus on overprescribing and deprescribing	41 older adults (mean age 77 years) with at least 3 chronic diseases (mean was 8) with ≥ 10 drugs in 2 regions of Germany	MediQuit - Electronic Deprescribing Tool (3 steps: 1. Med review with algorithm to flag PIM for deprescribing. 2. Communcation prompts, and risk-benefits. 3. Information on discontinuation process).	• Deprescribing was achieved in 70% of consultations in agreement with patients.<br>• Drugs deprescribed were symptom-lowering and preventive drugs<br>• GPs found MediQuit useful in initiating communication on this issue and enhancing deliberations for a deprescribing decision.<br>• GPs rated patient involvement higher than did patients themselves.
McCarthy et al. 2022	Patient and general practitioner experiences of implementing a	Ireland	Evaluation (RCT)	• Multiple medicines use	404 older adults (≥65 years) taking ≥15 regular	SPPiRE website: educational module	• The SPPiRE intervention had a small effect in reducing the number of medicines and this was primarily mediated through the brown bag review.

For peer review only

	medication review intervention in older people with multimorbidity: Process evaluation of the SPPiRE trial			• Higher levels of polypharmacy ≥ 15 medicines<br>• Focus on potentially inappropriate prescribing (STOPP/START)	medicines from 51 GP practices	template for an individualised patient medication review that identified PIP, deprescribing opportunities, and patient priorities	• The context of resource shortages and deep-seated views around medical decision-making influenced intervention implementation.<br>• Intervention delivery varied among practices and 45 patients (28%) had no review, primarily due to insufficient GP time. GPs and patients responded positively to the intervention but most GPs did not engage with the patient priority-setting process.<br>• GPs identified a lack of integration into practice software and resources as barriers to future implementation.
Jager et al. 2017	A tailored programme to implement recommendations for multimorbid patients with polypharmacy in primary care practices-process evaluation of a cluster randomized trial	German y	Evaluation (RCT)	• ≥4 drugs	273 adults (>50 years) suffering from at least 3 chronic diseases, receiving more than 4 drugs, and being at high risk for medication-related events according to the assessment of the treating GP	The tailored programme consisted of a workshop for practice teams, elaboration of implementation action plans, aids for medication reviews, a multilingual info-tool for patients on a tablet PC, posters and brown paper bags as reminders for patients.	• The most frequently reported effect of the tailored programme was the increase of awareness for the health problem and the recommendations, while implementation of routine processes was only reported for structured medication counselling.<br>• Several modifications of the tailored programme may enhance its effectiveness such as conducting outreach visits instead of a workshop, improved targeting, provision of evidence, integration of tools into the practice software and information materials in tailored formats.
Ose et al. 2012	Let's talk about medication: concordance in rating medication adherence among multimorbid patients and their general practitioners	German y	Cross-sectional survey	• Multiple medications use<br>• Focus on adherence (self-rated)	92 patients with multiple chronic conditions at high-risk of hospitalisation (subjective) across 10 primary care practices from a region in Germany	N/A	• The percentage of concordance ranges between 40% (forgot to take medication) and 61% (deliberately omitted a dose).<br>• Talking about medication on a regular basis and better continuity of care may enhance patient – provider concordance in rating medication adherence as a prerequisite for shared decisions concerning medication in patients with multiple chronic conditions.

For peer review only

Sirois et al. 2019	The delicate choice of optimal basic therapy for multimorbid older adults: A cross-sectional survey.	Canada	Cross-sectional survey	• Many medications<br>• ≥10 medications	Older adults (65–75 years) with type 2 diabetes, chronic obstructive pulmonary disease and heart failure	N/A	• At least half of the participants (pharmacists and geriatricians) considered polypharmacy (≥10 medications) inevitable for an optimal basic treatment of DM, COPD and HF.<br>• The heterogeneity of responses raises issues when considering quality indicators in population-based studies.
Aubert et al. 2016	Polypharmacy and specific comorbidities in university primary care settings.	Switzerland	Cross-sectional study	• ≥ 5 long-term prescribed drugs<br>• potentially inappropriate prescribing and potential prescribing omission (using STOPP/START)	1002 patients aged 50–80 years followed in Swiss university primary care settings	N/A	Polypharmacy is common in university primary care settings, is strongly associated with hypertension, diabetes mellitus, chronic kidney disease and cardiovascular diseases, and increases potentially inappropriate prescribing.
Calderon-Larranaga et al. 2013	Polypharmacy patterns: unravelling systematic associations between prescribed medications	Spain	Cross-sectional study	• Multiple medicines<br>• Focus on polypharmacy patterns or clusters	79,089 people (≥15 years, mean 47 years)	N/A	• 7 patterns of polypharmacy were identified, which may be classified depending on the type of disease they are intended to treat: cardiovascular, depression-anxiety, acute respiratory infection, chronic obstructive pulmonary disease, rhinitis-asthma, pain, and menopause.<br>• Almost all of the patterns included drugs for preventing or treating potential side effects of other drugs in the same pattern.<br>• It demonstrated the existence of non-random associations in drug prescriptions.
Guisado-Clavero et al. 2019	Medication patterns in older adults with multimorbidity: a cluster analysis of primary care patients.	Spain	Cross-sectional study	Multiple drug use (examined in categories of 1-4, 5-9, and 10+ medications)	164,513 older adults (64-94 years) with multimorbidity (≥2 chronic diseases) within 50 primary healthcare centres in Barcelona	N/A	• Six medication patterns were identified, 5 of which were related to one or more anatomical group, with associations among drugs from different systems.<br>• Overall, guidelines do not accurately reflect the situation of the elderly multimorbid, new strategies for managing multiple drug uses are needed to optimize prescribing in these patients.

Lopez-Rodriguez et al. 2015	Potentially inappropriate prescriptions according to explicit and implicit criteria in patients with multimorbidity and polypharmacy. MULTIPAP: A cross-sectional study	Spain	Cross-sectional study	• Simultaneous use of several medicines.<br>• Potentially inappropriate medications (using both explicit and implicit criteria)	593 community-dwelling elderly aged 65 to 74 years, with multimorbidity and polypharmacy	N/A	• Potentially inappropriate prescribing was detected in 57.7%, 43.6%, 68.8% and 71% of 50 patients according to the explicit criteria STOPP 2014, STOPP 2008, Beers 2019 and Beers 2015 respectively.<br>• The MAI criteria detected greater inappropriateness than did the explicit criteria, but their application was more complex and difficult to automate.<br>• For every new drug taken by a patient, the MAI score increased by 2.41 (95% CI 1.46; 3.35) points.<br>• Diabetes, ischaemic heart disease and asthma were independently associated with lower summated MAI scores.
Lozano-Hernandez et al. 2020	Social support, social context and nonadherence to treatment in young senior patients with multimorbidity and polypharmacy followed-up in primary care. MULTIPAP Study	Spain	Cross-sectional study	• Simultaneous consumption of ≥ 5 drugs<br>• Potentially inappropriate medication<br>• Focus on adherence	593 older adults (between 65-74 years) with multimorbidity (≥3 diseases) and polypharmacy (≥5 drugs) during the last three months	MULTIPAP intervention: GPs e-training and GP-patient-centred interview.	• Among patients 65-74 years of age with multimorbidity and polypharmacy, lower functional support was related to nonadherence to treatment<br>• The nonadherence decreased in those patients with higher functional support, lower urban vulnerability, and higher perceived health status according to the visual analog scale of health-related quality of life
Rieckert et al. 2018	Polypharmacy in older patients with chronic diseases: a cross-sectional analysis of factors associated with excessive polypharmacy.	Austria, Germany, Italy, and UK	Cross-sectional study	excessive polypharmacy (taking ≥10 substances daily).	3904 older adults (aged 75+)	N/A	Frailty, multimorbidity, obesity, and decreased physical as well as mental health status are risk factors for excessive polypharmacy. Sex, educational level, and smoking apparently do not seem to be related to excessive polypharmacy.
Rogero-Blanco et al. 2020	Use of an Electronic Clinical Decision Support System in Primary Care to Assess Inappropriate Polypharmacy in Young Seniors With Multimorbidity:	Spain	Cross-sectional study	Focus on potentially inappropriate medications (Beers and STOPP/START)	593 older adults (aged 65-75) with multimorbidity (≥3 diseases) and polypharmacy (≥5 medications)	N/A	• There is a high prevalence of potentially inappropriate medications<br>• Females, taking a greater number of medicines, working in the primary sector, and being prescribed drugs for the central nervous system were related to a higher frequency

For peer review only

	Observational, Descriptive, Cross-Sectional Study						• The most common potentially inappropriate medications included benzodiazepines and prolonged use of proton pump inhibitors
Troncoso-Marino et al. 2021	Medication-related problems in older people in Catalonia: A real-world data study.	Spain	Cross-sectional study	Focus on medication-related problems<br>• Duplicate therapy<br>• Drug-drug interactions<br>• Potentially inappropriate medications (Beers, STOPP/START, PRISCUS)<br>• Drugs contraindicated in chronic kidney disease and in liver diseases	853 085 older adults (aged 65-99) with multimorbidity in 284 primary health care centres in Catalonia	N/A	• The coexistence of multimorbidity and polypharmacy is associated with an elevated risk of medication-related problems<br>• The most common potentially inappropriate drugs were those that increase the risk of fall (66.8%), antiulcer agents without criteria for gastroprotection (40.6%), and the combination of drugs with anticholinergic effects (39.7%).
Carrier et al. 2019	GPs' management of polypharmacy and therapeutic dilemma in patients with multimorbidity: a cross-sectional survey of GPs in France	France	Cross-sectional	• Many drugs at the same time or an excessive number<br>• No consensus on threshold or temporality<br>• Focus on unfavourable risk–benefit and potentially inappropriate prescribing	Older adults (54-82 years) with multimorbidity	Survey amongst 1266 GPs	• Nearly all (91.4%) responders felt comfortable or fairly comfortable deprescribing inappropriate medications, but only 34.7% decided to do so often or very often<br>• In therapeutic dilemmas, some GPs choose to prioritise patients' requests over iatrogenic risks.<br>• GPs need pragmatic implementation tools for handling therapeutic dilemmas, and to improve their skills in medication management and patient engagement in such situations.
Cooper et al. 2016	Potentially inappropriate prescribing in two populations with differing socio-economic profiles: a cross-sectional database study using the PROMPT criteria.	UK (NI), Ireland	Cross-sectional	• ≥4 repeat medicines<br>• Focus on Potentially inappropriate prescribing (PROMPT)	>750,000 middle-aged adults (45–64 years) in 2 populations with differing socio-economic profiles in Ireland	N/A	• Age group, female gender and polypharmacy were significantly associated with PIP in both populations and polypharmacy had the strongest association.<br>• PIP is common amongst middle-aged people with the risk of PIP increasing with polypharmacy.<br>• Differences in the prevalence of polypharmacy and PIP between the two populations may relate

For peer review only - http://bmjopen.bmj.com/site/about/guidelines.xhtml

							to heterogeneity in healthcare services and different socio-economic profiles, with higher rates of multimorbidity and associated polypharmacy in more deprived groups.
Gutierrez-Valencia et al. 2019	Prevalence of polypharmacy and associated factors in older adults in Spain: Data from the National Health Survey 2017	Spain	Cross-sectional	• polypharmacy (≥ 5 medications)<br>• hyperpolypharmacy (≥10)	7023 older adults (aged 65+) from National Health Survey of Spain	N/A	• The prevalence of polypharmacy in the elderly in primary care continues to increase and could be widely underestimated.<br>• Factors such as functional capacity or geriatric syndromes, fundamental in elderly people, modulate the habits of consumption and prescription of drugs in this population.
Moreira et al. 2020	Polypharmacy among adult and older adult users of primary care services delivered through the Unified Health System in Minas Gerais, Brazil.	Brazil	Cross-sectional	≥5 medications	1,159 interviewees distributed across 104 cities and 253 primary healthcare services	N/A	Understanding the medication use profile in primary health care allows identifying groups of people that are more likely to experience polypharmacy, and planning measures to mitigate potential polypharmacy-related problems.
Neuner-Jehle et al. 2017	Patient–provider concordance in the perception of illness and disease: a cross-sectional study among multimorbid patients and their general practitioners in Switzerland	Switzerland	Cross-sectional	• Multiple medications<br>• Focus on concordance, complexity and patient-centeredness	334 older adults (≥60 years, mean 76.2) with multimorbidity across 46 GPs in North Switzerland	N/A	• A majority of GPs perceive the CCs of the multimorbid patients correctly, but there is room for improvement.<br>• Concordance between CCs and diagnosis was 53.6%.<br>• The younger age and higher intake of drugs were significantly associated with an increased concordance between CCs and diagnosis.
Ong et al. 2018	Variation of polypharmacy in older primary care attenders occurs at prescriber level	Malaysia	Cross-sectional	• Concomitant use of≥ 5 medications<br>• Inappropriate medication use	22,832 Older adults (≥65 years) attending 2914 primary care clinics in Malaysia (nationwide)	N/A	• A total of 20.3% of the older primary care attenders experienced polypharmacy (26.7%% in public and 11.0% in private practice).<br>• The adjusted odds ratio (OR) of polypharmacy were 6.37 times greater in public practices. Polypharmacy was associated with patients of female gender (OR 1.49), primary education level (OR 1.61) and multimorbidity (OR 14.21).<br>• The variation in rate of polypharmacy was mainly found at prescriber level.

For peer review only

Payne et al 2014	Prevalence of polypharmacy in a Scottish primary care population	UK (Scotland)	Cross-sectional	• multiple medications by a single patient	180,815 adult (≥20 years) patients permanently registered in 40 Scottish GP surgeries.	N/A	• Polypharmacy is common and significantly associated with multimorbidity, although considerable variation exists between different conditions.<br>• The impact of clinical conditions on the number of medicines is generally less in the presence of co-existing concordant conditions.
Rogero-Blanco et al. 2021	Drug interactions detected by a computer-assisted prescription system in primary care patients in Spain: MULTIPAP study	Spain	Cross-sectional	• Simultaneous consumption of ≥ 5 drugs<br>• Potentially inappropriate medication<br>• Focus on drug-drug interactions	593 older adults (between 65-74 years) with multimorbidity (≥3 diseases) and polypharmacy (≥5 drugs) during the last three months	MULTIPAP intervention: GPs e-training and GP-patient-centred interview.	• Half of the patients had at least one relevant drug interaction<br>• Factors associated with drug interactions were the use of more than 10 drugs (OR 11.86) and having anxiety/depressive disorder (OR 1.98), with protective factors against as hypertension, diabetes, and ischaemic heart disease
Khatter et al. 2020	Prevalence and predictors of potentially inappropriate prescribing in middle-aged adults: a repeated cross-sectional study	UK	Cross sectional	• ≥4 repeat medicines<br>• Focus on Potentially inappropriate prescribing (PROMPT)	>50,000 middle aged adults (45–64 years taking ≥1 medicine.	N/A	• The prevalence of PIP decreased from 20% in 2014 to 18% in 2019.<br>• The most prevalent PROMPT criteria were the use of ≥2 drugs from the same pharmacological class (7.6%), use of non-steroidal anti-inflammatory drugs for >3 months (7.1%) and use of proton pump inhibitors above recommended maintenance dosages for >8 weeks (3.1%).<br>• Over the study period, the prevalence of multimorbidity increased (47–52%) and polypharmacy was stable (27%).<br>• Polypharmacy, multimorbidity, deprivation, and age were independently associated with PIP. Sex was the only variable not associated.
Burt et al. 2018	Developing a measure of polypharmacy appropriateness in primary care: systematic review and expert consensus study.	UK	Consensus study (with review)	• Use of multiple medications in a single individual<br>• Focus on polypharmacy appropriateness	N/A	N/A	• Produced a set of 12 indicators of clinical importance considered relevant to polypharmacy appropriateness.<br>• Panel members particularly valued indicators concerned with adverse drug reactions, contraindications, drug-drug interactions, and the conduct of medication reviews.

For peer review only

Cooper et al. 2014	The development of the PROMPT (PRescribing Optimally in Middle-aged People's Treatments) criteria	UK and Ireland	Consensus study (with review)	Focus on potentially inappropriate prescribing	Middle-aged adults (45-64 years)	N/A	• PROMPT is the first set of prescribing criteria developed for use in middle-aged adults<br>• A final list of 22 criteria reached consensus after two rounds
George et al. 2004	Development and Validation of the Medication Regimen Complexity Index	Australi a	Consensus study (with review)	• Use of multiple medications<br>• Focus on complexity	N/A	N/A	• A 65–item Medication Regimen Complexity Index (MRCI) was developed.<br>• The total MRCI score had significant correlation with the number of drugs in the regimen, but not with the age and gender of the patients.
Basger et al. 2012	Validation of prescribing appropriateness criteria for older Australians using the RAND/UCLA appropriateness method	Australi a	Consensus study	• Use of multiple medications<br>• Focus on prescribing appropriateness	N/A	N/A	• A set of 41 Australian prescribing appropriateness criteria were validated by an expert panel.<br>• Use of these criteria, together with clinical judgement and other medication review processes such as patient interview, is intended to assist in improving patient care by efficiently detecting potential drug related problems related to commonly occurring medicines
Mann et al. 2022	Development of a deprescribing manual for frail older people for use in the COFRAIL study and in primary care	German y	Consensus study	• ≥5 medications<br>• Focus on deprescribing	Frail older patients (≥65 years	Structured manual to support deprescribing of medicines in frail, older adults in primary care	• After piloting and revisions, the deprescribing manual now covers 11 indications/topics.<br>• In each chapter, patient- and medication-related deprescribing criteria, monitoring and communication strategies, and information about concerns related to the use of specific drugs in older patients are provided.
Turner et al. 2016	What factors are important for deprescribing in Australian long-term care facilities? Perspectives of residents and health professionals	Australi a	Consensus study	• ≥5 medications<br>• Appropriate and inappropriate prescribing<br>• Focus on deprescribing of unnecessary or potentially inappropriate medications	Residents of long-term care facilities with multimorbidity and polypharmacy across metropolitan and regional South Australia.	Nominal group methods with 19 GPs, 12 nurses, 14 pharmacists, and 11 patients and carers	• No two groups had the same priorities. GPs ranked 'evidence for deprescribing' and 'communication with family/resident' as most important factors<br>• Pharmacists ranked 'clinical appropriateness of therapy' and 'identifying residents' goals of care' as most important.<br>• The multidisciplinary groups prioritised both 'adequacy of medical and medication history' and 'identifying goals of care'.

For peer review only

Molist-Brunet et al. 2022	Improving individualized prescription in patients with multimorbidity through medication review	Spain	Before and after study	• ≥5 medications continuously<br>• severe or excessive polypharmacy: ≥10 chronic medications	428 older adults (≥65 years, mean age 85.5) with multimorbidity (≥2 morbidities) across 3 primary care centres and 3 nursing homes.	Medication review by an interdisciplinary team (primary care team, consultant geriatrician and clinical pharmacist) by applying the Patient-Centered Prescription model (based on CGA) to align the treatment with care goals.	• An individualized medication review in frail older patients, applying the Patient-Centered Prescription model, decreases pharmacological parameters related to adverse drug effects, such as polypharmacy, therapeutical complexity, and anticholinergic and, or sedative burden.<br>• A decrease in polypharmacy, medication regimen complexity index, and drug burden index was more frequent among frail patients, especially those with severe frailty
San-Jose et al. 2021	Integrated health intervention on polypharmacy and inappropriate prescribing in elderly people with multimorbidity: Results at the end of the intervention and at 6 months after the intervention	Spain	Before and after study	• ≥ 5 medicines<br>• Focus on potentially inappropriate prescribing (STOPP/START)	100 older adults (≥ 65 years) with high multimorbidity according to Adjusted Morbidity Groups (AMG 3-4) with a recent unplanned hospital visit (including ED)	A CGA and appropriate prescribing review of medicines were performed by the team (one GP, one medical doctor, an Advanced Practice Nurse and a day nurse), then a care plan was developed.	• An integrated health intervention centred on polypharmacy in elderly people improves inappropriate prescribing that persists beyond the intervention.<br>• The proportion of patients with two or more STOPP criteria reduced from 37% at the beginning of the intervention to 18% at the end, and the proportion of those with START criteria from 13% to 6%. These differences persisted at 6 months.

Preferred Reporting Items for Systematic reviews and Meta-Analyses extension for Scoping Reviews (PRISMA-ScR) Checklist for:

Understanding, identifying, and addressing problematic polypharmacy within multimorbidity in primary care: A scoping review

SECTION	ITEM	PRISMA-ScR CHECKLIST ITEM	REPORTED ON PAGE #
**TITLE**			
Title	1	Identify the report as a scoping review.	1
**ABSTRACT**			
Structured summary	2	Provide a structured summary that includes (as applicable): background, objectives, eligibility criteria, sources of evidence, charting methods, results, and conclusions that relate to the review questions and objectives.	2
**INTRODUCTION**			
Rationale	3	Describe the rationale for the review in the context of what is already known. Explain why the review questions/objectives lend themselves to a scoping review approach.	4-5
Objectives	4	Provide an explicit statement of the questions and objectives being addressed with reference to their key elements (e.g., population or participants, concepts, and context) or other relevant key elements used to conceptualize the review questions and/or objectives.	5
**METHODS**			
Protocol and registration	5	Indicate whether a review protocol exists; state if and where it can be accessed (e.g., a Web address); and if available, provide registration information, including the registration number.	N/A
Eligibility criteria	6	Specify characteristics of the sources of evidence used as eligibility criteria (e.g., years considered, language, and publication status), and provide a rationale.	6-7
Information sources*	7	Describe all information sources in the search (e.g., databases with dates of coverage and contact with authors to identify additional sources), as well as the date the most recent search was executed.	5-6
Search	8	Present the full electronic search strategy for at least 1 database, including any limits used, such that it could be repeated.	5-6
Selection of sources of evidence†	9	State the process for selecting sources of evidence (i.e., screening and eligibility) included in the scoping review.	7-8
Data charting process‡	10	Describe the methods of charting data from the included sources of evidence (e.g., calibrated forms or forms that have been tested by the team before their use, and whether data charting was done independently or in duplicate) and any processes for obtaining and confirming data from investigators.	8
Data items	11	List and define all variables for which data were sought and any assumptions and simplifications made.	8
Critical appraisal of individual sources of evidence§	12	If done, provide a rationale for conducting a critical appraisal of included sources of evidence; describe the methods used and how this information was used in	6

For peer review only - http://bmjopen.bmj.com/site/about/guidelines.xhtml

SECTION	ITEM	PRISMA-ScR CHECKLIST ITEM	REPORTED ON PAGE #
		any data synthesis (if appropriate).	
Synthesis of results	13	Describe the methods of handling and summarizing the data that were charted.	8
**RESULTS**			
Selection of sources of evidence	14	Give numbers of sources of evidence screened, assessed for eligibility, and included in the review, with reasons for exclusions at each stage, ideally using a flow diagram.	8, figure 1
Characteristics of sources of evidence	15	For each source of evidence, present characteristics for which data were charted and provide the citations.	Supplementary file 1
Critical appraisal within sources of evidence	16	If done, present data on critical appraisal of included sources of evidence (see item 12).	N/A
Results of individual sources of evidence	17	For each included source of evidence, present the relevant data that were charted that relate to the review questions and objectives.	Supplementary file 1
Synthesis of results	18	Summarize and/or present the charting results as they relate to the review questions and objectives.	8-19
**DISCUSSION**			
Summary of evidence	19	Summarize the main results (including an overview of concepts, themes, and types of evidence available), link to the review questions and objectives, and consider the relevance to key groups.	19-20
Limitations	20	Discuss the limitations of the scoping review process.	21-22
Conclusions	21	Provide a general interpretation of the results with respect to the review questions and objectives, as well as potential implications and/or next steps.	22
**FUNDING**			
Funding	22	Describe sources of funding for the included sources of evidence, as well as sources of funding for the scoping review. Describe the role of the funders of the scoping review.	23

JBI = Joanna Briggs Institute; PRISMA-ScR = Preferred Reporting Items for Systematic reviews and Meta-Analyses extension for Scoping Reviews.
* Where *sources of evidence* (see second footnote) are compiled from, such as bibliographic databases, social media platforms, and Web sites.
† A more inclusive/heterogeneous term used to account for the different types of evidence or data sources (e.g., quantitative and/or qualitative research, expert opinion, and policy documents) that may be eligible in a scoping review as opposed to only studies. This is not to be confused with *information sources* (see first footnote).
‡ The frameworks by Arksey and O'Malley (6) and Levac and colleagues (7) and the JBI guidance (4, 5) refer to the process of data extraction in a scoping review as data charting.
§ The process of systematically examining research evidence to assess its validity, results, and relevance before using it to inform a decision. This term is used for items 12 and 19 instead of "risk of bias" (which is more applicable to systematic reviews of interventions) to include and acknowledge the various sources of evidence that may be used in a scoping review (e.g., quantitative and/or qualitative research, expert opinion, and policy document).

From: Tricco AC, Lillie E, Zarin W, O'Brien KK, Colquhoun H, Levac D, et al. PRISMA Extension for Scoping Reviews (PRISMAScR): Checklist and Explanation. Ann Intern Med. 2018;169:467–473. doi: 10.7326/M18-0850.

For peer review only - http://bmjopen.bmj.com/site/about/guidelines.xhtml

Defining, identifying, and addressing problematic polypharmacy within multimorbidity in primary care: A scoping review

Journal:	*BMJ Open*
Manuscript ID	bmjopen-2023-081698.R1
Article Type:	Original research
Date Submitted by the Author:	19-Mar-2024
Complete List of Authors:	Tsang, Jung Yin; The University of Manchester Division of Population Health Health Services Research and Primary Care, Centre for Primary Care and Health Services Research, School of Health Sciences; The University of Manchester, NIHR Greater Manchester Patient Safety Research Collaboration (GMPSRC), Faculty of Biology, Medicine and Health, Manchester Academic Health Sciences Centre (MAHSC)<br>Sperrin, Matthew; The University of Manchester, Division of Informatics, Imaging and Data Sciences, School of Health Sciences; The University of Manchester, NIHR Greater Manchester Patient Safety Research Collaboration (GMPSRC), Faculty of Biology, Medicine and Health, Manchester Academic Health Sciences Centre (MAHSC)<br>Blakeman, Thomas; The University of Manchester Division of Population Health Health Services Research and Primary Care, Centre for Primary Care and Health Services Research, School of Health Sciences; The University of Manchester, NIHR Greater Manchester Patient Safety Research Collaboration (GMPSRC), Faculty of Biology, Medicine and Health, Manchester Academic Health Sciences Centre (MAHSC)<br>Payne , Rupert A ; University of Exeter Medical School, Department of Health and Community Sciences<br>Ashcroft, Darren; The University of Manchester Faculty of Biology Medicine and Health, Division of Pharmacy and Optometry, School of Health Sciences; The University of Manchester, NIHR Greater Manchester Patient Safety Research Collaboration (GMPSRC), Faculty of Biology, Medicine and Health, Manchester Academic Health Sciences Centre (MAHSC)
**Primary Subject Heading**:	General practice / Family practice
Secondary Subject Heading:	Evidence based practice, Geriatric medicine, Patient-centred medicine, Medical management, Health services research
Keywords:	Drug Combinations, Chronic Disease, Adverse events < THERAPEUTICS, Primary Health Care, Primary Prevention, Medication Adherence

For peer review only - http://bmjopen.bmj.com/site/about/guidelines.xhtml

SCHOLARONE™
Manuscripts

I, the Submitting Author has the right to grant and does grant on behalf of all authors of the Work (as defined in the below author licence), an exclusive licence and/or a non-exclusive licence for contributions from authors who are: i) UK Crown employees; ii) where BMJ has agreed a CC-BY licence shall apply, and/or iii) in accordance with the terms applicable for US Federal Government officers or employees acting as part of their official duties; on a worldwide, perpetual, irrevocable, royalty-free basis to BMJ Publishing Group Ltd ("BMJ") its licensees and where the relevant Journal is co-owned by BMJ to the co-owners of the Journal, to publish the Work in this journal and any other BMJ products and to exploit all rights, as set out in our licence.

The Submitting Author accepts and understands that any supply made under these terms is made by BMJ to the Submitting Author unless you are acting as an employee on behalf of your employer or a postgraduate student of an affiliated institution which is paying any applicable article publishing charge ("APC") for Open Access articles. Where the Submitting Author wishes to make the Work available on an Open Access basis (and intends to pay the relevant APC), the terms of reuse of such Open Access shall be governed by a Creative Commons licence – details of these licences and which Creative Commons licence will apply to this Work are set out in our licence referred to above.

Other than as permitted in any relevant BMJ Author's Self Archiving Policies, I confirm this Work has not been accepted for publication elsewhere, is not being considered for publication elsewhere and does not duplicate material already published. I confirm all authors consent to publication of this Work and authorise the granting of this licence.

For peer review only

Defining, identifying, and addressing problematic polypharmacy within multimorbidity in primary care: A scoping review

Jung Yin Tsang [1,2,3]
Matthew Sperrin [2,3]
Thomas Blakeman [1,2]
Rupert A Payne [4]
Darren M Ashcroft [2,5]

Authors and affiliations:
[1]Centre for Primary Care and Health Services Research, School of Health Sciences, University of Manchester, UK
[2]NIHR Greater Manchester Patient Safety Research Collaboration (GMPSRC), Faculty of Biology, Medicine and Health, Manchester Academic Health Sciences Centre (MAHSC), University of Manchester, UK
[3]Division of Informatics, Imaging and Data Sciences, School of Health Sciences, University of Manchester, UK
[4]Department of Health and Community Sciences, University of Exeter Medical School, Exeter, United Kingdom
[5]Division of Pharmacy and Optometry, School of Health Sciences, University of Manchester, UK

Corresponding author:
Jung Yin Tsang
jungyin.tsang@manchester.ac.uk
Centre for Primary Care and Health Services Research, 6th Floor Williamson building, Oxford Road, University of Manchester, Manchester, UK, M13 9PL
Tel: 0161 275 0752

Word count: 3998 words
Key words: Polypharmacy; multimorbidity; Overtreatment; Inappropriate Prescribing; Deprescriptions; Adverse Drug Reaction; Drug Side Effects.

For peer review only

ABSTRACT

Introduction

Polypharmacy and multimorbidity pose escalating challenges. Despite numerous attempts, interventions have yet to show consistent improvements in health outcomes. A key factor may be varied approaches to targeting patients for intervention.

Objectives

To explore how patients are targeted for intervention by examining the literature with respect to understanding how polypharmacy is defined, identifying problematic polypharmacy in practice, and addressing problematic polypharmacy through interventions

Design

Scoping review as defined by the Joanna Briggs Institute (JBI)

Setting

Primary care

Data sources

Medline, Embase, CINAHL, and Cochrane along with ClinicalTrials.gov, Science.gov and WorldCat.org from January 2004 to February 2024

Eligibility criteria

We included all articles that had a focus on problematic polypharmacy in multimorbidity and primary care, incorporating multiple types of evidence, such as reviews, quantitative trials, qualitative studies, and policy documents. Articles focussing on a single index disease or not written in English were excluded.

For peer review only

Extraction and Analysis

We performed a narrative synthesis, comparing themes and findings across the collective evidence to draw contextualised insights and conclusions.

Results

In total, 157 articles were included. Case-finding methods often rely on basic medication counts (often 5 or more) without considering past medical history or whether individual medications are clinically appropriate. Other approaches highlight specific drug indicators and interactions as potentially inappropriate prescribing, failing to capture a proportion of patients not fitting criteria. Different potentially inappropriate prescribing criteria also show significant inconsistencies in determining the appropriateness of medications, often neglecting to consider multimorbidity and underprescribing. This may hinder the identification of the precise population requiring intervention.

Conclusions

Improved strategies are needed to target patients with polypharmacy, which should consider patient perspectives, individual factors, and clinical appropriateness. The development of a cross-cutting measure of problematic polypharmacy that consistently incorporates adjustment for multimorbidity may be a valuable next step to address frequent confounding.

For peer review only

For peer review only - http://bmjopen.bmj.com/site/about/guidelines.xhtml

STRENGTHS AND LIMITATIONS OF THIS STUDY

- This is the first scoping review to explore and conceptualise how patients with problematic polypharmacy are targeted for intervention
- It includes multiple types of evidence, including systematic reviews, quantitative, qualitative, and mixed methods studies, along with policy documents.
- Our synthesis capitalises on the shared challenges involved in managing both polypharmacy and multimorbidity with a greater focus on articles regarding polypharmacy in chronic conditions. Rather than acute medication adjustments.
- It was not always possible to separate results in studies encompassing both primary and secondary care

For peer review only

INTRODUCTION

Polypharmacy in multimorbidity is an increasing global priority.[1] With an ageing population, over a quarter of the population are living with Multiple Long Term Conditions (MLTC), i.e. multimorbidity, often associated with polypharmacy, which is broadly defined as the use of multiple medications.[2] Medications carry clear benefits, yet the use of multiple medicines can be linked to adverse consequences, including increased treatment burden, unplanned hospitalisation and death.[3,4] For single conditions, people with more severe disease often require more medications. For example, the National Institute for Health and Care Excellence (NICE) guidelines recommend six medicines to be initiated post-myocardial infarction for secondary prevention.[5] Yet in multimorbidity, the number of medicines quickly add up, with limited evidence of benefit over risk as this population is frequently excluded in trials.[6] As the number of medicines prescribed increases, so does the direct risk of adverse drug reactions, increasing health service costs and utilisation, reducing adherence, and decreasing quality of life.[7-9] This can be particularly problematic for older patients, for whom prescribing is more common and thus associated with greater possibility of prescribing errors. Moreover the risks of harms are increased due to changes in pharmacokinetics (e.g. impaired drug metabolism, changes in drug binding) and pharmacodynamics (e.g. increased sensitivity to adverse effects).[10-12] Problematic polypharmacy has previously been defined as "the prescribing of multiple medications inappropriately, or where the intended benefit of the medication is not realised."[3]

Despite numerous interventions targeting polypharmacy, there remains little evidence of improvement of health outcomes, such as hospitalisations and death.[13-15] However, some reductions in inappropriate prescribing have been observed. Successes of these interventions were highly variable and greatly affected by differences in implementation and targeting of

For peer review only - http://bmjopen.bmj.com/site/about/guidelines.xhtml

patients.[13-15] Further conceptualising the complex and varied approaches to targeting patients with problematic polypharmacy and multimorbidity may inform empirical research and improve future intervention design.[2] Therefore, a scoping review was performed, as this is an effective approach for assessing a broad evidence base. This review centres on considering the pivotal role of primary care professionals and capitalises on the shared challenges involved in managing polypharmacy and multimorbidity. The overarching aim of the review was to explore how patients are targeted for intervention by examining the literature with respect to 1) understanding how polypharmacy is defined; 2) identifying problematic polypharmacy in practice; and 3) addressing problematic polypharmacy through interventions.

METHODS

A scoping review as defined by the Joanna Briggs Institute (JBI) was performed consistent with the Preferred Reporting Items for Systematic Reviews extension for Scoping Reviews (PRISMA-ScR) guidance.[16] This allowed an exploration of both breadth and depth of the topic, which was imperative given the complexity and heterogeneity of evidence. We purposely retained multiple types of evidence (e.g. randomised controlled trials (RCT), consensus trials, qualitative video ethnography) to allow learning through quantitative, qualitative, mixed methods studies, as well as policy and grey literature, to increase relevance and examine the latest evidence base to date.

Search Strategy

A literature search was conducted within MEDLINE, Embase, Cumulative Index to Nursing and Allied Health Literature (CINAHL) and Cochrane Database of Systematic Reviews in Jan 2023. Search terms were developed after a preliminary search of articles covering our

For peer review only - http://bmjopen.bmj.com/site/about/guidelines.xhtml

population, concept and context of interest, provided in table 1. This included the population of people with multimorbidity, the concept of problematic polypharmacy, and the context of primary care. We limited our final search strategy to include only articles from 2004 onwards based on the earliest date of relevant articles from a preliminary search. Three additional databases were then searched for grey literature and clinical trial records: ClinicalTrials.gov, Science.gov and WorldCat in Feb 2023. We then followed an iterative process of snowballing through a supplementary search of references, citation lists and related articles using Google Scholar. Consistent with scoping reviews guidance, critical appraisal was not undertaken. An updated search was then completed in Feb 2024.

Table 1: Search terms used

**Category**	**Search terms used**
Multimorbidity	Multimorbid* or multiple long-term conditions or multiple health conditions
Polypharmacy	Polypharmacy or polypharmacotherapy or hyperpolypharmacy or polymedicine* or polimedicin* or multiple medic* or multimedic* or inappropriate prescrib* or overprescrib* or underprescrib* or deprescrib*
Primary Care	Primary care or primary healthcare or general practi*

Eligibility criteria

The eligibility criteria with typical exclusion examples are presented in table 2, guided by the Population, Concept and Context framework recommended by PRISMA-ScR:[16]

- Population – Adults living with multimorbidity
- Concept – Problematic polypharmacy
- Context – Primary Care

For peer review only - http://bmjopen.bmj.com/site/about/guidelines.xhtml

Table 2: Eligibility criteria and typical exclusion examples

Inclusion criteria	Typical exclusion examples
Population<br>• Studies must include adults (18 years and older)<br>• Studies must focus on those with multimorbidity – defined as 2 or more long-term conditions, not linked to an “index disease”	• Studies focussing on diabetic patients with renovascular disease (I.e. has an index condition of diabetes)
Concept<br>• Studies focussing on polypharmacy – defined as the concurrent use of multiple medications<br>• Studies that consider the long-term clinical impact of multiple medicines<br>• Studies that consider the consequences of multiple medicines or the ‘problematic’ element of polypharmacy	• Studies focussed on single medications<br>• Studies based on prescribing of antibiotics for acute presentations only<br>• Studies that are simply descriptive of the number of tablets taken and do not report any risk factors, outcomes, or consequences
Context<br>• Studies with relevance to primary care, including studies which crossed the primary-secondary care interface.	• Studies solely on hospital-based pharmacists
Study type<br>• Studies written in English<br>• Studies presenting full descriptions of the research (e.g. research studies, systematic reviews, randomised controlled trials, pilot studies, and policy documents)	• Letters, comments, conference abstracts, protocols, proceedings, etc.

For peer review only

Study selection

Studies meeting the inclusion criteria were initially selected, based on screening the titles, abstracts, and subsequent full papers by one researcher (JT). A random selection of 10% the records were analysed independently by a second researcher (TB) with 97% agreement of inclusion. Disagreements were resolved through discussion with the wider team.

Data Extraction and Analysis

The data were extracted from eligible studies using a standardised data extraction form and included the author, year of publication, country of origin, type of the publication, polypharmacy definitions, type of participants, descriptions of interventions (if applicable), and key findings (see additional file 1). Further elaboration of the extracted data involved grouping studies according to their focus on either defining, identifying, and addressing polypharmacy, with some spanning multiple elements. The main analysis took the form of a narrative synthesis, using mainly qualitative descriptive data consistent with PRISMA-ScR guidance.[16] This compared themes and findings from grouped studies and using the collective evidence to draw contextualised insights and conclusions.

RESULTS

The search yielded 727 unique articles, with the process illustrated in figure 1. During eligibility screening 486 were excluded after assessment of the abstract and 84 further full-text articles were excluded. A total of 157 articles were included in the final synthesis (supplementary file 1), of which 19 were added during the updated search. This included 52 meta-analyses and reviews, 55 quantitative (including nine RCTs and 19 longitudinal analyses), 36 qualitative studies (including six consensus studies and two RCT evaluations), nine pilot or feasibility studies, and five policy documents. The literature was varied with international articles covering a range of polypharmacy issues, from definitions to interventions, with some focussing on sub-populations with multimorbidity (e.g. frailty) and subcategories within the broader context of primary care (e.g. residential care facilities).

[INSERT FIGURE 1]

For peer review only - http://bmjopen.bmj.com/site/about/guidelines.xhtml

Understanding how polypharmacy is defined

Numerous polypharmacy definitions

There is no consensus on a definition for polypharmacy, with significant variations in approaches to targeting problematic polypharmacy.[2,3,17] Over 100 definitions of polypharmacy have been used, reflecting the discordance of approaches.[18,19] Two main approaches to defining polypharmacy can be grouped into quantitative (utilising a form of medication count) and qualitative definitions (utilising descriptive notions of prescribing quality), with some studies utilising a combination of these definitions. Table 3 gives illustrative examples of these definitions.

Table 3: Illustrative list of examples for polypharmacy definitions

Definitions	Descriptions/ Examples
**Quantitative definitions**	
Single cut-offs of medication count	≥2, ≥3, ≥4, ≥5, ≥8, ≥10, ≥11 or ≥20 medications
Single cut-offs of a medication group	>2 anticholinergic medications<br>>3 antipsychotic medications
Groups of medication counts	0–4 medications, 5–9 medications, 10–14 medications, ≥15 medications<br>0–5 medications, 6–8 medications, 9–11 medications, ≥12 medications<br>0–6 medications, 7–9 medications, 10–13 medications, ≥14 medications
Categorisation with levels or attributes	Mild polypharmacy 1–4 or 2–3 medications<br>Minor polypharmacy 2–4 medications<br>Major polypharmacy ≥5 medications<br>Standard polypharmacy 5–9 or 6-9 medications<br>Severe polypharmacy ≥6 or ≥10 medications<br>Extreme polypharmacy ≥10 medications<br>Hyper-polypharmacy ≥10 medications<br>High-level polypharmacy ≥10 medications
**Qualitative definitions**	
Overprescribing	More medications than clinically indicated or unnecessary medications or presence of medications with no clinical indications or for which a safer alternative exists
Underprescribing	Lack of an indicated medication, or prescribed an inadequate amount or prescribed less frequent than appropriate
Drug-drug interactions	Any potential interaction, or harmful combination
Inappropriate medications	Defined by set criteria, for example overprescribing, mis-prescribing and potential interactions
Prescribing cascade	Medication prescribed to treat the side effect of another medication

For peer review only - http://bmjopen.bmj.com/site/about/guidelines.xhtml

Absence of indication	Medication not matching the diagnosis
Therapeutic duplication	Same medicine used more than once or two within same therapeutic group used (e.g. multiple antidepressants)
No therapeutic benefit	Medications with lack of effectiveness
Not cost-effective	Availability of an equally effective, lower-cost alternative

Illustrative examples of wide range of definitions for polypharmacy used in the literature.[18,19] Generally, quantitative definitions focus on operationalising medication count, regardless of whether polypharmacy is problematic. Whereas most qualitative definitions attach descriptors to describe scenarios where polypharmacy may be clinically problematic.

Quantitative definitions of polypharmacy were more frequent, with over 90% of publications utilising some form of medication count.[2,18-21] For example, the World Health Organization defines polypharmacy as four or more medicines, academic studies most commonly use five or more.[1,2] Other quantitative definitions included categorisations rather than cut offs of medication count. These were frequently labelled levels (e.g. mild, moderate and severe) or attributes (e.g. excessive, extreme), yet counts within these categories were also inconsistent.[12,18,19,22-24] Generally, quantitative definitions were easier to operationalise and more reproducible, with a focus on medication count, regardless of whether polypharmacy is problematic. In contrast, qualitative definitions largely required clinical judgement to evaluate prescribing quality, carrying a focus on when polypharmacy becomes problematic. This frequently highlighted the overuse or overprescribing of medications. But definitions also covered aspects of mis-prescribing, often through applying a list of defined prescribing criteria, and also under-prescribing, though only a few studies emphasised this aspect. The terms ‘appropriate’, ‘inappropriate’ and ‘problematic’ polypharmacy were also commonly used to describe when multiple medications were justified compared to when the clinical indication was unclear.[3,18,19,25] These definitions have now been expanded to cover further dimensions of polypharmacy, such as the increasing recognition of the importance of patient and carer input in determining the appropriateness of medications.[26,27]

For peer review only

For peer review only - http://bmjopen.bmj.com/site/about/guidelines.xhtml

The challenges of defining when polypharmacy is 'problematic'

The understanding of polypharmacy has progressed over time, with an increasing shift to more clinically applicable definitions. This reflects the increasing complexity of decision making for combinations of medicines tailored to individual needs. There is also recognition that it is not possible to account for clinical appropriateness through simple medication counts.[18,19] Commonly people with multiple health needs may well be appropriately prescribed more than 10 medications for therapeutic and symptomatic benefit, which would be termed extreme polypharmacy in some studies and guidelines.[28,29] Yet there is some validity to numeric approaches as increasing medications are strongly associated with drug related problems, and very high counts of medication are usually questionable.[30] There is also a need to improve the consistency of reporting medication exposure characteristics.[18,19,31-33] Various definitions have been used to define temporality and 'long-term' use, with some publications including 'acute' and 'as required' medications as opposed to chronic medications, with varied definition of time periods (ranging from 1-240 days).[18,19] Terms such as problematic polypharmacy and inappropriate polypharmacy have been increasingly favoured, as they consider appropriateness and clinical decision-making.[34] Yet qualitative research suggest these labels were still insufficient to reflect the complexity of medicines management, with practitioners juggling terms such as "potentially inappropriate" and "specifically appropriate" and others considering them "judgemental" and even "accusatory".[35]

For peer review only - http://bmjopen.bmj.com/site/about/guidelines.xhtml

Identifying problematic polypharmacy in practice

Targeting potentially high-risk populations

Various strategies target higher risk populations to try and identify problematic polypharmacy. One common approach use simple cut offs of age (commonly ≥ 65 years) combined with cut offs of medications (frequently ≥ 5) and this was the main inclusion criterion for the majority of trials.[13] Another approach adopted by multiple national recommendations advocate case finding through high risk groups.[36-38] For instance, both NICE guidelines and the Australian Commission on Safety and Quality in Healthcare recommend greater attention for older people with frailty, and complex multimorbidity and co-existing mental and physical health problems.[2,29,36] Accordingly, several national indicators, initiatives and studies also use combinations of these approaches.[36-43] Other approaches include risk scores to identify patients at high-risk of particular outcomes (e.g. hospitalisations, or adverse drug reactions) but these require further development.[44,45] Overall, strategies to identify potentially high-risk populations currently demonstrate variable validity in polypharmacy and are seldom comprehensive or holistic, as they are specific to the needs of particular groups.[2,36,46]

Targeting potentially inappropriate medicines

Evaluating the appropriateness of individual medications is a common approach both as a case-finding approach and as a surrogate measure of prescribing quality across polypharmacy. Various tools have been developed to identify potentially inappropriate medicines and these can be split into explicit and implicit tools, with some tools combining both (examples in table 4).[47-49] The majority have been developed using expert opinion and consensus methodology, and originally were designed for evaluating individual medications, rather than polypharmacy as a whole.[47,50] Explicit tools contain specific criteria or scenarios leading to potential adverse drug events and carry advantages of reproducibility and ease of automation.[51-55]

For peer review only

Implicit tools require judgement, which means they can be subjective and demand more time and clinical expertise. Nevertheless, explicit tools are limited to specific drugs and diseases, but implicit tools can be applied to any medication. This perhaps allows implicit tools greater applicability in polypharmacy, as explicit tools will miss out any medicines outside criteria.[56]

Table 4: Key examples of explicit and implicit tools of appropriate prescribing

Tool	Description	Strengths	Limitations
**Beers Criteria** *(Explicit tool)*	- First widely used explicit criteria<br>- Contains over 200 criteria (2023 version) including potentially inappropriate medications to be avoided such as drug-disease and drug-drug interactions, particularly in older adults.	- International studies have shown predictive validity for adverse drug reactions, falls, cognitive function, hospitalisation and death.<br>- Endorsed by the American Geriatric Society and updated approximately every 3-4 years.<br>- Easier to automate in drug records as criteria are very specific	- No positive clinical outcomes in RCTs to date<br>- No prioritisation of medications for review<br>- Can be challenging to use as long list of criteria<br>- Does not address under-prescribing<br>- Focus is on individual medications rather than polypharmacy as a whole
**Screening Tool of Older Person's Prescriptions/ Screening Tool to Alert doctors to Right Treatment – STOPP/START** *(Explicit tool, but newer versions also contain implicit measures)*	- One of the most widely used explicit criteria globally for older adults<br>- Contains 133 criteria for potentially inappropriate medications, and 57 potential under-prescribing criteria (version 3), organised according to medication and disease groups	- Some positive outcomes shown in several RCTs<br>- Also addresses aspects of under-prescribing in addition to overprescribing<br>- Easier to automate in computerised drug records as most criteria are very specific	- Misses out medications out of criteria<br>- Potentially usability issues with such a long list<br>- No prioritisation of medications for review<br>- Can be challenging to use as long list of criteria<br>- Focus is on individual medications rather than polypharmacy as a whole
**Medication Appropriateness Index – MAI** *(Implicit tool)*	- First widely used implicit criteria<br>- Lists 10 criteria that evaluate various aspects of medication appropriateness (e.g. indication, effectiveness, dose)	- Some positive outcomes shown in several RCTs<br>- Can be applied to all medicines	- Time-consuming to execute<br>- Requires clinical expertise and can be subjective<br>- Difficult to automate<br>- No prioritisation of medications for review<br>- Focus is seldom on polypharmacy as a whole or under-prescribing
**Drug Burden Index – DBI** *(Implicit tool, as requires further judgement to evaluate appropriateness after calculating score)*	- Widely researched risk score<br>- Calculates the cumulative exposure of sedatives and anticholinergics to give a score between 0 and 1.	- International studies have shown predictive validity for falls, fractures, GP visits and admission.<br>- Takes into account licenced doses to allow transferability between counties<br>- Easier to automate in drug records.	- No positive clinical outcomes in RCTs to date<br>- No consideration for appropriateness or specific indication of medicines<br>- Only focussed on sedatives, and anticholinergics<br>- Can be challenging to calculate at point of care unless computerised

For peer review only

			- Does not address polypharmacy as a whole or under-prescribing

A descriptive summary of selected examples of widely studied explicit and implicit tools.[48,54,57-60]

Several systematic reviews have revealed a high level of variability of included criteria within explicit tools.[47-50,54,61] A review of 36 explicit tools reported criteria spanning 907 medications and medication classes, but only 44 medications and 4 classes were reported by the majority.[48] This was despite over 85% of these tools being developed based on either the Beers or the STOPP/START (Screening Tool of Older Person's Prescriptions/Screening Tool to Alert doctors to Right Treatment) criteria).[48] Due to this, many studies combine several explicit criteria to complement the list of medications included.[47,48,50,62-66] Only about a third of tools suggested alternative treatments to potentially inappropriate medicines, yet nearly 70% of suggested alternatives were deemed inappropriate by other tools.[47] Implicit tools are also diverse in nature, with reviews identifying over 16 different tools incorporating implicit criteria.[54,67] These ranged from risk scores to lists of questions specifying appropriate use or criteria to evaluate the administrative burden to patients.[54,67-69] Several tools combine implicit and explicit indicators, including documents used for national guidance (e.g. Australian Prescribing Indicators Tool).[67,70,71]

<u>Key limitations in identifying problematic polypharmacy in practice</u>

Current strategies to identify problematic polypharmacy demonstrate inadequate performance. At present, risk stratification tools remain too broad, and seldom consider the clinical appropriateness of individual medications.[34,72] Though comprehensive explicit criteria are helpful in identifying potentially inappropriate medications, translation into everyday care remains elusive due to challenges in clinical application, and the omission of medications not

For peer review only

For peer review only - http://bmjopen.bmj.com/site/about/guidelines.xhtml

included in criteria.[48,73] For instance, previous studies have found that less than 25% of adverse drug reactions are caused by drugs listed by Beers criteria.[74,75] Additionally, apart from STOPP/START, most widely used tools were not designed to also cover under-prescribing (Table 4), with some studies also choosing to omit many of the under-prescribing criteria in its application.[47-50,54] Furthermore, there have been questions as to the utility of long lists of medications as studies have shown a high prevalence of potentially inappropriate medications (over 30% of patients) but low variability within many criteria, potentially leaving little room for improvement.[76] Studies also mention usability issues with such long lists, even with computerised integration, and the difficulties of making treatment decisions without prioritisation of criteria, particularly as their predictive validity is unknown.[47,63,72,77,78] Still, as the majority of instruments were developed focussing on patients over 65 years old, the suitability for middle-aged adults is unknown, yet both polypharmacy and multimorbidity are increasing in this age-group.[20,30,79] Only a handful of criteria have been developed and validated (e.g. Prescribing Optimally in Middle-aged People's Treatments – PROMPT criteria), all including significantly fewer criteria for individual medications and medication classes.[54,56,67,80,81] Again, this further limits applicability in problematic polypharmacy, where the whole of the medication regimen should be considered.

Addressing problematic polypharmacy through interventions

Large variability in interventions addressing polypharmacy

Interventions to address problematic polypharmacy have covered a wide range of aims, such as reducing adverse drug reactions, increasing the appropriateness of medicines use, reducing falls, improving patient adherence, and maintaining quality of life.[13,82-85] To combat overprescribing specifically, deprescribing interventions have also received significant attention, though interventions that focus on under-prescribing are much less.[86-90] Several

For peer review only - http://bmjopen.bmj.com/site/about/guidelines.xhtml

large reviews highlight good evidence of improving prescribing patterns, yet mixed and low certainty of evidence in improving patient-relevant outcome measures.[2,13-15,84,91-94] Reviews covering over 150 primary studies reported no differences in all-cause mortality and no clear evidence of benefit in reduced hospitalisations, when comparing interventions to usual care.[13,84,92,95-98] There were also no differences in quality of life, adverse drug reactions, readmission rates, primary care visits and emergency department visits.[13,84,96-98] Two reviews have highlighted some economic benefits in reducing healthcare expenditure, but others highlight inconsistencies due to low quality evidence.[96,97,99] Overall, there is evidence that these interventions are safe and do not lead to harm, but may be time and resource intensive for both patients and practitioners, as many require continuing input.[13,84,86] Likewise, mixed evidence of improved clinical outcomes, such as falls, is also observed even in more focussed populations, such as those with frailty and in long-term care facilities.[88-90,100]

Multiple intervention components to address polypharmacy, with unclear effectiveness

The majority of polypharmacy interventions were multimodal with a review revealing fourteen different elements from 80 studies and an average of 2.5 elements per intervention.[13,101] The most common elements included medication reviews and training for professionals and utilising tools, such as clinical decision support, checklists or audit and feedback.[13,43,78,101-104] Other components strengthened inter-professional or multidisciplinary collaborations by involving clinical pharmacists, nurses or geriatricians.[13,96,98,101,104-111] There were also patient facing components, such as education and training for patients and patient interviews to seek their understanding and lived experiences with their medicines.[112-117] Despite the growing literature on the importance of patient-centred care in medicines management, current literature highlights that patient

For peer review only - http://bmjopen.bmj.com/site/about/guidelines.xhtml

priorities are seldom fully integrated into polypharmacy interventions.[13,86,95,101,118-125] Patient-centred approaches also appear to be key to improving adherence, as a frequent discordance between practitioner and patient views is reported.[13,15,101,126-132] More recent interventions that do adopt a patient centred model, show some mixed improvements in appropriate prescribing and limited improvements in outcomes, reflecting some of the challenges of integrating patient priorities into routine medication reviews.[103,112-117,133,134]

In terms of effectiveness of individual intervention elements, similar effect sizes have been observed in reducing the number of potentially inappropriate medicines, with no particular components showing particular superiority.[13,84,101] However, generalised professional education programmes appear to be less effective than individualised interventions.[13,135] Medication reviews are also the most commonly adopted component, but as a single intervention, there remains insufficient evidence of medication reviews alone improving clinical outcome.[88,136,137] Despite the advantages of automation, electronic tools in trials demonstrate high variability in implementation within large pan-European and global trials, and no clear positive advantages on relevant patient outcomes have been reported.[13,138,139] Pharmacists show promise as an extra resource for managing polypharmacy in individual studies, but two recent reviews revealed uncertain effects on optimising medicines.[96,98,106-110,140,141] Community pharmacists can contribute to medication safety, but more in-depth management such as polypharmacy medication reviews were seen as outside the scope of community pharmacy.[109,142,143]

For peer review only

Key challenges in addressing problematic polypharmacy

In spite of the breadth of interventions targeting polypharmacy, it remains unclear which intervention components are more important.[13] Theory informed interventions are few and there are opportunities for improvements in intervention design through stronger foundations on theoretical frameworks and behaviour change techniques.[132,144-148] Widespread variation exists in the everyday management of medicines and polypharmacy.[2,3,149-151] These variations occur at patient, prescriber, regional and international levels, and indicate links between problematic polypharmacy and health inequalities.[1-3,39,149,150,152-154] As such, multiple challenges to addressing problematic polypharmacy need to be overcome, going beyond the identification of individual barriers and facilitators and translating these into practice within the complexity of interlinked systems of care.[2,39,155,156] The failure of the implementation of interventions is commonly down to the lack of consideration of integration into an already high demand system in everyday primary care.[156-159]

For patients with polypharmacy and multimorbidity, prioritisation and decision making is a challenge, given that they can receive ten times the amount of information during consultations due to compounding health issues, interacting medications and complex social issues.[160] Yet patient priorities and shared decision making is vital to deciding the appropriateness of medications, so improvements need to be made to both the clarity of information provided and the integration of patient views into polypharmacy decisions.[2,26,27,118,122,125,132,134,160,161] The majority of patients appear willing to discuss deprescribing medications, particularly if they have a good relationship with their doctor.[86,109,122,139,159] However, they also have strong beliefs and attitudes of the value of their medicines, with inertia generated when feeling well on their current medication regimen. [86,122,124,156,162-164]

For peer review only

For health professionals, work and effort are required to even consider deprescribing, particularly as prescribing is so embedded in routine practice and finding an appropriate time to initiate the discussion is often difficult given competing priorities.[157,158,165-167] A comprehensive polypharmacy medication review is described as 'impossible' to complete in 10 minutes, leading to practitioners defaulting to a swifter review and degrading medication reviews to being 'mundane' tasks.[162] This is combined with the work to gain awareness (of new policies, guidelines and tools), overcome significant uncertainty in evidence (with 'unmeasurable' risk-benefit), and increase self-efficacy with limited resources and alternatives.[153,158,166-171] On an organisational and systems level, fragmentation of care and poor co-ordination between healthcare teams and specialists often lead to deferring ownership of deprescribing attempts, and miscommunication to patients, leading to medication related problems.[153,155,165,170,172,173] More comprehensive approaches and better resources are needed to support practitioners and organisations in pushing for improved polypharmacy decisions in a patient centred manner, rather than simply maintaining the 'status quo'.[35,86,152,166,168]

DISCUSSION

The evidence highlights that there are significant challenges to optimising the targeting of patients with problematic polypharmacy for intervention. Despite the extensive number of studies, there is little evidence of improved patient outcomes even for higher risk populations, including individuals with frailty and those in long-term care facilities. This is highly suggestive that the targeting of patients with problematic polypharmacy needs to be more

For peer review only - http://bmjopen.bmj.com/site/about/guidelines.xhtml

focussed or even that the incorrect populations and medications are currently being targeted. Simple counts or 'at-risk' populations appear too broad as case-finding approaches. Though potentially inappropriate prescribing criteria can be helpful, this approach is also inadequate as it omits many patients not fitting criteria, lacks consistency across criteria, and often overlooks underprescribing and multimorbidity. Furthermore, given the complexity of prescribing decisions in multimorbidity, and the importance of considering patient values, potentially inappropriate criteria can rarely be used alone in assessing appropriateness. Due to the frequent confounding of multimorbidity observed in studies evaluating polypharmacy outcomes, coupled with the diverse combinations of medications involved adverse drug reactions, there is a need for cross-cutting tools that can effectively capture the interplay of multiple health conditions in patients.[95,151] Ultimately, the targeting of patients with problematic polypharmacy need to take into account patient perspectives, individual factors and clinical appropriateness.

Implications for further research and practice

The approach to targeting patients needs to be improved as a first step, which may allow the identification of an optimal population for polypharmacy interventions. A next step to enhance clinical utility may be the routine adjustment of multimorbidity, as there is frequent confounding of polypharmacy outcomes within studies.[95] In doing so, we may be able to identify patients who are both over-prescribed and under-prescribed medicines yet consider some degree of clinical appropriateness. An opportunity exists to produce a cross-cutting measure beyond single diseases and individual drug interactions to evaluate patients as a whole, with the aim of improving overall health.[72,168]

For peer review only - http://bmjopen.bmj.com/site/about/guidelines.xhtml

The multifactorial drivers of polypharmacy also mean that approaches to address problematic polypharmacy need to go beyond targeting patients and practitioners alone.[39,156] Despite this, evidence of a systems approach encompassing policy makers, organisations, practitioners, patients and carers is lacking. [2,39,155,156] Both the growth of evidence-based medicine and desire to minimise all risk are significant drivers of increased medicines burden and problematic polypharmacy. Yet polypharmacy is rarely 'evidence-based', as it would be impossible to have a large enough sample size to perform drug trials and meta-analyses of the millions of combinations that patients with multimorbidity are taking.[6,174] Studies examining exclusion criteria of RCTs estimates that over 90% of this population would be excluded from trials, questioning their representativeness.[175] The emphasis on following guidelines and increasing treatment intensity should be balanced with the understanding that high quality personalised healthcare can only be achieved through also carefully reducing, stopping, or not initiating medication, with shared decision-making and agreed care objectives.[176,177]

Strengths and Limitations

This scoping review syntheses a wide breath of literature to explore the existing evidence. It allowed a systematic approach on an initial search strategy and was also adaptable to heterogeneous sources (e.g. policy documents) and developing literature (e.g. pilot studies) through related article, supplementary and grey literature searching. It examined the overlapping concepts of polypharmacy and multimorbidity concurrently, allowing synergies in evidence generation and critique.

There are several limitations of our review to consider. As with other scoping reviews, critical appraisal was not performed. Polypharmacy is an area that has received widespread attention,

For peer review only - http://bmjopen.bmj.com/site/about/guidelines.xhtml

with hundreds of primary studies and dozens of systematic reviews. Hence, in our attempts to present generalisable findings, the nuances within primary studies may be lost, such as differences in study setting, population, or intervention characteristics. While we made efforts to specifically extract primary care-related findings, it was not always possible to separate results in studies encompassing both primary and secondary care. Furthermore, by emphasising multimorbidity and primary care in our search, we may have overlooked research investigating more acute medication adjustments in polypharmacy patients.

CONCLUSION

An optimal approach for targeting patients with problematic polypharmacy is yet to be determined. To address the challenges posed by confounding, it may be valuable to develop a cross-cutting measure of polypharmacy that consistently accounts for multimorbidity. The complexities of prescribing decisions in polypharmacy highlight the importance of improved approaches that consider patient perspectives, individual factors, and clinical appropriateness.

Author's contributions

JT, MS, TB, RP and DMA were all involved in the conceptualisation and design of the study. JT performed the search, selection, extraction and synthesis with contributions from TB. JT wrote the first draft of the manuscript. All authors contributed to the content and revisions of the review and approval of the final manuscript.

For peer review only

Competing interests

DMA. reports research funding from Abbvie, Almirall, Celgene, Eli Lilly, Novartis, UCB, and the Leo Foundation outside the submitted work. The other authors have no conflicts of interest to disclose.

Funding statement

JT is funded by the NIHR Doctoral Fellowship Programme (Ref: NIHR302624) for this research project. TB and DMA are supported by the NIHR Greater Manchester Patient Safety Research Collaboration. The views expressed in this document are those of the authors and not necessarily those of the NIHR, NHS or the UK Department of Health and Social Care.

Patient and public involvement

Four members of patients and the public are involved in a steering group and provide invaluable contributions to the design, conduct, reporting and dissemination plans of our research.

Data sharing statement

All data relevant to the study are included in the article or uploaded as supplementary information.

REFERENCES

1. World Health Organization. Medication without harm: World Health Organization, 2017.
2. The Department of Health and Social Care. National overprescribing review report: Good for you, good for us, good for everybody, 2021.
3. Duerden M, Avery T, Payne R. Polypharmacy and medicines optimisation. The King's Fund. London, 2013.

For peer review only - http://bmjopen.bmj.com/site/about/guidelines.xhtml

4. Hajjar ER, Cafiero AC, Hanlon JT. Polypharmacy in elderly patients. *The American Journal of Geriatric Pharmacotherapy* 2007;5(4):345-51. doi: 10.1016/j.amjopharm.2007.12.002

5. National Institute for Health and Care Excellence (NICE). Acute Coronary Syndromes [NG185], 2020.

6. Buffel Du Vaure C, Dechartres A, Battin C, et al. Exclusion of patients with concomitant chronic conditions in ongoing randomised controlled trials targeting 10 common chronic conditions and registered at ClinicalTrials.gov: a systematic review of registration details. *BMJ Open* 2016;6(9):e012265. doi: 10.1136/bmjopen-2016-012265

7. Osanlou R, Walker L, Hughes DA, et al. Adverse drug reactions, multimorbidity and polypharmacy: a prospective analysis of 1 month of medical admissions. *BMJ Open* 2022;12(7):e055551. doi: 10.1136/bmjopen-2021-055551

8. Gellad WF, Grenard JL, Marcum ZA. A Systematic Review of Barriers to Medication Adherence in the Elderly: Looking Beyond Cost and Regimen Complexity. *The American Journal of Geriatric Pharmacotherapy* 2011;9(1):11-23. doi: 10.1016/j.amjopharm.2011.02.004

9. Nordin Olsson I, Runnamo R, Engfeldt P. Medication quality and quality of life in the elderly, a cohort study. *Health and Quality of Life Outcomes* 2011;9(1):95. doi: 10.1186/1477-7525-9-95

10. Davies EA, O'Mahony MS. Adverse drug reactions in special populations - the elderly: ADRs in the elderly. *British Journal of Clinical Pharmacology* 2015;80(4):796-807. doi: 10.1111/bcp.12596

11. Corsonello A, Pedone C, Incalzi R. Age-Related Pharmacokinetic and Pharmacodynamic Changes and Related Risk of Adverse Drug Reactions. *Current Medicinal Chemistry* 2010;17(6):571-84. doi: 10.2174/092986710790416326

12. Doherty AS, Boland F, Moriarty F, et al. Adverse drug reactions and associated patient characteristics in older community-dwelling adults: a 6-year prospective cohort study. *Br J Gen Pract* 2023;73(728):e211-e19. doi: 10.3399/bjgp.2022.0181 [published Online First: 20230223]

13. Cole JA, Gonçalves-Bradley DC, Alqahtani M, et al. Interventions to improve the appropriate use of polypharmacy for older people. *Cochrane Database of Systematic Reviews* 2023(10) doi: 10.1002/14651858.CD008165.pub5

14. Alldred DP, Kennedy M-C, Hughes C, et al. Interventions to optimise prescribing for older people in care homes. *Cochrane Database of Systematic Reviews* 2016;2016(2) doi: 10.1002/14651858.CD009095.pub3

15. Cross AJ, Elliott RA, Petrie K, et al. Interventions for improving medication-taking ability and adherence in older adults prescribed multiple medications. *Cochrane Database of Systematic Reviews* 2020(5) doi: 10.1002/14651858.CD012419.pub2

16. Tricco AC, Lillie E, Zarin W, et al. PRISMA Extension for Scoping Reviews (PRISMA-ScR): Checklist and Explanation. *Annals of Internal Medicine* 2018;169(7):467-73. doi: 10.7326/M18-0850

17. Taghy N, Cambon L, Cohen J-M, et al. Failure to Reach a Consensus in Polypharmacy Definition: An Obstacle to Measuring Risks and Impacts—Results of a Literature Review. *Therapeutics and Clinical Risk Management* 2020;Volume 16:57-73. doi: 10.2147/TCRM.S214187

18. Masnoon N, Shakib S, Kalisch-Ellett L, et al. What is polypharmacy? A systematic review of definitions. *BMC Geriatrics* 2017;17(1):230. doi: 10.1186/s12877-017-0621-2

For peer review only - http://bmjopen.bmj.com/site/about/guidelines.xhtml

19. Sirois C, Domingues NS, Laroche M-L, et al. Polypharmacy Definitions for Multimorbid Older Adults Need Stronger Foundations to Guide Research, Clinical Practice and Public Health. *Pharmacy* 2019;7(3):126. doi: 10.3390/pharmacy7030126
20. Vos R, Boesten J, Van Den Akker M. Fifteen-year trajectories of multimorbidity and polypharmacy in Dutch primary care—A longitudinal analysis of age and sex patterns. *PLOS ONE* 2022;17(2):e0264343. doi: 10.1371/journal.pone.0264343
21. Aubert CE, Streit S, Da Costa BR, et al. Polypharmacy and specific comorbidities in university primary care settings. *European Journal of Internal Medicine* 2016;35:35-42. doi: 10.1016/j.ejim.2016.05.022
22. Gutiérrez-Valencia M, Aldaz Herce P, Lacalle-Fabo E, et al. Prevalence of polypharmacy and associated factors in older adults in Spain: Data from the National Health Survey 2017. *Medicina Clínica* 2019;153(4):141-50. doi: 10.1016/j.medcli.2018.12.013
23. Rieckert A, Trampisch US, Klaaßen-Mielke R, et al. Polypharmacy in older patients with chronic diseases: a cross-sectional analysis of factors associated with excessive polypharmacy. *BMC Family Practice* 2018;19(1):113. doi: 10.1186/s12875-018-0795-5
24. Payne RA, Avery AJ, Duerden M, et al. Prevalence of polypharmacy in a Scottish primary care population. *European Journal of Clinical Pharmacology* 2014;70(5):575-81. doi: 10.1007/s00228-013-1639-9
25. Monégat M, Sermet C, Perronnin M, et al. Polypharmacy: definitions, measurement and stakes involved. *Review of the literature and measurement tests* 2014;8
26. Mair A, Wilson M, Dreischulte T. Addressing the Challenge of Polypharmacy. *Annual Review of Pharmacology and Toxicology* 2020;60(1):661-81. doi: 10.1146/annurev-pharmtox-010919-023508
27. Heaton J, Britten N, Krska J, et al. Person-centred medicines optimisation policy in England: an agenda for research on polypharmacy. *Primary Health Care Research & Development* 2017;18(01):24-34. doi: 10.1017/S1463423616000207
28. Sirois C, Lunghi C, Laroche M-L, et al. The delicate choice of optimal basic therapy for multimorbid older adults: A cross-sectional survey. *Research in Social and Administrative Pharmacy* 2019;15(6):761-66. doi: 10.1016/j.sapharm.2018.09.008
29. National Institute for Health and Care Excellence (NICE). Multimorbidity and polypharmacy [KTT18], 2017.
30. Guthrie B, Makubate B, Hernandez-Santiago V, et al. The rising tide of polypharmacy and drug-drug interactions: population database analysis 1995–2010. *BMC Medicine* 2015;13(1):74. doi: 10.1186/s12916-015-0322-7
31. McCarthy C, Flood M, Clyne B, et al. Medication changes and potentially inappropriate prescribing in older patients with significant polypharmacy. *International Journal of Clinical Pharmacy* 2023;45(1):191-200. doi: 10.1007/s11096-022-01497-2
32. Von Buedingen F, Hammer MS, Meid AD, et al. Changes in prescribed medicines in older patients with multimorbidity and polypharmacy in general practice. *BMC Family Practice* 2018;19(1):131. doi: 10.1186/s12875-018-0825-3
33. Calderón-Larrañaga A, Gimeno-Feliu LA, González-Rubio F, et al. Polypharmacy Patterns: Unravelling Systematic Associations between Prescribed Medications. *PLoS ONE* 2013;8(12):e84967. doi: 10.1371/journal.pone.0084967
34. Kadam UT, Roberts I, White S, et al. Conceptualizing multiple drug use in patients with comorbidity and multimorbidity: proposal for standard definitions beyond the term polypharmacy. *Journal of Clinical Epidemiology* 2019;106:98-107. doi: 10.1016/j.jclinepi.2018.10.014
35. Clyne B, Cooper JA, Hughes CM, et al. ‘Potentially inappropriate or specifically appropriate?’ Qualitative evaluation of general practitioners views on prescribing,

For peer review only - http://bmjopen.bmj.com/site/about/guidelines.xhtml

polypharmacy and potentially inappropriate prescribing in older people. *BMC Family Practice* 2016;17(1):109. doi: 10.1186/s12875-016-0507-y
36. Australian Commission on Safety and Quality in Health Care. Polypharmacy, 75 years and over, 2020.
37. Scottish Government Polypharmacy Model of Care Group. Polypharmacy guidance, realistic prescribing. 3rd Edn: Scottish Government: Edinburgh, 2018.
38. All Wales Medicines Strategy Group. Polypharmacy in older people: A guide for healthcare professionals, 2023.
39. McIntosh J, Alonso A, MacLure K, et al. A case study of polypharmacy management in nine European countries: Implications for change management and implementation. *PLOS ONE* 2018;13(4):e0195232. doi: 10.1371/journal.pone.0195232
40. Krüger C, Schäfer I, Van Den Bussche H, et al. Non-random relations in drug use expressed as patterns comprising prescription and over-the-counter drugs in multimorbid elderly patients in primary care: Data of the exploratory analysis of the multicentre, observational cohort study MultiCare. *European Journal of General Practice* 2021;27(1):119-29. doi: 10.1080/13814788.2021.1933425
41. Villén N, Guisado-Clavero M, Fernández-Bertolín S, et al. Multimorbidity patterns, polypharmacy and their association with liver and kidney abnormalities in people over 65 years of age: a longitudinal study. *BMC Geriatrics* 2020;20(1):206. doi: 10.1186/s12877-020-01580-1
42. Monterde D, Vela E, Clèries M, et al. Multimorbidity as a predictor of health service utilization in primary care: a registry-based study of the Catalan population. *BMC Family Practice* 2020;21(1):39. doi: 10.1186/s12875-020-01104-1
43. Jäger C, Freund T, Steinhäuser J, et al. Impact of a tailored program on the implementation of evidence-based recommendations for multimorbid patients with polypharmacy in primary care practices—results of a cluster-randomized controlled trial. *Implementation Science* 2017;12(1):8. doi: 10.1186/s13012-016-0535-y
44. Novella A, Elli C, Tettamanti M, et al. Relation between drug therapy-based comorbidity indices, Charlson's comorbidity index, polypharmacy and mortality in three samples of older adults. *Archives of Gerontology and Geriatrics* 2022;100:104649. doi: 10.1016/j.archger.2022.104649
45. Häppölä P, Havulinna AS, Tasa T, et al. A data-driven medication score predicts 10-year mortality among aging adults. *Scientific Reports* 2020;10(1):15760. doi: 10.1038/s41598-020-72045-z
46. Guisado-Clavero M, Violán C, López-Jimenez T, et al. Medication patterns in older adults with multimorbidity: a cluster analysis of primary care patients. *BMC Family Practice* 2019;20(1):82. doi: 10.1186/s12875-019-0969-9
47. Schiavo G, Forgerini M, Lucchetta RC, et al. A comprehensive look at explicit screening tools for potentially inappropriate medication: A systematic scoping review. *Australasian Journal on Ageing* 2022;41(3):357-82. doi: 10.1111/ajag.13046
48. Motter FR, Fritzen JS, Hilmer SN, et al. Potentially inappropriate medication in the elderly: a systematic review of validated explicit criteria. *European Journal of Clinical Pharmacology* 2018;74(6):679-700. doi: 10.1007/s00228-018-2446-0
49. Lucchetti G, Lucchetti ALG. Inappropriate prescribing in older persons: A systematic review of medications available in different criteria. *Archives of Gerontology and Geriatrics* 2017;68:55-61. doi: 10.1016/j.archger.2016.09.003
50. Lee G, Lim JF, Page AT, et al. Applicability of explicit potentially inappropriate medication lists to the Australian context: A systematic review. *Australasian Journal on Ageing* 2022;41(2):200-21. doi: 10.1111/ajag.13038

For peer review only

51. Vrdoljak D, Borovac JA. Medication in the elderly - considerations and therapy prescription guidelines. *Acta Medica Academica* 2015;44(2):159. doi: 10.5644/ama2006-124.142
52. Lopez-Rodriguez JA, Rogero-Blanco E, Aza-Pascual-Salcedo M, et al. Potentially inappropriate prescriptions according to explicit and implicit criteria in patients with multimorbidity and polypharmacy. MULTIPAP: A cross-sectional study. *PLOS ONE* 2020;15(8):e0237186. doi: 10.1371/journal.pone.0237186
53. Sánchez-Fidalgo S, Guzmán-Ramos MI, Galván-Banqueri M, et al. Prevalence of drug interactions in elderly patients with multimorbidity in primary care. *International Journal of Clinical Pharmacy* 2017;39(2):343-53. doi: 10.1007/s11096-017-0439-1
54. Masnoon N, Shakib S, Kalisch-Ellett L, et al. Tools for Assessment of the Appropriateness of Prescribing and Association with Patient-Related Outcomes: A Systematic Review. *Drugs & Aging* 2018;35(1):43-60. doi: 10.1007/s40266-018-0516-8
55. Tampaki M, Livada A, Fourka MN, et al. Inappropriate prescribing in geriatric rural primary care: impact on adverse outcomes and relevant risk factors in a prospective observational cohort study. *Aging Clinical and Experimental Research* 2023;35(9):1901-07. doi: 10.1007/s40520-023-02475-y
56. Cooper JA, Ryan C, Smith SM, et al. The development of the PROMPT (PRescribing Optimally in Middle-aged People's Treatments) criteria. *BMC Health Services Research* 2014;14(1):484. doi: 10.1186/s12913-014-0484-6
57. American Geriatrics Society Beers Criteria® Update Expert Panel. American Geriatrics Society 2023 updated AGS Beers Criteria® for potentially inappropriate medication use in older adults. *Journal of the American Geriatrics Society* 2023;71(7):2052-81. doi: 10.1111/jgs.18372
58. O'Mahony D, Cherubini A, Guiteras AR, et al. STOPP/START criteria for potentially inappropriate prescribing in older people: version 3. *Eur Geriatr Med* 2023;14(4):625-32. doi: 10.1007/s41999-023-00777-y [published Online First: 20230531]
59. Hanlon JT, Schmader KE. The medication appropriateness index: A clinimetric measure. *Psychotherapy and Psychosomatics* 2022;91(2):78-83.
60. Liu BM, Kouladjian O'Donnell L, Redston MR, et al. Association of the Drug Burden Index (DBI) exposure with outcomes: A systematic review. *Journal of the American Geriatrics Society* 2023
61. Lee CS, Tan NC, Goh KLS, et al. Factors associated with potentially inappropriate prescribing among older persons in primary care settings: Systematic review. *Proceedings of Singapore Healthcare* 2023;32 doi: 10.1177/20101058231181478
62. Troncoso-Mariño A, López-Jiménez T, Roso-Llorach A, et al. Medication-related problems in older people in Catalonia: A real-world data study. *Pharmacoepidemiology and Drug Safety* 2021;30(2):220-28. doi: 10.1002/pds.5149
63. Rogero-Blanco E, Del-Cura-González I, Aza-Pascual-Salcedo M, et al. Drug interactions detected by a computer-assisted prescription system in primary care patients in Spain: MULTIPAP study. *European Journal of General Practice* 2021;27(1):90-96. doi: 10.1080/13814788.2021.1917543
64. Rogero-Blanco E, Lopez-Rodriguez JA, Sanz-Cuesta T, et al. Use of an Electronic Clinical Decision Support System in Primary Care to Assess Inappropriate Polypharmacy in Young Seniors With Multimorbidity: Observational, Descriptive, Cross-Sectional Study. *JMIR Medical Informatics* 2020;8(3):e14130. doi: 10.2196/14130

For peer review only - http://bmjopen.bmj.com/site/about/guidelines.xhtml

65. D'Aiuto C, Lunghi C, Guenette L, et al. Health care system costs related to potentially inappropriate medication use involving opioids in older adults in Canada. *BMC health services research* 2023;23(1):1295. doi: 10.1186/s12913-023-10303-2
66. Manirajan P, Sivanandy P. Drug Utilisation Review among Geriatric Patients with Noncommunicable Diseases in a Primary Care Setting in Malaysia. *Healthcare (Basel, Switzerland)* 2023;11(12) doi: 10.3390/healthcare11121665
67. Kaufmann CP, Tremp R, Hersberger KE, et al. Inappropriate prescribing: a systematic overview of published assessment tools. *European Journal of Clinical Pharmacology* 2014;70(1):1-11. doi: 10.1007/s00228-013-1575-8
68. Burt J, Elmore N, Campbell SM, et al. Developing a measure of polypharmacy appropriateness in primary care: systematic review and expert consensus study. *BMC Medicine* 2018;16(1):91. doi: 10.1186/s12916-018-1078-7
69. George J, Phun Y-T, Bailey MJ, et al. Development and Validation of the Medication Regimen Complexity Index. *Annals of Pharmacotherapy* 2004;38(9):1369-76. doi: 10.1345/aph.1D479
70. Basger BJ, Chen TF, Moles RJ. Validation of prescribing appropriateness criteria for older Australians using the RAND/UCLA appropriateness method. *BMJ Open* 2012;2(5):e001431. doi: 10.1136/bmjopen-2012-001431
71. Scott I, Anderson K, Freeman C. Review of structured guides for deprescribing. *European Journal of Hospital Pharmacy* 2017;24(1):51-57. doi: 10.1136/ejhpharm-2015-000864
72. Cadogan CA, Ryan C, Hughes CM. Appropriate Polypharmacy and Medicine Safety: When Many is not Too Many. *Drug Safety* 2016;39(2):109-16. doi: 10.1007/s40264-015-0378-5
73. Akyon SH, Akyon FC, Yilmaz TE. Artificial intelligence-supported web application design and development for reducing polypharmacy side effects and supporting rational drug use in geriatric patients. *Frontiers in Medicine* 2023;10:1029198. doi: 10.3389/fmed.2023.1029198
74. Laroche M-L, Charmes J-P, Nouaille Y, et al. Is inappropriate medication use a major cause of adverse drug reactions in the elderly? *British Journal of Clinical Pharmacology* 2007;63(2):177-86. doi: 10.1111/j.1365-2125.2006.02831.x
75. Miller GC, Valenti L, Britt H, et al. Drugs causing adverse events in patients aged 45 or older: a randomised survey of Australian general practice patients. *BMJ Open* 2013;3(10):e003701. doi: 10.1136/bmjopen-2013-003701
76. Brown JD, Hutchison LC, Li C, et al. Predictive Validity of the Beers and Screening Tool of Older Persons' Potentially Inappropriate Prescriptions (STOPP) Criteria to Detect Adverse Drug Events, Hospitalizations, and Emergency Department Visits in the United States. *Journal of the American Geriatrics Society* 2016;64(1):22-30. doi: 10.1111/jgs.13884
77. Cahir C, Moriarty F, Teljeur C, et al. Potentially Inappropriate Prescribing and Vulnerability and Hospitalization in Older Community-Dwelling Patients. *Annals of Pharmacotherapy* 2014;48(12):1546-54. doi: 10.1177/1060028014552821
78. Del Cura-González I, López-Rodríguez JA, Leiva-Fernández F, et al. How to Improve Healthcare for Patients with Multimorbidity and Polypharmacy in Primary Care: A Pragmatic Cluster-Randomized Clinical Trial of the MULTIPAP Intervention. *Journal of Personalized Medicine* 2022;12(5):752. doi: 10.3390/jpm12050752
79. O'Regan A, Glynn L, Niranjamn V, et al. How often do patients attend general practice, how often are they referred to hospital, and how do multi-morbidity and polypharmacy affect general practice attendance and referral rates? *Rural Remote Health* 2023;23(1):8106. doi: 10.22605/rrh8106 [published Online First: 20230110]

For peer review only - http://bmjopen.bmj.com/site/about/guidelines.xhtml

80. Khatter A, Moriarty F, Ashworth M, et al. Prevalence and predictors of potentially inappropriate prescribing in middle-aged adults: a repeated cross-sectional study. *British Journal of General Practice* 2021;71(708):e491-e97. doi: 10.3399/BJGP.2020.1048

81. Cooper JA, Moriarty F, Ryan C, et al. Potentially inappropriate prescribing in two populations with differing socio-economic profiles: a cross-sectional database study using the PROMPT criteria. *European Journal of Clinical Pharmacology* 2016;72(5):583-91. doi: 10.1007/s00228-015-2003-z

82. Rankin A, Cadogan CA, Patterson SM, et al. Interventions to improve the appropriate use of polypharmacy for older people. *Cochrane Database of Systematic Reviews* 2018;2018(9) doi: 10.1002/14651858.CD008165.pub4

83. Cooper JA, Cadogan CA, Patterson SM, et al. Interventions to improve the appropriate use of polypharmacy in older people: a Cochrane systematic review. *BMJ Open* 2015;5(12):e009235. doi: 10.1136/bmjopen-2015-009235

84. Keller MS, Qureshi N, Mays AM, et al. Cumulative Update of a Systematic Overview Evaluating Interventions Addressing Polypharmacy. *JAMA Network Open* 2024;7(1):e2350963-e63. doi: 10.1001/jamanetworkopen.2023.50963

85. Johansson T, Abuzahra ME, Keller S, et al. Impact of strategies to reduce polypharmacy on clinically relevant endpoints: a systematic review and meta-analysis: Impact of strategies to reduce polypharmacy. *British Journal of Clinical Pharmacology* 2016;82(2):532-48. doi: 10.1111/bcp.12959

86. Reeve J, Maden M, Hill R, et al. Deprescribing medicines in older people living with multimorbidity and polypharmacy: the TAILOR evidence synthesis. *Health Technology Assessment* 2022;26(32):1-148. doi: 10.3310/AAFO2475

87. Reeve E, Gnjidic D, Long J, et al. A systematic review of the emerging definition of 'deprescribing' with network analysis: implications for future research and clinical practice.: The emerging definition of 'deprescribing'. *British Journal of Clinical Pharmacology* 2015;80(6):1254-68. doi: 10.1111/bcp.12732

88. Seppala LJ, Kamkar N, Van Poelgeest EP, et al. Medication reviews and deprescribing as a single intervention in falls prevention: a systematic review and meta-analysis. *Age and Ageing* 2022;51(9):afac191. doi: 10.1093/ageing/afac191

89. Kua C-H, Mak VSL, Huey Lee SW. Health Outcomes of Deprescribing Interventions Among Older Residents in Nursing Homes: A Systematic Review and Meta-analysis. *Journal of the American Medical Directors Association* 2019;20(3):362-72.e11. doi: 10.1016/j.jamda.2018.10.026

90. Shrestha S, Poudel A, Cardona M, et al. Impact of deprescribing dual-purpose medications on patient-related outcomes for older adults near end-of-life: a systematic review and meta-analysis. *Therapeutic Advances in Drug Safety* 2021;12:204209862110523. doi: 10.1177/20420986211052343

91. Soler O, Barreto JOM. Community-Level Pharmaceutical Interventions to Reduce the Risks of Polypharmacy in the Elderly: Overview of Systematic Reviews and Economic Evaluations. *Frontiers in Pharmacology* 2019;10:302. doi: 10.3389/fphar.2019.00302

92. Anderson LJ, Schnipper JL, Nuckols TK, et al. A systematic overview of systematic reviews evaluating interventions addressing polypharmacy. *American Journal of Health-System Pharmacy* 2019;76(21):1777-87. doi: 10.1093/ajhp/zxz196

93. Dills H, Shah K, Messinger-Rapport B, et al. Deprescribing Medications for Chronic Diseases Management in Primary Care Settings: A Systematic Review of Randomized Controlled Trials. *Journal of the American Medical Directors Association* 2018;19(11):923-35.e2. doi: 10.1016/j.jamda.2018.06.021

For peer review only

94. Ulley J, Harrop D, Ali A, et al. Deprescribing interventions and their impact on medication adherence in community-dwelling older adults with polypharmacy: a systematic review. *BMC Geriatrics* 2019;19(1):15. doi: 10.1186/s12877-019-1031-4
95. Ali MU, Sherifali D, Fitzpatrick-Lewis D, et al. Interventions to address polypharmacy in older adults living with multimorbidity: Review of reviews. *Canadian Family Physician* 2022;68(7):e215-e26. doi: 10.46747/cfp.6807e215
96. Riordan DO, Walsh KA, Galvin R, et al. The effect of pharmacist-led interventions in optimising prescribing in older adults in primary care: A systematic review. *SAGE Open Medicine* 2016;4:205031211665256. doi: 10.1177/2050312116652568
97. Hill-Taylor B, Walsh KA, Stewart S, et al. Effectiveness of the STOPP/START (Screening Tool of Older Persons' potentially inappropriate Prescriptions/Screening Tool to Alert doctors to the Right Treatment) criteria: systematic review and meta-analysis of randomized controlled studies. *Journal of Clinical Pharmacy and Therapeutics* 2016;41(2):158-69. doi: 10.1111/jcpt.12372
98. Tasai S, Kumpat N, Dilokthornsakul P, et al. Impact of Medication Reviews Delivered by Community Pharmacist to Elderly Patients on Polypharmacy: A Meta-analysis of Randomized Controlled Trials. *Journal of Patient Safety* 2021;17(4):290-98. doi: 10.1097/PTS.0000000000000599
99. Laberge M, Sirois C, Lunghi C, et al. Economic Evaluations of Interventions to Optimize Medication Use in Older Adults with Polypharmacy and Multimorbidity: A Systematic Review. *Clinical Interventions in Aging* 2021;Volume 16:767-79. doi: 10.2147/CIA.S304074
100. Ibrahim K, Cox NJ, Stevenson JM, et al. A systematic review of the evidence for deprescribing interventions among older people living with frailty. *BMC Geriatrics* 2021;21(1):258. doi: 10.1186/s12877-021-02208-8
101. Lee JQ, Ying K, Lun P, et al. Intervention elements to reduce inappropriate prescribing for older adults with multimorbidity receiving outpatient care: a scoping review. *BMJ Open* 2020;10(8):e039543. doi: 10.1136/bmjopen-2020-039543
102. Michiels-Corsten M, Gerlach N, Schleef T, et al. Generic instruments for drug discontinuation in primary care: A systematic review. *British Journal of Clinical Pharmacology* 2020;86(7):1251-66. doi: 10.1111/bcp.14287
103. Zechmann S, Senn O, Valeri F, et al. Effect of a patient-centred deprescribing procedure in older multimorbid patients in Swiss primary care - A cluster-randomised clinical trial. *BMC Geriatrics* 2020;20(1):471. doi: 10.1186/s12877-020-01870-8
104. Jäger C, Steinhäuser J, Freund T, et al. A tailored programme to implement recommendations for multimorbid patients with polypharmacy in primary care practices—process evaluation of a cluster randomized trial. *Implementation Science* 2017;12(1):31. doi: 10.1186/s13012-017-0559-y
105. San-José A, Pérez-Bocanegra C, Agustí A, et al. Integrated health intervention on polypharmacy and inappropriate prescribing in elderly people with multimorbidity: Results at the end of the intervention and at 6 months after the intervention. *Medicina Clínica* 2021;156(6):263-69. doi: 10.1016/j.medcli.2020.04.030
106. Cardwell K, Smith SM, Clyne B, et al. Evaluation of the General Practice Pharmacist (GPP) intervention to optimise prescribing in Irish primary care: a non-randomised pilot study. *BMJ Open* 2020;10(6):e035087. doi: 10.1136/bmjopen-2019-035087
107. Alaa Eddine N, Schreiber J, El-Yazbi AF, et al. A pharmacist-led medication review service with a deprescribing focus guided by implementation science. *Frontiers in Pharmacology* 2023;14:1097238. doi: 10.3389/fphar.2023.1097238

For peer review only

For peer review only - http://bmjopen.bmj.com/site/about/guidelines.xhtml

108. Benson M, Murphy D, Hall L, et al. Medication management for complex patients in primary care: application of a remote, asynchronous clinical pharmacist model. *Postgraduate Medicine* 2021;133(7):784-90. doi: 10.1080/00325481.2021.1934492
109. Uhl MC, Muth C, Gerlach FM, et al. Patient-perceived barriers and facilitators to the implementation of a medication review in primary care: a qualitative thematic analysis. *BMC Family Practice* 2018;19(1):3. doi: 10.1186/s12875-017-0707-0
110. Bell HT, Granas AG, Enmarker I, et al. Nurses' and pharmacists' learning experiences from participating in interprofessional medication reviews for elderly in primary health care - a qualitative study. *BMC Family Practice* 2017;18(1):30. doi: 10.1186/s12875-017-0598-0
111. Köberlein-Neu J, Mennemann H, Hamacher S, et al. Interprofessional Medication Management in Patients With Multiple Morbidities. *Deutsches Ärzteblatt international* 2016 doi: 10.3238/arztebl.2016.0741
112. Molist-Brunet N, Sevilla-Sánchez D, Puigoriol-Juvanteny E, et al. Improving individualized prescription in patients with multimorbidity through medication review. *BMC Geriatrics* 2022;22(1):417. doi: 10.1186/s12877-022-03107-2
113. McCarthy C, Clyne B, Boland F, et al. GP-delivered medication review of polypharmacy, deprescribing, and patient priorities in older people with multimorbidity in Irish primary care (SPPiRE Study): A cluster randomised controlled trial. *PLOS Medicine* 2022;19(1):e1003862. doi: 10.1371/journal.pmed.1003862
114. McCarthy C, Pericin I, Smith SM, et al. Patient and general practitioner experiences of implementing a medication review intervention in older people with multimorbidity: Process evaluation of the SPPiRE trial. *Health Expectations* 2022;25(6):3225-37. doi: 10.1111/hex.13630
115. McCarthy C, Moriarty F, Wallace E, et al. The evolution of an evidence based intervention designed to improve prescribing and reduce polypharmacy in older people with multimorbidity and significant polypharmacy in primary care (SPPiRE). *Journal of Comorbidity* 2020;10:2235042X094624. doi: 10.1177/2235042X20946243
116. Schäfer I, Kaduszkiewicz H, Mellert C, et al. Narrative medicine-based intervention in primary care to reduce polypharmacy: results from the cluster-randomised controlled trial MultiCare AGENDA. *BMJ Open* 2018;8(1):e017653. doi: 10.1136/bmjopen-2017-017653
117. Mann N-K, Schmiedl S, Mortsiefer A, et al. Development of a deprescribing manual for frail older people for use in the COFRAIL study and in primary care. *Therapeutic Advances in Drug Safety* 2022;13:204209862211226. doi: 10.1177/20420986221122684
118. Mangin D, Risdon C, Lamarche L, et al. 'I think this medicine actually killed my wife': patient and family perspectives on shared decision-making to optimize medications and safety. *Therapeutic Advances in Drug Safety* 2019;10:204209861983879. doi: 10.1177/2042098619838796
119. Schöpf AC, Von Hirschhausen M, Farin E, et al. Elderly patients' and GPs' perspectives of patient–GP communication concerning polypharmacy: a qualitative interview study. *Primary Health Care Research & Development* 2018;19(04):355-64. doi: 10.1017/S1463423617000883
120. Knowles S, Hays R, Senra H, et al. Empowering people to help speak up about safety in primary care: Using codesign to involve patients and professionals in developing new interventions for patients with multimorbidity. *Health Expectations* 2018;21(2):539-48. doi: 10.1111/hex.12648

For peer review only

121. Noel PH, Chris Frueh B, Larme AC, et al. Collaborative care needs and preferences of primary care patients with multimorbidity. *Health Expectations* 2005;8(1):54-63. doi: 10.1111/j.1369-7625.2004.00312.x

122. Maidment I, Lawson S, Wong G, et al. Towards an understanding of the burdens of medication management affecting older people: the MEMORABLE realist synthesis. *BMC Geriatrics* 2020;20(1):183. doi: 10.1186/s12877-020-01568-x

123. Reeve E, Low L-F, Hilmer SN. Beliefs and attitudes of older adults and carers about deprescribing of medications: a qualitative focus group study. *British Journal of General Practice* 2016;66(649):e552-e60. doi: 10.3399/bjgp16X685669

124. Zechmann S, Trueb C, Valeri F, et al. Barriers and enablers for deprescribing among older, multimorbid patients with polypharmacy: an explorative study from Switzerland. *BMC Family Practice* 2019;20(1):64. doi: 10.1186/s12875-019-0953-4

125. Adamson J, Hanson H, Todd A, et al. Medication work among nonagenarians: a qualitative study of the Newcastle 85+ cohort participants at 97 years old. *British Journal of General Practice* 2023;73(729):E267-E75. doi: 10.3399/BJGP.2022.0188

126. Reeve E, Shakib S, Hendrix I, et al. Review of deprescribing processes and development of an evidence-based, patient-centred deprescribing process: Patient-centred deprescribing process. *British Journal of Clinical Pharmacology* 2014;78(4):738-47. doi: 10.1111/bcp.12386

127. Lozano-Hernández CM, López-Rodríguez JA, Leiva-Fernández F, et al. Social support, social context and nonadherence to treatment in young senior patients with multimorbidity and polypharmacy followed-up in primary care. MULTIPAP Study. *PLOS ONE* 2020;15(6):e0235148. doi: 10.1371/journal.pone.0235148

128. Ose D, Mahler C, Vogel, et al. Lets talk about medication: concordance in rating medication adherence among multimorbid patients and their general practitioners. *Patient Preference and Adherence* 2012:839. doi: 10.2147/PPA.S35498

129. Weir KR, Naganathan V, Carter SM, et al. The role of older patients' goals in GP decision-making about medicines: a qualitative study. *BMC Family Practice* 2021;22(1):13. doi: 10.1186/s12875-020-01347-y

130. Junius-Walker U, Viniol A, Michiels-Corsten M, et al. MediQuit, an Electronic Deprescribing Tool for Patients on Polypharmacy: Results of a Feasibility Study in German General Practice. *Drugs & Aging* 2021;38(8):725-33. doi: 10.1007/s40266-021-00861-7

131. Neuner-Jehle S, Zechmann S, Grundmann Maissen D, et al. Patient-provider concordance in the perception of illness and disease: a cross-sectional study among multimorbid patients and their general practitioners in Switzerland. *Patient Prefer Adherence* 2017;11:1451-58. doi: 10.2147/ppa.S137388 [published Online First: 20170822]

132. Ie K, Machino R, Albert SM, et al. Deprescribing as an Opportunity to Facilitate Patient-Centered Care: A Qualitative Study of General Practitioners and Pharmacists in Japan. *International Journal of Environmental Research and Public Health* 2023;20(4):3543. doi: 10.3390/ijerph20043543

133. Michiels-Corsten M, Gerlach N, Junius-Walker U, et al. MediQuit – an electronic deprescribing tool: a pilot study in German primary care; GPs' and patients' perspectives. *BMC Primary Care* 2022;23(1):252. doi: 10.1186/s12875-022-01852-2

134. Mangin D, Lamarche L, Agarwal G, et al. Team approach to polypharmacy evaluation and reduction: feasibility randomized trial of a structured clinical pathway to reduce polypharmacy. *Pilot and Feasibility Studies* 2023;9(1):84. doi: 10.1186/s40814-023-01315-0

For peer review only - http://bmjopen.bmj.com/site/about/guidelines.xhtml

135. Page AT, Clifford RM, Potter K, et al. The feasibility and effect of deprescribing in older adults on mortality and health: a systematic review and meta-analysis. *British Journal of Clinical Pharmacology* 2016;82(3):583-623. doi: 10.1111/bcp.12975
136. Verma A, Saha S, Jarl J, et al. An Overview of Systematic Reviews and Meta-Analyses on the Effect of Medication Interventions Targeting Polypharmacy for Frail Older Adults. *Journal of Clinical Medicine* 2023;12(4):1379. doi: 10.3390/jcm12041379
137. Anderson LJ, Schnipper JL, Nuckols TK, et al. Effect of medication reconciliation interventions on outcomes: A systematic overview of systematic reviews. *American Journal of Health-System Pharmacy* 2019;76(24):2028-40. doi: 10.1093/ajhp/zxz236
138. Rieckert A, Reeves D, Altiner A, et al. Use of an electronic decision support tool to reduce polypharmacy in elderly people with chronic diseases: cluster randomised controlled trial. *BMJ* 2020:m1822. doi: 10.1136/bmj.m1822
139. Muth C, Uhlmann L, Haefeli WE, et al. Effectiveness of a complex intervention on Prioritising Multimedication in Multimorbidity (PRIMUM) in primary care: results of a pragmatic cluster randomised controlled trial. *BMJ Open* 2018;8(2):e017740. doi: 10.1136/bmjopen-2017-017740
140. Hasan Ibrahim AS, Barry HE, Hughes CM. A systematic review of general practice-based pharmacists' services to optimize medicines management in older people with multimorbidity and polypharmacy. *Family Practice* 2021;38(4):509-23. doi: 10.1093/fampra/cmaa146
141. Croke A, Cardwell K, Clyne B, et al. The effectiveness and cost of integrating pharmacists within general practice to optimize prescribing and health outcomes in primary care patients with polypharmacy: a systematic review. *BMC Primary Care* 2023;24(1):41. doi: 10.1186/s12875-022-01952-z
142. Collier A, Balmer D, Dai L, et al. Older people, medication safety, and the role of the community pharmacist: a longitudinal ethnographic study. *Journal of Pharmacy Practice and Research* 2023;53(1):18-25. doi: 10.1002/jppr.1839
143. Fudge N, Swinglehurst D. 'It's all about patient safety': an ethnographic study of how pharmacy staff construct medicines safety in the context of polypharmacy. *BMJ Open* 2021;11(2):e042504. doi: 10.1136/bmjopen-2020-042504
144. Hansen CR, O'Mahony D, Kearney PM, et al. Identification of behaviour change techniques in deprescribing interventions: a systematic review and meta-analysis: Behaviour change techniques in deprescribing interventions. *British Journal of Clinical Pharmacology* 2018;84(12):2716-28. doi: 10.1111/bcp.13742
145. Gorman A, Rankin A, Hughes C, et al. Theoretically derived interventions aimed at improving appropriate polypharmacy in primary care: A systematic review. *Exploratory Research in Clinical and Social Pharmacy* 2022;7:100166. doi: 10.1016/j.rcsop.2022.100166
146. Rankin A, Gorman A, Cole J, et al. An external pilot cluster randomised controlled trial of a theory-based intervention to improve appropriate polypharmacy in older people in primary care (PolyPrime). *Pilot and Feasibility Studies* 2022;8(1):203. doi: 10.1186/s40814-022-01161-6
147. Kirwan C, Hynes L, Hart N, et al. The multimorbidity collaborative medication review and decision making (MyComrade) study: a pilot cluster randomised trial in two healthcare systems. *Pilot and Feasibility Studies* 2022;8(1):225. doi: 10.1186/s40814-022-01107-y
148. Cadogan CA, Ryan C, Francis JJ, et al. Improving appropriate polypharmacy for older people in primary care: selecting components of an evidence-based intervention to target prescribing and dispensing. *Implementation Science* 2015;10(1):161. doi: 10.1186/s13012-015-0349-3

For peer review only - http://bmjopen.bmj.com/site/about/guidelines.xhtml

149. Khezrian M, McNeil CJ, Murray AD, et al. An overview of prevalence, determinants and health outcomes of polypharmacy. *Therapeutic Advances in Drug Safety* 2020;11:204209862093374. doi: 10.1177/2042098620933741
150. Ong SM, Lim YMF, Sivasampu S, et al. Variation of polypharmacy in older primary care attenders occurs at prescriber level. *BMC Geriatrics* 2018;18(1):59. doi: 10.1186/s12877-018-0750-2
151. Fahmi A, Wong D, Walker L, et al. Combinations of medicines in patients with polypharmacy aged 65-100 in primary care: Large variability in risks of adverse drug related and emergency hospital admissions. *PLoS ONE* 2023;18(2 February):e0281466. doi: 10.1371/journal.pone.0281466
152. Kardas P, Mair A, Stewart D, et al. Optimizing polypharmacy management in the elderly: a comprehensive European benchmarking survey and the development of an innovative online benchmarking application. *Frontiers in Pharmacology* 2023;14:1254912. doi: 10.3389/fphar.2023.1254912
153. King E, Bazargan M, Entsuah N, et al. Potentially Inappropriate Medication Use among Underserved Older Latino Adults. *Journal of Clinical Medicine* 2023;12(9):3067. doi: 10.3390/jcm12093067
154. Barrio-Cortes J, Benito-Sanchez B, Villimar-Rodriguez AI, et al. Differences in healthcare service utilization in patients with polypharmacy according to their risk level by adjusted morbidity groups: a population-based cross-sectional study. *Journal of Pharmaceutical Policy and Practice* 2023;16(1):161. doi: 10.1186/s40545-023-00665-7
155. Sawan M, Reeve E, Turner J, et al. A systems approach to identifying the challenges of implementing deprescribing in older adults across different health-care settings and countries: a narrative review. *Expert Review of Clinical Pharmacology* 2020;13(3):233-45. doi: 10.1080/17512433.2020.1730812
156. Doherty AJ, Boland P, Reed J, et al. Barriers and facilitators to deprescribing in primary care: a systematic review. *BJGP Open* 2020;4(3):bjgpopen20X101096. doi: 10.3399/bjgpopen20X101096
157. Wallis KA, Andrews A, Henderson M. Swimming Against the Tide: Primary Care Physicians' Views on Deprescribing in Everyday Practice. *The Annals of Family Medicine* 2017;15(4):341-46. doi: 10.1370/afm.2094
158. Anderson K, Stowasser D, Freeman C, et al. Prescriber barriers and enablers to minimising potentially inappropriate medications in adults: a systematic review and thematic synthesis. *BMJ Open* 2014;4(12):e006544. doi: 10.1136/bmjopen-2014-006544
159. Lee JW, Jeong S, Han HR, et al. Barriers and facilitators to deprescribing before surgery: A qualitative study of providers and older adults. *Geriatric nursing (New York, NY)* 2023;53:135-40. doi: 10.1016/j.gerinurse.2023.07.018
160. Bujold M, Pluye P, Légaré F, et al. Decision-making and related outcomes of patients with complex care needs in primary care settings: a systematic literature review with a case-based qualitative synthesis. *BMC Primary Care* 2022;23(1):279. doi: 10.1186/s12875-022-01879-5
161. Engels LWS, van Merode T, Heijmans M, et al. Measurement of treatment burden in patients with multimorbidity in the Netherlands: translation and validation of the Multimorbidity Treatment Burden Questionnaire (NL-MTBQ). *Family practice* 2023 doi: 10.1093/fampra/cmad100
162. Swinglehurst D, Hogger L, Fudge N. Negotiating the polypharmacy paradox: a video-reflexive ethnography study of polypharmacy and its practices in primary care. *BMJ Quality & Safety* 2023;32(3):150-59. doi: 10.1136/bmjqs-2022-014963

For peer review only - http://bmjopen.bmj.com/site/about/guidelines.xhtml

163. Swinglehurst D, Fudge N. Organising polypharmacy: unpacking medicines, unpacking meanings—an ethnographic study. *BMJ Open* 2021;11(8):e049218. doi: 10.1136/bmjopen-2021-049218

164. Reeve E, To J, Hendrix I, et al. Patient Barriers to and Enablers of Deprescribing: a Systematic Review. *Drugs & Aging* 2013;30(10):793-807. doi: 10.1007/s40266-013-0106-8

165. Cairo Notari S, Sader J, Caire Fon N, et al. Understanding GPs' clinical reasoning processes involved in managing patients suffering from multimorbidity: A systematic review of qualitative and quantitative research. *International Journal of Clinical Practice* 2021;75(9) doi: 10.1111/ijcp.14187

166. Sinnott C, Hugh SM, Boyce MB, et al. What to give the patient who has everything? A qualitative study of prescribing for multimorbidity in primary care. *British Journal of General Practice* 2015;65(632):e184-e91. doi: 10.3399/bjgp15X684001

167. Carrier H, Zaytseva A, Bocquier A, et al. GPs' management of polypharmacy and therapeutic dilemma in patients with multimorbidity: a cross-sectional survey of GPs in France. *British Journal of General Practice* 2019;69(681):e270-e78. doi: 10.3399/bjgp19X701801

168. Anderson K, Foster M, Freeman C, et al. Negotiating "Unmeasurable Harm and Benefit": Perspectives of General Practitioners and Consultant Pharmacists on Deprescribing in the Primary Care Setting. *Qualitative Health Research* 2017;27(13):1936-47. doi: 10.1177/1049732316687732

169. Mc Namara KP, Breken BD, Alzubaidi HT, et al. Health professional perspectives on the management of multimorbidity and polypharmacy for older patients in Australia. *Age and Ageing* 2016:ageing;afw200v2. doi: 10.1093/ageing/afw200

170. Turner JP, Edwards S, Stanners M, et al. What factors are important for deprescribing in Australian long-term care facilities? Perspectives of residents and health professionals. *BMJ Open* 2016;6(3):e009781. doi: 10.1136/bmjopen-2015-009781

171. Sinnige J, Korevaar JC, Van Lieshout J, et al. Medication management strategy for older people with polypharmacy in general practice: a qualitative study on prescribing behaviour in primary care. *British Journal of General Practice* 2016;66(649):e540-e51. doi: 10.3399/bjgp16X685681

172. Smith SM, O'Kelly S, O'Dowd T. GPs' and pharmacists' experiences of managing multimorbidity: a 'Pandora's box'. *British Journal of General Practice* 2010;60(576):e285-e94. doi: 10.3399/bjgp10X514756

173. Laursen J, Kornholt J, Betzer C, et al. General Practitioners' Barriers Toward Medication Reviews in Polymedicated Multimorbid Patients: How can a Focus on the Pharmacotherapy in an Outpatient Clinic Support GPs? *Health Services Research and Managerial Epidemiology* 2018;5:233339281879216. doi: 10.1177/2333392818792169

174. Tsoi CS, Chow JY, Choi KS, et al. Medical characteristics of the oldest old: retrospective chart review of patients aged 85+ in an academic primary care centre. *BMC Research Notes* 2014;7(1):340. doi: 10.1186/1756-0500-7-340

175. Tan YY, Papez V, Chang WH, et al. Comparing clinical trial population representativeness to real-world populations: an external validity analysis encompassing 43 895 trials and 5 685 738 individuals across 989 unique drugs and 286 conditions in England. *The Lancet Healthy Longevity* 2022;3(10):e674-e89. doi: 10.1016/S2666-7568(22)00186-6

176. Wallace E, Salisbury C, Guthrie B, et al. Managing patients with multimorbidity in primary care. *BMJ* 2015;350(jan20 2):h176-h76. doi: 10.1136/bmj.h176

For peer review only - http://bmjopen.bmj.com/site/about/guidelines.xhtml

177. Scott IA, Anderson K, Freeman CR, et al. First do no harm: a real need to deprescribe in older patients. *Med J Aust* 2014;201(7):390-2. doi: 10.5694/mja14.00146

For peer review only

List of figures and supplementary files.

Figure 1: A PRIMSA Flow Diagram illustrating search results

Supplementary file 1: Descriptions of the included 157 papers in the synthesis of the scoping review

For peer review only

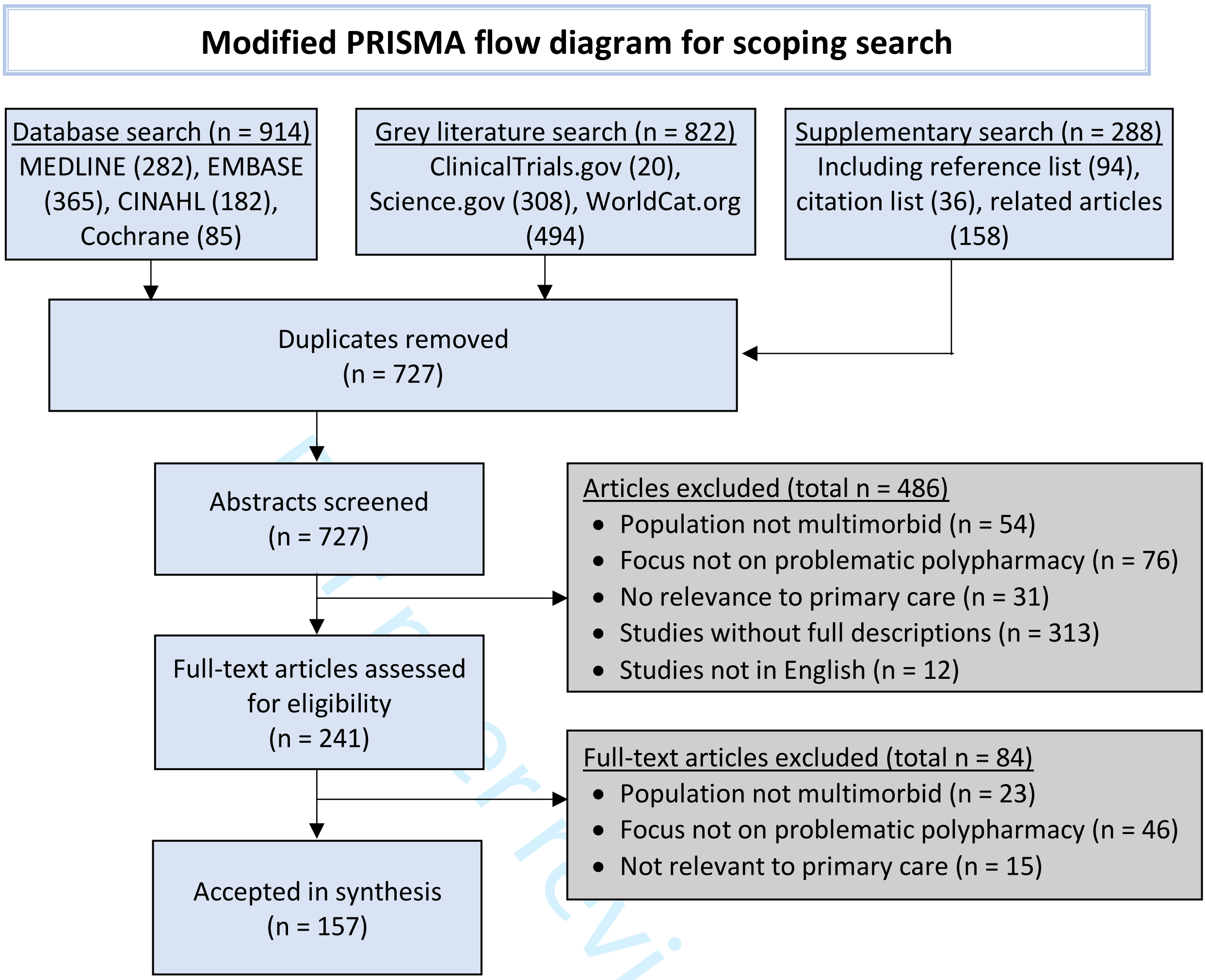
Modified PRISMA flow diagram for scoping search
Database search (n = 914)
MEDLINE (282), EMBASE (365), CINAHL (182), Cochrane (85)
Grey literature search (n = 822)
ClinicalTrials.gov (20), Science.gov (308), WorldCat.org (494)
Supplementary search (n = 288)
Including reference list (94), citation list (36), related articles (158)
Duplicates removed
(n = 727)
Abstracts screened
(n = 727)
Articles excluded (total n = 486)
Population not multimorbid (n = 54)
Focus not on problematic polypharmacy (n = 76)
No relevance to primary care (n = 31)
Studies without full descriptions (n = 313)
Studies not in English (n = 12)
Full-text articles assessed for eligibility
(n = 241)
Full-text articles excluded (total n = 84)
Population not multimorbid (n = 23)
Focus not on problematic polypharmacy (n = 46)
Not relevant to primary care (n = 15)
Accepted in synthesis
(n = 157)

Supplementary file 1: Descriptions of the included 157 papers in the synthesis of the scoping review

Author & Year	Title	Country	Publication type	Key Polypharmacy definitions	Population	Intervention (if applicable)	Key findings/ conclusions
Ali et al. 2022	Interventions to address polypharmacy in older adults living with multimorbidity.	Canada	Review of reviews	• Concurrent use of multiple medications<br>• ≥5 chronic medicines	Older adults (all 64+ years) with chronic conditions taking 5 or more medication across 5 reviews	Any polypharmacy intervention in the primary care setting	• Polypharmacy interventions are associated with reductions in potentially inappropriate prescribing and improvements in medication adherence.<br>• However, there is limited evidence of their effectiveness for clinical and intermediate outcomes.
Anderson et al. 2019	A systematic overview of systematic reviews evaluating interventions addressing polypharmacy.	USA	Review of reviews	• Use of multiple medications daily<br>• Appropriate and inappropriate polypharmacy	Mainly older adults (≥ 65 years) from 6 systematic reviews (in a range of primary care, community, nursing home and secondary care settings)	Any intervention addressing polypharmacy	• Despite the low quality of evidence in the underlying primary studies, there was evidence polypharmacy interventions improved medication appropriateness.<br>• However, there was no consistent evidence of any impact on downstream patient-centred outcomes such as healthcare utilization, morbidity, or mortality.
Anderson et al. 2019	Effect of medication reconciliation interventions on outcomes: A systematic overview of systematic reviews.	USA	Review of reviews	• Many medications<br>• Focus on medication discrepancies	Adult or paediatric populations	Interventions that included medication reconciliation as a core part	• An overview of systematic reviews of medication reconciliation interventions found 9 high-quality systematic reviews.<br>• A minority of those reviews' conclusions were consistent with medication reconciliation alone having a measurable impact, and such conclusions were almost all based on very low-quality evidence.
Soler et al. 2019	Community-Level Pharmaceutical Interventions to Reduce the Risks of Polypharmacy in the Elderly: Overview of Systematic Reviews and Economic Evaluations	Brazil	Review of reviews	• Prescription of multiple drugs to the individual<br>• Adequate polypharmacy and inadequate polypharmacy	Mainly older adults (≥ 65 years) from 13 systematic reviews and 3 economic analyses (range of primary/secondary care settings)	Any pharmaceutical intervention to reduce risks of polypharmacy	• There is evidence of improvement in clinical, epidemiological, humanistic, and economic outcomes for various types of community-level pharmaceutical interventions,<br>• Differences in observed outcomes may be due to study designs, primary study sample sizes, risk of bias, difficulty in aggregating data, heterogeneity of indicators and quality of evidence
Page et al. 2016	The feasibility and effect of deprescribing in older adults on	Australia	Review and meta-analysis	• Use of many medications	34 143 older adults (≥65 years,	Any deprescribing intervention	• Although nonrandomized data suggested that deprescribing reduces mortality, deprescribing

For peer review only - http://bmjopen.bmj.com/site/about/guidelines.xhtml

	mortality and health: a systematic review and meta-analysis			• Focus on deprescribing	mean age 73.8) across 132 papers		was not shown to alter mortality in randomized studies.<br>• Mortality was significantly reduced when applying patient-specific interventions in randomized studies.
Hill-Taylor et al. 2016	Effectiveness of the STOPP/START (Screening Tool of Older Persons' potentially inappropriate Prescriptions/Screening Tool to Alert doctors to the Right Treatment) criteria: systematic review and meta-analysis of randomized controlled studies	Canada	Review & meta-analysis	• Focus on potentially inappropriate prescribing	1925 older adults (≥65 years) from 4 RCTs across four countries (both acute and long-term care settings)	Interventions involving prospective application of the STOPP and/or START criteria on medication profiles	• Meta-analysis found that the STOPP criteria reduced PIM rates in all four studies, but study heterogeneity (I2 = 86·7%) prevented the calculation of a meaningful statistical summary.<br>• We found evidence that use of the criteria reduces falls, delirium episodes, hospital length-of-stay, care visits (primary and emergency) and medication costs, but no evidence of improvements in quality of life or mortality.
Johansson et al. 2016	Impact of strategies to reduce polypharmacy on clinically relevant endpoints: a systematic review and meta-analysis	Austria	Review & meta-analysis	• ≥4 or ≥5 drugs<br>• Use of potentially inappropriate medication	10 980 older adults (65+ years) across 25 controlled trials (multiple settings)	Any intervention aimed at reducing inappropriate polypharmacy	• There is no convincing evidence that the strategies assessed in the present review are effective in reducing polypharmacy<br>• These strategies to reduce polypharmacy had no effect on all-cause mortality (odds ratio 1.02; 95% confidence interval 0.84, 1.23). Only single studies found improvements, in terms of reducing the number of hospital admissions, in favour of the intervention group.
Keller et al. 2024	Cumulative Update of a Systematic Overview Evaluating Interventions Addressing Polypharmacy	USA (multiple countries included)	Review & meta-analysis	• ≥5 medications<br>• Use of potentially inappropriate medication (PIMs) or potential prescribing omissions (PPOs)	Mainly older adults (mainly ≥65 years) across 179 trials (multiple settings)	Any intervention aimed at addressing polypharmacy	• There is little evidence of an association between polypharmacy-related interventions and reduced important clinical and health care use outcomes.<br>• More evidence is needed regarding which interventions are most useful and which populations would benefit most.
Kua et al. 2019	Health outcomes of deprescribing interventions among older residents in nursing homes: a systematic review and meta-analysis.	Malaysia	Review & meta-analysis	• Concurrent use of multiple medications<br>• Potentially inappropriate medications<br>• Focus on deprescribing	Older Residents (≥ 60 years) in Nursing Homes across 41 RCTs	Any deprescribing intervention (defined as either medication discontinuation, substitution, or reduction)	• Deprescribing interventions significantly reduced the number of residents with potentially inappropriate medications by 59%<br>• Compared to other deprescribing interventions, medication review–directed deprescribing had significant benefits on older residents in nursing homes. Further research is required to elicit

							other clinical benefits of medication review–directed deprescribing practice.
Seppala et al. 2022	Medication reviews and deprescribing as a single intervention in falls prevention: a systematic review and meta-analysis.	Netherlands	Review & meta-analysis	• Use of multiple medications<br>• Focus on deprescribing	Older adults (aged ≥ 60 years) across 49 studies (separate analyses for community, hospital, and long-term care settings)	Any medication review or deprescribing intervention as a single intervention in falls prevention	• In meta-analyses, no significant associations between medication reviews and fall outcomes were found in any of the settings.<br>• There was a trend for a lower number of fallers in the meta-analysis assessing medication reviews in long-term care.<br>• In a frail subgroup, medication review might be effective even as a single intervention.
Shrestha et al. 2021	Impact of deprescribing dual-purpose medications on patient-related outcomes for older adults near end-of-life: a systematic review and meta-analysis.	Australia	Review & meta-analysis	• Potentially inappropriate medications<br>• Focus on deprescribing	689 Older adults (mean 81.6-85.7 years) with at least one life-limiting illness and at least one potentially inappropriate dual-purpose medication (disease prevention and symptom control) across 5 studies	Any deprescribing intervention	• The deprescribing of DPMs lowered the risk of mortality (risk ratio (RR) = 0.59, 95% confidence interval (CI) = 0.44-0.79) and referral to acute care facilities (RR = 0.40, 95% CI = 0.22-0.73), but did not have a significant impact on the risk of falls, non-vertebral fracture, emergency presentation, unplanned hospital admission, or general practitioner visits<br>• Insufficient good-quality studies powered to confirm a benefit in terms of quality of life, physical or cognitive function, health service utilisation and adverse events.
Tasai et al. 2021	Impact of Medication Reviews Delivered by Community Pharmacist to Elderly Patients on Polypharmacy: A Meta-analysis of Randomized Controlled Trials	Thailand	Review & meta-analysis	• ≥4 prescribed medications	4633 older adults (65+ years) across 4 studies	Interventions incorporating medication reviews delivered by community pharmacist	• When compared with usual care, medication reviews provided by community pharmacist significantly reduced risk of ED visits (risk ratio = 0.68; 95% CI = 0.48–0.96)
Alldred et al. 2016	Interventions to optimise prescribing for older people in care homes.	UK	Review	• ≥4 regular medicines<br>• Focus on appropriate & inappropriate polypharmacy (Beers, STOPP/START, MAI)	10,953 older residents (≥ 65 years) in 355 care homes in 10 countries.	Any intervention to optimise overall prescribing	• Could not draw robust conclusions from the evidence due to variability in design, outcomes and results<br>• The interventions implemented in the studies in this review led to the identification and resolution of medication-related problems and improvements in medication appropriateness

For peer review only

							• Evidence of a consistent effect on resident-related outcomes was not found.
Anderson et al. 2014	Prescriber barriers and enablers to minimising potentially inappropriate medications in adults: a systematic review and thematic synthesis	Australi a	Review	• Use of multiple medications concurrently<br>• ≥5 prescription, over-the-counter or complementary medicines every day<br>• potentially inappropriate medications	Adults of all ages, with some focus on older adults with chronically prescribed potent ially inappropriate medications across 21 studies (18 in primary care, 2 in residential care, 1 in secondary care)	N/A	• Barriers and enablers to minimising PIMs emerged within four analytical themes: problem awareness; inertia secondary to lower perceived value proposition for ceasing versus continuing PIMs; self-efficacy regarding personal ability to alter prescribing; and feasibility of altering prescribing in routine care given external constraints.<br>• The first 3 themes are intrinsic to the prescriber (e.g., beliefs, attitudes, knowledge, skills, behaviour) and the fourth is extrinsic (e.g., patient, work setting, health system and cultural factors).
Cadogan et al. 2013	Appropriate Polypharmacy and Medicine Safety: When Many is not Too Many	UK (NI), Ireland	Review	• Use of multiple medicines<br>• Balancing 'too little' and 'too many'<br>• Focus on appropriate polypharmacy	Middle-aged and older population with multimorbidity	N/A	• Differentiating between 'many' drugs and 'too many' drugs is proving ever more complex.<br>• Conceptualising polypharmacy as a numerical threshold is unhelpful because it fails to consider that the appropriate number of medicines varies according to individual patients' clinical needs<br>• Increased use of the term 'appropriate polypharmacy' could encourage greater consideration of the clinical context underlying prescribing, as well as increased acceptance that the prescribing of multiple medicines is 'potentially problematic rather than always inappropriate'.
Cairo Notari et al. 2021	Understanding GPs' clinical reasoning processes involved in managing patients suffering from multimorbidity: A systematic review of qualitative and quantitative research	Switzerl and	Review	• Multiple medicines and associated harmful effects<br>• Focus on deprescribing	Adults with multimorbidity (≥2 chronic conditions) across 32 studies	N/A	• In the absence of guidelines adapted to multimorbidity, there is no single correct plan, but competing priorities and unavoidable uncertainties.<br>• Thus, GPs have to consider and weigh multiple factors simultaneously. In the context of multimorbidity,<br>• GPs describe their reasoning as essentially intuitive and seem to perceive it as less accurate.<br>• These clinical reasoning processes are nevertheless more analytical than they might think and rooted in deep knowledge of the individual patient.

For peer review only

Cole et al. 2023	Interventions to improve the appropriate use of polypharmacy for older people	UK (Ireland), included trials from 19 countries	Review	• Polypharmacy (4 or more medicines), which used a validated tool to assess prescribing appropriateness<br>• Use of potentially inappropriate medication (PIMs) or potential prescribing omissions (PPOs)	18,073 older adults (aged ≥ 65) from 38 studies	Any intervention to improve appropriate polypharmacy	• It is unclear whether interventions to improve appropriate polypharmacy resulted in clinically significant improvement (including medication appropriateness, PIMs, PPOs, hospital admissions, quality of life, medication related problems).<br>• Since the last update in 2018, there appears to have been an increase in the number of studies seeking to address potential prescribing omissions and more interventions being delivered by multidisciplinary teams.
Cooper et al. 2015	Interventions to improve the appropriate use of polypharmacy in older people: a Cochrane systematic review	UK (Northern Ireland)	Review	• ≥4 regular medicines<br>• Focus on appropriate & inappropriate polypharmacy (Beers, STOPP/START, MAI)	Older adults (≥ 65 years) with ≥ 1 long-term condition who were receiving polypharmacy (≥ 4 regular medicines) from 12 studies (5 in community care) across 5 countries	All interventions improving appropriate polypharmacy.	• The included 12 interventions demonstrated improvements in appropriate polypharmacy based on reductions in inappropriate prescribing.<br>• However, it was unclear if interventions resulted in clinically significant improvements
Croke et al. 2023	The effectiveness and cost of integrating pharmacists within general practice to optimize prescribing and health outcomes in primary care patients with polypharmacy: a systematic review	Ireland	Review	• ≥5 regular medications<br>• Potentially inappropriate prescribing (any explicit/implicit criteria)	23,516 community dwelling adults (≥18 years) and in the primary care setting with polypharmacy (23 studies in 3 continents)	Any intervention involving pharmacist integration within General Practice (Including remote integration)	• Pharmacist integration probably reduced PIP and number of medications however, there was no clear effect on other patient outcomes<br>• While interventions in a small number of studies appeared to be cost-effective, further robust, well-designed cluster RCTs with economic evaluations are required to determine cost-effectiveness of pharmacist integration.
Dills et al. 2018	Deprescribing Medications for Chronic Diseases Management in Primary Care Settings: A Systematic Review of Randomized Controlled Trials	USA	Review	• Use of multiple prescription drugs<br>• Focus on deprescribing	Adults in a range of settings (primary care, assisted living, nursing home, outpatient and acute care settings only if medications for chronic disease	Deprescribing interventions involving chronic medical and mental health conditions managed by primary care professionals	• Deprescription may be successful and effective in select classes of drugs, with collaboration of clinical pharmacists for patient and provider education, and patient-specific drug recommendations, complemented by close clinical follow-up to detect early signs of exacerbation of chronic diseases.<br>• Deprescription may (1) require expensive intensive, ongoing interventions by clinical teams; (2) not lead to expected outcomes such as improved falls rate, cognition, and quality of

For peer review only

					were deprescribed		life, or a lower admission rate; and (3) have unexpected adverse outcomes affecting patients' quality of life.
Doherty et al. 2020	Barriers and facilitators to deprescribing in primary care: a systematic review	UK	Review	• Multiple concurrent medications<br>• ≥4 of any type of medications<br>• Focus on deprescribing	Adults (aged ≥18 years) with multimorbidity and polypharmacy (≥2 long-term health conditions), across 40 studies from 14 countries	Any deprescribing intervention	A whole system, patient-centred approach to safe deprescribing interventions is required, involving key decision-makers, healthcare professionals, patients, and carers.
Hasan Ibrahim et al. 2021	A systematic review of general practice-based pharmacists' services to optimize medicines management in older people with multimorbidity and polypharmacy	UK (Northern Ireland)	Review	• Concomitant use of ≥4 medicines<br>• Focus on medicines optimisation	Older adults (≥65 years) with multimorbidity (≥2 long-term conditions) across 7 studies in 5 countries	Practice-based pharmacists optimizing medicines management for older people with both multimorbidity and polypharmacy	• All studies employed pharmacist-led medication reviews accompanied by recommendations agreed and implemented by GPs.<br>• The limited available evidence suggested that, in collaboration with other practice team members, had mixed effects on outcomes focused on optimizing medicines management for older people.<br>• Most included studies were of poor quality and data to estimate the risk of bias often missing.
Heaton et al.	Person-centred medicines optimisation policy in England: an agenda for research on polypharmacy.	UK	Review	• Use of multiple medications<br>• 'Appropriate' and 'problematic' polypharmacy	Patients using multiple medicines in the UK	N/A	Reports varied in their inclusion of patient perspectives and person-centred care values, and in the extent to which they drew on evidence from research on patients' experiences of polypharmacy and medicines use.
Ibrahim et al. 2021	A systematic review of the evidence for deprescribing interventions among older people living with frailty.	UK	Review	• ≥5 regular medications<br>• Focus on deprescribing	657 older adults with frailty (mean age 79–87 years)	Any deprescribing medication review intervention accounting for at least 50% of changes	• All studies described medication-related outcomes and reported a reduction in potentially inappropriate medications and total number of medications per-patient.<br>• Feasibility of deprescribing in 4 studies which showed that 72–91% of recommendations made were implemented.<br>• 2 studies evaluated and reported acceptability of their interventions and 2 described cost savings.<br>• But there was a paucity of research about the impact of deprescribing in older people living with frailty

For peer review only

Kaufmann et al. 2014	Inappropriate prescribing: a systematic overview of published assessment tools.	Switzerland	Review	• Use of multiple medicines<br>• Focus on appropriate and inappropriate prescribing	Adults (majority older adults) with potentially inappropriate prescribing	N/A	• Out of 46 tools to assess inappropriate prescribing. 28 (61%) were explicit, 8 (17%) were implicit and 10 (22%) used a mixed approach.<br>• 36 tools named older people as target patients and 10 (22%) tools did not specify the target age group.<br>• Only 9 tools (19.5%) focused on patients in ambulatory care and 6 (13%) were developed for use in long-term care. 27 (59%) tools did not specify the health care setting.
Khezrian et al. 2020	An overview of prevalence, determinants and health outcomes of polypharmacy.	UK (Scotland)	Review	• No generally accepted definition<br>• Numerical (e.g. ≥ 5 medications concurrently or excessive polypharmacy ≥ 10 medications concurrently)<br>• Descriptive methods (patients visiting multiple pharmacies to obtain medications' and 'use of additional medications to correct adverse effects)<br>• Appropriate vs inappropriate polypharmacy	Older adults	N/A	• Polypharmacy was most often defined in terms of the number of medications that are being taken by an individual at any given time.<br>• Our review showed that the prevalence of polypharmacy varied between 10% to as high as around 90% in different populations.<br>• Chronic conditions, demographics, socioeconomics and self-assessed health factors were independent predictors of polypharmacy.<br>• Polypharmacy was reported to be associated with various adverse outcomes after adjusting for health conditions.
Laberge et al. 2021	Economic Evaluations of Interventions to Optimize Medication Use in Older Adults with Polypharmacy and Multimorbidity: A Systematic Review	Canada	Review	• Consumption of multiple medications simultaneously<br>• use of ≥5 medications<br>• potentially inappropriate medications and adverse drug reactions	6375 older adults (≥65 years) with multimorbidity (2+ conditions) from 11 studies across 8 countries (settings included primary care, nursing home, pharmacies, and secondary care)	Any intervention aimed at optimizing medication use	• Interventions were generally associated with a reduction in medication expenditure. The benefits of the intervention in terms of clinical outcomes remain limited.<br>• Five studies were cost-benefit analyses, which had a net benefit that was either null or positive.<br>• However, the quality of the studies was generally low.

For peer review only

Lee et al. 2020	Intervention elements to reduce inappropriate prescribing for older adults with multimorbidity receiving outpatient care: a scoping review.	Singapore	Review	• ≥5 medications daily<br>• Focus on potentially inappropriate prescribing	Older adults with multimorbidity	Interventions to reduce inappropriate prescribing	• 4 intervention elements were identified. An average of 2-3 to three elements were adopted in each intervention. The three most frequently adopted intervention elements were medication review (70%), training (26.3%) and tool/instrument(s) (22.5%).<br>• The 14 intervention elements were mapped onto five intervention functions: 'education', 'persuasion', 'training', 'environmental restructuring' and 'enablement'
Lee et al. 2022	Applicability of explicit potentially inappropriate medication lists to the Australian context: A systematic review.	Australia	Review	Focus on potentially inappropriate medications	Older adults prescribed potentially inappropriate medications	N/A	• Applicability of each explicit list ranged from 50-96% according to medications available in Australia and 25-83% according to medications available under subsidy.<br>• Pooling data from different lists may help to identify potentially inappropriate medications that may be applicable to local settings.
Lee et al. 2023	Factors associated with potentially inappropriate prescribing among older persons in primary care settings: Systematic review	Singapore	Review	• Focus on Potentially inappropriate prescribing (PIP)	2,893,925 older adults (≥60 years, mean 70-84 years) from 25 studies	N/A	• Risk factors of PIP could be classified into patient, physician and system factors.<br>• Patient factors were related to patient demographics (advanced age, lower education level and lower socioeconomic status), medical comorbidities (polypharmacy and multimorbidity) and lifestyle factors (unhealthy habits and use of over-the-counter medications).<br>• Physician and system factors included older, male, solo general practitioner (GP), higher number of visits of pharmaceutical sales representatives to GP, centrally located GP practice, and smaller number of older patients following up with GP, and medication source from public health system.
Lucchetti et al. 2017	Inappropriate prescribing in older persons: A systematic review of medications available in different criteria	Brazil	Review	Focus on potentially inappropriate medications	Older adults prescribed potentially inappropriate medications	N/A	• More than 85% used a Delphi method.<br>• There were 729 different medications/classes reported in all criteria.<br>• Diazepam was included in all 14 criteria followed by amitriptyline (13 criteria) and doxepin (12 criteria).<br>• Benzodiazepines, NSAIDs, antihistamines and antipsychotics were the most common drugs reported as potentially inappropriate for older persons.

For peer review only

Maidment et al. 2020	Towards an understanding of the burdens of medication management affecting older people: the MEMORABLE realist synthesis	UK	Review	• ≥5 medications<br>• Or if less – a complex regime	Older adults (≥ 60) years with multimorbidity (≥2 long term conditions) and polypharmacy	Medication management as a extended, complex process	• Older people and family carers often find medication management challenging and burdensome particularly for complex regimens.<br>• Practitioners need to be aware of this potential challenge, and work with older people and their carers to minimise the burden associated with medication management.
Mair et al. 2020	Addressing the Challenge of Polypharmacy.	UK (Scotland)	Review	• Use of multiple medications<br>• Appropriate and inappropriate polypharmacy	Older adults with multimorbidity and polypharmacy	N/A	• Guidance is needed to support patients and clinicians in defining and achieving realistic goals of drug treatment, and system change is necessary to aid implementation.
Masnoon et al 2018	Tools for Assessment of the Appropriateness of Prescribing and Association with Patient-Related Outcomes: A Systematic Review.	Australia	Review	• Multiple medicines prescribed together<br>• Focus on appropriate and inappropriate prescribing	Adults (mainly ≥45 years) with multimorbidity and polypharmacy	N/A	• Out of the 42 tools, 78.6% (n = 33) provided guidance around stopping inappropriate medications, 28.6% (n = 12) around starting appropriate medications, 61.9% (n = 26) were explicit (criteria based) and 31.0% (n = 13) had been externally validated, with hospitalisation being the most commonly used patient-related outcome (n = 9, 21.4%).<br>• Less than 50% of available tools have been externally validated, limiting their use in clinical practice.
Masnoon et al.	What is polypharmacy? A systematic review of definitions.	Australia	Review	• 138 definitions of polypharmacy and associated terms were obtained.<br>• 111 numerical only definitions (80.4% of all definitions), 15 numerical definitions which incorporated a duration of therapy or healthcare setting (10.9%) and 12 descriptive definitions (8.7%).	Older adults with multimorbidity and polypharmacy	N/A	• Polypharmacy definitions were variable.<br>• Numerical definitions of polypharmacy did not account for specific comorbidities present and make it difficult to assess safety and appropriateness of therapy in the clinical setting.
Michiels-Corsten et al. 2020	Generic instruments for drug discontinuation in primary care: A systematic review	Germany	Review	• Concurrent use of several (mostly ≥5) long-term medications	Adults in primary care	Any generic guiding instruments for drug discontinuation in	• Instruments revealed diverging emphases on the stages of deprescribing, i.e. preparation, drug evaluation, decision-making and implementation. Accordingly, 3 types of instruments emerged: general frameworks,

For peer review only

				• Potentially inappropriate medications<br>• Focus on discontinuation/ deprescribing		patients with polypharmacy in the primary care setting.	detailed drug assessment tools and comprehensive discontinuation guidelines.<br>• There is still a need for practical and user-friendly tools that support physicians in communicational aspects, visualise trade-offs and also enhance patient involvement.
Monégat et al, 2014	Polypharmacy: definitions, measurement and stakes involved	France	Review	Simultaneous, cumulative, and continuous polypharmacy) examined according to different thresholds – mainly ≥ 5 medications	69,324 older adults (aged 75+) from French GPs	N/A	• These definitions can used together, provide a broader overview of the use of medication.<br>• However, if polypharmacy is generally related to inappropriate prescribing, it is not sufficient on its own to identify them. Counting the number of medications does not allow distinguishing between those that are justified for a given pathology and those that are not.
Rankin et al. 2018	Interventions to improve the appropriate use of polypharmacy for older people.	UK (Ireland), trials from 12 countries	Review	Polypharmacy (4 or more medicines), which used a validated tool to assess prescribing appropriateness	28,672 older adults (aged 65+) from 32 studies	Any intervention to improve appropriate polypharmacy	• Unclear whether interventions resulted in clinically significant improvements<br>• May be slightly beneficial in terms of reducing potential prescribing omissions; but this effect estimate is based on only two studies, which had serious limitations in terms of risk bias.
Reeve et al. 2022	Deprescribing medicines in older people living with multimorbidity and polypharmacy: the TAILOR evidence synthesis	UK	Review	• Concurrent use of multiple medicines in a single person<br>• Use of ≥5 medications<br>• Appropriate and problematic polypharmacy<br>• Focus on deprescribing	Adults (aged ≥ 50 years) with multimorbidity (≥2 conditions)	Deprescribing interventions	• Deprescribing is a complex intervention<br>• Need to integrate patient-centred and contextual factors into best practice models.<br>• 34 context-mechanism-outcome configurations describe the knowledge work of tailored prescribing under eight headings related to organisational, health-care professional and patient factors, and interventions to improve deprescribing.<br>• Robust tailored deprescribing requires attention to providing an enabling infrastructure, access to data, tailored explanations, and trust.
Reeve et al. 2013	Patient barriers to and enablers of deprescribing: a systematic review	Australia	Review	• Many medicines<br>• Focus on potentially inappropriate medication and deprescribing	Mainly adult with some paediatric studies across 21 studies.	Stopping a medication/ deprescribing	• Three themes: disagreement/ agreement with 'appropriateness' of cessation, absence/presence of a 'process' for cessation, and negative/positive 'influences' to cease medication, were identified as both potential barriers and enablers, with 'fear' of cessation and 'dislike' of medications as a fourth barrier and enabler, respectively.<br>• The most common barrier/enabler identified was 'appropriateness' of cessation, with 15

For peer review only

							studies identifying this as a barrier and 18 as an enabler.
Reeve et al. 2014	Review of deprescribing processes and development of an evidence-based, patient-centred deprescribing process	Australia	Review	• Many medicines<br>• Focus on inappropriate medication use and deprescribing	Mainly focussed on older adults (≥65 years) with one study range 25-75) with polypharmacy across 10 studies	Deprescribing	• The developed patient-centred deprescribing process, which is a 5-step cycle: gaining a comprehensive medication history, identifying potentially inappropriate medications, determining whether the potentially inappropriate medication can be ceased, planning the withdrawal regimen (e.g. tapering where necessary) and provision of monitoring, support and documentation.<br>• This process focuses on engaging patients throughout the process, with the aim of improving long-term health outcomes.
Reeve et al. 2015	A systematic review of the emerging definition of 'deprescribing' with network analysis: implications for future research and clinical practice	Australia	Review	• Use of multiple medications<br>• Focus on deprescribing	Older adults prescribed inappropriate medications	N/A	• 8 characteristics of definitions: stop/withdraw/cease/discontinue, aspect of prescribing included e.g. long term therapy/ inappropriate medications, use of the term 'process' or 'structured', withdrawal is planned/supervised/judicious, involving multiple steps, includes dose reduction/ substitution/tapering, desired goals/ outcomes.<br>• "Deprescribing is the process of withdrawal of an inappropriate medication, supervised by a health care professional with the goal of managing polypharmacy and improving outcomes."
Riordan et al. 2016	The effect of pharmacist-led interventions in optimising prescribing in older adults in primary care: A systematic review	Ireland	Review	• Focus on potentially inappropriate prescribing and medication related problems	Community-dwelling older adults (≥65 years) across 5 studies	Any pharmacist-led intervention designed to reduce potentially inappropriate prescribing or improve medication appropriateness in primary care.	• Overall, this review demonstrates that pharmacist-led interventions may improve prescribing appropriateness in community-dwelling older adults.<br>• However, the quality of evidence is low.
Sanchez-Fidalgo et al. 2017	Prevalence of drug interactions in elderly patients with multimorbidity in primary care.	Spain	Review	• Use of multiple medications<br>• Focus on drug interactions	Older adults (aged 65+) in primary care	N/A	• The prevalence of drug-drug interactions in the elderly with multimorbidity is high, with ACEIs, diuretics and NSAID being the most common therapeutic groups involved

For peer review only - http://bmjopen.bmj.com/site/about/guidelines.xhtml

Sawan et al. 2020	A systems approach to identifying the challenges of implementing deprescribing in older adults across different health-care settings and countries: a narrative review	Australia	Review	• Medications for which the risk outweighs the benefit in the individual<br>• ≥5 regularly prescribed medications<br>• Focus on deprescribing	Older adults (≥ 65 years) with multimorbidity (≥2 chronic diseases) and polypharmacy	N/A	• Deprescribing intervention studies are inherently heterogeneous because of the complexity of interventions employed and often do not reflect the real-world.<br>• Process evaluations in deprescribing intervention studies are needed to determine the contextual factors that are important to the translation of the interventions in the real-world.<br>• Deprescribing interventions may need to be individually tailored to target the unique barriers and opportunities to deprescribing in different clinical settings.<br>• Introduction of national policies to encourage deprescribing may be beneficial but need to be evaluated to determine if there are any unintended consequences.
Schiavo et al. 2022	A comprehensive look at explicit screening tools for potentially inappropriate medication: A systematic scoping review.	Brazil	Review	Focus on potentially inappropriate medications	Older adults prescribed potentially inappropriate medications	N/A	• Fifty-eight tools reported 614 PIMs and 747 PIMs–interactions.<br>• Limited overlap between the tools was observed: 123 (69.1%) of 178 therapeutic alternatives proposed by the tools were considered inappropriate by other tools, and 222 (36.1%) of the 614 PIMs identified were named as being inappropriate only once.<br>• Only 21 tools were developed by a Delphi panel technique associated with systematic review.<br>• The PIMs listed as essential medication in Brazil and by the WHO were 30.6% and 23.3% of the total reported, respectively.
Scott et al. 2017	Review of structured guides for deprescribing	Australia	Review	• Appropriate and inappropriate polypharmacy<br>• Focus on deprescribing	Older adults prescribed potentially inappropriate medications in both secondary and primary care	N/A	• The 7 included guides had considerable heterogeneity, with some guides constituting little more than a set of principles while others entail detailed processes and sub-steps which addressed multiple determinants of drug appropriateness<br>• Evidence of effectiveness for each guide was limited in that none have been evaluated in RCTs, and pilot or feasibility studies have involved relatively small samples<br>• More research is needed for determining effectiveness and ease of use in routine clinical practice, especially in primary care settings.

For peer review only - http://bmjopen.bmj.com/site/about/guidelines.xhtml

Sirois et al.	Polypharmacy definitions for multimorbid older adults need stronger foundations to guide research, clinical practice and public health	Canada	Review	• > 46 definitions of polypharmacy<br>• Several thresholds based on count<br>• The majority of the publications (58%) used a minimal threshold of 5 medications.<br>• Heterogeneous qualitative definitions, mostly stating "more drugs than needed".	Older adults with multimorbidity and polypharmacy	N/A	• The wide variety of definitions for polypharmacy precludes comparisons, appropriate identification, and management of polypharmacy in multimorbid older adults.<br>• Standardized definitions would allow more coherent judgments regarding the individual and collective stakes of polypharmacy.
Taghy et al. 2020	Failure to Reach a Consensus in Polypharmacy Definition: An Obstacle to Measuring Risks and Impacts—Results of a Literature Review.	France	Review	2 main approaches:<br>• Quantitative, applying varying thresholds and types of polypharmacy based on the medication number<br>• Qualitative, based on the clinical indications and effects of a given drug regimen, with a growing number of descriptive characteristics	Polypharmacy in the elderly and the general population (1 included review in children also).	N/A	• The term "inappropriate" is increasingly associated with polypharmacy especially in studies that aimed to use this definition to identify possible solutions for healthcare providers in the field related to aging.<br>• High variability and an evolution in the approaches defining "polypharmacy" in the absence of a consensus following standardized criteria. That makes it very difficult to estimate and measure the outcomes associated with this phenomenon.
Ulley et al. 2019	Deprescribing interventions and their impact on medication adherence in community-dwelling older adults with polypharmacy: a systematic review	UK	Review	• Use of any potentially inappropriate medication<br>• Focus on deprescribing<br>• Focus on adherence	Adults (46-97 years) that are community dwelling from 22 studies in 13 countries	Any deprescribing intervention	• There is insufficient evidence to show that deprescribing improves medication adherence. Only 13 studies (of 22) reported adherence of which only 5 were randomised controlled trials.<br>• Adherence was reported as a secondary outcome in all but one study.
Verma et al. 2023	An Overview of Systematic Reviews and Meta-Analyses on the Effect of Medication Interventions Targeting	Sweden	Review	• Use of multiple medications<br>• ≥5 or ≥10 medications for more than 90 days	Frail older patients (≥60 years)	Any strategy targeting polypharmacy	• Six systematic reviews reported a statistically significant reduction in the number of inappropriately prescribed medications.<br>• Medication reviews help in reducing the use of inappropriate medications in frail older adults

	Polypharmacy for Frail Older Adults						• But there is insufficient evidence in terms of frailty score and hospital admissions.
Vrdoljak et al. 2015	Medication in the elderly - considerations and therapy prescription guidelines.	Croatia	Review	More than a few medications potentially inappropriate medications	Older adults with multimorbidity and polypharmacy	N/A	• Lists (e.g. START/STOPP and Beers) are an important practical support in a GP's everyday work.<br>• Implementation of such therapeutic aids reduces the possibility of medical error and minimizes the chance of an inappropriate prescription for this vulnerable population stratum.
Del Cura-González et al. 2022	How to Improve Healthcare for Patients with Multimorbidity and Polypharmacy in Primary Care: A Pragmatic Cluster-Randomized Clinical Trial of the MULTIPAP Intervention	Spain	RCT (cluster)	• Simultaneous consumption of ≥ 5 drugs<br>• Potentially inappropriate medication<br>• Focus on appropriateness (MAI)	593 older adults (between 65-74 years) with multimorbidity (≥3 diseases) and polypharmacy (≥5 drugs) during the last three months	MULTIPAP intervention: GPs e-training and GP-patient-centred interview.	• The intervention significantly improved medication appropriateness.<br>• The observed quality of life improvement was not significant.<br>• GPs e-training in multimorbidity has shown to be feasible and well accepted by the professionals.
Jager et al. 2017	Impact of a tailored program on the implementation of evidence-based recommendations for multimorbid patients with polypharmacy in primary care practices-results of a cluster-randomized controlled trial.	Germany	RCT (Cluster)	More than 4 drugs	273 adults (aged >50 years, suffering from at least 3 chronic diseases, receiving more than 4 drugs, and being at high risk for medication-related events according to the assessment of the treating GP)	The tailored program consisted of a workshop for GPs and health care assistants, educational materials and reminders for patients, and the elaboration of implementation action plans.	• The tailored program may improve implementation of medication counselling and brown bag review, whereas the use of medication lists and medication reviews did not improve.<br>• No effect of the tailored program on the combined primary outcome could be substantiated
Jungo et al. 2023	Optimising prescribing in older adults with multimorbidity and polypharmacy in primary care (OPTICA): cluster randomised clinical trial.	Switzerland	RCT (cluster)	• ≥5 long-term medications<br>• Focus on appropriate polypharmacy and potentially inappropriate medications – STOPP/START	323 older adults (≥ 65 years) with ≥3 conditions in Swiss primary care	eCDSS – with STOPP/START and STRIPA – a 6-step medication review conducted by GPs, followed by shared decision making	• No evidence of improvement in appropriateness of medication or a reduction in prescribing omissions at 12 months<br>• On average, one recommendation to stop or start a medication was reported to be implemented per patient.<br>• At 12 months, the results of the intention-to-treat analysis of the improvement in appropriateness of medication (odds ratio 1.05, 95% confidence interval 0.59 to 1.87) and the

For peer review only

							number of prescribing omissions (0.90, 0.41 to 1.96) were inconclusive.
McCarthy et al. 2022	GP-delivered medication review of polypharmacy, deprescribing, and patient priorities in older people with multimorbidity in Irish primary care (SPPiRE Study): A cluster randomised controlled trial	Ireland	RCT (Cluster)	• Multiple medicines use<br>• Higher levels of polypharmacy ≥ 15 medicines<br>• Focus on potentially inappropriate prescribing (STOPP/START)	404 older adults (≥65 years) taking ≥15 regular medicines from 51 GP practices	SPPiRE website: (educational module; template for an individualised patient med review that identified PIP, deprescribing opportunities, and patient priorities	The SPPiRE intervention resulted in a small but significant reduction in the number of medicines but no evidence of a clear effect on PIP.<br>At 6-month follow-up, both intervention and control groups had reductions in the numbers of medicines with a small but significantly greater reduction in the intervention group<br>Less than 2% of drug withdrawals in the intervention group led to a reported adverse drug events.
Muth et al. 2018	Effectiveness of a complex intervention on Prioritising Multimedication in Multimorbidity (PRIMUM) in primary care: results of a pragmatic cluster randomised controlled trial	German y	RCT (Cluster)	≥5 long-term drug prescriptions with systemic effects	505 older adults (≥60 years) with multimorbidity (≥3 chronic conditions) across 72 general practices in Hesse, Germany.	Healthcare assistant conducted a checklist-based interview with patients on medication-related problems and reconciled their medications. Then using a computerised decision support system, the GP optimised medication	• This study found the complex intervention to have no significant effects in older patients with multimorbidity and polypharmacy in general practice.<br>• At baseline, many patients already received appropriate prescriptions and enjoyed good quality of life and functional status.
Rieckert et al. 2020	Use of an electronic decision support tool to reduce polypharmacy in elderly people with chronic diseases: cluster randomised controlled trial.	German y	RCT (Cluster)	• ≥8 drugs<br>• Focus on inappropriate polypharmacy and adverse drug events	3904 older adults (≥75 years) using ≥8 medications across 359 practices (4 countries)	An electronic decision support tool comprising a comprehensive drug review to support general practitioners in deprescribing potentially inappropriate and non-evidence-based drugs.	• In intention-to-treat analysis, a computerised decision support tool for comprehensive drug review of elderly people with polypharmacy showed no conclusive effects on the composite of unplanned hospital admission or death by 24 months.<br>• A reduction in drugs was achieved without detriment to patient outcomes.

For peer review only

Schafer et al. 2018	Narrative medicine-based intervention in primary care to reduce polypharmacy: results from the cluster-randomised controlled trial MultiCare AGENDA	Germany	RCT (Cluster)	• Co-prescription of multiple medications<br>• Focus on the patient's perspectives	604 older adults (mean age 73 years) from 55 practices in 3 areas in Germany	GPs had 3x 30 min consultations.<br>1. aimed at identifying treatment targets and priorities of the patient.<br>2. 'brown bag' review<br>3. discuss goal attainment and future treatment targets.	Intensifying the doctor–patient dialogue and discussing the patient's agenda and personal needs did not lead to a reduction of medication intake and did not alter health-related quality of life.
Zechmann et al. 2020	Effect of a patient-centred deprescribing procedure in older multimorbid patients in Swiss primary care - A cluster-randomised clinical trial	Switzerland	RCT (cluster)	• Multiple medications<br>• ≥5 drugs for ≥6 months<br>• Focus on number, but also patient safety and QoL	334 older adults (≥60 years, mean 76.2) with multimorbidity across 46 GPs in North Switzerland	GP training encouraging the use of a validated deprescribing-algorithm including shared-decision-making	• The patient-centred deprescribing procedure is effective immediately after the intervention, but not after 6 and 12 months.<br>• Further research needs to determine the optimal interval of repeated deprescribing interventions for a sustainable effect on polypharmacy at mid- and long-term.
Koberlein-Neu et al. 2016	Interprofessional medication management in patients with multiple morbidities.	Germany	RCT (cluster, stepped wedge)	• Many medications<br>• ≥5 long-term medications (>3months)<br>• Focus on drug related problems	142 older adults (mean age 76.8 years) with multimorbidity (≥ 3 conditions) in 2 regions in Germany	Medication management and case management (including specialist home visit and pharmacist input)	• Interprofessional collaboration increased medication safety.<br>• Working across disciplinary boundaries allowed for a decrease in drug-related problems and brought up aspects outside the purview of the primary care physician.
Adamson et al. 2023	Medication work among nonagenarians: a qualitative study of the Newcastle 85+ cohort participants at 97 years old.	UK	Qualitative study (interviews)	• Use of multiple medicines	296 older adults (≥ 65 years)	Semi-structured interviews	• This study has shown a high level of acceptance of the work associated with medications among this group and trust in the prescribers to provide the most appropriate care.<br>• Medicines optimisation should build on this trust and be presented as personalised, evidence-based care.
Ie et al. 2023	Deprescribing as an Opportunity to Facilitate Patient-Centered Care: A Qualitative Study of	Japan	Qualitative study (focus group interviews)	• Focus on deprescribing	Older adults with multimorbidity and polypharmacy	8 Interview focus groups to 36 professionals (including 19 GPs	• Healthcare providers acted on the basis of their attitudes and beliefs on deprescribing, the influence of subjective norms, and perceived behavioural control for deprescribing.

	General Practitioners and Pharmacists in Japan.					and 16 clinical pharmacists)	• These processes are influenced by factors such as drug class, prescribers, patients, deprescribing experience, and environment/education.<br>• Healthcare providers' attitudes, beliefs, and behavioural control (along with deprescribing strategies) evolve in a dynamic interplay with experience, environment, and education
Knowles et al. 2018	Empowering people to help speak up about safety in primary care: Using codesign to involve patients and professionals in developing new interventions for patients with multimorbidity	UK	Qualitative (workshop)	• Large number of medications<br>• Complex medication schedule	11 patients with multimorbidity or carers (plus a second workshop with five HCPs)	An intervention to empower patients and carers to raise safety issues in primary care	• Both patients and professionals prioritized polypharmacy as a threat to safety.<br>• Findings emphasized the limited capacity of patients with multimorbidity and the need for services to proactively offer support to reduce the burden of managing complex treatment regimes.<br>• There is a need for accessible reminders to support medication adherence and medication reviews for particularly vulnerable patients conducted with pharmacists within GP practices.
Rozsnyai et al. 2020	What do older adults with multimorbidity and polypharmacy think about deprescribing? The LESS study - a primary care-based survey	Switzerl and	Qualitative (questionnai re)	• ≥5 chronic medications<br>• Focus on inappropriate medications and deprescribing	300 older adults (≥70 years,) with multimorbidity (≥3 chronic conditions) and polypharmacy	Deprescribing	• The majority of participants (77%) were willing to deprescribe one or more of their medicines if their doctor said it was possible. There was no association with sex, age or the number of medicines and willingness to deprescribe.<br>• Increased willingness to deprescribe was linked to a good relationship with their GP and that they would feel that deprescribing was safe and if new studies showed an avoidable risk.<br>• The most common barriers towards deprescribing were patients feeling well on their current medicines and being convinced that they need all their medicines.
Engels et al. 2023	Measurement of treatment burden in patients with multimorbidity in the Netherlands: translation and validation of the Multimorbidity Treatment Burden Questionnaire (NL-MTBQ).	Netherl ands	Qualitative (questionnai re with interviews)	• Focus on treatment burden<br>• Inappropriate polypharmacy	959 adults (17 - 96 years, mean = 69.9 years)	Multimorbidity Treatment Burden Questionnaire	• Median global NL-MTBQ score was 3.85 (interquartile range 0–9.62), representing low treatment burden.<br>• Factor analysis supported a single-factor structure.<br>• The Dutch version of the 13-item MTBQ is a single-structured, valid, and compact patient-reported outcome measure to assess treatment burden in primary care patients with multimorbidity.

For peer review only

Anthierens et al. 2010	Qualitative insights into general practitioners views on polypharmacy	Belgium	Qualitative (interviews)	• concomitant use of ≥3 drugs<br>• use of more drugs than indicated	People with polypharmacy	Interviews with 65 GPs	• It is a problem in their older patient population, especially because of the risk of adverse drug reactions, interactions and lowered adherence.<br>• Difficulties in keeping an overview of the exact medication intake<br>• Patients' strong belief in their medication and self-medication are seen as important barriers<br>• According to the respondents, prevention and evidence based medicine guidelines often induce polypharmacy.
Clyne et al. 2016	"Potentially inappropriate or specifically appropriate?" Qualitative evaluation of general practitioners' views on prescribing, polypharmacy and potentially inappropriate prescribing in older people	Ireland	Qualitative (interviews)	• Multiple medications<br>• Focus on Potentially inappropriate prescribing (PIP)	196 older adults (≥70 years) with multimorbidity (≥2 chronic medical conditions)	OPTI-SCRIPT (academic detailing with a pharmacist on conducting GP-led medicines review with participating patients; medicines reviews supported by web-based algorithms for GPs providing alternatives for specific PIP meds identified by the research pharmacist; and tailored patient information leaflets)	• 3 main, inter-related themes emerged (complex prescribing environment, paternalistic doctor-patient relationship, and relevance of PIP concept).<br>• Patient complexity (e.g. polypharmacy, multimorbidity), as well as prescriber complexity (e.g. multiple prescribers, poor communication, restricted autonomy) were all identified as factors contributing to a complex prescribing environment where PIP could occur, as was a paternalistic-doctor patient relationship.<br>• The concept of PIP was perceived to be of variable usefulness to GPs and the criteria to measure it may be at odds with the complex processes of prescribing for this patient population.
Laursen et al. 2018	General Practitioners' Barriers Toward Medication Reviews in Polymedicated Multimorbid Patients: How can a Focus on the Pharmacotherapy in an Outpatient Clinic Support GPs?	Denmar k	Qualitative (interviews)	• concomitant use of ≥2 drugs<br>• often arbitrarily set at cut-off value ≥5	People with polypharmacy and multimorbidity (≥2 chronic diseases)	Interviews with 14 GPs	• The primary barriers toward multimorbid patients with polypharmacy were the need for communication and teamwork with specialists<br>• Often, GPs felt that the specialists were more concerned about following standards and guidelines regarding specific diseases instead of a more holistic patient approach.<br>• To improve management of polypharmacy patients, the GPs suggest that a joint force is

For peer review only

							necessary, a partner-like relationship with greater transparency regarding information transfer, feedback, and shared decision-making.
Lee et al. 2023	Barriers and facilitators to deprescribing before surgery: A qualitative study of providers and older adults.	USA	Qualitative (interviews)	• ≥5 medications	8 older adults (mean 74 years) with polypharmacy (mean 8 medications)	Interviews with 8 primary care physicians in Maryland	• Facilitators and barriers both followed the following themes:<br>• Attitudes towards deprescribing before surgery, perceived benefits of deprescribing before surgery, patient-provider relationship and shared decision-making, hope for surgery, barriers to deprescribing before surgery, and preferences for deprescribing follow-up.
McNamara et al. 2017	Health professional perspectives on the management of multimorbidity and polypharmacy for older patients in Australia	Australi a	Qualitative (interviews)	• Using multiple medications<br>• Focus on appropriate medications management	Older adults (≥65 years) with multimorbidity and polypharmacy	Interviews with a range of 26 health professionals (14 prescribers and 12 non-prescribers, with 5 GPs, 5 hospital professionals, 6 nursing, 6 pharmacy staff)	• Most participants did not routinely use structured approaches to incorporate patients' preferences in clinical decision-making, address conflicting prescriber advice, assess patients' adherence to treatment plans or seek to optimise care plans.<br>• Challenges with coordination and continuity of care, pressures of workload and poorly defined individual responsibilities for care, all contributed to participants' avoiding ownership of multimorbidity management.
Schöpf et al. 2018	Elderly patients' and GPs' perspectives of patient–GP communication concerning polypharmacy: a qualitative interview study	German y	Qualitative (interviews)	• Concurrent prescription of at least 4 or 5 medications<br>• Appropriate medication therapy	6 older adults (≥65 years) along with 3 GPs in a single primary care centre in Germany	Communication about polypharmacy, safety and empowerment	• Patients' awareness of the significance of their active role in addressing polypharmacy needs to be increased.<br>• This includes understanding that trusting the doctor does not preclude asking questions or seeking more information.<br>• GPs might support patients by 'inviting' their contribution.<br>• We need interventions which improve patients' communication skills and address specific issues of polypharmacy, particularly in elderly patients
Sinnott et al. 2015	What to give the patient who has everything? A qualitative study of prescribing for multimorbidity in primary care	Ireland	Qualitative (interviews)	• Appropriate and inappropriate polypharmacy	51 adults (median 75 years, range 39-92 years) with multimorbidity (mean 8.3 conditions)	GP interviews	• Difficulties arose when recommendations or preferences conflicted, to which GPs responded by 'satisficing': accepting care that they deemed satisfactory and sufficient for a particular patient.<br>• Satisficing was manifest as relaxing targets for disease control, negotiating compromise with the patient, or making 'best guesses' about the most appropriate course of action.

For peer review only

							• In patients perceived as stable, GPs preferred to 'maintain the status quo' rather than rationalise medications, even in cases with significant polypharmacy.<br>• Proactive changes in medications were facilitated by continuity of care, sufficient consultation time, and open lines of communication.
Wallis et al. 2017	Swimming Against the Tide: Primary Care Physicians' Views on Deprescribing in Everyday Practice	New Zealand	Qualitative (interviews)	• Multiple medicines<br>• Focus on potentially inappropriate prescribing and deprescribing	People with polypharmacy and multimorbidity (many chronic conditions)	Interviews with 24 GPs	• Deprescribing was "swimming against the tide" of patient expectations, the medical culture of prescribing, and organizational constraints.<br>• It came with inherent risks for both themselves and patients and conveyed a sense of vulnerability in practice.<br>• The only incentive to deprescribing was the duty to do what was right for the patient.<br>• Physicians recommended organizational changes to support safer prescribing, including targeted funding for annual medicines review, computer prompts, improved information flows between prescribers, improved access to expert advice and user-friendly decision support, increased availability of non-pharmaceutical therapies, and enhanced patient engagement in medicines management.
Weir et al. 2021	The role of older patients' goals in GP decision-making about medicines: a qualitative study	Australi a	Qualitative (interviews)	• Taking ≥ 5 medicines<br>• Over-prescribing/ under-prescribing<br>• Inappropriate selection of a medication<br>• Avoidable adverse drug reactions	GPs caring for older patients with polypharmacy	N/A	• Most GPs recognised some value in understanding older patients' goals and preferences regarding their medicines.<br>• GPs differed on the following main themes: 1) definition and perception of patients' goals, 2) relationship with the patient, 3) approach to medicines management and prioritisation.<br>• We observed that GPs preferred one of three different practice patterns in their approach to patients' goals in medicines decisions: 1) goals and preferences considered lower priority – 'Directive'; 2) goals seen as central – 'Goal-oriented'; 3) goals and preferences considered but not explicitly elicited – 'Tacit'.
Zechmann et al. 2019	Barriers and enablers for deprescribing among older,	Switzerl and	Qualitative (interviews)	• > 5 drugs/day<br>• Inappropriate polypharmacy	19 older adults (mean age 76.9 years) with	Interviews	• We identified patient involvement in deprescribing and coordination of care as key issues for deprescribing

For peer review only

	multimorbid patients with polypharmacy: an explorative study from Switzerland			• Focus on benefit and harm ratio, admissions and mortality	multimorbidity taking 8.9 mean drugs per day.		• Conservatism/inertia and fragmented medical care were the main barriers towards deprescribing. No patient felt devalued as a consequence of the deprescribing offer.<br>• GPs concerns regarding patients' devaluation should not prevent them from actively discussing the reduction of drugs.
Anderson et al. 2017	Negotiating "Unmeasurable Harm and Benefit": Perspectives of General Practitioners and Consultant Pharmacists on Deprescribing in the Primary Care Setting	Australi a	Qualitative (focus groups)	• Use of more medicines than are clinically indicated or concurrent use of multiple medicines<br>• no consensus, but ≥5 commonly cited<br>• Focus on potentially inappropriate polypharmacy and deprescribing	Older adults (≥65 years) with polypharmacy who were still residing in the community	Focus group (32 GPs and 15 clinical pharmacists)	• Deprescribing is an inherently uncertain venture<br>• The option to deprescribe is shaped by many factors, including a clinician's perception of the risk/benefit ratio of persisting with the status quo versus deprescribing.<br>• It is more likely to occur in the presence of a continuous therapeutic relationship between the GP and patient and in response to a clear clinical trigger or finding of "low-hanging fruit."<br>• However, poorly developed interprofessional relationships and a lack of dedicated time and tacit knowledge/familiarity with patients, respectively, are important barriers to deprescribing.
Mangin et al. 2019	"I think this medicine actually killed my wife": patient and family perspectives on shared decision-making to optimize medications and safety	Canada	Qualitative (focus groups)	• ≥5 long-term medications<br>• Inappropriate medications<br>• Focus on benefit vs harms	16 adults (55-90 years) with multimorbidity (≥ 2 chronic conditions) from Southern Ontario and British Columbia (convenience sample)	How patient/family preferences and priorities are considered in medication-related discussions and decisions within the healthcare system	• Shared decision-making resulted from both recognition and integration of the personal expertise of the patient and family in medications, and perceived patient-centredness.<br>• It is complex, dynamic, and nonlinear, and patient priorities are not as integrated into shared decision-making about medications as we would hope.<br>• This is broadly consistent with the current conceptualization of evidence-based medicine.<br>• This suggests the need for developing a systematic process to elicit, record, and integrate patient preferences and priorities about medications to create space for a more patient-centred conversation.
Noel et al. 2005	Collaborative care needs and preferences of primary care patients with multimorbidity	USA	Qualitative (focus groups)	• Multiple medications<br>• Medication side effects and burdens	60 patients with ≥2 chronic illnesses (taking 8-27 medications) across 8 primary care clinics within	Any care need or preference for primary care patients with multiple chronic illnesses	• Polypharmacy was a major concern.<br>• Problematic interactions with providers and the health care system were also mentioned, often in relation to specialty care and included incidents in which providers had ignored concerns or provided conflicting advice

For peer review only - http://bmjopen.bmj.com/site/about/guidelines.xhtml

					the Veterans Health Administration.		• Knowledge and skills deficits interfered with self-management.<br>• Participants were willing to use technology for monitoring or educational purposes if it did not preclude human contact
Sinnige et al. 2016	Medication management strategy for older people with polypharmacy in general practice: a qualitative study on prescribing behaviour in primary care.	Netherl ands	Qualitative (Focus groups)	• Chronic use of ≥5 medications<br>• appropriate polypharmacy	Older adults (aged 68–84 years) diagnosed with multiple, highly prevalent chronic diseases, often part of a cluster of diseases	Focus groups with 12 GPs	• Similar strategy regarding the patients' medication management: defining treatment goals; determining primary goals; and adjusting medications based on the treatment effect, GPs' and patients' preferences, and patient characteristics.<br>• The GPs would like to discuss their choices with other professionals, and they valued structured medication reviews with the patient, as well as quick and practical support tools that work on demand.
Smith et al. 2010	GPs' and pharmacists' experiences of managing multimorbidity: a "Pandora's box"	Ireland	Qualitative (Focus groups)	• Multiple medications use<br>• ≥5 medications	People with multimorbidity	3 focus groups with 13 GPs and 7 pharmacists	• Themes included 1) the concept of multimorbidity and the link to polypharmacy and ageing; 2) health systems issues relating to lack to time, inter-professional communication difficulties, and fragmentation of care; 3) individual issues from clinicians relating to professional roles, clinical uncertainty, and avoidance; 4) patient issues; and 5) potential management solutions.
Uhl et al. 2018	Patient-perceived barriers and facilitators to the implementation of a medication review in primary care: a qualitative thematic analysis.	German y	Qualitative (focus groups and telephone interviews)	• ≥5 drugs per day<br>• Inappropriate polypharmacy	31 older adults (range 62–88 years), ≥3 chronic diseases, from 17 General practices in Frankfurt (convenience sample)	Medication review	• Barriers to patient participation concerned patient autonomy, while facilitators involved patient awareness of medication-related problems.<br>• Barriers to GP-led reviews concerned GP's lack of resources while facilitators related to the trusting relationship between patient and GP.<br>• Pharmacist-led reviews might be hindered by a lack of patients' confidence in pharmacists' expertise, but facilitated by pharmacies' digital records of the patients' medications.
Reeve et al. 2016	Beliefs and attitudes of older adults and carers about deprescribing of medications: a qualitative focus group study	Australi a	Qualitative (focus group)	• Many medicines<br>• Focus on inappropriate medication and deprescribing	14 older adults (mean age 75 years) and 14 carers (mean age 79) across 4 focus groups	Deprescribing	• Main factors: Their perception of the appropriateness of that medication; fear of outcomes of withdrawal; dislike of taking medications; and the availability of a process for withdrawal (including a discussion with a

For peer review only

							healthcare professional and knowing that the medication could be restarted if necessary).<br>• A patient's regular GP was identified as a strong influence both for and against.<br>• A theme unique to the carers was the complexity involved in making decisions about medications for their care recipients.
Bell et al. 2017	Nurses' and pharmacists' learning experiences from participating in interprofessional medication reviews for elderly in primary health care - a qualitative study	Norway	Qualitative (focus group and telephone interviews)	• ≥4 drugs<br>• Inappropriate drug use	Elderly patients with polypharmacy and multimorbidity in primary care	Interprofessional medication reviews	• Although experiencing challenges in conducting IMRs, the nurses and pharmacists had learning experiences they said improved both their own practice and the quality of drug management.<br>• There are some challenges concerning how to ensure participation of all three professions and how to get thorough information about the patient.
Collier et al. 2023	Older people, medication safety, and the role of the community pharmacist: a longitudinal ethnographic study	Australia	Qualitative (ethnography)	• ≥5 regular or 'as required', prescription or non-prescription medications<br>• Focus on medication safety	20 older adults (≥65 years) with frailty and multimorbidity	Community pharmacist involvement	• Community pharmacists play a significant role in the medication safety of older people with frailty and polypharmacy. Analysis resulted in three main themes:<br>1) the older person–determined role of the pharmacist,<br>2) the 'taken for granted' safety work of the pharmacist<br>3) collective agency and medication safety.
Fudge et al 2021	'It's all about patient safety': an ethnographic study of how pharmacy staff construct medicines safety in the context of polypharmacy	UK	Qualitative (ethnography)	• Concurrent use of ≥5 medications<br>• Focus on medication safety	33 pharmacy staff (counter staff, technicians, dispensers, pharmacists)	Community pharmacist involvement	• 'Safety' in the performance of practices relating to polypharmacy was not a fixed, defined notion, but an ongoing, collaborative accomplishment, emerging within an organisational narrative of 'care'.<br>• Despite meticulous attention to 'safety', carefully guarded professional boundaries meant that addressing polypharmacy per se in the context of community pharmacy was beyond reach.
Swinglehurst et al. 2021	Organising polypharmacy: unpacking medicines, unpacking meanings—an ethnographic study	UK	Qualitative (ethnography)	• Multiple medications<br>• ≥10 'higher-risk' polypharmacy<br>• Appropriate and inappropriate polypharmacy	24 older adults (≥65 years) with multimorbidity and ≥10 regular medications from 3 NHS GP Practices	Medication reviews	• Polypharmacy demands careful organising.<br>• All patients had developed strategies and routines for organising medicines into their lives, negotiating medicine taking to enable acceptable adherence and make their medicines manageable.

For peer review only - http://bmjopen.bmj.com/site/about/guidelines.xhtml

					(purposive sampling), ethnography also covered 4 community pharmacies		• Strategies adopted by patients often involved the use of 'do-it-yourself' dosette boxes.<br>• Patients incorporated a range of approaches to manage supplies and flex their regimens to align with personal values and priorities. Practices of organising medicines are effortful, creative and often highly collaborative.<br>• Patients strive for adherence, but their organisational efforts privilege 'living with medicines' over taking medicines strictly 'as prescribed'.
Swinglehurst et al. 2023	Negotiating the polypharmacy paradox: a video-reflexive ethnography study of polypharmacy and its practices in primary care	UK	Qualitative (ethnography)	• Multiple medications<br>• ≥10 'higher-risk' polypharmacy<br>• Appropriate and inappropriate polypharmacy	24 older adults (≥65 years) with multimorbidity and ≥10 regular medications from 3 NHS GP Practices (purposive sampling), ethnography also covered 4 community pharmacies	Medication reviews	• Participants rarely referenced biomedical aspects of prescribing (eg, drug-drug interactions, 'Numbers Needed to Treat/Harm') focussing instead on polypharmacy as an emotional and relational challenge. Clinicians initially denigrated their medication review work as mundane. Through VRE they reframed their work as complex, identifying polypharmacy as a delicate matter to negotiate.<br>• Medication review was identified as an ongoing process, rather than a discrete 'one-off' activity. Meaningful progress towards tackling polypharmacy was only possible through small, incremental, carefully supported changes in which both patient and clinician negotiated a sharing of responsibility, best supported by continuity of care.
McIntosh et al. 2018	A case study of polypharmacy management in nine European countries: Implications for change management and implementation.	Spain (authors consortium includes 8 EU countries)	Qualitative (case study)	• ≥5 medications<br>• Appropriate and inappropriate polypharmacy	Older adults (varied but all ≥50 years old), some specific to geriatric departments, or care homes	Varied identified initiatives across 5 sites: Spain (Catalonia), Germany (Lower Saxony), Sweden (Uppsala), and two UK sites (Northern Ireland and Scotland).	• Within the studied EU countries, polypharmacy management was not widely addressed.<br>• Results highlight the importance of change management and theory-based implementation strategies.
All Wales Medicines	Polypharmacy in older people: A guide for	UK (Wales)	Policy	• Use of multiple medications	Adults with polypharmacy	N/A	• Assessing polypharmacy and patient adherence to medication is imperative.

Strategy Group. 2023	healthcare professionals.			• Appropriate and problematic polypharmacy<br>• Particular high risk medications			• To support medicines optimisation in older patients who may be subject to inappropriate polypharmacy, deprescribing high risk medicines is practical
Australian Commission on Safety and Quality in Health Care. 2020	Polypharmacy, 75 years and over	Australia	Policy	≥5 medications at the same time	Older adults (≥75 years) with polypharmacy	N/A	• There are widespread variations in polypharmacy prevalence across the country<br>• Further guidance is needed for communication between medical teams and residential homes, medication management reviews and system changes including establishment, governance and advisory committees.
King's Fund	Polypharmacy and medicines optimisation.	UK	Policy	• Appropriate polypharmacy: prescribing for an individual for complex conditions or for multiple conditions in circumstances where medicines use has been optimised and where the medicines are prescribed according to best evidence.<br>• Problematic polypharmacy: prescribing multiple medications inappropriately, or where the intended benefit of the medication is not realised.	Adults with polypharmacy	N/A	• For many people, appropriate polypharmacy will extend life expectancy and improve quality of life. With meds optimised and prescribed according to best evidence.<br>• Problematic polypharmacy can cause an increased risk of drug interactions and adverse drug reactions, together with impaired adherence and QoL<br>• Many clinical trials and practice guidelines do not consider polypharmacy in the context of multi-morbidity.
Scottish Government Polypharmacy Model of Care Group. 2018	Polypharmacy guidance, realistic prescribing. 3rd Edition.	UK (Scotland)	Policy	• Many medications<br>• Appropriate and inappropriate polypharmacy	Adults with polypharmacy	N/A	• The case for effective polypharmacy management is quite clear, but in a complex healthcare setting with many competing priorities it is useful to outline the quality and economic reasons why it should be prioritised.

For peer review only - http://bmjopen.bmj.com/site/about/guidelines.xhtml

							• Need to address polypharmacy management as a public health issue, as multi-morbidities do not just affect the elderly.<br>• Greater emphasis has been placed on shared-decision making to actively engage the patient with the 7-Step medication review.<br>• The Drug Efficacy (NNT) tables help discussion regarding the relative potential benefits of a range of common therapeutic interventions.
The Department of Health and Social Care 2021	National overprescribing review report: Good for you, good for us, good for everybody	UK	Policy	• Overprescribing – the use of a medicine where there is a better non-medicine alternative, or the use is inappropriate for that patients' circumstances and wishes.<br>• Polypharmacy – the concurrent use of multiple medicines for one person. There is currently no consensus on a definition for polypharmacy.<br>• Prevalence calculated at 5+ and 8+.	Adults with polypharmacy	N/A	• Overprescribing is a serious problem in health systems internationally that has grown dramatically over the last 25 years, with systemic and cultural causes.<br>• Estimates at least 10% of medicines overprescribed
McCarthy et al 2020	The evolution of an evidence based intervention designed to improve prescribing and reduce polypharmacy in older people with multimorbidity and significant polypharmacy in primary care (SPPiRE)	Ireland	Pilot and protocol	• Multiple medicines use<br>• Higher levels of polypharmacy ≥ 15 medicines<br>• Focus on potentially inappropriate prescribing (STOPP/START)	10 older adults (≥65 years) taking ≥15 regular medicines	SPPiRE website: educational module template for an individualised patient medication review that identified PIP, deprescribing opportunities, and patient priorities	• A framework was used to systematically describe how and why the original intervention was modified, allowing the new intervention to build upon an effective and robustly developed intervention but also to be relevant in the context of the current evidence base.

For peer review only

McCarthy et al. 2017	Supporting prescribing in older people with multimorbidity and significant polypharmacy in primary care (SPPiRE): a cluster randomised controlled trial protocol and pilot.	Ireland	Pilot and protocol	• Multiple medicines use<br>• Higher levels of polypharmacy ≥ 15 medicines<br>• Focus on potentially inappropriate prescribing (STOPP/START)	10 older adults (≥65 years) taking ≥15 regular medicines	SPPiRE website: educational module template for an individualised patient medication review that identified PIP, deprescribing opportunities, and patient priorities	• The feasibility of the finder tool and intervention were assessed through GP feedback and analysis of use of the online material. Overall, the intervention was well received by the GPs and their patients many of whom reported feeling reassured that their medicines were being reviewed and rationalised.<br>• When the higher prevalence, lower risk proton pump inhibitor PIP was excluded, 90% of patients had at least 1 PIP and the mean number of PIP per patient was 1.1. Identified instances of PIP were acted on in 44% of cases and 45% when the proton pump inhibitor PIP was excluded.<br>• The most common patient priorities were treating pain, followed by fatigue, and reducing the number of repeat medicines.
Kirwan et al. 2022	The multimorbidity collaborative medication review and decision making (MyComrade) study: a pilot cluster randomised trial in two healthcare systems	UK and Ireland	Pilot (Cluster RCT)	• ≥10 medications	121 adults (≥18 years and over, with multi morbidity and ≥10 medications living in the community across 15 GP practices in Northern Ireland and Ireland	Training (Face-to-face or pre-recorded) and inclusion of practice-based pharmacists in NI as collaborative reviewers using the NO TEARS tool to encourage shared decision making	• Both practice staff and patients found the intervention acceptable and reported strong fidelity to the My Comrade intervention components.<br>• Some staff highlighted concerns such as poor communication of the reviews to patients, dissatisfaction regarding incentivisation and in ROI the sustainability of two GPs collaboratively conducting the medication reviews.<br>• Pairing of GP and pharmacist may be more sustainable to implement in routine practice.
Rankin et al. 2022	An external pilot cluster randomised controlled trial of a theory-based intervention to improve appropriate polypharmacy in older people in primary care (PolyPrime).	UK and Ireland	Pilot (Cluster RCT)	• ≥ 5 medicines<br>• ≥ 4 medications for ≥3 months<br>• Focus on potentially inappropriate prescribing (STOPP/START and NO TEARS)	120 older adults (≥ 70 years old) prescribed ≥4 medications for ≥3 months living in the community across 12 GP practices in Northern Ireland and Ireland	An online video (incorporating behaviour change) and scheduled medication reviews with patients on 2 occasions.	• The intervention was successfully delivered as intended; it was acceptable to GPs, practice staff, and patients; and potential mechanisms of action have been identified.<br>• It may be feasible to conduct an intervention to improve appropriate polypharmacy in older people in primary care across two healthcare jurisdictions.
Cardwell et al. 2020	Evaluation of the General Practice Pharmacist (GPP) intervention to	Ireland	Pilot	• Many medications<br>• Focus on potentially inappropriate prescribing, high risk	786 older adults (mean age 69.8 years) from 4 General practices	A pharmacist joined the practice team for 6 months and	An intervention involving pharmacists, working within general practices is feasible to implement and has potential to improve prescribing quality.

For peer review only

	optimise prescribing in Irish primary care: a non-randomised pilot study.			medications and deprescribing		undertook medication reviews (face to face or chart based), provided prescribing advice and facilitated practice-based education	
Michiels-Corsten et al. 2022	MediQuit – an electronic deprescribing tool: a pilot study in German primary care; GPs' and patients' perspectives	Germany	Pilot	• Multiple medications<br>• ≥ 5 drugs<br>• Excessive polypharmacy = ≥ 10 drugs<br>• Inappropriate polypharmacy<br>• Focus on overprescribing and deprescribing	41 older adults (mean age 77 years) with at least 3 chronic diseases (mean was 8) with ≥ 10 drugs in 2 regions of Germany	MediQuit - Electronic Deprescribing Tool (3 steps: 1. Med review with algorithm to flag PIM for deprescribing. 2. Communcation prompts, and risk-benefits. 3. Information on discontinuation process).	• Identification (step 1) and implementation elements (Step 3) were perceived most helpful by GPs. Whereas, shared-decision making elements (step 2) revealed room for improvement.<br>• Patients were broadly satisfied with the deprescribing consultation (85%) and with their decision made regarding their medication (90%).<br>• GPs were satisfied tool and gave important hints for future development.
Benson et al. 2021	Medication management for complex patients in primary care: application of a remote, asynchronous clinical pharmacist model	USA	Feasibility	• ≥ 5 medicines<br>• Focus on drug related problems (Indication, Effectiveness, Safety, and Compliance)	202 adults (aged 40+) with multimorbidity (≥2 chronic conditions) and high health expenditure	A remotely delivered Comprehensive Medication Management allowing pharmacists to make prescription changes (for 6 months)	• A clinical pharmacist found that 86% of participants had a drug therapy problem according to classification criteria. Seventy-nine percent of all drug therapy problems identified were resolved upon completion of the study.<br>• A service model using remote pharmacist services may be an effective means of improving team-based primary care medication management for this population.
Junius-Walker et al. 2021	MediQuit, an Electronic Deprescribing Tool for Patients on Polypharmacy: Results of a Feasibility Study in German General Practice	Germany	Feasibility	• Multiple medications<br>• ≥ 5 drugs<br>• Excessive polypharmacy = ≥ 10 drugs<br>• Inappropriate polypharmacy	41 older adults (mean age 77 years) with at least 3 chronic diseases (mean was 8) with ≥ 10 drugs in 2 regions of Germany	MediQuit - Electronic Deprescribing Tool (3 steps: 1. Med review with algorithm to flag PIM for deprescribing. 2.	• Deprescribing was achieved in 70% of consultations in agreement with patients.<br>• Drugs deprescribed were symptom-lowering and preventive drugs<br>• GPs found MediQuit useful in initiating communication on this issue and enhancing deliberations for a deprescribing decision.

For peer review only

				• Focus on overprescribing and deprescribing		Communication prompts, and risk-benefits. 3. Information on discontinuation process).	• GPs rated patient involvement higher than did patients themselves.
Mangin et al. 2023	Team approach to polypharmacy evaluation and reduction: feasibility randomized trial of a structured clinical pathway to reduce polypharmacy.	Canada	Feasibility	• ≥5 long-term medications<br>• Focus on appropriateness and patient preferences.	37 older adults (≥ 70 years) on ≥ 5 long-term medications	TAPER – a team based clinical pathway for a complete medication review by the pharmacist and the physician aimed at reducing medication burden	• Results from this feasibility study indicate that TAPER as a clinical pathway is feasible to implement in a primary care team setting and in an RCT research framework.<br>• Outcome trends suggest effectiveness.
Cahir et al. 2014	Potentially inappropriate prescribing and vulnerability and hospitalization in older community-dwelling patients	Ireland	Longitudinal study (retrospective cohort)	• Multiple medications<br>• Focus on potentially inappropriate prescribing (compares Beers and STOPP criteria)	931 community-dwelling patients aged ≥70 years in 15 general practices in Ireland	N/A	• The prevalence by the Beers 2012 and STOPP criteria was 28% (n = 246) and 42% (n = 377), respectively. Patients with ≥2 PIP indicators were almost twice as likely to be classified as vulnerable.<br>• STOPP is a more sensitive measure of PIP than the Beers 2012 criteria and of clinical benefit in primary care settings, particularly for hospital visits.
Doherty et al. 2023	Adverse drug reactions and associated patient characteristics in older community-dwelling adults: a 6-year prospective cohort study	Ireland	Longitudinal study (prospective cohort)	• ≥5 regular prescribed medications<br>• major polypharmacy (≥10 drug classes)	592 older adults (aged ≥ 70 years) across 15 practices in Ireland	N/A	• A total of 211 ADRs were recorded for 159 participants, resulting in a cumulative incidence of 26.9% over 6 years.<br>• The majority of ADRs detected were mild (89.1%), with the remainder classified as moderate (10.9%). Eight moderate ADRs, representing 34.8% of moderate ADRs and 3.8% of all ADRs, required an emergency hospital admission.<br>• ADRs were independently associated with female sex, polypharmacy (5–9 drug classes) and major polypharmacy (≥10 drug classes)
McCarthy et al. 2023	Medication changes and potentially inappropriate prescribing in older	Ireland	Longitudinal study (secondary analysis of	• Multiple medicines use<br>• Focus on potentially inappropriate	404 older adults (aged ≥ 65 years), prescribed ≥ 15 repeat medicines,	N/A	• There were reductions in the prescription of most drug groups with the largest reduction in antiplatelet prescriptions.

For peer review only

	patients with significant polypharmacy.		RCT at 2 time points)	prescriptions (SPPiRE PIP criteria)	from 51 different general practices		• Considering medication discontinuations, initiations and switches, there was a median of 5 medication changes per person<br>• A high proportion of benzodiazepine, anticholinergic and diuretic prescriptions were potentially inappropriate suggesting these higher risk groups may warrant specific attention.
O'Regan et al. 2023	How often do patients attend general practice, how often are they referred to hospital, and how do multi-morbidity and polypharmacy affect general practice attendance and referral rates?	Ireland	Longitudinal study (retrospective cohort)	• ≥5 regular medications	6603 older adults (50+ years) from 72 GP practices	N/A	• Increasing age, number of chronic illnesses and number of medications were associated with increased attendance rates to the GP and practice nurse and home visits but did not significantly increase the ratio of attendance to referral rate.<br>• General practice must be supported to provide person centred care to an ageing population with rising rates of multi-morbidity and polypharmacy.
Tampaki et al. 2023	Inappropriate prescribing in geriatric rural primary care: impact on adverse outcomes and relevant risk factors in a prospective observational cohort study.	Greece	Longitudinal study (prospective cohort)	• Use of potentially inappropriate medication (PIMs) or potential prescribing omissions (PPOs). [using STOPP/START]	104 older adults (≥ 65 years, median age 78 years) receiving a median of 6 drugs	N/A	• PPO was found in 78% and PIMs in 61%. PIM was multivariately correlated with multimorbidity (p = 0.029) and polypharmacy (p < 0,001), while drug-PPO was only associated with multimorbidity (p = 0.039).<br>• The number of PIM predicted emergency department visits and hospitalizations at 6-month follow-up (p value 0.011), independent of age, sex, frailty, comorbidities, and total medication number.
Muller et al. 2020	Development and internal validation of prognostic models to predict negative health outcomes in older patients with multimorbidity and polypharmacy in general practice.	Germany	Longitudinal study (prognostic modelling)	• Multiple medicines use<br>• Appropriate & inappropriate polypharmacy<br>• Focus on Quality of life	592 958 older adults (≥60 years, ≥5 drugs, ≥3 chronic diseases, excluding dementia) in Germany	N/A	• Best trial data-based model predicted HRQoL after 6 months well and included parameters of well-being not found in claims.<br>• Performance of claims data-based models and models predicting long-term outcomes was relatively weak.
Kruger et al. 2021	Non-random relations in drug use expressed as patterns comprising prescription and over-the-counter drugs in	Germany	Longitudinal study (retrospective cohort study)	Chronic co-prescription or co-application of different drugs at the same time	3189 older adults (65-85 years) with at least 3 chronic diseases	N/A	There are strong associations between drug patterns and multimorbidity clusters, which enrich the knowledge about the treatment of multimorbid elderly patients in primary care.

For peer review only - http://bmjopen.bmj.com/site/about/guidelines.xhtml

	multimorbid elderly patients in primary care: Data of the exploratory analysis of the multicentre, observational cohort study MultiCare.						
Carr et al 2021	A multidimensional measure of polypharmacy for older adults using the Health and Retirement Study	UK	Longitudinal study	• Concurrent use of multiple medications by one individual<br>• Added dimensions e.g. temporality, dosage, appropriateness, anticholinergic properties, potential drug interactions<br>• Cut offs of 5+ and 9+ used in comparative analyses	2141 adults (aged 50-80) living in the USA	N/A	• A four-class model was selected based on fit statistics and clinical interpretability.<br>• Participants in the 'low risk' class tended to be male, cohabitating, and reported fewer health conditions, compared to 'high risk' classes.<br>• The three 'high risk' classes overlapped with the groups concurrently taking 5+ and 9+ medications per month.
D'Aiuto et al. 2023	Health care system costs related to potentially inappropriate medication use involving opioids in older adults in Canada	Canada	Longitudinal study	• Potentially inappropriate medication (PIMs) – Beer's criteria<br>• Potentially inappropriate medication use involving opioids (PIOU)	1201 older adults (≥ 65 years)	N/A	• Potentially inappropriate medication use involving opioids is associated with higher costs compared to those observed with opioid use and no use.<br>• There is a need for more effective use of health care resources to reduce costs for the health care system.
Fahmi et al. 2023	Combinations of medicines in patients with polypharmacy aged 65-100 in primary care: Large variability in risks of adverse drug related and emergency hospital admissions.	UK	Longitudinal study	• Use of multiple medicines<br>• Use of ≥5 drugs (within 84 days prior)	89,235 older adults (≥ 65 years)	N/A	• There were over 112,000 different combinations of the 50 medicine classes most implicated in ADR-related hospital admission in the RF models, with the most important medicine classes being loop diuretics, domperidone and/or metoclopramide, medicines, and sulfonamides and/or trimethoprim.<br>• Polypharmacy involves a very large number of different combinations of medicines, with substantial differences in risks of ADR-related and emergency hospital admissions.<br>• Simple tools based on few medicine classes may not be effective in identifying high risk patients.

For peer review only

Häppölä et al. 2020	A data-driven medication score predicts 10-year mortality among aging adults.	Finland	Longitudinal study	Use of numerous potentially interacting medications.	20,078 adults (aged 46-74) in community sites across Finland (mainly North)	N/A	• The resulting score is strongly associated with all-cause mortality (HR 1.18 per point increase in score; 95% CI 1.14–1.22)<br>• When combined with Charlson comorbidity index, individuals had over 6x risk (HR 6.30; 95% CI 3.84–10.3) compared to individuals with a protective score profile.<br>• Alone, the medication score performs similarly to the Charlson comorbidity index
Monterde et al. 2020	Multimorbidity as a predictor of health service utilization in primary care: a registry-based study of the Catalan population.	Spain	Longitudinal study	Prescription of more than 8 drugs over one year	6,102,595 adults (≥18 years) in Catalonia	N/A	• Multimorbidity assessment enhanced prediction of use of healthcare resources at community level<br>• Clinical risk groups had a higher predictive performance for polypharmacy over Charlson Index and Adjusted Morbidity Groups
Villén et al. 2020	Multimorbidity patterns, polypharmacy and their association with liver and kidney abnormalities in people over 65 years of age: a longitudinal study.	Spain	Longitudinal study	concomitant consumption of ≥5 medications	743,827 older adults (65–99 years) in 285 primary health care centres (PHCCs) in Catalonia	N/A	• The most frequently prescribed medicines were related to multimorbidity patterns and their consumption was maintained throughout the follow-up period.<br>• A higher risk of abnormal kidney and liver function was observed in specific multimorbidity patterns.
Guthrie et al. 2015	The rising tide of polypharmacy and drug-drug interactions: population database analysis 1995-2010	UK (Scotland)	Longitudinal analysis (retrospective cohort)	• concomitant prescription of ≥5 or ≥10 drugs<br>• Major or excessive polypharmacy<br>• 3 levels of analysis for polypharmacy as ≥5, ≥10, and ≥15 drugs dispensed in the previous 84 days.<br>• Focus on drug-drug interactions	310,000 adults (≥20 years, mean 48 years) in one region of Scotland	N/A	• Between 1995 and 2010, the proportion of adults dispensed ≥5 drugs doubled to 20.8%, and the proportion dispensed ≥10 tripled to 5.8%.<br>• Receipt of ≥10 drugs was strongly associated with increasing age but was also in people living in more deprived areas or in a care home<br>• The proportion with potentially serious drug-drug interactions more than doubled to 13% of adults in 2010, and the number of drugs was most strongly associated with this
Payne et al. 2014	Is polypharmacy always hazardous? A retrospective cohort analysis using linked electronic health records from primary and secondary care	UK	Longitudinal analysis (retrospective cohort)	• Prescription of multiple medications<br>• Focus on appropriate vs inappropriate polypharmacy	180 815 adults with long-term clinical conditions and numbers of regular medications	N/A	• Admissions were more common in patients on multiple medications, but admission risk varied with the number of conditions.<br>• Unplanned hospitalization is strongly associated with the number of regular medications. However, the effect is reduced in patients with multiple conditions, in whom only the most

For peer review only

							extreme levels of polypharmacy are associated with increased admissions.<br>• Assumptions that polypharmacy is always hazardous and represents poor care should be tempered by clinical assessment of the conditions for which those drugs are being prescribed.
von Buedingen et al. 2018	Changes in prescribed medicines in older patients with multimorbidity and polypharmacy in general practice	German y	Longitudinal analysis (secondary analysis of RCT data)	• Regular use of five or more drugs<br>• Inappropriate prescriptions	505 older adults (median age 72 years) with multimorbidity (≥3 chronic conditions) across 72 general practices in Hesse, Germany.	PRIMUM (The healthcare assistant conducted a checklist-based interview with patients on medication-related problems and reconciled their medications. Then, using a computerised decision support system, the GP optimised medication, with patients)	• Medication regimens in older patients with multimorbidity and polypharmacy changed frequently.<br>• These are mostly due to discontinuations and dosage alterations, followed by additions and restarts.<br>• These findings cast doubt on the effectiveness of cross-sectional assessments of medication and support longitudinal assessments where possible.
Alaa Eddine et al. 2020	A pharmacist-led medication review service with a deprescribing focus guided by implementation science	Lebano n	Longitudinal analysis	• ≥ 5 medicines<br>• Drug related problems and potentially inappropriate prescribing<br>• Focus on deprescribing	143 older adults (≥ 65 years) at a single site (mixed GP/physicians in primary care facility) in Lebanon for low-income patients (receiving free medication)	An pharmacist-led medication review, with recommendation s placed with GPs/ physicians at the facility.	• The intervention pharmacist provided 221 recommendations to physicians, of which 52% were to discontinue one or more medications.<br>• Patients in the intervention group showed significantly higher satisfaction compared to the ones in the control group ($p < 0.001$, effect size = 1.75).<br>• Of all recommendations, 30% were accepted by the physicians.
Vos et al. 2022	Fifteen-year trajectories of multimorbidity and polypharmacy in Dutch primary care—A longitudinal analysis of age and sex patterns.	Netherl ands	Longitudinal analysis	≥5 or more different medications in one year	10,037 patients, all ages from GP practices in South Netherlands	N/A	• Multimorbidity and polypharmacy are common, and their prevalence is accelerating, with a relatively rapid increase in younger groups.<br>• This underlines the need for a longitudinal approach and a life course perspective in patient care.

For peer review only

Novella et al. 2022	Relation between drug therapy-based comorbidity indices, Charlson's comorbidity index, polypharmacy and mortality in three samples of older adults.	Italy	Longitudinal analysis (retrospective cohort)	• Polypharmacy: ≥5 medications<br>• Excessive polypharmacy: ≥10 medications<br>• This definition included systemic and topical drugs	Older adults (all 65+ years) including 2,389 nursing home residents, 4,765 and 633 older adults admitted acutely to geriatric or internal medicine wards	N/A	On the whole, comorbidity indices did not perform well in our three settings, although the highest level of each index was associated with higher mortality.
McCarthy et al. 2022	Patient and general practitioner experiences of implementing a medication review intervention in older people with multimorbidity: Process evaluation of the SPPiRE trial	Ireland	Evaluation (RCT)	• Multiple medicines use<br>• Higher levels of polypharmacy ≥ 15 medicines<br>• Focus on potentially inappropriate prescribing (STOPP/START)	404 older adults (≥65 years) taking ≥15 regular medicines from 51 GP practices	SPPiRE website: educational module template for an individualised patient medication review that identified PIP, deprescribing opportunities, and patient priorities	• The SPPiRE intervention had a small effect in reducing the number of medicines and this was primarily mediated through the brown bag review.<br>• The context of resource shortages and deep-seated views around medical decision-making influenced intervention implementation.<br>• Intervention delivery varied among practices and 45 patients (28%) had no review, primarily due to insufficient GP time. GPs and patients responded positively to the intervention but most GPs did not engage with the patient priority-setting process.<br>• GPs identified a lack of integration into practice software and resources as barriers to future implementation.
Jager et al. 2017	A tailored programme to implement recommendations for multimorbid patients with polypharmacy in primary care practices-process evaluation of a cluster randomized trial	Germany	Evaluation (RCT)	• ≥4 drugs	273 adults (>50 years) suffering from at least 3 chronic diseases, receiving more than 4 drugs, and being at high risk for medication-related events according to the assessment of the treating GP	The tailored programme consisted of a workshop for practice teams, elaboration of implementation action plans, aids for medication reviews, a multilingual info-tool for patients on a tablet PC, posters and	• The most frequently reported effect of the tailored programme was the increase of awareness for the health problem and the recommendations, while implementation of routine processes was only reported for structured medication counselling.<br>• Several modifications of the tailored programme may enhance its effectiveness such as conducting outreach visits instead of a workshop, improved targeting, provision of evidence, integration of tools into the practice software and information materials in tailored formats.

For peer review only

						brown paper bags as reminders for patients.	
Akyon et al. 2023	Artificial intelligence-supported web application design and development for reducing polypharmacy side effects and supporting rational drug use in geriatric patients	Turkiye	Cross-sectional study	• simultaneous use of ≥5 drugs<br>• Focus on potentially inappropriate medications	296 older adults (≥ 65 years)	AI supported web application - auxiliary reference tool for drug–drug, and drug-disease interactions	• While the PIM coverage rate with the proposed tool was 75.3%, the PIM coverage rate of EU(7)-PIM, US-FORTA, TIME-to-STOPP, Beers 2019, STOPP, Priscus criteria in the web application database respectively(63.5%–19.5%) from the highest to the lowest.<br>• PIM criteria alone are insufficient to include actively used medicines and it shows heterogeneity.
Aubert et al. 2016	Polypharmacy and specific comorbidities in university primary care settings.	Switzerland	Cross-sectional study	• ≥ 5 long-term prescribed drugs<br>• potentially inappropriate prescribing and potential prescribing omission (using STOPP/START)	1002 patients aged 50–80 years followed in Swiss university primary care settings	N/A	Polypharmacy is common in university primary care settings, is strongly associated with hypertension, diabetes mellitus, chronic kidney disease and cardiovascular diseases, and increases potentially inappropriate prescribing.
Barrio-Cortes et al. 2023	Differences in healthcare service utilization in patients with polypharmacy according to their risk level by adjusted morbidity groups: a population-based cross-sectional study.	Spain	Cross-sectional study	• ≥ 6 medicines	1598 adults (mean age 82.7)	N/A	• Polypharmacy population compared to non-polypharmacy was characterized by a more advanced age, predominance of women, high-risk, complexity, numerous comorbidities, dependency and remarkable healthcare utilization.<br>• Factors associated with a greater primary care utilization in patients with polypharmacy were elevated complexity, high risk level and dysrhythmia.
Bužančić et al. 2023	Deprescribing in a multimorbid older adult: A case vignette study among community pharmacists and primary care physicians.	Croatia	Cross-sectional study	• ≥5 regular medications concomitantly<br>• ≥10 medications (Hyperpolypharmacy)<br>• Focus on deprescribing	Older adult with taking 16 medications	Case vignette (comparing pharmacist and physician deprescribing acceptance)	• Physicians would accept rationales to deprescribe a median of 10 medicines, while pharmacist recommend a median of six medicines.<br>• Most difference lays in deprescribing of preventative medicines.<br>• Action is needed to improve pharmacists' skills in recognizing deprescribing targets and confidence in making suggestions, which could lead to opening of possibilities for joint patient care.

For peer review only

Calderon-Larranaga et al. 2013	Polypharmacy patterns: unravelling systematic associations between prescribed medications	Spain	Cross-sectional study	• Multiple medicines<br>• Focus on polypharmacy patterns or clusters	79,089 people (≥15 years, mean 47 years)	N/A	• 7 patterns of polypharmacy were identified, which may be classified depending on the type of disease they are intended to treat: cardiovascular, depression-anxiety, acute respiratory infection, chronic obstructive pulmonary disease, rhinitis-asthma, pain, and menopause.<br>• Almost all of the patterns included drugs for preventing or treating potential side effects of other drugs in the same pattern.<br>• It demonstrated the existence of non-random associations in drug prescriptions.
Guisado-Clavero et al. 2019	Medication patterns in older adults with multimorbidity: a cluster analysis of primary care patients.	Spain	Cross-sectional study	Multiple drug use (examined in categories of 1-4, 5-9, and 10+ medications)	164,513 older adults (64-94 years) with multimorbidity (≥2 chronic diseases) within 50 primary healthcare centres in Barcelona	N/A	• Six medication patterns were identified, 5 of which were related to one or more anatomical group, with associations among drugs from different systems.<br>• Overall, guidelines do not accurately reflect the situation of the elderly multimorbid, new strategies for managing multiple drug uses are needed to optimize prescribing in these patients.
Kardas et al. 2023	Optimizing polypharmacy management in the elderly: a comprehensive European benchmarking survey and the development of an innovative online benchmarking application.	Poland, UK and Qatar (Study across all EU)	Cross-sectional study	• Multiple medications<br>• ≥5 medications	Older adults (≥ 65 years)	911 professionals (mostly pharmacists, GPs, consultants nurses and other stakeholders) from all but 2 EU countries	• Out of the survey participants, 496 (54.4%) reported availability of various activities or formal programs targeting polypharmacy in the elderly that were known to them.<br>• These programs had multiple goals, of which improved patient safety was indicated as the most common objective (65.1% of the cases).<br>• The most typical settings for such programs was primary care (49.4%), with pharmacists and primary care doctors being indicated most often as those providing the programs (61.7% and 35.5% of cases, respectively).<br>• Vast majority of programs applied diverse forms of drug reviews.
King et al. 2023	Potentially Inappropriate Medication Use among Underserved Older Latino Adults.	USA	Cross-sectional study	• ≥5 medications<br>• Focus on potentially inappropriate medication (PIM) – Beer's criteria	126 older adults (≥ 65 years) living in 14 mostly Latino senior	N/A	• One-third of participants had at least one PIM.<br>• Polypharmacy (≥5 medications) was observed in 55% of our sample. In addition, 46% took drugs to be used with caution (UWC).

For peer review only - http://bmjopen.bmj.com/site/about/guidelines.xhtml

					centers and housing units		• In total, 16% were taking between 9 and 24 medications, whereas 39% and 46% were taking 5 to 8 and 1 to 4 prescription medications.<br>• The multinomial logit regression analysis showed that (controlling for demographic variables) increased PIM use was associated with an increased number of prescription medications, number of chronic conditions, sleep difficulty, lack of access to primary care, financial strains, and poor self-rated health.
Lopez-Rodriguez et al. 2015	Potentially inappropriate prescriptions according to explicit and implicit criteria in patients with multimorbidity and polypharmacy. MULTIPAP: A cross-sectional study	Spain	Cross-sectional study	• Simultaneous use of several medicines.<br>• Potentially inappropriate medications (using both explicit and implicit criteria)	593 community-dwelling elderly aged 65 to 74 years, with multimorbidity and polypharmacy	N/A	• Potentially inappropriate prescribing was detected in 57.7%, 43.6%, 68.8% and 71% of 50 patients according to the explicit criteria STOPP 2014, STOPP 2008, Beers 2019 and Beers 2015 respectively.<br>• The MAI criteria detected greater inappropriateness than did the explicit criteria, but their application was more complex and difficult to automate.<br>• For every new drug taken by a patient, the MAI score increased by 2.41 (95% CI 1.46; 3.35) points.<br>• Diabetes, ischaemic heart disease and asthma were independently associated with lower summated MAI scores.
Lozano-Hernandez et al. 2020	Social support, social context and nonadherence to treatment in young senior patients with multimorbidity and polypharmacy followed-up in primary care. MULTIPAP Study	Spain	Cross-sectional study	• Simultaneous consumption of ≥ 5 drugs<br>• Potentially inappropriate medication<br>• Focus on adherence	593 older adults (between 65-74 years) with multimorbidity (≥3 diseases) and polypharmacy (≥5 drugs) during the last three months	MULTIPAP intervention: GPs e-training and GP-patient-centred interview.	• Among patients 65-74 years of age with multimorbidity and polypharmacy, lower functional support was related to nonadherence to treatment<br>• The nonadherence decreased in those patients with higher functional support, lower urban vulnerability, and higher perceived health status according to the visual analog scale of health-related quality of life
Manirajan et al. 2023	Drug Utilisation Review among Geriatric Patients with Noncommunicable Diseases in a Primary Care Setting in Malaysia.	Malaysia	Cross-sectional study	• ≥5 more medications<br>• ≥10 = excessive medications	310 older adults (≥ 65 years) in one primary care clinic in Malaysia	N/A	• Combination therapy was prescribed to more than 97% (n = 302) of the elderly, whereas cardiovascular and endocrine medications were the most common.<br>• 10 prescriptions were found to have drug-related problems, prescribing cascade (80%), lack of medicine optimisation (10%), and inappropriate prescription (10%).

For peer review only - http://bmjopen.bmj.com/site/about/guidelines.xhtml

Moreira et al. 2020	Polypharmacy among adult and older adult users of primary care services delivered through the Unified Health System in Minas Gerais, Brazil.	Brazil	Cross-sectional study	≥5 medications	1,159 interviewees distributed across 104 cities and 253 primary healthcare services	N/A	Understanding the medication use profile in primary health care allows identifying groups of people that are more likely to experience polypharmacy, and planning measures to mitigate potential polypharmacy-related problems.
Neuner-Jehle et al. 2017	Patient–provider concordance in the perception of illness and disease: a cross-sectional study among multimorbid patients and their general practitioners in Switzerland	Switzerland	Cross-sectional study	• Multiple medications<br>• Focus on concordance, complexity and patient-centeredness	334 older adults (≥60 years, mean 76.2) with multimorbidity across 46 GPs in North Switzerland	N/A	• A majority of GPs perceive the CCs of the multimorbid patients correctly, but there is room for improvement.<br>• Concordance between CCs and diagnosis was 53.6%.<br>• The younger age and higher intake of drugs were significantly associated with an increased concordance between CCs and diagnosis.
Ong et al. 2018	Variation of polypharmacy in older primary care attenders occurs at prescriber level	Malaysia	Cross-sectional study	• Concomitant use of≥ 5 medications<br>• Inappropriate medication use	22,832 Older adults (≥65 years) attending 2914 primary care clinics in Malaysia (nationwide)	N/A	• A total of 20.3% of the older primary care attenders experienced polypharmacy (26.7%% in public and 11.0% in private practice).<br>• The adjusted odds ratio (OR) of polypharmacy were 6.37 times greater in public practices. Polypharmacy was associated with patients of female gender (OR 1.49), primary education level (OR 1.61) and multimorbidity (OR 14.21).<br>• The variation in rate of polypharmacy was mainly found at prescriber level.
Ose et al. 2012	Let's talk about medication: concordance in rating medication adherence among multimorbid patients and their general practitioners	Germany	Cross-sectional study	• Multiple medications use<br>• Focus on adherence (self-rated)	92 patients with multiple chronic conditions at high-risk of hospitalisation (subjective) across 10 primary care practices from a region in Germany	N/A	• The percentage of concordance ranges between 40% (forgot to take medication) and 61% (deliberately omitted a dose).<br>• Talking about medication on a regular basis and better continuity of care may enhance patient – provider concordance in rating medication adherence as a prerequisite for shared decisions concerning medication in patients with multiple chronic conditions.
Rieckert et al. 2018	Polypharmacy in older patients with chronic diseases: a cross-sectional analysis of	Austria, Germany, Italy, and UK	Cross-sectional study	excessive polypharmacy (taking ≥10 substances daily).	3904 older adults (aged 75+)	N/A	Frailty, multimorbidity, obesity, and decreased physical as well as mental health status are risk factors for excessive polypharmacy. Sex,

For peer review only

	factors associated with excessive polypharmacy.						educational level, and smoking apparently do not seem to be related to excessive polypharmacy.
Rogero-Blanco et al. 2020	Use of an Electronic Clinical Decision Support System in Primary Care to Assess Inappropriate Polypharmacy in Young Seniors With Multimorbidity: Observational, Descriptive, Cross-Sectional Study	Spain	Cross-sectional study	Focus on potentially inappropriate medications (Beers and STOPP/START)	593 older adults (aged 65-75) with multimorbidity (≥3 diseases) and polypharmacy (≥5 medications)	N/A	• There is a high prevalence of potentially inappropriate medications<br>• Females, taking a greater number of medicines, working in the primary sector, and being prescribed drugs for the central nervous system were related to a higher frequency<br>• The most common potentially inappropriate medications included benzodiazepines and prolonged use of proton pump inhibitors
Rogero-Blanco et al. 2021	Drug interactions detected by a computer-assisted prescription system in primary care patients in Spain: MULTIPAP study	Spain	Cross-sectional study	• Simultaneous consumption of ≥ 5 drugs<br>• Potentially inappropriate medication<br>• Focus on drug-drug interactions	593 older adults (between 65-74 years) with multimorbidity (≥3 diseases) and polypharmacy (≥5 drugs) during the last three months	MULTIPAP intervention: GPs e-training and GP-patient-centred interview.	• Half of the patients had at least one relevant drug interaction<br>• Factors associated with drug interactions were the use of more than 10 drugs (OR 11.86) and having anxiety/depressive disorder (OR 1.98), with protective factors against as hypertension, diabetes, and ischaemic heart disease
Sirois et al. 2019	The delicate choice of optimal basic therapy for multimorbid older adults: A cross-sectional survey.	Canada	Cross-sectional study	• Many medications<br>• ≥10 medications	Older adults (65–75 years) with type 2 diabetes, chronic obstructive pulmonary disease and heart failure	N/A	• At least half of the participants (pharmacists and geriatricians) considered polypharmacy (≥10 medications) inevitable for an optimal basic treatment of DM, COPD and HF.<br>• The heterogeneity of responses raises issues when considering quality indicators in population-based studies.
Troncoso-Marino et al. 2021	Medication-related problems in older people in Catalonia: A real-world data study.	Spain	Cross-sectional study	Focus on medication-related problems<br>• Duplicate therapy<br>• Drug-drug interactions<br>• Potentially inappropriate medications (Beers, STOPP/START, PRISCUS)	853 085 older adults (aged 65-99) with multimorbidity in 284 primary health care centres in Catalonia	N/A	• The coexistence of multimorbidity and polypharmacy is associated with an elevated risk of medication-related problems<br>• The most common potentially inappropriate drugs were those that increase the risk of fall (66.8%), antiulcer agents without criteria for gastroprotection (40.6%), and the combination of drugs with anticholinergic effects (39.7%).

For peer review only

				• Drugs contraindicated in chronic kidney disease and in liver diseases			
Carrier et al. 2019	GPs' management of polypharmacy and therapeutic dilemma in patients with multimorbidity: a cross-sectional survey of GPs in France	France	Cross-sectional study	• Many drugs at the same time or an excessive number<br>• No consensus on threshold or temporality<br>• Focus on unfavourable risk–benefit and potentially inappropriate prescribing	Older adults (54-82 years) with multimorbidity	Survey amongst 1266 GPs	• Nearly all (91.4%) responders felt comfortable or fairly comfortable deprescribing inappropriate medications, but only 34.7% decided to do so often or very often<br>• In therapeutic dilemmas, some GPs choose to prioritise patients' requests over iatrogenic risks.<br>• GPs need pragmatic implementation tools for handling therapeutic dilemmas, and to improve their skills in medication management and patient engagement in such situations.
Cooper et al. 2016	Potentially inappropriate prescribing in two populations with differing socio-economic profiles: a cross-sectional database study using the PROMPT criteria.	UK (NI), Ireland	Cross-sectional study	• ≥4 repeat medicines<br>• Focus on Potentially inappropriate prescribing (PROMPT)	>750,000 middle-aged adults (45–64 years) in 2 populations with differing socio-economic profiles in Ireland	N/A	• Age group, female gender and polypharmacy were significantly associated with PIP in both populations and polypharmacy had the strongest association.<br>• PIP is common amongst middle-aged people with the risk of PIP increasing with polypharmacy.<br>• Differences in the prevalence of polypharmacy and PIP between the two populations may relate to heterogeneity in healthcare services and different socio-economic profiles, with higher rates of multimorbidity and associated polypharmacy in more deprived groups.
Gutierrez-Valencia et al. 2019	Prevalence of polypharmacy and associated factors in older adults in Spain: Data from the National Health Survey 2017	Spain	Cross-sectional study	• polypharmacy (≥ 5 medications)<br>• hyperpolypharmacy (≥10)	7023 older adults (aged 65+) from National Health Survey of Spain	N/A	• The prevalence of polypharmacy in the elderly in primary care continues to increase and could be widely underestimated.<br>• Factors such as functional capacity or geriatric syndromes, fundamental in elderly people, modulate the habits of consumption and prescription of drugs in this population.
Payne et al 2014	Prevalence of polypharmacy in a Scottish primary care population	UK (Scotland)	Cross-sectional	• multiple medications by a single patient<br>•	180,815 adult (≥20 years) patients permanently registered in 40	N/A	• Polypharmacy is common and significantly associated with multimorbidity, although considerable variation exists between different conditions.

For peer review only

					Scottish GP surgeries.		• The impact of clinical conditions on the number of medicines is generally less in the presence of co-existing concordant conditions.<br>•
Khatter et al. 2020	Prevalence and predictors of potentially inappropriate prescribing in middle-aged adults: a repeated cross-sectional study	UK	Cross sectional study	• ≥4 repeat medicines<br>• Focus on Potentially inappropriate prescribing (PROMPT)	>50,000 middle aged adults (45–64 years taking ≥1 medicine.	N/A	• The prevalence of PIP decreased from 20% in 2014 to 18% in 2019.<br>• The most prevalent PROMPT criteria were the use of ≥2 drugs from the same pharmacological class (7.6%), use of non-steroidal anti-inflammatory drugs for >3 months (7.1%) and use of proton pump inhibitors above recommended maintenance dosages for >8 weeks (3.1%).<br>• Over the study period, the prevalence of multimorbidity increased (47–52%) and polypharmacy was stable (27%).<br>• Polypharmacy, multimorbidity, deprivation, and age were independently associated with PIP. Sex was the only variable not associated.
Molist-Brunet et al. 2022	Improving individualized prescription in patients with multimorbidity through medication review	Spain	Before and after study	• ≥5 medications continuously<br>• severe or excessive polypharmacy: ≥10 chronic medications	428 older adults (≥65 years, mean age 85.5) with multimorbidity (≥2 morbidities) across 3 primary care centres and 3 nursing homes.	Medication review by an interdisciplinary team (primary care team, consultant geriatrician and clinical pharmacist) by applying the Patient-Centered Prescription model (based on CGA) to align the treatment with care goals.	• An individualized medication review in frail older patients, applying the Patient-Centered Prescription model, decreases pharmacological parameters related to adverse drug effects, such as polypharmacy, therapeutical complexity, and anticholinergic and, or sedative burden.<br>• A decrease in polypharmacy, medication regimen complexity index, and drug burden index was more frequent among frail patients, especially those with severe frailty
San-Jose et al. 2021	Integrated health intervention on polypharmacy and inappropriate prescribing in elderly people with multimorbidity: Results	Spain	Before and after study	• ≥ 5 medicines<br>• Focus on potentially inappropriate prescribing (STOPP/START)	100 older adults (≥ 65 years) with high multimorbidity according to Adjusted Morbidity Groups (AMG 3-4) with a	A CGA and appropriate prescribing review of medicines were performed by the team (one GP,	• An integrated health intervention centred on polypharmacy in elderly people improves inappropriate prescribing that persists beyond the intervention.<br>• The proportion of patients with two or more STOPP criteria reduced from 37% at the beginning of the intervention to 18% at the end,

	at the end of the intervention and at 6 months after the intervention				recent unplanned hospital visit (including ED)	one medical doctor, an ANP and a day nurse), then a care plan was developed.	and the proportion of those with START criteria from 13% to 6%. These differences persisted at 6 months.
Basger et al. 2012	Validation of prescribing appropriateness criteria for older Australians using the RAND/UCLA appropriateness method	Australia	Consensus study	• Use of multiple medications<br>• Focus on prescribing appropriateness	N/A	N/A	• A set of 41 Australian prescribing appropriateness criteria were validated by an expert panel.<br>• Use of these criteria, together with clinical judgement and other medication review processes such as patient interview, is intended to assist in improving patient care by efficiently detecting potential drug related problems related to commonly occurring medicines
Burt et al. 2018	Developing a measure of polypharmacy appropriateness in primary care: systematic review and expert consensus study.	UK	Consensus study (with review)	• Use of multiple medications in a single individual<br>• Focus on polypharmacy appropriateness	N/A	N/A	• Produced a set of 12 indicators of clinical importance considered relevant to polypharmacy appropriateness.<br>• Panel members particularly valued indicators concerned with adverse drug reactions, contraindications, drug-drug interactions, and the conduct of medication reviews.
Cooper et al. 2014	The development of the PROMPT (PRescribing Optimally in Middle-aged People's Treatments) criteria	UK and Ireland	Consensus study (with review)	Focus on potentially inappropriate prescribing	Middle-aged adults (45-64 years)	N/A	• PROMPT is the first set of prescribing criteria developed for use in middle-aged adults<br>• A final list of 22 criteria reached consensus after two rounds
George et al. 2004	Development and Validation of the Medication Regimen Complexity Index	Australia	Consensus study (with review)	• Use of multiple medications<br>• Focus on complexity	N/A	N/A	• A 65–item Medication Regimen Complexity Index (MRCI) was developed.<br>• The total MRCI score had significant correlation with the number of drugs in the regimen, but not with the age and gender of the patients.
Mann et al. 2022	Development of a deprescribing manual for frail older people for use in the COFRAIL study and in primary care	Germany	Consensus study	• ≥5 medications<br>• Focus on deprescribing	Frail older patients (≥65 years	Structured manual to support deprescribing of medicines in frail, older adults in primary care	• After piloting and revisions, the deprescribing manual now covers 11 indications/topics.<br>• In each chapter, patient- and medication-related deprescribing criteria, monitoring and communication strategies, and information about concerns related to the use of specific drugs in older patients are provided.
Turner et al. 2016	What factors are important for	Australia	Consensus study	• ≥5 medications	Residents of long-term care	Nominal group methods with 19	• No two groups had the same priorities. GPs ranked 'evidence for deprescribing' and

For peer review only

	deprescribing in Australian long-term care facilities? Perspectives of residents and health professionals			• Appropriate and inappropriate prescribing<br>• Focus on deprescribing of unnecessary or potentially inappropriate medications	facilities with multimorbidity and polypharmacy across metropolitan and regional South Australia.	GPs, 12 nurses, 14 pharmacists, and 11 patients and carers	'communication with family/resident' as most important factors<br>• Pharmacists ranked 'clinical appropriateness of therapy' and 'identifying residents' goals of care' as most important.<br>• The multidisciplinary groups prioritised both 'adequacy of medical and medication history' and 'identifying goals of care'.

For peer review only

Preferred Reporting Items for Systematic reviews and Meta-Analyses extension for Scoping Reviews (PRISMA-ScR) Checklist for:

Understanding, identifying, and addressing problematic polypharmacy within multimorbidity in primary care: A scoping review

SECTION	ITEM	PRISMA-ScR CHECKLIST ITEM	REPORTED ON PAGE #
**TITLE**			
Title	1	Identify the report as a scoping review.	1
**ABSTRACT**			
Structured summary	2	Provide a structured summary that includes (as applicable): background, objectives, eligibility criteria, sources of evidence, charting methods, results, and conclusions that relate to the review questions and objectives.	2
**INTRODUCTION**			
Rationale	3	Describe the rationale for the review in the context of what is already known. Explain why the review questions/objectives lend themselves to a scoping review approach.	4-5
Objectives	4	Provide an explicit statement of the questions and objectives being addressed with reference to their key elements (e.g., population or participants, concepts, and context) or other relevant key elements used to conceptualize the review questions and/or objectives.	5
**METHODS**			
Protocol and registration	5	Indicate whether a review protocol exists; state if and where it can be accessed (e.g., a Web address); and if available, provide registration information, including the registration number.	N/A
Eligibility criteria	6	Specify characteristics of the sources of evidence used as eligibility criteria (e.g., years considered, language, and publication status), and provide a rationale.	6-7
Information sources*	7	Describe all information sources in the search (e.g., databases with dates of coverage and contact with authors to identify additional sources), as well as the date the most recent search was executed.	5-6
Search	8	Present the full electronic search strategy for at least 1 database, including any limits used, such that it could be repeated.	5-6
Selection of sources of evidence†	9	State the process for selecting sources of evidence (i.e., screening and eligibility) included in the scoping review.	7-8
Data charting process‡	10	Describe the methods of charting data from the included sources of evidence (e.g., calibrated forms or forms that have been tested by the team before their use, and whether data charting was done independently or in duplicate) and any processes for obtaining and confirming data from investigators.	8
Data items	11	List and define all variables for which data were sought and any assumptions and simplifications made.	8
Critical appraisal of individual sources of evidence§	12	If done, provide a rationale for conducting a critical appraisal of included sources of evidence; describe the methods used and how this information was used in	6

St. Michael's
Inspired Care.
Inspiring Science.

For peer review only - http://bmjopen.bmj.com/site/about/guidelines.xhtml

SECTION	ITEM	PRISMA-ScR CHECKLIST ITEM	REPORTED ON PAGE #
		any data synthesis (if appropriate).	
Synthesis of results	13	Describe the methods of handling and summarizing the data that were charted.	8
**RESULTS**			
Selection of sources of evidence	14	Give numbers of sources of evidence screened, assessed for eligibility, and included in the review, with reasons for exclusions at each stage, ideally using a flow diagram.	8, figure 1
Characteristics of sources of evidence	15	For each source of evidence, present characteristics for which data were charted and provide the citations.	Supplementary file 1
Critical appraisal within sources of evidence	16	If done, present data on critical appraisal of included sources of evidence (see item 12).	N/A
Results of individual sources of evidence	17	For each included source of evidence, present the relevant data that were charted that relate to the review questions and objectives.	Supplementary file 1
Synthesis of results	18	Summarize and/or present the charting results as they relate to the review questions and objectives.	8-19
**DISCUSSION**			
Summary of evidence	19	Summarize the main results (including an overview of concepts, themes, and types of evidence available), link to the review questions and objectives, and consider the relevance to key groups.	19-20
Limitations	20	Discuss the limitations of the scoping review process.	21-22
Conclusions	21	Provide a general interpretation of the results with respect to the review questions and objectives, as well as potential implications and/or next steps.	22
**FUNDING**			
Funding	22	Describe sources of funding for the included sources of evidence, as well as sources of funding for the scoping review. Describe the role of the funders of the scoping review.	23

JBI = Joanna Briggs Institute; PRISMA-ScR = Preferred Reporting Items for Systematic reviews and Meta-Analyses extension for Scoping Reviews.
* Where *sources of evidence* (see second footnote) are compiled from, such as bibliographic databases, social media platforms, and Web sites.
† A more inclusive/heterogeneous term used to account for the different types of evidence or data sources (e.g., quantitative and/or qualitative research, expert opinion, and policy documents) that may be eligible in a scoping review as opposed to only studies. This is not to be confused with *information sources* (see first footnote).
‡ The frameworks by Arksey and O'Malley (6) and Levac and colleagues (7) and the JBI guidance (4, 5) refer to the process of data extraction in a scoping review as data charting.
§ The process of systematically examining research evidence to assess its validity, results, and relevance before using it to inform a decision. This term is used for items 12 and 19 instead of "risk of bias" (which is more applicable to systematic reviews of interventions) to include and acknowledge the various sources of evidence that may be used in a scoping review (e.g., quantitative and/or qualitative research, expert opinion, and policy document).

From: Tricco AC, Lillie E, Zarin W, O'Brien KK, Colquhoun H, Levac D, et al. PRISMA Extension for Scoping Reviews (PRISMAScR): Checklist and Explanation. Ann Intern Med. 2018;169:467–473. doi: 10.7326/M18-0850.
